# Electrochemical Three‐component Synthesis of Alkenesulfonates from Cinnamic Acids, SO_2_, and Alcohols

**DOI:** 10.1002/cssc.202500186

**Published:** 2025-04-16

**Authors:** Po‐Chung Chien, Florian A. Breitschaft, Harald Kelm, Siegfried R. Waldvogel, Georg Manolikakes

**Affiliations:** ^1^ Department of Chemistry RPTU Kaiserslautern‐Landau Erwin‐Schrödinger‐Str. 54 D‐67663 Kaiserslautern Germany; ^2^ Department of Electrosynthesis Max Planck Institute for Chemical Energy Conversion Stiftstraße 34–36 45470 Mülheim an der Ruhr Germany

**Keywords:** alkenesulfonates, decarboxylative functionalization, electrochemistry, multicomponent reactions, sulfur dioxide

## Abstract

A novel, electrochemical three‐component reaction for the synthesis of alkyl alkenesulfonates from cinnamic acids, SO_2_, and alkyl alcohols is reported. This metal‐free process employs inexpensive and readily available graphite electrodes in combination with easy‐to‐use stock solutions of SO_2_ and enables a straightforward construction of the styrene sulfonate scaffold via a decarboxylative transformation. Mechanistic studies indicate a pseudo‐Kolbe type reaction. This novel reaction pathway enables a regioselective synthesis of alkenesulfonates from substituted cinnamic acids without double‐bond translocation. Gram‐scale and anolyte reusability experiments demonstrate the applicability of this process for the construction of alkenesulfonates from cinnamic acids as potential biogenic feedstock.

## Graphical abstract

An electrochemical three‐component synthesis of alkyl alkenesulfonates from cinnamic acids, SO_2_, and alkyl alcohols is reported. This metal‐free process employs inexpensive and readily available graphite electrodes in combination with easy‐to‐use stock solutions of SO_2_ and enables a straightforward decarboxylative preparation of styrene sulfonates via a pseudo‐Kolbe type pathway.

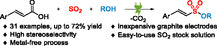

## 1 Introduction

The toxic and corrosive gas sulfur dioxide (SO_2_) is both, an important feedstock for the chemical industry, produced annually on a million‐ton scale,^[^ ^1^ ^]^ and a major air pollutant with severe impacts on environment and health.^[^ ^2^ ^]^ The direct incorporation of SO_2_ into organic molecules enables an efficient and modular synthesis of various interesting scaffolds bearing different sulfonyl (—SO_2_—)‐based functional groups, such as sulfones or sulfonamides.^[^ ^3^, ^4^, ^5^ ^]^ The introduction of easy‐to‐handle SO_2_ surrogates, in particular the solid DABCO‐bis(sulfur dioxide) adduct, DABSO (1,4‐diazabicyclo[2.2.2]octane bis(sulfur dioxide) adduct),^[^ ^6^, ^7^ ^]^ has paved the way for the development of novel approaches for the synthesis of sulfur‐containing molecules for potential applications in agrochemistry, drug development, or materials sciences.^[^ ^8^, ^9^, ^10^, ^11^ ^]^ Among the various methods established in recent years, approaches based on radical transformations are particularly well‐suited for the incorporation of SO_2_ in organic molecules.^[^ ^12^, ^13^ ^]^ In this regard, both photo‐ and electrochemistry are enabling tools for the development of novel, sustainable processes for the direct fixation of SO_2_ into value‐added products.^[^ ^14^, ^15^ ^]^ In particular, electrochemical reactions have greatly expanded the toolbox of synthetic chemists. In many cases, the otherwise necessary prefunctionalization of the substrates and redox reagents can be omitted, which leads to a minimized amount of reagent waste, thus, simplifies the work‐up and costs.^[^ ^16^, ^17^, ^18^, ^19^, ^20^ ^]^ Recently, the groups of Waldvogel, Han, and Ye have described the electrochemical dehydrogenative synthesis of sulfonates,^[^ ^21^, ^22^, ^23^ ^]^ sulfonamides,^[^ ^24^, ^25^, ^26^ ^]^ and sulfamides.^[^ ^27^ ^]^

**Figure 1:**
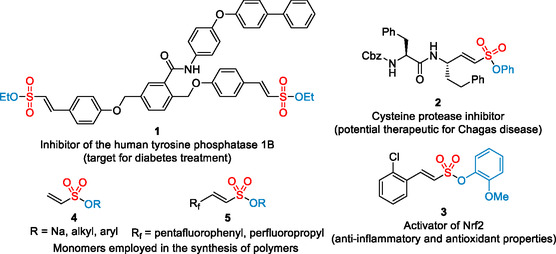
Representative examples of relevant alkenesulfonates.

Among the different sulfonyl‐based molecular entities, alkenesulfonates play an important role. The alkenesulfonate motif can be found in molecules with interesting biological features. Three representative examples, the styrene sulfonate **1**,^[^ ^28^, ^29^ ^]^ an inhibitor of the human tyrosine phosphatase 1B (target for diabetes treatment), the cysteine protease inhibitor **2** (potential therapeutic for Chagas disease),^[^ ^30^ ^]^ and the nuclear factor E2‐related factor‐2 (Nrf2) activator **3** (anti‐inflammatory and antioxidant properties)^[^ ^31^ ^]^ are depicted in **Figure** **1**. Furthermore, alkenesulfonates, such as **4** or **5**, are common monomers employed in the synthesis of polymers containing sulfonate groups attached directly to the polymer backbone.^[^ ^32^ ^]^ These polymers display interesting properties and have found application in blood‐compatible materials^[^ ^33^ ^]^ or proton exchange membranes.^[^ ^34^ ^]^

**Scheme 1:**
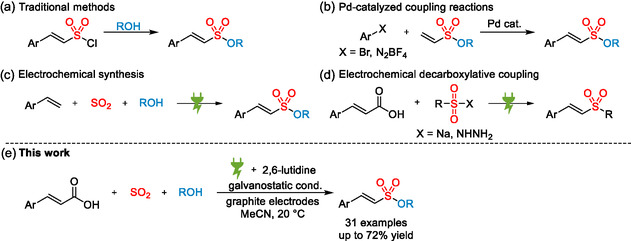
Selected approaches toward *β* ‐styryl sulfones and *β* ‐styryl sulfonates.

Traditional methods for the synthesis of alkenesulfonates utilize the corresponding vinyl sulfonyl chlorides (**Scheme** **1**a).^[^ ^35^, ^36^ ^]^ These key intermediates can be prepared by addition of sulfur trioxide^[^ ^37^ ^]^ or sulfuryl chloride to an olefin.^[^ ^38^ ^]^ More contemporary processes are based on palladium‐catalyzed coupling reactions of vinyl sulfonates with aryl bromides,^[^ ^39^ ^]^ aryldiazonium salts,^[^ ^40^ ^]^ or sulfur‐fluoride‐exchange (SuFEx) chemistry employing the corresponding vinyl sulfonyl fluorides (Scheme 1b).^[^ ^41^ ^]^ Although the latter two methods do not require harsh conditions or reagents associated with the traditional approaches, they rely exclusively on vinyl building blocks already containing a sulfonyl functionality, which necessitates additional synthetic efforts. In 2024, the Waldvogel group reported an electrochemical multicomponent synthesis of alkenesulfonates from styrenes, alcohols, and SO_2_, which enables a direct construction of the sulfonate functionality from two simple organic starting materials (Scheme 1c).^[^ ^42^, ^43^, ^44^ ^]^ However, this dehydrogenative anodic transformation exhibits a limited scope. In the last years, decarboxylative coupling reactions of carboxylic acids have emerged as a powerful tool for the construction of C—C and C–heteroatom bonds.^[^ ^45^, ^46^, ^47^, ^48^, ^49^ ^]^ Decarboxylative transformations of cinnamic acids enable the efficient synthesis of various functionalized styrene derivatives.^[^ ^50^, ^51^ ^]^ Numerous groups have disclosed methods for the decarboxylative coupling of cinnamic acids with sodium sulfinates or sulfonyl hydrazides for the synthesis of vinyl sulfones,^[^ ^52^, ^53^, ^54^ ^]^ including an electrochemical process from the Huang and Wang groups (Scheme 1d).^[^ ^55^, ^56^ ^]^ To the best of our knowledge, decarboxylative sulfonylations of cinnamic acids with direct SO_2_ incorporation have not yet been reported. However, such processes would offer an attractive approach for the direct transformations of cinnamic acids as biobased, renewable feedstock^[^ ^57^, ^58^ ^]^ into value‐added sulfonyl‐compounds.

Herein, we describe a so far unprecedented electrochemical three‐component reaction for the synthesis of alkenesulfonates based on the decarboxylation of cinnamic acids with simultaneous incorporation of SO_2_ (Scheme 1e). This novel approach features the use of easy‐to‐handle SO_2_ stock solutions and inexpensive graphite electrodes and affords *β*‐styryl sulfonates with CO_2_ and H_2_ as the only formed byproducts.

## 2 Results and Discussion

### 2.1 Optimization of the Reaction Conditions

We started our investigations with the electrolysis of cinnamic acid (**6a**), a SO_2_ stock solution (5.0 M in MeCN, see SI, Supplementary Information, for further details), and neopentyl alcohol (**7a**) together with an auxiliary base and tetrabutylammonium salts as supporting electrolyte using different anode materials in a divided setup (**Table** **1**). For the cathodic reaction, a catholyte consisting of the tetrabutylammonium salt as supporting electrolyte together with 5 equiv. of AcOH as proton source for hydrogen evolution^[^ ^59^, ^60^, ^61^ ^]^ in combination with the same electrode materials as for the anodic reaction was used. Best yields were obtained with inexpensive graphite electrodes, *n*Bu_4_NPF_6_ (0.1 M) as supporting electrolyte, a current density of 10 mA cm^−2^, and an amount of applied charge of 3.5 *F*, leading to the alkenesulfonate **8a** in 70% isolated yield (entry 1). Whereas the use of pyridine, 2,4,6‐collidine, or 1,8‐diazabicyclo[5.4.0]undec‐7‐ene (DBU), as auxiliary base afforded the sulfonate product in slightly decreased yields of 54%–62% (entries 2–4). Simple amine bases, such as *N*,*N*‐diisopropylethylamine (DIPEA), led to the desired product only in trace amounts, presumably due to competing anodic oxidation of the amine itself (entry 5). The *n*Bu_4_NPF_6_ with a concentration of 0.1 M proved to be the optimal supporting electrolyte. Increased or decreased concentrations of *n*Bu_4_NPF_6_ as well as other supporting electrolytes, such as *n*Bu_4_NBF_4_, resulted in lower yields (entries 6–8). The use of other electrode materials commonly used in electrochemical SO_2_ fixation,^[^ ^24^, ^25^, ^26^, ^27^, ^42^ ^]^ for example, glassy carbon, platinum, or boron‐doped diamond (BDD), furnished the styrene sulfonate **8a** in lower yields of 25%–46% (entries 9–11). Modulation of the current density (5 or 15 mA cm^−2^) or the applied amount of charge (3.0 or 3.8 *F*) led to decreased yields of 54%–65% (entries 12–15). Best yields were obtained with 3.0 equiv. of neopentyl alcohol (**7a**), 6.0 equiv. of the 2,6‐lutidine, and 10.0 equiv. of SO_2_ (entry 1). Lowering or increasing the amount of alcohol, base, or SO_2_ furnished product **8a** in diminished yields (entries 16–21). As already reported before, DABSO is unfortunately unsuitable for electrosynthesis due to the quite low oxidation potential of DABCO (1,4‐diazabicyclo[2.2.2]octan), leading to competitive anodic oxidation processes.^[^ ^15^ ^]^ Finally, no product formation was observed in the absence of a base or without the application of electric current (entries 22 and 23). Noteworthy, selective formation of the *E*‐alkenesulfonate was observed in all cases.^[^ ^62^ ^]^

**Table 1:** Influence of different parameters on the reaction outcome.

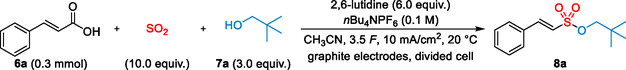					
Entry	Deviation from the standard conditions	Yield [%]a)	Entry	Deviation from the standard conditions	Yield [%]a)
1	None	71 (70)b)	12	5 mA cm^−2^	55
2	Pyridine	54	13	15 mA cm^−2^	65
3	2,4,6‐Collidine	55	14	3.0 *F*	63
4	DBU	62	15	3.8 *F*	54
5	DIPEA	Traces	16	Neopentyl alcohol (2.0 equiv.)	50
6	*n*Bu_4_NPF_6_ (0.2 M)	43	17	Neopentyl alcohol (4.0 equiv.)	47
7	*n*Bu_4_NPF_6_ (0.05 M)	58	18	2,6‐Lutidine (4.0 equiv.)	43
8	*n*Bu_4_NBF_4_	50	19	2,6‐Lutidine (8.0 equiv.)	49
9	Glassy carbon electrodes	46	20	SO_2_ (7.5 equiv.)	63
10	Pt foil electrodes	40	21	SO_2_ (12.5 equiv.)	68
11	BDD electrodes	25	22	No base	0
			23	No electric current	0

a) ^1^H NMR yield with the use of CHPh_3_ as the internal standard.

b) Isolated yield.

### 2.2 Scope of the Reaction

**Scheme 2:**
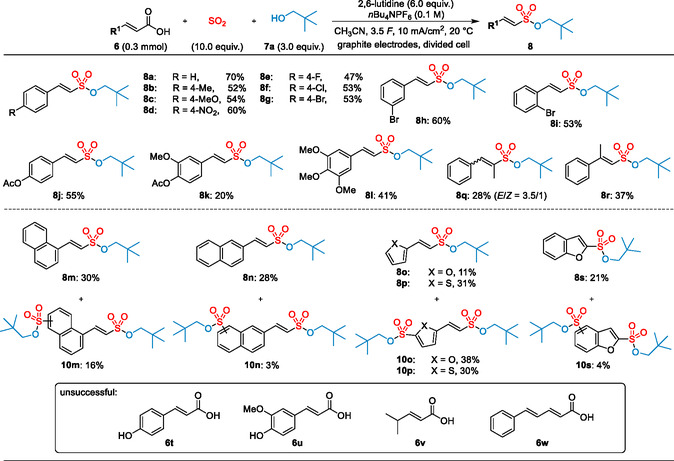
Scope of cinnamic acids and related substrates for the synthesis of alkenesulfonates **8** and **10**. Isolated yields are shown.

With the optimized conditions, the scope of the reaction was explored with different cinnamic acids (**Scheme** **2**). In general, both electron‐donating and electron‐withdrawing substituents on the aromatic core were well tolerated, affording the alkenesulfonates **8a**–**8i** in 47%–70% yield. Reactions with halogenated cinnamic acids **6e**–**6i** furnished the desired products **8e**–**8i** in 47%–60% yield, without a significant influence of the substitution pattern. Both electron‐rich (**6b** or **6c**) and electron‐poor substrates (**6d**) underwent a smooth decarboxylative sulfonylation to the desired alkenesulfonates in comparable yields of 52%–60%. Even a sensitive nitro functionality (**6d**) was well tolerated. Unfortunately, reactions with hydroxy cinnamic acids, such as **6t** and **6u**, did not yield the desired product, presumably due to oxidative degradation of the phenolic core.^[^ ^59^, ^60^, ^61^ ^]^ On the contrary, transformations of different hydroxycinnamic acid derivatives without free OH‐functionalities, such as **6j** (from *p*‐hydroxycinnamic acid), **6k** (from ferulic acid), or **6l**, (from sinapinic acid), afforded the corresponding alkenesulfonates **8j**–**8l** in 20%–55% yield. Since hydroxycinnamic acids can be easily obtained from lignocellulosic biomass,^[^ ^57^, ^58^ ^]^ our novel process provides an attractive opportunity to access interesting alkenesulfonates from renewable feedstocks.

Reactions of the two naphthyl derivatives **6m** and **6n** furnished the corresponding sulfonates **8m** and **8n** in 30% and 28% yield. In both cases, side products **10m** and **10n** arising from an additional sulfonylation of the aromatic core were obtained in 16% and 3% yield. Furthermore, the two heterocyclic acid derivatives **6o** and **6p** underwent decarboxylative SO_2_ insertion delivering the sulfonate products **8o** and **8p** in lowered yields of 11% and 31%, together with substantial amounts of the disulfonylated products **10o** and **10p**. In all cases, a selective formation of the *E*‐configured product was observed. Reactions of the *α*‐ or *β*‐methylcinnamic acids **6q** and **6r** afforded the trisubstituted alkenesulfonates **8q** and **8r** in 28% and 37% yield, respectively. Whereas the sulfonate **8r** was again obtained exclusively as *E*‐isomer, the sulfonylated product **8q** was formed as diastereomeric mixture (*E*/*Z* = 3.5:1). Contrary to the previous work from Waldvogel^[^ ^42^ ^]^ no translocation of the double bond was observed, indicating an alternative reaction pathway. Interestingly, 2‐benzofuranoic acid **6s** could also be converted into the sulfonate **8s**, albeit in only 21% yield together with 4% of the disulfonylated product **10s**. Unfortunately, all attempts to convert simple acrylic acid derivatives, such as **6v**, or conjugated dienoic acids, such as **6w**, failed under our standard reaction conditions.

**Scheme 3:**
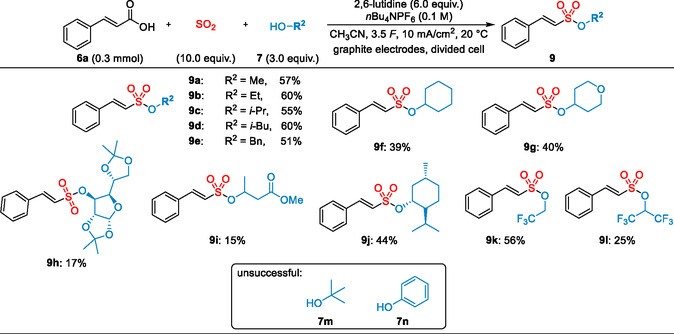
Scope of alcohols for the synthesis of alkenesulfonates **9**. Isolated yields are shown.

Next, reactions of cinnamic acid **6a** with different alcohols **7** were investigated using the optimized conditions (**Scheme** **3**). Yields of 51%–60% were obtained with different simple primary or secondary alcohols, such as MeOH, EtOH, or *i*PrOH. Even benzylic alcohol **7e**, usually more sensitive to anodic oxidation, was well tolerated, affording the expected alkenesulfonate **9e** in 51% yield. In the case of cyclohexanol **7f** and the tetrahydropyran‐derived alcohol **7g,** the desired products, **9f** and **9g,** were isolated in slightly reduced yields of 39% and 40%. Reactions with structurally more complex alcohols, such as the *α*‐D‐glucofuranose derivative **7h** and methyl 3‐hydroxybutyrate **7i**, did proceed less efficiently and yielded the sulfonates **9h** and **9i** in only 17% and 15% yield. Still, these examples show that the incorporation of structurally more complex alcohols is feasible. On the contrary, the reaction of menthol **7j** afforded the desired product **9j** in 44% yield. Reactions with tertiary alcohols, such as *t*BuOH, or with phenols did not afford the desired products. On the contrary, fluorinated alcohols, such as 2,2,2‐trifluoroethanol (TFE) or 1,1,1,3,3,3‐hexafluoroisopropan‐2‐ol (HFIP), delivered the fluoroalkyl sulfonates **9k** and **9L** in 56% and 25% yield.

### 2.3 Scale‐up and Reusability Tests

**Scheme 4:**
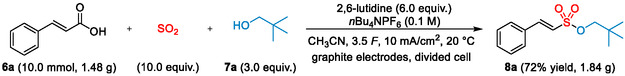
Scale‐up experiment.

In order to demonstrate the applicability of this new protocol, a 33‐fold scale‐up experiment was performed (**Scheme** **4**). To our delight, this electro‐transformation turns out to be scalable in a robust manner. Alkenesulfonate **8a** could be isolated in 72% yield (equals to 41% current efficiency (CE); max. CE (for 3.5 *F*) = 57%) in the gram‐scale reaction compared to 70% in the micromolar setup.

**Figure 2:**
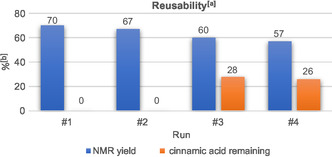
Reusability tests. ^[a]^Anolyte: **6a** (0.3 mmol, 0.1 M), SO_2_ (10.0 equiv.), **7a** (3.0 equiv.), 2,6‐lutidine (6.0 equiv.), *n*Bu_4_NPF_6_ (0.1 M), MeCN, divided cell (glass frit), graphite electrodes, 10 mA cm^−2^, 3.5 *F*, 20 °C. Catholyte: *n*Bu_4_NPF_6_ (0.1 M), AcOH (5.0 equiv.), MeCN. ^[b]1^H NMR yield with the use of CHPh_3_ as the internal standard.

Furthermore, we evaluated the electrolysis setup for repeated use. Therefore, the reaction was carried out at identical conditions four consecutive times. Only the anolyte was exchanged after each run, while the catholyte and electrodes were reused directly (see Supporting Information). These experiments showed a gradual drop in yield from 70% to 57% together with an incomplete conversion of the cinnamic acid after the third and fourth run (**Figure** **2**). This can be rationalized by minor electro‐fouling, due to the formation of a polymer layer on the anode (see Supporting Information for further details) as observed in similar transformations.^[^ ^42^, ^63^, ^64^ ^]^ As shown by the Waldvogel group, recycling of electrolyte mixtures containing an alcohol, an amine, and SO_2_ is straightforward.^[^ ^25^ ^]^ Therefore, evaporation of the anolyte components can facilitate downstream processing for larger electrolysis applications, a key step toward the translation in technical processes.^[^ ^65^ ^]^ In combination, these studies showcase the utility of the developed method for larger technical applications.

### 2.4 Mechanistic Studies

**Scheme 5:**
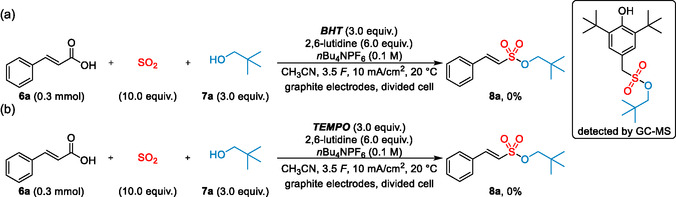
Control experiments.

Finally, further studies and control experiments were performed, in order to elucidate the reaction mechanism. As shown in Table 1, no product formation was observed in the absence of a base or when the electric current was omitted (entries 22 and 23). After addition of radical scavengers, such as 2,6‐di‐*tert*‐butyl‐4‐methylphenol (BHT) or 2,2,6,6‐tetramethylpiperidinyl‐oxyl (TEMPO), no product was detected in the crude reaction mixture (**Scheme** **5**). In both cases, only partial decomposition of cinnamic acid **6a** was observed. In the presence of BHT, an adduct, potentially arising either from trapping of an alkoxy sulfonyl radical species or Michael‐type addition of a monalkylsulfite to an in situ formed quinoid methide, could be detected by GC‐MS (see supporting information for further details).

**Figure 3:**
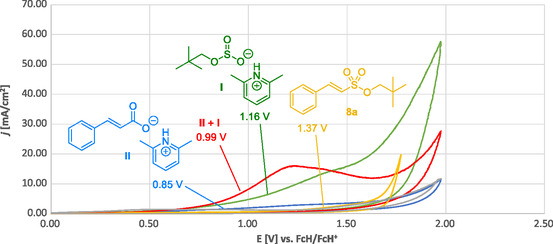
Cyclic voltammograms: Cinnamate **II** (blue), monoalkylsulfite intermediate **I** (green), alkenesulfonate **8a** (yellow), mixture of cinnamate **II** and monoalkylsulfite intermediate **I** (red), and blank measurement (0.1 M *n*Bu_4_NPF_6_ in MeCN; gray). The numbers given refer to the half‐wave oxidation potential of the respective compounds or mixtures.

Finally, cyclic voltammetry revealed that under the standard reaction conditions (excess of base), an early oxidation of the cinnamate salt should take place before oxidation of the monoalkyl sulfite or overoxidation of the alkenesulfonate (see supporting information for further details) (**Figure** **3**).

**Scheme 6:**
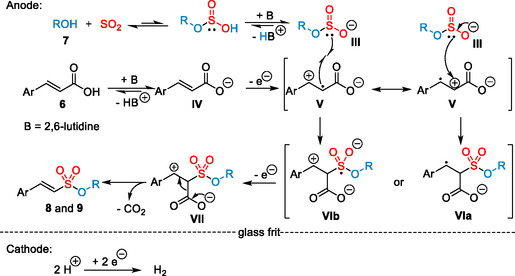
Proposed reaction pathway.

These results, together with the absence of any disulfonated products in the reaction of *β*‐methylcinnamic acid, indicate an alternative reaction pathway compared to the previously described sulfonation of styrenes.^[^ ^42^ ^]^ Based on the results from our mechanistic investigations and precedents from the literature,^[^ ^52^, ^53^, ^54^, ^55^, ^56^ ^]^ we propose pseudo‐Kolbe type reaction pathway, viz oxidation of the carbon scaffold instead of the carboxylic acid functionality (**Scheme** **6**).^[^ ^66^, ^67^ ^]^ At first, *O*‐monoalkylsulfite **III** is generated in situ from the alcohol **7** and SO_2_. The base, 2,6‐lutidine, assists in shifting the equilibrium toward the deprotonated species.^[^ ^21^, ^22^, ^23^, ^24^, ^25^, ^26^, ^68^, ^69^, ^70^, ^71^ ^]^ Furthermore, cinnamic acid will be deprotonated to the corresponding carboxylate **IV.** One‐electron oxidation of cinnamate **IV** affords radical cation **V**. Regioselective addition of *O*‐monoalkylsulfite **I** to **V** can furnish the stabilized zwitterion **VIb** containing an *S*‐centered radical. Alternatively, addition of *O*‐monoalkylsulfite **III** to **V** can also lead to the stabilized benzylic radical **VIa**. A second anodic oxidation of **VIa** or **VIb** yields the benzylic cationic **VII**, which undergoes a decarboxylation to generate the final products **8** and **9**. The stereoselective formation of the *E*‐isomer presumably occurs through the most stable staggered conformation in kinetically controlled fashion. On the cathodic side, hydrogen is generated from acetic acid in a hydrogen evolution process as simple and efficient counter reaction.

Noteworthy, pseudo‐Kolbe systems are usually limited to benzylic‐type substrates. To the best of our knowledge, herein, we describe a so far unprecedented pseudo‐Kolbe type decarboxylation of cinnamic acids. We envision that the extension of the pseudo‐Kolbe reaction to a new scaffold can be utilized for other decarboxylative functionalizations as well.^[^ ^72^, ^73^ ^]^

## 3 Conclusion

In summary, a novel electrochemical three‐component reaction for the synthesis of alkyl *β*‐styrenesulfonates is described. This metal‐free process utilizes widely available cinnamic acids, alcohols, and SO_2_ stock solution in combination with inexpensive graphite electrodes. The utility of this transformation was demonstrated in a substrate scope of 31 examples with up to 72% yield as well as in scale‐up into multigram range and anolyte reusability studies. Mechanistic investigations indicate a so far unprecedented pseudo‐Kolbe type decarboxylation of cinnamic acids. Contrary to previous methods, this new approach enables the regioselective synthesis of substituted styrene sulfonates without double bond translocation or double addition products. The direct decarboxylative conversion of biobased cinnamic acid derivatives opens an attractive access to the intriguing styrenesulfonate scaffold from renewable building blocks. Studies to electrochemically exploit the synthetic utility of the pseudo‐Kolbe decarboxylation of conjugated alkenoic acid as well as investigations to extend the electrochemical, decarboxylative SO_2_ insertion to other scaffolds are currently ongoing in our laboratories.^[^ ^74^ ^,^ ^75^ ^]^

## Supporting Information

Supporting Information File 1: Experimental details, spectral and crystal data, DOIs, and copies of NMR spectra for all compounds prepared in this study. X‐Ray Data: cif and checkcif files for compound **10o** (CCDC 2381294). The authors have cited additional references within the Supporting Information.^[76‐81]^

## Conflict of Interest

The authors declare no conflict of interest.

## Supporting information

Supplementary Material

## Data Availability

The data that support the findings of this study are openly available in [Chemotion Repository] at [https://dx.doi.org/10.14272/collection/PCC_2024‐07‐25], reference number [1014272].

## References

[1] Q. Zhong , H. Shen , X. Yun , Y. Chen , Y. Ren , H. Xu , G. Shen , W. Du , J. Meng , W. Li , J. Ma , S. Tao , Environ. Sci. Technol. 2020, 54, 6508. 32379431 10.1021/acs.est.9b07696

[2] H. Müller , Ullmann's Encyclopedia of Industrial Chemistry, Wiley‐VCH Verlag GmbH & Co., KGaA: Weinheim 2012, p. 73.

[3] P. Vogel , M. Turks , L. Bouchez , D. Marković , A. Varela‐Álvarez , J. Á. Sordo , Acc. Chem. Res. 2007, 40, 931. 17685582 10.1021/ar700096h

[4] G. Liu , C. Fan , J. Wu , Org. Biomol. Chem. 2015, 13, 1592. 25502340 10.1039/c4ob02139h

[5] N.‐W. Liu , S. Liang , G. Manolikakes , Synthesis 2016, 48, 1939.

[6] H. Woolven , C. González‐Rodríguez , I. Marco , A. L. Thompson , M. C. Willis , Org. Lett. 2011, 13, 4876. 21866926 10.1021/ol201957n

[7] J. A. Andrews , M. C. Willis , Synthesis 2022, 54, 1695.

[8] P. Bisseret , N. Blanchard , Org. Biomol. Chem. 2013, 11, 5393. 23851973 10.1039/c3ob40997j

[9] E. J. Emmett , M. C. Willis , Asian J. Org. Chem. 2015, 4, 602.

[10] S. Ye , M. Yang , J. Wu , Chem. Commun. 2020, 56, 4145. 10.1039/d0cc01775b 32242574

[11] S. Liang , K. Hofman , M. Friedrich , J. Keller , G. Manolikakes , ChemSusChem 2021, 14, 4878. 34476903 10.1002/cssc.202101635 PMC9292207

[12] G. Qiu , K. Zhou , L. Gao , J. Wu , Org. Chem. Front. 2018, 5, 691.

[13] K. Hofman , N.‐W. Liu , G. Manolikakes , Chem. Eur. J. 2018, 24, 11852. 29315874 10.1002/chem.201705470

[14] S. Ye , X. Li , W. Xie , J. Wu , Eur. J. Org. Chem. 2020, 10, 1274.

[15] S. P. Blum , K. Hofman , G. Manolikakes , S. R. Waldvogel , Chem. Commun. 2021, 57, 8236. 10.1039/d1cc03018c 34319313

[16] S. Möhle , M. Zirbes , E. Rodrigo , T. Gieshoff , A. Wiebe , S. R. Waldvogel , Angew. Chem. Int. Ed. 2018, 57, 6018. 10.1002/anie.201712732 PMC6001547 29359378

[17] M. Yan , Y. Kawamata , P. S. Baran , Chem. Rev. 2017, 117, 13230. 28991454 10.1021/acs.chemrev.7b00397 PMC5786875

[18] D. Pollok , S. R. Waldvogel , Chem. Sci. 2020, 11, 12386. 34123227 10.1039/d0sc01848a PMC8162804

[19] R. D. Little , K. D. Moeller , Chem. Rev. 2018, 118, 4483. 29739195 10.1021/acs.chemrev.8b00197

[20] T. H. Meyer , I. Choi , C. Tian , L. Ackermann , Chem 2020, 6, 2484.

[21] S. P. Blum , D. Schollmeyer , M. Turks , S. R. Waldvogel , Chem. Eur. J. 2020, 26, 8358. 32338808 10.1002/chem.202001180 PMC7383810

[22] C. Zhang , M. Yang , Y. Qiu , M. Song , H. Wang , M. Yang , W. Xie , J. Wu , S. Ye , Chem. Sci. 2022, 13, 11785. 36320920 10.1039/d2sc04027a PMC9580505

[23] J. Liu , J. Xu , H. Mei , J. Han , Green Chem. 2022, 24, 6113.

[24] S. P. Blum , T. Karakaya , D. Schollmeyer , A. Klapars , S. R. Waldvogel , Angew. Chem. Int. Ed. 2021, 60, 5056. 10.1002/anie.202016164 PMC7985875 33372349

[25] J. Schneider , S. P. Blum , S. R. Waldvogel , ChemElectroChem 2023, 10, e202300456.

[26] M. M. Hielscher , J. Schneider , A. H. J. Lohmann , S. R. Waldvogel , ChemElectroChem 2024, e202400360.

[27] S. P. Blum , L. Schäffer , D. Schollmeyer , S. R. Waldvogel , Chem. Commun. 2021, 57, 4775. 10.1039/d1cc01428e 33876137

[28] W. R. Roush , S. L. Gwaltney , J. Cheng , K. A. Scheidt , J. H. McKerrow , E. Hansell , J. Am. Chem. Soc. 1998, 120, 10994.

[29] J. J. Reddick , J. Cheng , W. R. Roush , Org. Lett. 2003, 5, 1967. 12762698 10.1021/ol034555l

[30] F. Yang , F. Xie , Y. Zhang , Y. Xia , W. Liu , F. Jiang , C. Lam , Y. Qiao , D. Xie , J. Li , L. Fu , Bioorg. Med. Chem. Lett. 2017, 27, 2166. 28372909 10.1016/j.bmcl.2017.03.060

[31] J. W. Choi , S. J. Shin , H. J. Kim , J.‐H. Park , H. J. Kim , E. H. Lee , A. N. Pae , Y. S. Bahn , K. D. Park , ACS Med. Chem. Lett. 2019, 10, 1061. 31312409 10.1021/acsmedchemlett.9b00163 PMC6627712

[32] H. Mori , E. Kudo , Y. Saito , A. Onuma , M. Morishima , Macromolecules 2010, 43, 7021.

[33] J. H. Lee , S. H. Oh , W. G. Kim , J. Mater. Sci. Mater. Med. 2004, 15, 155. 15330050 10.1023/b:jmsm.0000011817.47636.59

[34] A. Monopoli , M. Casiello , P. Cotugno , A. Milella , F. Palumbo , F. Fracassi , A. Nacci , Molecules 2021, 26, 5592. 34577063 10.3390/molecules26185592 PMC8470954

[35] F. G. Bordwell , C. S. Rondestvedt , J. Am. Chem. Soc. 1948, 70, 2429.

[36] S. Hartig , J. Prakt. Chem. 1966, 33, 215.

[37] F. G. Bordwell , C. M. Suter , J. M. Holbert , C. S. Rondestvedt , J. Am. Chem. Soc. 1946, 68, 139.

[38] B. M. Culbertson , S. Dietz , J. Chem. Soc. C 1968, 992.

[39] A. Battace , T. Zair , H. Doucet , M. Santelli , Synthesis 2006, 20, 3495.

[40] B. Schmidt , F. Wolf , H. Brunner , Eur. J. Org. Chem. 2016, 17, 2972.

[41] A. S. Barrow , C. J. Smedley , Q. Zheng , S. Li , J. Dong , J. E. Moses , Chem. Soc. Rev. 2019, 48, 4731. 31364998 10.1039/c8cs00960k

[42] A. de A. Bartolomeu , F. A. Breitschaft , D. Schollmeyer , R. A. Pilli , S. R. Waldvogel , Chem. Eur. J. 2024, 30, e202400557. 38335153 10.1002/chem.202400557

[43] X. Wang , S. Feng , J. Han , Y. Hu , S. Ye , J. Wu , J. Org. Chem. 2024, 89, 16873. 39504407 10.1021/acs.joc.4c02270

[44] X. Wang , Q. Chen , J. Zhou , Y. Hu , S. Ye , J. Wu , Chin. J. Chem. 2025, 43, 292.

[45] N. Rodríguez , L. J. Goossen , Chem. Soc. Rev. 2011, 40, 5030. 21792454 10.1039/c1cs15093f

[46] T. Patra , D. Maiti , Chem. Eur. J. 2017, 23, 7382. 27859719 10.1002/chem.201604496

[47] V. Ramadoss , Y. Zheng , X. Shao , L. Tian , Y. Wang , Chem. Eur. J. 2021, 27, 3213. 32633436 10.1002/chem.202001764

[48] V. T. Nguyen , G. C. Haug , V. D. Nguyen , N. T. H. Vuong , H. D. Arman , O. V. Larionov , Chem. Sci. 2021, 12, 6429. 34084443 10.1039/d1sc01389k PMC8115300

[49] Y. Dong , N. Xiong , Z. Rong , R. Zeng , Org. Lett. 2024, 26, 2381. 38488149 10.1021/acs.orglett.4c00410

[50] X. Xu , E. V. Van der Eycken , H. Feng , Chin. J. Chem. 2020, 38, 1780.

[51] A. J. Borah , G. Yan , Org. Biomol. Chem. 2015, 13, 8094. 26118850 10.1039/c5ob00727e

[52] J. Gao , J. Lai , G. Yuan , RSC Adv. 2015, 5, 66723.

[53] R. Singh , B. K. Allam , N. Singh , K. Kumari , S. K. Singh , K. N. Singh , Org. Lett. 2015, 17, 2656. 25954832 10.1021/acs.orglett.5b01037

[54] S. Cai , Y. Xu , D. Chen , L. Li , Q. Chen , M. Huang , W. Weng , Org. Lett. 2016, 18, 2990. 27268708 10.1021/acs.orglett.6b01353

[55] P. Qian , M. Bi , J. Su , Z. Zha , Z. Wang , J. Org. Chem. 2016, 81, 4876. 27175916 10.1021/acs.joc.6b00661

[56] Y. Zhao , Y.‐L. Lai , K.‐S. Du , D.‐Z. Lin , J.‐M. Huang , J. Org. Chem. 2017, 82, 9655. 28853571 10.1021/acs.joc.7b01741

[57] A. Vargas‐Tah , G. Gosset , Front. Bioeng. Biotechnol. 2015, 3, 116. 26347861 10.3389/fbioe.2015.00116 PMC4542537

[58] A. L. Flourat , J. Combes , C. Bailly‐Maitre‐Grand , K. Magnien , A. Haudrechy , J.‐H. Renault , F. Allais , ChemSusChem 2021, 14, 118. 33058548 10.1002/cssc.202002141

[59] M. Klein , S. R. Waldvogel , Angew. Chem. Int. Ed. 2022, 61, e202204140. 10.1002/anie.202204140 PMC9828107 35668714

[60] J. L. Röckl , D. Pollok , R. Franke , S. R. Waldvogel , Acc. Chem. Res. 2020, 53, 45. 31850730 10.1021/acs.accounts.9b00511

[61] S. R. Waldvogel , S. Lips , M. Selt , B. Riehl , C. J. Kampf , Chem. Rev. 2018, 118, 6706. 29963856 10.1021/acs.chemrev.8b00233

[62] Configuration of the double bond could be assigned via the ^3^J coupling constants of the olefinic protons. Assignment using vincinal coupling constants was verified by single crystal X‐Ray diffraction of side product **10o** (see Supporting Information for more details). Deposition Numbers CCDC 2381294, contain the supplementary crystallographic data for this paper. These data are provided free of charge by the joint. Cambridge Crystallographic Data Centre and Fachinformationszentrum Karlsruhe Access Structures service www.ccdc.cam.ac.uk/structures. Configurations of all other compounds were assigned by analogy based on ^1^H and ^13^C NMR spectroscopy.

[63] M. Katz , P. Riemenschneider , H. Wendt , Electrochim. Acta 1972, 17, 1595.

[64] V. Plzak , H. Schneider , H. Wendt , Ber. Bunsenges. Phys. Chem. 1974, 78, 1373.

[65] J. Seidler , J. Strugatchi , T. Gärtner , S. R. Waldvogel , MRS Energy Sustainability 2020, 7, 42.

[66] J. P. Coleman , R. Lines , J. H. P. Utley , B. C. L. Weedon , J. Chem. Soc. Perkin Trans. 1974, 2, 1064.

[67] M. Galicia , M. A. González‐Fuentes , D. P. Valencia , F. J. González , J. Electroanal. Chem. 2012, 672, 28.

[68] I. Anugwom , V. Eta , P. Virtanen , P. Mäki‐Arvela , M. Hedenström , M. Hummel , H. Sixta , J.‐P. Mikkola , ChemSusChem 2014, 7, 1170. 24616172 10.1002/cssc.201300773

[69] I. Anugwom , P. Mäki‐Arvela , P. Virtanen , P. Damlin , R. Sjöholm , J.‐P. Mikkola , RSC Adv. 2011, 1, 452.

[70] D. J. Heldebrant , C. R. Yonker , P. G. Jessop , L. Phan , Chem. Eur. J. 2009, 15, 7619. 19551772 10.1002/chem.200802602

[71] V.‐C. Arunasalam , I. Baxter , M. B. Hursthouse , K. M. A. Malik , D. M. P. Mingos , J. C. Plakatouras , J. Chem. Soc. Chem. Commun. 1994, 23, 2695.

[72] M. Berger , J. D. Herszman , Y. Kurimoto , G. H. M. de Kruijff , A. Schüll , S. Ruf , S. R. Waldvogel , Chem. Sci. 2020, 11, 6053. 34094098 10.1039/d0sc02417a PMC8159297

[73] G. H. M. de Kruijff , S. R. Waldvogel , ChemElectroChem 2019, 6, 4180.

[74] P‐C. Chien , F. A. Breitschaft , H. Kelm , S. R. Waldvogel , G. Manolikakes , Chemotion Repository 2024, 10.14272/collection/PCC_2024-07-25. PMC12175053 40066727

[75] P‐C. Chien , F. A. Breitschaft , H. Kelm , S. R. Waldvogel , G. Manolikakes , ChemRxiv 2024, 10.26434/chemrxiv-2024-7h1sm. PMC12175053 40066727

