# Supplementary material for: Electrochemical Three‐component Synthesis of Alkenesulfonates from Cinnamic Acids, SO2, and Alcohols
Source: ChemSusChem. 2025 Apr 16;18(12):e202500186. doi: 10.1002/cssc.202500186 (PMC12175053; doi:10.1002/cssc.202500186)
Supplement: Supplementary file 1 — Supplementary Material [file CSSC-18-e202500186-s001.pdf]

Electrochemical 3-Component Synthesis of Alkenesulfonates from Cinnamic Acids, SO_2 and Alcohols

Po-Chung Chien,[a] Florian A. Breitschaft,[b] Harald Kelm,[a] Siegfrid R. Waldvogel,[b] Georg Manolikakes*[a]

[a] Department of Chemistry
RPTU Kaiserslautern-Landau
Erwin-Schrödinger-Str. 54
D-67663 Kaiserslautern (Germany)
manolikakes@chemie.uni-kl.de

[b] Max Planck Institute for Chemical Energy Conversion
Department of Electrosynthesis
Stiftstraße 34–36
45470 Mülheim an der Ruhr (Germany)

Table of Contents

1 General Information....3

1.1. Instruments and Analytical Methods....3

1.2. Electrochemical Setup....4

2 Preparation of the SO_2 Stock Solutions....6

2.1 The Procedure for Preparation of the SO_2 Stock Solutions....6

2.2 Determination of the SO_2 Concentration of the Stock Solution....6

3 Synthesis of Starting Materials....7

3.1 Typical Procedure for the Preparation of Naphthylacrylic Acid (TP-1)....7

3.2 Typical Procedure for the Preparation of Acetoxyphenylacrylic Acid (TP-2)....7

3.3 Analytical Data for Starting Materials of Type 6....8

4 Optimization of Reaction Conditions....12

4.1 Typical Procedure for the Optimization of Reaction Conditions (TP-3) 12
4.2 Optimization of Reaction Conditions 13
5 Synthesis of Alkyl Alkenesulfonates [Scope of Cinnamic Acids and Related Substrates] .. 14
5.1 Typical Procedure for the Preparation of Alkyl Alkenesulfonates [Scope of Cinnamic Acids and Related Substrates] (TP-4) 14
5.2 Analytical Data for Compounds of Type 8 and 10 15
5.3 Scale-up Reaction 34
5.4 Reusability Tests 35
6 Synthesis of Alkyl Alkenesulfonates [Scope of Alcohols] 37
6.1 Typical Procedure for the Preparation of Alkyl Alkenesulfonates [Scope of Alcohols] (TP-5) 37
6.2 Analytical Data for Compounds of Type 9 38
7 Unsuccessful Substrates 49
8 Control Experiments 50
9 Cyclic Voltammetry Results 51
10 References 53
11 X-ray crystallographic data for selected compounds 54
12 ^{1}H, ^{13}C NMR, and ^{19}F NMR spectra for compounds 6, 8, 9, and 10 55

1 General Information

Unless otherwise mentioned, all reactions were performed under ambient conditions and chemicals in analytical grade were used as purchased without further purification. Trifluoroethanol and 1,1,1,3,3,3-hexafluoroisopropanol (HFIP) were freshly distilled from 3 Å molecular sieves and stored over 3 Å molecular sieves. All yields refer to isolated yields of compounds estimated to be > 95% pure as determined by ^{1}H-NMR.

1.1. Instruments and Analytical Methods

Chromatography: Thin-layer chromatography was performed on precoated aluminum-backed silica gel plate (Merck 60 F254, 0.2 mm thickness) which was visualized by florescence quenching. Flash-chromatography was performed on silica gel (Macherey-Nagel Kieselgel 60 0.063–0.2 mm).
Automated flash column chromatography: Preparative flash column chromatography was performed on a prepacked puriFlash™ silica column (80 g, 25 µm, PF-25SiHC-F0080, *Interchim*, Montlucon Cedex, France) using a puriFlash™-System (puriFlash™ XS520Plus, *Interchim*, Montlucon Cedex, France) with an integrated UV detector and fraction collector. Detection of the product was achieved using the absorption at 254 nm.
Solvents: Anhydrous acetonitrile (CH_3CN), purchased from Thermo Fisher Scientific was used without any further purification. The solvents for column chromatography were technical standard.

Materials: Starting materials which were not commercially available were synthesized according to the previously reported methods.[76,77]

NMR spectroscopy: Spectra of proton (^{1}H), carbon (^{13}C) and fluorine (^{19}F) nuclear magnetic resonance were recorded at 400 MHz, 101 MHz and 376 MHz respectively. Chemical shifts are reported in δ ppm referenced to an internal standard, such as TMS for ^{1}H-NMR (δ = 0.0 ppm), $CDCl_3$ for ^{13}C-NMR (δ = 77.16 ppm), DMSO-d_6 for ^{1}H-NMR (δ = 2.50 ppm), DMSO-d_6 for ^{13}C-NMR (δ = 39.52 ppm). The coupling constants (J) are reported in Hz and the following abbreviations were used to explain the multiplicities: s (singlet), d (doublet), t (triplet), q (quartet), m (multiplet), dd (doublet of doublet), td (triplet of doublet), tt (triplet of triplet), qq (quartet of quartet), and septetd (septet of doublet).

Melting points: Melting points were measured on a hot stage meting point apparatus and are uncorrected.

Mass spectrometry: Mass spectra (MS) were measured using electrospray ionization (ESI) or

atmospheric-pressure chemical ionization (APCI) techniques with a quadrupole mass analyzer. High resolution mass spectra (HRMS) were measured using electron ionization mass spectroscopy with a time-of-flight mass analyzer (EI-MS-TOF), electrospray ionization mass spectroscopy (ESI-MS) with quadrupole-orbitrap mass analyzer, and gas chromatography coupled with electron ionization mass spectroscopy (GC-EI-MS) and quadrupole-orbitrap mass analyzer.

Gas Chromatography coupled with Mass Spectrometry (GC/MS): Analysis of crude reaction mixtures was performed using Thermo Scientific ISQ 7000 equipped with an electron ionization (EI) source and a quadrupole mass analyzer.

Infrared spectroscopy: Infrared spectra (IR) of neat substances were recorded on an FT-IR (Fourier transform infrared spectroscopy) spectrometer equipped with a diamond universal ATR sampling technique (attenuated total reflectance). The absorption bands are reported in wave numbers (cm^{-1}) and only selected peaks are shown.

X-ray Crystallography: The measurement of the crystal structure was carried out on a Rigaku/Oxford diffraction Xcalibur/Gemini dual wavelength diffractometer with a Cu-Kα (λ =1.54178 Å) radiation X-ray source.

Cyclic Voltammetry (CV) Measurements: Cyclic voltammetry was performed using Interface 1010T (Gamry Instruments, Pennsylvania, United States of America). WE: Pt electrode; CE: Pt electrode; RE: Ag/AgCl; Scan rate v = 100 mV/s. All data is displayed against the half-wave potential of ferrocene/ferrocenium redox couple (FcH/FcH^+; 0.02 V vs. Ag/AgCl) as the internal reference. Oxidation potentials are marked and displayed as the half-wave potential of the respective peak.

1.2. Electrochemical Setup

Screening and small-scale reactions: Electrochemical reactions were carried out using an IKA ElectraSyn 2.0. Parallel reactions were performed with the IKA Carousell. The divided cell used for screening or small-scale reactions was an IKA Pro-Divide equipped with a glass frit (pore size 10-16 microns). All used electrodes were purchased from IKA.

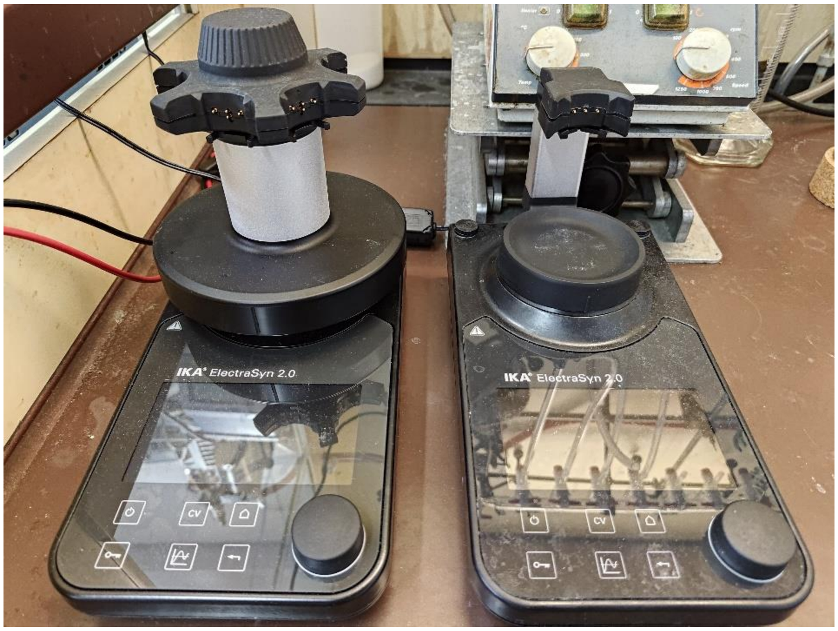

Figure S1: IKA ElectraSyn 2.0 and IKA Carousell.

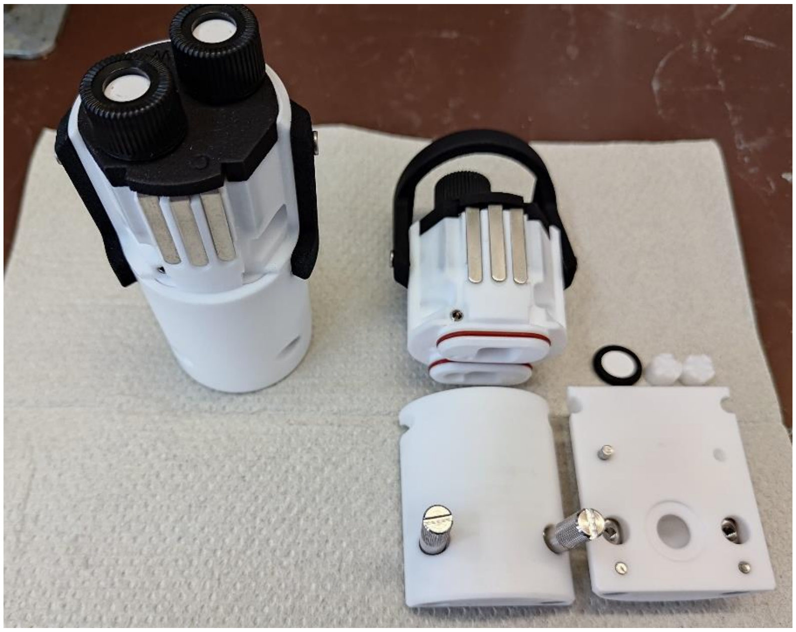

Figure S2: IKA Pro-Divide divided cell.

Scale-up cell: The scale-up experiment was performed in a divided glass cell with a volume of 100 mL per half-cell equipped with a PTFE stopper and sleeve, electrodes, and electrode holders, a glass frit (P4, d = 24 mm) and cross-shaped stirring bars. The electrolysis was conducted using a TDK-Lambda Z+ series (*TDK-Lambda UK Limited*, Devon, United Kingdom) as power source. The electrode dimensions were 60 mm × 20 mm × 2 mm, with 45 mm submerged into the reaction mixture, resulting in an active electrode area of 9.0 cm^2. The graphite employed was Sigrafine^{TM} V2100 by SGL Carbon/Bad Godesberg, Germany. The stoppers, sleeves, electrodes and their

holders are available at Sigma-Aldrich within the SynLectro™ series.

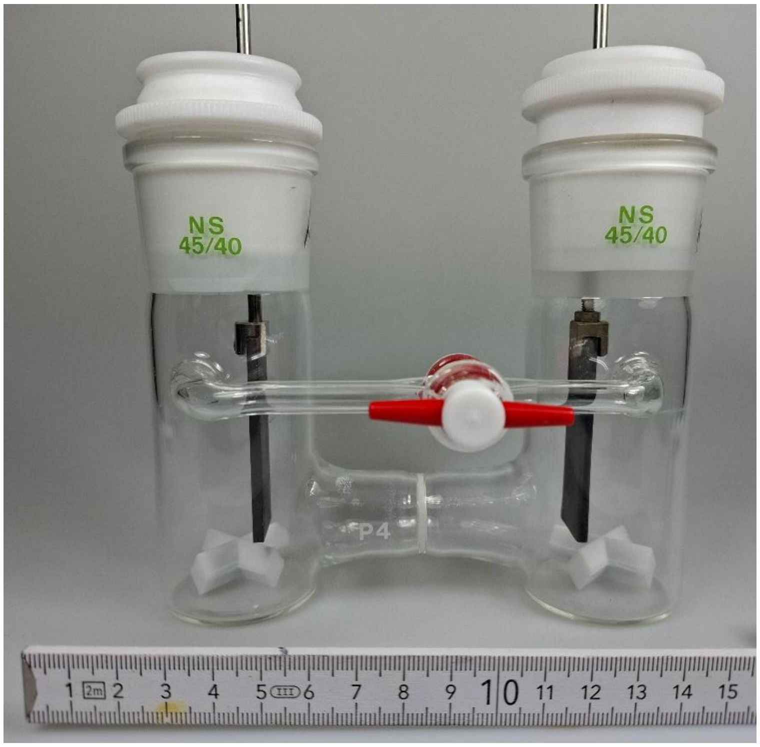

Figure S3: Electrochemical glass cell with 100 mL per half-cell used for the scale-up experiment equipped with a P4 frit, graphite electrodes, Teflon™ plug, and electrode holders. Scale is given in cm.

2 Preparation of the SO_2 Stock Solutions

2.1 The Procedure for Preparation of the SO_2 Stock Solutions

300 mL of anhydrous acetonitrile and molecular sieves (3 Å, 50.0 g) were transferred into a dry two-neck round bottom flask. Sulfur dioxide was bubbled into the solution at a slow flow rate for 15 minutes at 0 °C. Excess gaseous sulfur dioxide leaving the apparatus was purged with aq. NaOH solution.

2.2 Determination of the SO_2 Concentration of the Stock Solution

The exact concentration of the prepared SO_2 solution was determined according to iodometric titration.[78] To a solution of I_2 (1.27 g, 5.00 mmol) and KI (2.20 g, 13.3 mmol) in H_2O (100 mL) was slowly added the freshly prepared SO_2 stock solution (1.0 mL). The resulting solution was then titrated with a freshly prepared aq. $Na_2S_2O_3$ solution (0.2 M) as titrant to determine the amount of excess I_2 (end point marked a color change from brown to colorless; optionally starch can be

added for better visualization). Titrations of individual batches of stock solutions were repeated three times for a more accurate determination of the concentration.

3 Synthesis of Starting Materials

3.1 Typical Procedure for the Preparation of Naphthylacrylic Acid (TP-1)

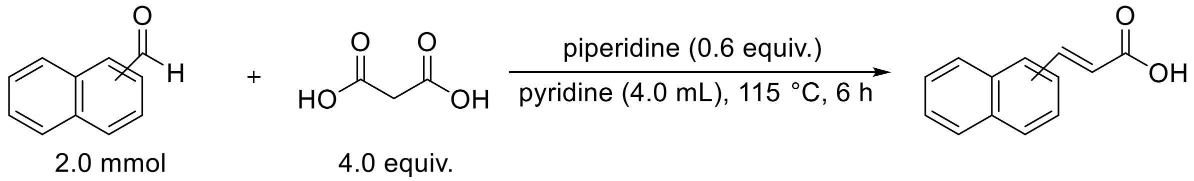

The naphthylacrylic acid was synthesized according to the reported literature.[76]

A 25.0 mL single neck round-bottomed flask equipped with a magnetic stir bar was charged with the corresponding naphthaldehyde (312.4 mg, 2.0 mmol), malonic acid (832.5 mg, 4.0 equiv.), piperidine (0.13 mL, 0.6 equiv.), and pyridine (4.0 mL) and heated to 115 °C for 6 h. Afterwards, the reaction mixture was cooled to room temperature, poured into an Erlenmeyer flask, and acidified with 2.0 M HCl to a final pH = 1. The resulting precipitate was filtered and washed with distilled water (30.0 mL) to afford the naphthylacrylic acid.

3.2 Typical Procedure for the Preparation of Acetoxyphenylacrylic Acid (TP-2)

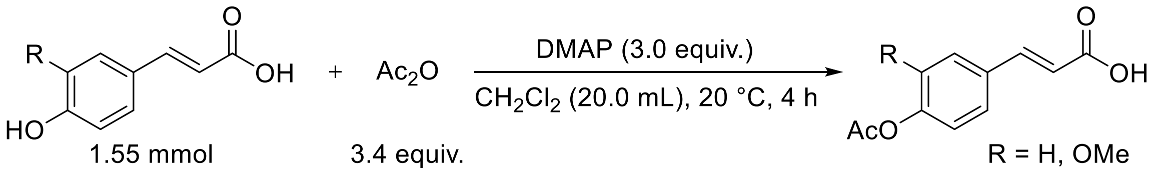

The acetoxyphenylacrylic acid was synthesized according to the reported literature.[77]

A 50.0 mL single neck round-bottomed flask equipped with a magnetic stir bar was charged with hydroxycinnamic acid (1.55 mmol), CH_2Cl_2 (20.0 ml), acetic anhydride (0.5 mL, 3.4 equiv.), and DMAP (568.1 mg, 3.0 equiv.). The resulting solution was stirred at 20 °C for 4 h. After completion of the reaction, the reaction mixture was acidified (pH = 1) with 2.0 M HCl and extracted with CH_2Cl_2 (3 x 20.0 mL). The organic layer was dried over anhydrous Na_2SO_4 and concentrated in *vacuo*. The crude residue was subjected to flash column chromatography on silica gel to obtain acetoxyphenylacrylic acid.

3.3 Analytical Data for Starting Materials of Type 6

(*E*)-3-(Naphthalen-1-yl)acrylic acid (6m)

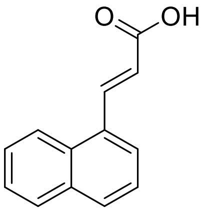

Following the **TP-1**, 1-naphthaldehyde (312.4 mg, 2.0 mmol), malonic acid (832.5 mg, 4.0 equiv.), and piperidine (0.13 mL, 0.6 equiv.) were used as substrates. After 6 h, the reaction mixture was acidified. The resulting precipitate was filtered and washed with distilled water to obtain **6m** as a colorless solid (337.5 mg, 85%). Analytical data for **6m** match those previously described in the literature.[79]

mp: 178.9–179.8 °C. R_f = 0.38 (*n*-hexane/EtOAc = 1:1).

^{1}H NMR (400 MHz, DMSO-d_6): δ 12.62 (s, 1H), 8.41 (d, *J* = 15.6 Hz, 1H), 8.18 (d, *J* = 8.4 Hz, 1H), 7.99 (d, *J* = 8.4 Hz, 1H), 7.97 (d, *J* = 8.4 Hz, 1H), 7.93 (d, *J* = 7.6 Hz, 1H), 7.64–7.50 (m, 3H), 6.61 (d, *J* = 15.6 Hz, 1H).

^{13}C{^{1}H}-NMR (101 MHz, DMSO-d_6): δ 167.5, 140.2, 133.3, 131.0, 130.8, 130.4, 128.7, 127.1, 126.3, 125.7, 125.2, 123.0, 122.0.

IR (ATR) $\tilde{\nu}$ (cm^{-1}): 3046, 1677, 1614, 975, 763.

MS (APCI): *m/z* calcd. for $C_{13}H_9O_2$ ([M-H]$^-$) 197.1, found 197.3.

HRMS (EI$^+$): *m/z* calcd. for $C_{13}H_{10}O_2$ ([M]$^+$) 198.0681, found 198.0686.

Additional information on the chemical synthesis is available *via* Chemotion repository:
https://doi.org/10.14272/reaction/SA-FUHFF-UHFFFADPSC-WPXMLUUYWN-UHFFFADPSC-NUHFF-NSEGZ-NUHFF-ZZZ

Additional information on the analysis of the target compound is available *via* Chemotion repository:
https://doi.org/10.14272/WPXMLUUYWNHQOR-CMDGGOBGSA-N.1

(*E*)-3-(Naphthalen-2-yl)acrylic acid (6n)

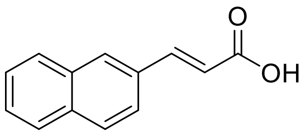

Following the **TP-1**, 2-naphthaldehyde (312.4 mg, 2.0 mmol), malonic acid (832.5 mg, 4.0 equiv.), and piperidine (0.13 mL, 0.6 equiv.) were used as substrates. After 6 h, the reaction mixture was acidified. The resulting precipitate was filtered and washed with distilled water to obtain **6n** as a colorless solid (315.3 mg, 80%). Analytical data for **6n** match those previously described in the literature.[80]

mp: 176.7–177.3 °C. R_f = 0.30 (*n*-hexane/EtOAc = 1:1).

^{1}H NMR (400 MHz, DMSO-d_6): δ 12.47 (s, 1H), 8.18 (s, 1H), 7.99–7.90 (m, 3H), 7.87 (dd, *J* = 8.6, 1.4 Hz, 1H), 7.76 (d, *J* = 16.0 Hz, 1H), 7.61–7.50 (m, 2H), 6.67 (d, *J* = 16.0 Hz, 1H).

^{13}C{^{1}H}-NMR (101 MHz, DMSO-d_6): δ 167.7, 143.9, 133.7, 132.9, 131.9, 129.7, 128.53, 128.47, 127.7, 127.3, 126.8, 124.0, 119.6.

IR (ATR) $\tilde{\nu}$ (cm^{-1}): 3045, 1680, 1624, 983, 739.

MS (APCI): *m/z* calcd. for $C_{13}H_{11}O_2$ ([M+H]$^+$) 199.1, found 199.3.

HRMS (EI$^+$): *m/z* calcd. for $C_{13}H_{10}O_2$ ([M]$^+$) 198.0681, found 198.0687.

Additional information on the chemical synthesis is available *via* Chemotion repository:
https://doi.org/10.14272/reaction/SA-FUHFF-UHFFFADPSC-KWGPBDBAAX-UHFFFADPSC-NUHFF-NCGTV-NUHFF-ZZZ

Additional information on the analysis of the target compound is available *via* Chemotion repository:
https://doi.org/10.14272/KWGPBDBAAXYWOJ-SOFGYWHQSA-N.1

(*E*)-3-(4-Acetoxyphenyl)acrylic acid (6j)

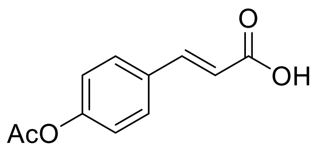

Following the **TP-2**, *p*-hydroxycinnamic acid (254.4 mg, 1.55 mmol), acetic anhydride (0.5 mL, 3.4 equiv.), and DMAP (568.1 mg, 3.0 equiv.) were used as substrates. After 4 h, the reaction mixture was acidified. The crude residue was purified by flash chromatography over silica gel (*n*-hexane/EtOAc = 2:1) to obtain **6j** as a colorless solid (287.0 mg, 90% yield). Analytical data for **6j** match those previously described in the literature.[81]

mp: 207.5–208.2 °C. R_f = 0.30 (*n*-hexane/EtOAc = 1:1).

^{1}H NMR (400 MHz, DMSO-d_6): δ 12.44 (s, 1H), 7.89–7.68 (m, 2H), 7.61 (d, *J* = 15.6 Hz, 1H), 7.33–7.06 (m, 2H), 6.52 (d, *J* = 15.6 Hz, 1H), 2.27 (s, 3H).

^{13}C{^{1}H}-NMR (101 MHz, DMSO-d_6): δ 169.1, 167.6, 151.9, 143.0, 132.0, 129.5, 122.4, 119.4, 20.9.

IR (ATR) $\tilde{\nu}$ (cm^{-1}): 2816, 1741, 1672, 1192, 992, 793.

MS (APCI): *m/z* calcd. for $C_{11}H_{11}O_4$ ($[M+H]^+$) 207.1, found 207.3.

HRMS (EI^+): *m/z* calcd. for $C_{11}H_{10}O_4$ ($[M]^+$) 206.0579, found 206.0574.

Additional information on the chemical synthesis is available *via* Chemotion repository: https://doi.org/10.14272/reaction/SA-FUHFF-UHFFFADPSC-BYHBHNKBIS-UHFFFADPSC-NUHFF-NNUHD-NUHFF-ZZZ

Additional information on the analysis of the target compound is available *via* Chemotion repository:
https://doi.org/10.14272/BYHBHNKBISXCEP-QPJJXVBHSA-N.1

(*E*)-3-(4-Acetoxy-3-methoxyphenyl)acrylic acid (6k)

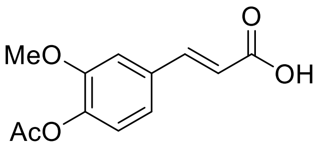

Following the **TP-2**, (*E*)-3-(4-hydroxy-3-methoxyphenyl)acrylic acid (301.0 mg, 1.55 mmol), acetic anhydride (0.5 mL, 3.4 equiv.), and DMAP (568.1 mg, 3.0 equiv.) were used as substrates. After 4 h, the reaction mixture was acidified. The crude residue was purified by flash chromatography over silica gel (*n*-hexane/EtOAc = 2:1) to obtain **6k** as a colorless solid (306.1 mg, 84% yield). Analytical data for **6k** match those previously described in the literature.[81]

mp: 199.5–200.3 °C. R_f = 0.20 (*n*-hexane/EtOAc = 1:1).

^{1}H NMR (400 MHz, DMSO-d_6): δ 12.43 (s, 1H), 7.58 (d, *J* = 16.0 Hz, 1H), 7.48 (d, *J* = 1.6 Hz, 1H), 7.26 (dd, *J* = 8.2, 1.6 Hz, 1H), 7.12 (d, *J* = 8.2 Hz, 1H), 6.59 (d, *J* = 16.0 Hz, 1H), 3.82 (s, 3H), 2.26 (s, 3H).

^{13}C{^{1}H}-NMR (101 MHz, DMSO-d_6): δ 168.5, 167.7, 151.2, 143.4, 140.8, 133.3, 123.2, 121.4, 119.6, 111.9, 56.0, 20.4.

IR (ATR) $\tilde{\nu}$ (cm^{-1}): 3012, 1760, 1630, 1221, 1198, 983.

MS (APCI): *m/z* calcd. for $C_{12}H_{13}O_5$ ([M+H]$^+$) 237.1, found 237.1.

HRMS (ESI$^-$): *m/z* calcd. for $C_{12}H_{11}O_5$ ([M-H]$^-$) 235.061200, found 235.061300.

Additional information on the chemical synthesis is available *via* Chemotion repository:
https://doi.org/10.14272/reaction/SA-FUHFF-UHFFFADPSC-IHKNVZISLL-UHFFFADPSC-NUHFF-NDUUA-NUHFF-ZZZ

Additional information on the analysis of the target compound is available *via* Chemotion repository:
https://doi.org/10.14272/IHKNVZISLLDMOR-GQCTYLIASA-N.1

4 Optimization of Reaction Conditions

4.1 Typical Procedure for the Optimization of Reaction Conditions (TP-3)

The reactions were carried out using IKA Pro-Divide cells with a glass frit membrane.

Anolyte: An oven-dried 10.0 mL pear-shaped flask was charged with cinnamic acid (44.5 mg, 0.3 mmol), neopentyl alcohol, ammonium salt, and anhydrous acetonitrile. The mixture was cooled to 0 °C in an ice bath, followed by the addition of SO_2 stock solution and base so that a total volume of 3.0 mL was achieved.
Catholyte: An oven-dried 10.0 mL pear-shaped flask was charged with ammonium salt, acetic acid (5.0 equiv.), and anhydrous acetonitrile so that a total volume of 3.0 mL was achieved as well.

The reaction mixtures were transferred with syringes to their respective compartment simultaneously. The amperage and amount of applied charge was set to the desired values and the electrolysis was conducted at room temperature under constant stirring (400 rpm).

After completion of the electrolysis, the anolyte was transferred to a round bottom flask, and the anode compartment was rinsed with additional EtOAc (2 x 3.0 mL). ^{1}H NMR yields were calculated by addition of triphenylmethane (73.2 mg, 1.0 equiv.) as the internal standard to this mixture. After 5 min of stirring, 1.0 mL of the mixture was taken, and the solvent was removed under reduced pressure. The crude residue was dissolved in $CDCl_3$ for ^{1}H NMR experiments.

4.2 Optimization of Reaction Conditions

Optimization of the conditions was carried out by using linear screening with cinnamic acid and neopentyl alcohol as substrates according to the procedure described in **TP-3**. Neopentyl alcohol was chosen due to the enhanced stability of sulfonate neopentyl esters.

Table S1: Screening of electrodes, applied charge, current density, base, and electrolyte.

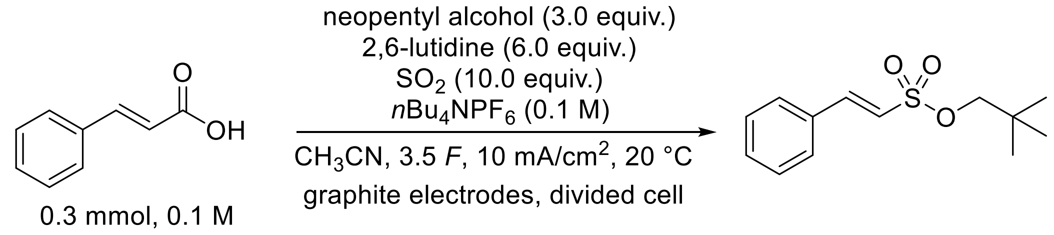

entry	deviation from the standard conditions	yield (%)[a]	cinnamic acid (%)[a]
**1**	**none**	**71 (70)[b]**	**0**
2	BDD electrodes	25	25
3	glassy carbon electrodes	46	traces
4	Pt foil electrodes	40	15
5	3.0 *F*	63	0
6	3.8 *F*	54	0
7	15 mA/cm^2	65	0
8	5 mA/cm^2	55	0
9	2,4,6-collidine	55	traces
10	pyridine	54	traces
11	DBU	62	0
12	DIPEA	traces	53
13	*n*Bu_4NBF_4	50	0

[a]^{1}H-NMR yield with the use of $CHPh_3$ as the internal standard. [b]Isolated yield.

Table S2: Screening of the stoichiometry of alcohol, base, SO_2, and other parameters.

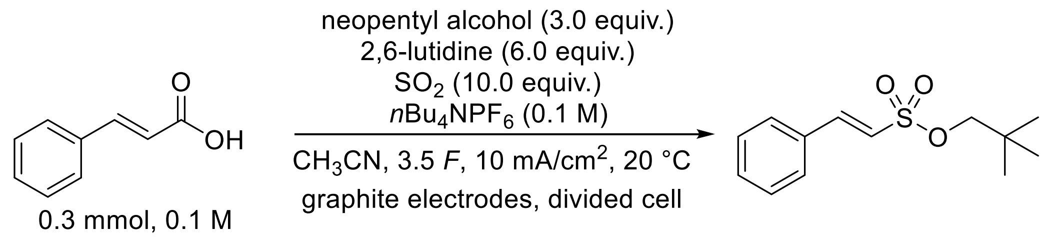

entry	deviation from the standard conditions	yield (%)[a]	cinnamic acid (%)[a]
**1**	**none**	**71 (70)[b]**	**0**
2	nBu$_4$NPF$_6$ (0.05 M)	58	0
3	nBu$_4$NPF$_6$ (0.2 M)	43	0
4	neopentyl alcohol (2.0 equiv.)	50	0
5	neopentyl alcohol (4.0 equiv.)	47	0
6	2,6-lutidine (4.0 equiv.)	43	0
7	2,6-lutidine (8.0 equiv.)	49	0
8	SO_2 (7.5 equiv.)	63	0
9	SO_2 (12.5 equiv.)	68	0
10	CH_3CN:HFIP = 1:1	56	0

[a]^{1}H-NMR yield with the use of $CHPh_3$ as the internal standard. [b]Isolated yield.

5 Synthesis of Alkyl Alkenesulfonates [Scope of Cinnamic Acids and Related Substrates]

5.1 Typical Procedure for the Preparation of Alkyl Alkenesulfonates [Scope of Cinnamic Acids and Related Substrates] (TP-4)

The reactions were carried out using IKA Pro-Divide cells with a glass frit membrane.

Anolyte: An oven-dried 10.0 mL pear-shaped flask was charged with neopentyl alcohol (79.3 mg, 3.0 equiv.), nBu$_4$NPF$_6$ (116.3 mg, 1.0 equiv.), and anhydrous acetonitrile (2.1 mL). The mixture was cooled to 0 °C in an ice bath, followed by the addition of SO_2 stock solution (0.6 mL, 5.0 M in acetonitrile, 10.0 equiv.) and 2,6-lutidine (0.21 mL, 6.0 equiv.) so that a total volume of 3.0 mL was achieved.

Catholyte: An oven-dried 10.0 mL pear-shaped flask was charged with nBu$_4$NPF$_6$ (116.3 mg, 1.0 equiv.), acetic acid (0.09 mL, 5.0 equiv.), and anhydrous acetonitrile (2.9 mL) so that a total volume

of 3.0 mL was achieved as well.

The reaction mixtures were transferred with syringes to their respective compartment simultaneously and the anolyte was additionally loaded with the respective acid (0.3 mmol). The amperage was set accordingly so that a current density of 10 mA×cm^{-2} was reached (8.8 mA with the setup described herein) and the amount of applied charge was set to 3.5 *F*. The electrolysis was conducted at room temperature under constant stirring (400 rpm) for ca. 3 h 11 min.

After completion of the electrolysis, the anolyte was transferred to a separatory funnel and the anode compartment was rinsed with additional EtOAc (2 x 3.0 mL). Distilled water (10.0 mL) was added and the obtained mixture extracted with EtOAc (2 x 10.0 mL). The organic layer was dried over anhydrous Na_2SO_4 and concentrated in *vacuo*. The crude residue was subjected to flash column chromatography on silica gel to obtain the product.

5.2 Analytical Data for Compounds of Type 8 and 10

Neopentyl (*E*)-2-phenylethene-1-sulfonate (8a)

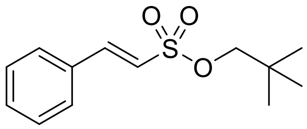

Following the **TP-4**, cinnamic acid (44.5 mg, 0.3 mmol), neopentyl alcohol (79.3 mg, 3.0 equiv.), and SO_2 stock solution (10.0 equiv.) were used as substrates. The residue was purified by flash chromatography over silica gel (*n*-hexane/EtOAc = 97:3) to obtain **8a** as a colorless solid (53.5 mg, 70% yield). Analytical data for **8a** match those previously described in the literature.[42]
mp: 71.3–72.1 °C. R_f = 0.55 (*n*-hexane/EtOAc = 4:1).
^{1}H NMR (400 MHz, $CDCl_3$): δ 7.60 (d, *J* = 15.6 Hz, 1H), 7.55–7.50 (m, 2H), 7.50–7.41 (m, 3H), 6.75 (d, *J* = 15.6 Hz, 1H), 3.80 (s, 2H), 0.98 (s, 9H).

^{13}C{^{1}H}-NMR (101 MHz, $CDCl_3$): δ 144.8, 132.1, 131.6, 129.3, 128.6, 121.1, 79.7, 31.8, 26.2.

IR (ATR) $\tilde{\nu}$ (cm^{-1}): 3067, 2966, 1625, 1348, 1159, 953, 746.

MS (APCI): *m/z* calcd. for $C_{13}H_{19}O_3S$ ($[M+H]^+$) 255.1, found 255.4.

HRMS (EI$^+$): *m/z* calcd. for $C_{13}H_{18}O_3S$ ($[M]^+$) 254.0977, found 254.0989.

Additional information on the chemical synthesis is available *via* Chemotion repository:

https://doi.org/10.14272/reaction/SA-FUHFF-UHFFFADPSC-JSPSUKCDZR-UHFFFADPSC-NUHFF-NAIXQ-NUHFF-ZZZ

Additional information on the analysis of the target compound is available *via* Chemotion repository:

https://doi.org/10.14272/JSPSUKCDZRFMIT-MDZDMXLPSA-N.1

Neopentyl (*E*)-2-(*p*-tolyl)ethene-1-sulfonate (8b)

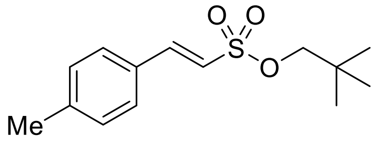

Following the **TP-4**, (*E*)-3-(*p*-tolyl)acrylic acid (48.7 mg, 0.3 mmol), neopentyl alcohol (79.3 mg, 3.0 equiv.), and SO_2 stock solution (10.0 equiv.) were used as substrates. The residue was purified by flash chromatography over silica gel (*n*-hexane/EtOAc = 98:2) to obtain **8b** as a colorless solid (41.8 mg, 52% yield). Analytical data for **8b** match those previously described in the literature.[42]

mp: 82.3–82.5 °C. R_f = 0.32 (*n*-hexane/EtOAc = 9:1).

^{1}H NMR (400 MHz, $CDCl_3$): δ 7.57 (d, J = 15.6 Hz, 1H), 7.44–7.39 (m, 2H), 7.26–7.21 (m, 2H), 6.68 (d, J = 15.6 Hz, 1H), 3.79 (s, 2H), 2.40 (s, 3H), 0.98 (s, 9H).

^{13}C{^{1}H}-NMR (101 MHz, $CDCl_3$): δ 144.9, 142.3, 130.1, 129.4, 128.6, 119.9, 79.6, 31.9, 26.2, 21.7.

IR (ATR) $\tilde{\nu}$ (cm^{-1}): 3059, 2959, 1620, 1338, 1161, 955, 795.

MS (APCI): *m/z* calcd. for $C_{14}H_{21}O_3S$ ([M+H]$^+$) 269.1, found 269.4.

HRMS (EI$^+$): *m/z* calcd. for $C_{14}H_{20}O_3S$ ([M]$^+$) 268.1133, found 268.1142.

Additional information on the chemical synthesis is available *via* Chemotion repository:
https://doi.org/10.14272/reaction/SA-FUHFF-UHFFFADPSC-NRHDKVMGUT-UHFFFADPSC-NUHFF-NAIXQ-NUHFF-ZZZ

Additional information on the analysis of the target compound is available *via* Chemotion repository:
https://doi.org/10.14272/NRHDKVMGUTWHAC-MDZDMXLPSA-N.1

Neopentyl (*E*)-2-(4-methoxyphenyl)ethene-1-sulfonate (8c)

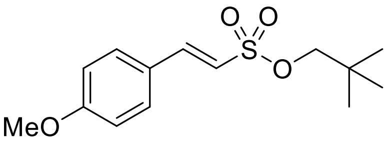

Following the **TP-4**, (*E*)-3-(4-methoxyphenyl)acrylic acid (53.5 mg, 0.3 mmol), neopentyl alcohol (79.3 mg, 3.0 equiv.), and SO_2 stock solution (10.0 equiv.) were used as substrates. The residue was purified by flash chromatography over silica gel (*n*-hexane/EtOAc = 98:2) to obtain **8c** as a colorless solid (46.3 mg, 54% yield). Analytical data for **8c** match those previously described in the literature.[42]

mp: 125.0–126.0 °C. R_f = 0.20 (*n*-hexane/EtOAc = 9:1).

^{1}H NMR (400 MHz, $CDCl_3$): δ 7.54 (d, *J* = 15.6 Hz, 1H), 7.49–7.45 (m, 2H), 6.97–6.92 (m, 2H), 6.58 (d, *J* = 15.6 Hz, 1H), 3.86 (s, 3H), 3.78 (s, 2H), 0.98 (s, 9H).

^{13}C{^{1}H}-NMR (101 MHz, $CDCl_3$): δ 162.4, 144.6, 130.4, 124.7, 118.2, 114.7, 79.5, 55.6, 31.8, 26.2.

IR (ATR) $\tilde{\nu}$ (cm^{-1}): 3065, 2956, 1604, 1349, 1158, 962, 792.

MS (APCI): *m/z* calcd. for $C_{14}H_{21}O_4S$ ($[M+H]^+$) 285.1, found 285.4.

HRMS (EI^+): *m/z* calcd. for $C_{14}H_{20}O_4S$ ($[M]^+$) 284.1082, found 284.1100.

Additional information on the chemical synthesis is available *via* Chemotion repository:
https://doi.org/10.14272/reaction/SA-FUHFF-UHFFFADPSC-CXLLNBAHTN-UHFFFADPSC-NUHFF-NAIXQ-NUHFF-ZZZ

Additional information on the analysis of the target compound is available *via* Chemotion repository:
https://doi.org/10.14272/CXLLNBAHTNGKGY-MDZDMXLPSA-N.1

Neopentyl (*E*)-2-(4-nitrophenyl)ethene-1-sulfonate (8d)

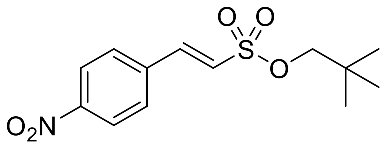

Following the **TP-4**, (*E*)-3-(4-nitrophenyl)acrylic acid (58.0 mg, 0.3 mmol), neopentyl alcohol (79.3 mg, 3.0 equiv.), and SO_2 stock solution (10.0 equiv.) were used as substrates. The residue was purified by flash chromatography over silica gel (*n*-hexane/EtOAc = 95:5) to obtain **8d** as a colorless solid (53.7 mg, 60% yield).

mp: 88.8–89.7 °C. R_f = 0.15 (*n*-hexane/EtOAc = 9:1).

^{1}H NMR (400 MHz, $CDCl_3$): δ 8.33–8.28 (m, 2H), 7.73–7.69 (m, 2H), 7.66 (d, *J* = 15.6 Hz, 1H), 6.92 (d, *J* = 15.6 Hz, 1H), 3.86 (s, 2H), 1.00 (s, 9H).

^{13}C{^{1}H}-NMR (101 MHz, $CDCl_3$): δ 149.3, 141.6, 138.1, 129.3, 125.7, 124.5, 80.2, 31.9, 26.2.

IR (ATR) $\tilde{\nu}$ (cm^{-1}): 3126, 2960, 1625, 1529, 1344, 1165, 959, 803.

MS (APCI): *m/z* calcd. for $C_{13}H_{18}NO_5S$ ($[M+H]^+$) 300.1, found 300.5.

HRMS (EI^+): *m/z* calcd. for $C_{13}H_{17}NO_5S$ ($[M]^+$) 299.0827, found 299.0838.

Additional information on the chemical synthesis is available *via* Chemotion repository:

https://doi.org/10.14272/reaction/SA-FUHFF-UHFFFADPSC-MFGOXJJGCZ-UHFFFADPSC-NUHFF-NSEGZ-NUHFF-ZZZ

Additional information on the analysis of the target compound is available *via* Chemotion repository:

https://doi.org/10.14272/MFGOXJJGCZRRRX-CMDGGOBGSA-N.1

Neopentyl (*E*)-2-(4-fluorophenyl)ethene-1-sulfonate (8e)

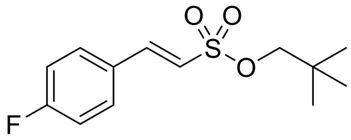

Following the **TP-4**, (*E*)-3-(4-fluorophenyl)acrylic acid (49.8 mg, 0.3 mmol), neopentyl alcohol (79.3 mg, 3.0 equiv.), and SO_2 stock solution (10.0 equiv.) were used as substrates. The residue was purified by flash chromatography over silica gel (*n*-hexane/EtOAc = 98:2) to obtain **8e** as a colorless liquid (38.5 mg, 47% yield). Analytical data for **8e** match those previously described in

the literature.[42]

R$_f$ = 0.25 (*n*-hexane/EtOAc = 9:1).

^{1}H NMR (400 MHz, $CDCl_3$): δ 7.57 (d, *J* = 15.6 Hz, 1H), 7.55–7.50 (m, 2H), 7.10–7.02 (m, 2H), 6.68 (d, *J* = 15.6 Hz, 1H), 3.80 (s, 2H), 0.98 (s, 9H).

^{13}C{^{1}H}-NMR (101 MHz, $CDCl_3$): δ 164.6 (d, $^1J_{C\text{-}F}$ = 253.4 Hz, 1C), 143.4, 130.7 (d, $^3J_{C\text{-}F}$ = 8.8 Hz, 2C), 128.4 (d, $^4J_{C\text{-}F}$ = 3.4 Hz, 1C), 121.0 (d, $^5J_{C\text{-}F}$ = 2.0 Hz, 1C), 116.6 (d, $^2J_{C\text{-}F}$ = 22.0 Hz, 2C), 79.7, 31.9, 26.2.

^{19}F{^{1}H}-NMR (376 MHz, $CDCl_3$): δ -107.4.

IR (ATR) $\tilde{\nu}$ (cm^{-1}): 3003, 2963, 1710, 1358, 1221, 1166, 966, 852.

MS (APCI): *m/z* calcd. for $C_{13}H_{18}O_3FS$ ([M+H]$^+$) 273.1, found 273.2.

HRMS (EI$^+$): *m/z* calcd. for $C_{13}H_{17}O_3FS$ ([M]$^+$) 272.0882, found 272.0886.

Additional information on the chemical synthesis is available *via* Chemotion repository:
https://doi.org/10.14272/reaction/SA-FUHFF-UHFFFADPSC-RAAPJZFEUQ-UHFFFADPSC-NUHFF-NSEGZ-NUHFF-ZZZ

Additional information on the analysis of the target compound is available *via* Chemotion repository:
https://doi.org/10.14272/RAAPJZFEUQUCRF-CMDGGOBGSA-N.1

Neopentyl (*E*)-2-(4-chlorophenyl)ethene-1-sulfonate (8f)

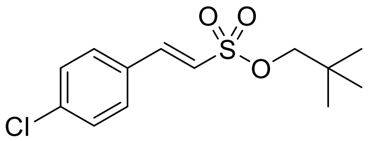

Following the **TP-4**, (*E*)-3-(4-chlorophenyl)acrylic acid (54.8 mg, 0.3 mmol), neopentyl alcohol (79.3 mg, 3.0 equiv.), and SO_2 stock solution (10.0 equiv.) were used as substrates. The residue was purified by flash chromatography over silica gel (*n*-hexane/EtOAc = 98:2) to obtain **8f** as a colorless solid (45.5 mg, 53% yield). Analytical data for **8f** match those previously described in the literature.[42]

mp: 97.3–97.9 °C. R$_f$ = 0.25 (*n*-hexane/EtOAc = 9:1).

^{1}H NMR (400 MHz, $CDCl_3$): δ 7.55 (d, *J* = 15.6 Hz, 1H), 7.48–7.44 (m, 2H), 7.44–7.39 (m, 2H), 6.72 (d, *J* = 15.6 Hz, 1H), 3.81 (s, 2H), 0.98 (s, 9H).

^{13}C{^{1}H}-NMR (101 MHz, $CDCl_3$): δ 143.3, 137.7, 130.6, 129.8, 129.7, 121.8, 79.8, 31.9, 26.2.

IR (ATR) $\tilde{\nu}$ (cm^{-1}): 3056, 2957, 1620, 1359, 1165, 971, 836, 796.

MS (APCI): *m/z* calcd. for $C_{13}H_{18}O_3SCl$ ($[M+H]^+$) 289.1, found 289.4.

HRMS (EI^+): *m/z* calcd. for $C_{13}H_{17}O_2SCl$ ($[M]^+$) 288.0587, found 288.0594.

Additional information on the chemical synthesis is available *via* Chemotion repository:
https://doi.org/10.14272/reaction/SA-FUHFF-UHFFFADPSC-VXGBLHDVVU-UHFFFADPSC-NUHFF-NSEGZ-NUHFF-ZZZ

Additional information on the analysis of the target compound is available *via* Chemotion repository:
https://doi.org/10.14272/VXGBLHDVVUZHMT-CMDGGOBGSA-N.1

Neopentyl (*E*)-2-(4-bromophenyl)ethene-1-sulfonate (8g)

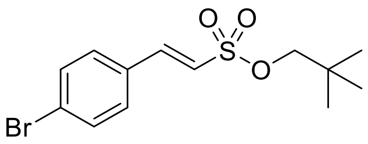

Following the **TP-4**, (*E*)-3-(4-bromophenyl)acrylic acid (68.1 mg, 0.3 mmol), neopentyl alcohol (79.3 mg, 3.0 equiv.), and SO_2 stock solution (10.0 equiv.) were used as substrates. The residue was purified by flash chromatography over silica gel (*n*-hexane/EtOAc = 98:2) to obtain **8g** as a colorless solid (52.5 mg, 53% yield). Analytical data for **8g** match those previously described in the literature.[42]

mp: 133.3–134.1 °C. R_f = 0.25 (*n*-hexane/EtOAc = 9:1).

^{1}H NMR (400 MHz, $CDCl_3$): δ 7.60–7.56 (m, 2H), 7.54 (d, *J* = 15.6 Hz, 1H), 7.41–7.36 (m, 2H), 6.75 (d, *J* = 15.6 Hz, 1H), 3.81 (s, 2H), 0.98 (s, 9H).

^{13}C{^{1}H}-NMR (101 MHz, $CDCl_3$): δ 143.4, 132.6, 131.0, 129.9, 126.0, 121.9, 79.8, 31.9, 26.2.

IR (ATR) $\tilde{\nu}$ (cm^{-1}): 2955, 1620, 1358, 1162, 975, 795, 566.

MS (APCI): *m/z* calcd. for $C_{13}H_{18}O_3SBr$ ($[M+H]^+$) 333.0, found 333.5.

HRMS (EI^+): *m/z* calcd. for $C_{13}H_{17}O_3SBr$ ($[M]^+$) 332.0082, found 332.0086.

Additional information on the chemical synthesis is available *via* Chemotion repository:
https://doi.org/10.14272/reaction/SA-FUHFF-UHFFFADPSC-UBHWQYVHFI-UHFFFADPSC-

NUHFF-NSEGZ-NUHFF-ZZZ

Additional information on the analysis of the target compound is available *via* Chemotion repository:

https://doi.org/10.14272/UBHWQYVHFIXUHK-CMDGGOBGSA-N.1

Neopentyl (*E*)-2-(3-bromophenyl)ethene-1-sulfonate (8h)

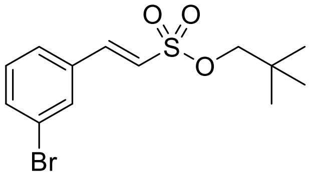

Following the **TP-4**, (*E*)-3-(3-bromophenyl)acrylic acid (68.1 mg, 0.3 mmol), neopentyl alcohol (79.3 mg, 3.0 equiv.), and SO_2 stock solution (10.0 equiv.) were used as substrates. The residue was purified by flash chromatography over silica gel (*n*-hexane/EtOAc = 98:2) to obtain **8h** as a colorless solid (60.0 mg, 60% yield).

mp: 65.2–66.1 °C. R_f = 0.30 (*n*-hexane/EtOAc = 9:1).

^{1}H NMR (400 MHz, $CDCl_3$): δ 7.66 (s, 1H), 7.58 (d, *J* = 7.8 Hz, 1H), 7.52 (d, *J* = 15.6 Hz, 1H), 7.44 (d, *J* = 7.8 Hz, 1H), 7.32 (t, *J* = 7.8 Hz, 1H), 6.76 (d, *J* = 15.6 Hz, 1H), 3.81 (s, 2H), 0.99 (s, 9H).

^{13}C{^{1}H}-NMR (101 MHz, $CDCl_3$): δ 143.0, 134.3, 134.2, 131.2, 130.8, 127.2, 123.4, 122.8, 79.9, 31.9, 26.2.

IR (ATR) $\tilde{\nu}$ (cm^{-1}): 3063, 2959, 1621, 1348, 1161, 955, 773, 540.

MS (APCI): *m/z* calcd. for $C_{13}H_{18}O_3SBr$ ($[M+H]^+$) 333.0, found 333.1.

HRMS (EI^+): *m/z* calcd. for $C_{13}H_{17}O_3SBr$ ($[M]^+$) 332.0082, found 332.0090.

Additional information on the chemical synthesis is available *via* Chemotion repository:

https://doi.org/10.14272/reaction/SA-FUHFF-UHFFFADPSC-IWWPEDCMKL-UHFFFADPSC-NUHFF-NSXYH-NUHFF-ZZZ

Additional information on the analysis of the target compound is available *via* Chemotion repository:

https://doi.org/10.14272/IWWPEDCMKLEIIS-BQYQJAHWSA-N.1

Neopentyl (*E*)-2-(2-bromophenyl)ethene-1-sulfonate (8i)

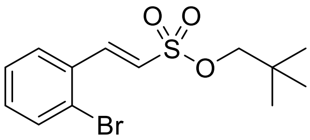

Following the **TP-4**, (*E*)-3-(2-bromophenyl)acrylic acid (68.1 mg, 0.3 mmol), neopentyl alcohol (79.3 mg, 3.0 equiv.), and SO_2 stock solution (10.0 equiv.) were used as substrates. The residue was purified by flash chromatography over silica gel (*n*-hexane/EtOAc = 98:2) to obtain **8i** as a colorless solid (53.0 mg, 53% yield).

mp: 74.6–75.4 °C. R_f = 0.20 (*n*-hexane/EtOAc = 9:1).

^{1}H NMR (400 MHz, $CDCl_3$): δ 7.97 (d, *J* = 15.6 Hz, 1H), 7.66 (dd, *J* = 7.8, 1.4 Hz, 1H), 7.55 (dd, *J* = 7.8, 1.4 Hz, 1H), 7.38 (t, *J* = 7.8 Hz, 1H), 7.31 (td, *J* = 7.8, 1.4 Hz, 1H), 6.72 (d, *J* = 15.6 Hz, 1H), 3.84 (s, 2H), 1.00 (s, 9H).

^{13}C{^{1}H}-NMR (101 MHz, $CDCl_3$): δ 143.2, 133.9, 132.5, 132.4, 128.3, 128.1, 125.5, 124.2, 80.0, 31.9, 26.2.

IR (ATR) $\tilde{v}$ (cm^{-1}): 3063, 2966, 1624, 1341, 1159, 956, 750, 546.

MS (APCI): *m/z* calcd. for $C_{13}H_{18}O_3SBr$ ($[M+H]^+$) 333.0, found 333.5.

HRMS (EI^+): *m/z* calcd. for $C_{13}H_{17}O_3SBr$ ($[M]^+$) 332.0082, found 332.0093.

Additional information on the chemical synthesis is available *via* Chemotion repository:
https://doi.org/10.14272/reaction/SA-FUHFF-UHFFFADPSC-CAPWWSFATX-UHFFFADPSC-NUHFF-NSEGZ-NUHFF-ZZZ

Additional information on the analysis of the target compound is available *via* Chemotion repository:
https://doi.org/10.14272/CAPWWSFATXLFOT-CMDGGOBGSA-N.1

(*E*)-4-(2-((Neopentyloxy)sulfonyl)vinyl)phenyl acetate (8j)

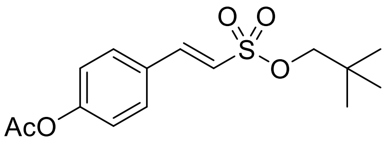

Following the **TP-4**, (*E*)-3-(4-acetoxyphenyl)acrylic acid (61.9 mg, 0.3 mmol), neopentyl alcohol (79.3 mg, 3.0 equiv.), and SO_2 stock solution (10.0 equiv.) were used as substrates. The residue was purified by flash chromatography over silica gel (*n*-hexane/EtOAc = 98:2) to obtain **8j** as a colorless solid (51.5 mg, 55% yield). Analytical data for **8j** match those previously described in the literature.[42]

mp: 118.5–119.0 °C. R_f = 0.20 (*n*-hexane/EtOAc = 9:1).

^{1}H NMR (400 MHz, $CDCl_3$): δ 7.58 (d, *J* = 15.6 Hz, 1H), 7.55–7.52 (m, 2H), 7.20–7.15 (m, 2H), 6.70 (d, *J* = 15.6 Hz, 1H), 3.79 (s, 2H), 2.32 (s, 3H), 0.98 (s, 9H).

^{13}C{^{1}H}-NMR (101 MHz, $CDCl_3$): δ 169.1, 153.0, 143.6, 129.81, 129.76, 122.6, 121.2, 79.7, 31.8, 26.2, 21.2.

IR (ATR) $\tilde{v}$ (cm^{-1}): 3063, 2960, 1761, 1621, 1345, 1159, 955, 802.

MS (APCI): *m/z* calcd. for $C_{15}H_{21}O_5S$ ($[M+H]^+$) 313.4, found 313.5.

HRMS (EI^+): *m/z* calcd. for $C_{15}H_{20}O_5S$ ($[M]^+$) 312.1031, found 312.1041.

Additional information on the chemical synthesis is available *via* Chemotion repository:
https://doi.org/10.14272/reaction/SA-FUHFF-UHFFFADPSC-XSFAWQKHXV-UHFFFADPSC-NUHFF-NAIXQ-NUHFF-ZZZ

Additional information on the analysis of the target compound is available *via* Chemotion repository:
https://doi.org/10.14272/XSFAWQKHXVRJFW-MDZDMXLPSA-N.1

(*E*)-2-Methoxy-4-(2-((neopentyloxy)sulfonyl)vinyl)phenyl acetate (8k)

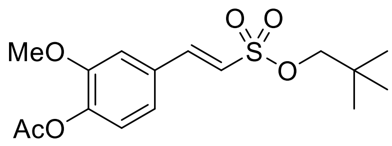

Following the **TP-4**, (*E*)-3-(4-acetoxy-3-methoxyphenyl)acrylic acid (70.9 mg, 0.3 mmol), neopentyl alcohol (79.3 mg, 3.0 equiv.), and SO_2 stock solution (10.0 equiv.) were used as substrates. The residue was purified by flash chromatography over silica gel (*n*-hexane/EtOAc = 90:10) to obtain **8k** as a colorless solid (20.9 mg, 20% yield).

mp: 136.3–136.8 °C. R_f = 0.30 (*n*-hexane/EtOAc = 4:1).

^{1}H NMR (400 MHz, $CDCl_3$): δ 7.55 (d, *J* = 15.6 Hz, 1H), 7.15–7.05 (m, 3H), 6.69 (d, *J* = 15.6 Hz, 1H), 3.88 (s, 3H), 3.79 (s, 2H), 2.33 (s, 3H), 0.98 (s, 9H).

^{13}C{^{1}H}-NMR (101 MHz, $CDCl_3$): δ 168.8, 151.8, 144.1, 142.4, 131.0, 123.7, 121.7, 121.3, 111.8, 79.8, 56.1, 31.9, 26.2, 20.8.

IR (ATR) $\tilde{\nu}$ (cm^{-1}): 3063, 2966, 1760, 1618, 1354, 1161, 951.

MS (APCI): *m/z* calcd. for $C_{16}H_{23}O_6S$ ($[M+H]^+$) 343.1, found 343.2.

HRMS (ESI^+): *m/z* calcd. for $C_{16}H_{22}O_6SNa$ ($[M+Na]^+$) 365.102931, found 365.102900.

Additional information on the chemical synthesis is available *via* Chemotion repository:
https://doi.org/10.14272/reaction/SA-FUHFF-UHFFFADPSC-VWCNVJOEAV-UHFFFADPSC-NUHFF-NSEGZ-NUHFF-ZZZ

Additional information on the analysis of the target compound is available *via* Chemotion repository:
https://doi.org/10.14272/VWCNVJOEAVXQIX-CMDGGOBGSA-N.1

Neopentyl (*E*)-2-(3,4,5-trimethoxyphenyl)ethene-1-sulfonate (8l)

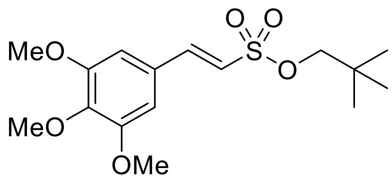

Following the **TP-4**, (*E*)-3-(3,4,5-trimethoxyphenyl)acrylic acid (71.5 mg, 0.3 mmol), neopentyl alcohol (79.3 mg, 3.0 equiv.), and SO_2 stock solution (10.0 equiv.) were used as substrates. The residue was purified by flash chromatography over silica gel (*n*-hexane/EtOAc = 93:7) to obtain **8l** as a colorless solid (42.3 mg, 41% yield).

mp: 110.9–111.2 °C. R_f = 0.25 (*n*-hexane/EtOAc = 4:1).

^{1}H NMR (400 MHz, $CDCl_3$): δ 7.51 (d, *J* = 15.6 Hz, 1H), 6.74 (s, 2H), 6.67 (d, *J* = 15.6 Hz, 1H), 3.89 (s, 9H), 3.79 (s, 2H), 0.97 (s, 9H).

^{13}C{^{1}H}-NMR (101 MHz, $CDCl_3$): δ 153.7, 144.8, 141.1, 127.4, 120.1, 105.8, 79.6, 61.1, 56.3, 31.8, 26.2.

IR (ATR) $\tilde{v}$ (cm^{-1}): 3059, 2947, 1614, 1336, 1158, 958, 789.

MS (APCI): *m/z* calcd. for $C_{16}H_{25}O_6S$ ($[M+H]^+$) 345.1, found 345.6.

HRMS (EI^+): *m/z* calcd. for $C_{16}H_{24}O_6S$ ($[M]^+$) 344.1294, found 344.1308.

Additional information on the chemical synthesis is available *via* Chemotion repository:
https://doi.org/10.14272/reaction/SA-FUHFF-UHFFFADPSC-YQRZVSXQCQ-UHFFFADPSC-NUHFF-NSXYH-NUHFF-ZZZ

Additional information on the analysis of the target compound is available *via* Chemotion repository:
https://doi.org/10.14272/YQRZVSXQCQZRBP-BQYQJAHWSA-N.1

Neopentyl (*E*)-2-(naphthalen-1-yl)ethene-1-sulfonate (8m)

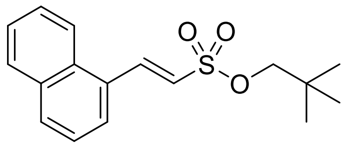

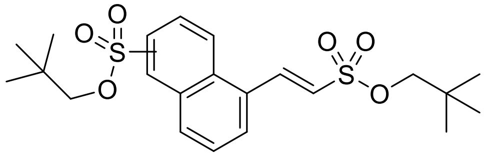

Following the **TP-4**, (*E*)-3-(naphthalen-1-yl)acrylic acid (59.5 mg, 0.3 mmol), neopentyl alcohol (79.3 mg, 3.0 equiv.), and SO_2 stock solution (10.0 equiv.) were used as substrates. The residue was purified by flash chromatography over silica gel (*n*-hexane/EtOAc = 98:2) to obtain **8m** as a colorless solid (26.9 mg, 30% yield) together with the side product **10m** (21.8 mg, 16% yield, colorless solid).

Analytica data for **8m**:

mp: 76.1–77.1 °C. R_f = 0.31 (*n*-hexane/EtOAc = 9:1).

^{1}H NMR (400 MHz, $CDCl_3$): δ 8.43 (d, *J* = 15.4 Hz, 1H), 8.13 (d, *J* = 8.4 Hz, 1H), 7.97 (d, *J* = 8.4 Hz, 1H), 7.91 (d, *J* = 7.4 Hz, 1H), 7.73 (d, *J* = 7.4 Hz, 1H), 7.65–7.60 (m, 1H), 7.60–7.55 (m, 1H), 7.52 (t, *J* = 7.4 Hz, 1H), 6.85 (d, *J* = 15.4 Hz, 1H), 3.87 (s, 2H), 1.01 (s, 9H).

^{13}C{^{1}H}-NMR (101 MHz, $CDCl_3$): δ 141.9, 133.8, 131.8, 131.3, 129.4, 129.1, 127.6, 126.8, 125.8, 125.5, 123.6, 123.1, 79.8, 31.9, 26.3.

IR (ATR) $\tilde{\nu}$ (cm^{-1}): 3066, 2965, 1612, 1344, 1162, 953, 863, 793.

MS (APCI): *m/z* calcd. for $C_{17}H_{21}O_3S$ ($[M+H]^+$) 305.1, found 305.5.

HRMS (EI^+): *m/z* calcd. for $C_{17}H_{20}O_3S$ ($[M]^+$) 304.1133, found 304.1134.

Analytica data for **10m**:

10m was formed as single regioisomer. However, the regiochemistry of **10m** could not be assigned unambiguously within this study.

mp: 133.1–134.1 °C. R_f = 0.20 (*n*-hexane/EtOAc = 9:1).

^{1}H NMR (400 MHz, $CDCl_3$): δ 8.72 (d, *J* = 8.4 Hz, 1H), 8.40 (d, *J* = 15.4 Hz, 1H), 8.30 (d, *J* = 7.4 Hz, 1H), 8.18 (d, *J* = 8.4 Hz, 1H), 7.82–7.77 (m, 1H), 7.77–7.72 (m, 2H), 6.94 (d, *J* = 15.4 Hz, 1H), 3.92 (s, 2H), 3.66 (s, 2H), 1.02 (s, 9H), 0.85 (s, 9H).

^{13}C{^{1}H}-NMR (101 MHz, $CDCl_3$): δ 140.5, 136.4, 134.0, 131.9, 129.7, 129.2, 128.8, 128.6, 127.5, 126.1, 124.2, 123.8, 80.4, 80.2, 32.0, 31.7, 26.24, 26.16.

IR (ATR) $\tilde{\nu}$ (cm^{-1}): 3060, 2960, 1609, 1345, 1166, 952, 818.

MS (APCI): *m/z* calcd. for $C_{44}H_{61}O_{12}S_4$ ([2M+H]$^+$) 909.3, found 908.9.

HRMS (EI$^+$): *m/z* calcd. for $C_{22}H_{30}O_6S_2$ ([M]$^+$) 454.1484, found 454.1497.

Additional information on the chemical synthesis is available *via* Chemotion repository: https://doi.org/10.14272/reaction/SA-FUHFF-UHFFFADPSC-NNWNPNNOQF-UHFFFADPSC-NUHFF-NZJBK-NUHFF-ZZZ

Additional information on the analysis of the target compound is available *via* Chemotion repository:

https://doi.org/10.14272/NNWNPNNOQFFLLH-VAWYXSNFSA-N.1

Neopentyl (*E*)-2-(naphthalen-2-yl)ethene-1-sulfonate (8n)

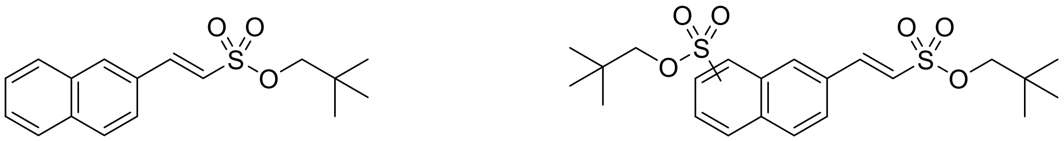

Following the **TP-4**, (*E*)-3-(naphthalen-2-yl)acrylic acid (59.5 mg, 0.3 mmol), neopentyl alcohol (79.3 mg, 3.0 equiv.), and SO_2 stock solution (10.0 equiv.) were used as substrates. The residue was purified by flash chromatography over silica gel (*n*-hexane/EtOAc = 98:2) to obtain **8n** as a colorless solid (26.0 mg, 28% yield) together with the side product **10n** (3.7 mg, 3% yield, colorless liquid).

Analytica data for **8n**:

mp: 117.1–117.8 °C. R_f = 0.31 (*n*-hexane/EtOAc = 9:1).

^{1}H NMR (400 MHz, $CDCl_3$): δ 7.96 (s, 1H), 7.91–7.84 (m, 3H), 7.76 (d, *J* = 15.4 Hz, 1H), 7.61 (dd, *J* = 8.6, 1.6 Hz, 1H), 7.59–7.52 (m, 2H), 6.84 (d, *J* = 15.4 Hz, 1H), 3.83 (s, 2H), 0.99 (s, 9H).

^{13}C{^{1}H}-NMR (101 MHz, $CDCl_3$): δ 144.9, 134.7, 133.2, 131.1, 129.6, 129.3, 128.9, 128.1, 128.0, 127.3, 123.3, 121.2, 79.7, 31.9, 26.2.

IR (ATR) $\tilde{\nu}$ (cm^{-1}): 3060, 2966, 1612, 1349, 1162, 951, 860, 806.

MS (APCI): *m/z* calcd. for $C_{17}H_{21}O_3S$ ([M+H]$^+$) 305.1, found 305.5.

HRMS (EI$^+$): *m/z* calcd. for $C_{17}H_{20}O_3S$ ([M]$^+$) 304.1133, found 304.1131.

Analytica data for **10n**:

10n was formed as single regioisomer. However, the regiochemistry of **10n** could not be assigned unambiguously within this study.

R_f = 0.20 (*n*-hexane/EtOAc = 9:1).

^{1}H NMR (400 MHz, $CDCl_3$): δ 8.83 (d, *J* = 8.4 Hz, 1H), 8.61 (d, *J* = 15.4 Hz, 1H), 8.15 (d, *J* = 8.4 Hz, 1H), 7.95 (d, *J* = 8.4 Hz, 1H), 7.74 (td, *J* = 8.4, 1.4 Hz, 1H), 7.66 (td, *J* = 8.4, 1.4 Hz, 1H), 7.50 (d, *J* = 8.4 Hz, 1H), 6.56 (d, *J* = 15.4 Hz, 1H), 3.98 (s, 2H), 3.66 (s, 2H), 1.00 (s, 9H), 0.86 (s, 9H).

^{13}C{^{1}H}-NMR (101 MHz, $CDCl_3$): δ 145.5, 135.4, 134.7, 134.6, 130.7, 129.5, 129.4, 128.9, 128.2, 126.2, 126.0, 125.9, 80.5, 80.2, 31.9, 31.8, 26.22, 26.21.

IR (ATR) $\tilde{\nu}$ (cm^{-1}): 3005, 2970, 1712, 1359, 1219, 962, 848.

MS (APCI): *m/z* calcd. for $C_{22}H_{31}O_6S_2$ ($[M+H]^+$) 455.2, found 455.6.

HRMS (EI^+): *m/z* calcd. for $C_{22}H_{30}O_6S_2$ ($[M]^+$) 454.1184, found 454.1181.

Additional information on the chemical synthesis is available *via* Chemotion repository:
https://doi.org/10.14272/reaction/SA-FUHFF-UHFFFADPSC-RBMWJOBEYR-UHFFFADPSC-NUHFF-NZAEC-NUHFF-ZZZ

Additional information on the analysis of the target compound is available *via* Chemotion repository:
https://doi.org/10.14272/RBMWJOBEYRKEOU-ZHACJKMWSA-N.1

Neopentyl (*E*)-2-(furan-2-yl)ethene-1-sulfonate (8o)

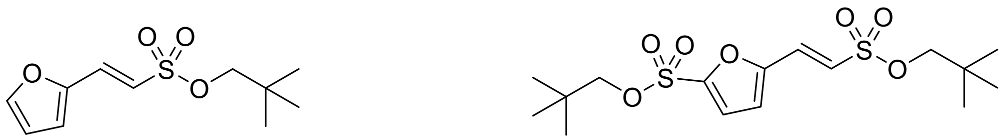

Following the **TP-4**, (*E*)-3-(furan-2-yl)acrylic acid (41.4 mg, 0.3 mmol), neopentyl alcohol (79.3 mg, 3.0 equiv.), and SO_2 stock solution (10.0 equiv.) were used as substrates. The residue was purified by flash chromatography over silica gel (*n*-hexane/EtOAc = 98:2) to obtain **8o** as a colorless liquid (7.9 mg, 11%) together with the side product **10o** (45.3 mg, 38% yield, colorless solid).

Analytica data for **8o**:

R_f = 0.25 (*n*-hexane/EtOAc = 9:1).

^{1}H NMR (400 MHz, $CDCl_3$): δ 7.55–7.52 (m, 1H), 7.35 (d, *J* = 15.2 Hz, 1H), 6.72 (d, *J* = 3.4 Hz, 1H), 6.61 (d, *J* = 15.2 Hz, 1H), 6.52 (dd, *J* = 3.4, 1.6 Hz, 1H), 3.78 (s, 2H), 0.98 (s, 9H).

^{13}C{^{1}H}-NMR (101 MHz, $CDCl_3$): δ 148.4, 145.9, 131.1, 118.5, 117.2, 112.8, 79.7, 31.9, 26.2.

IR (ATR) $\tilde{\nu}$ (cm^{-1}): 3005, 2965, 1711, 1358, 1219, 1091.

MS (APCI): *m/z* calcd. for $C_{11}H_{17}O_4S$ ($[M+H]^+$) 245.1, found 245.3.

HRMS (EI^+): *m/z* calcd. for $C_{11}H_{16}O_4S$ ($[M]^+$) 244.0769, found 244.0785.

Analytica data for **10o**:

mp: 112.6–113.0 °C. R_f = 0.13 (*n*-hexane/EtOAc = 9:1).

^{1}H NMR (400 MHz, $CDCl_3$): δ 7.37 (d, *J* = 15.4 Hz, 1H), 7.21 (d, *J* = 3.6 Hz, 1H), 6.87 (d, *J* = 15.4 Hz, 1H), 6.81 (d, *J* = 3.6 Hz, 1H), 3.88 (s, 2H), 3.83 (s, 2H), 0.99 (s, 9H), 0.95 (s, 9H).

^{13}C{^{1}H}-NMR (101 MHz, $CDCl_3$): δ 151.9, 147.0, 129.2, 124.0, 119.9, 116.3, 81.8, 80.3, 31.94, 31.90, 26.2, 26.0.

IR (ATR) $\tilde{\nu}$ (cm^{-1}): 3003, 2969, 1711, 1358, 1219, 1092.

MS (APCI): *m/z* calcd. for $C_{16}H_{27}O_7S_2$ ($[M+H]^+$) 395.1, found 395.1.

HRMS (EI^+): *m/z* calcd. for $C_{16}H_{26}O_7S_2$ ($[M]^+$) 394.1120, found 394.1134.

Additional information on the chemical synthesis is available *via* Chemotion repository:
https://doi.org/10.14272/reaction/SA-FUHFF-UHFFFADPSC-MJDINSPTRU-UHFFFADPSC-NUHFF-NXLOP-NUHFF-ZZZ

Additional information on the analysis of the target compound is available *via* Chemotion repository:

https://doi.org/10.14272/GOJGFMQYZBAXMX-SOFGYWHQSA-N.1

https://doi.org/10.14272/JXPGRUREMBXEGE-MDZDMXLPSA-N.1

Neopentyl (*E*)-2-(thiophen-2-yl)ethene-1-sulfonate (8p)

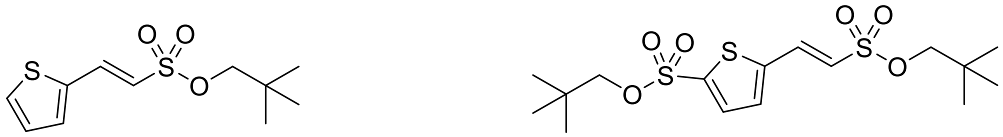

Following the **TP-4**, (*E*)-3-(thiophen-2-yl)acrylic acid (46.3 mg, 0.3 mmol), neopentyl alcohol (79.3 mg, 3.0 equiv.), and SO_2 stock solution (10.0 equiv.) were used as substrates. The residue was purified by flash chromatography over silica gel (*n*-hexane/EtOAc = 98:2) to obtain **8p** as a colorless solid (24.2 mg, 31% yield) together with the side product **10p** (36.6 mg, 30% yield,

colorless solid).

Analytica data for **8p**:

mp: 60.2–60.8 °C. R_f = 0.25 (*n*-hexane/EtOAc = 9:1).

^{1}H NMR (400 MHz, $CDCl_3$): δ 7.70 (d, *J* = 15.2 Hz, 1H), 7.49 (d, *J* = 5.0 Hz, 1H), 7.34 (d, *J* = 3.6 Hz, 1H), 7.11 (dd, *J* = 5.0, 3.6 Hz, 1H), 6.52 (d, *J* = 15.2 Hz, 1H), 3.79 (s, 2H), 0.98 (s, 9H).

^{13}C{^{1}H}-NMR (101 MHz, $CDCl_3$): δ 137.3, 136.6, 132.7, 130.2, 128.6, 119.2, 79.7, 31.9, 26.2.

IR (ATR) $\tilde{\nu}$ (cm^{-1}): 3067, 2962, 1602, 1348, 1159, 955.

MS (APCI): *m/z* calcd. for $C_{11}H_{17}O_3S_2$ ([M+H]$^+$) 261.1, found 261.3.

HRMS (EI$^+$): *m/z* calcd. for $C_{11}H_{16}O_3S_2$ ([M]$^+$) 260.0541, found 260.0554.

Analytica data for **10p**:

mp: 125.1–125.9 °C. R_f = 0.15 (*n*-hexane/EtOAc = 9:1).

^{1}H NMR (400 MHz, $CDCl_3$): δ 7.66 (d, *J* = 15.4 Hz, 1H), 7.65 (d, *J* = 4.0 Hz, 1H), 7.33 (d, *J* = 4.0 Hz, 1H), 6.70 (d, *J* = 15.4 Hz, 1H), 3.84 (s, 2H), 3.82 (s, 2H), 0.99 (s, 9H), 0.95 (s, 9H).

^{13}C{^{1}H}-NMR (101 MHz, $CDCl_3$): δ 143.3, 139.0, 135.1, 134.4, 131.2, 123.9, 81.2, 80.3, 31.92, 31.86, 26.2, 26.1.

IR (ATR) $\tilde{\nu}$ (cm^{-1}): 3059, 2959, 1614, 1356, 1164, 952.

MS (APCI): *m/z* calcd. for $C_{16}H_{27}O_6S_3$ ([M+H]$^+$) 411.1, found 411.5.

HRMS (EI$^+$): *m/z* calcd. for $C_{16}H_{26}O_6S_3$ ([M]$^+$) 410.0892, found 410.0913.

Additional information on the chemical synthesis is available *via* Chemotion repository:
https://doi.org/10.14272/reaction/SA-FUHFF-UHFFFADPSC-ZREWVIAMZP-UHFFFADPSC-NUHFF-NXLOP-NUHFF-ZZZ

Additional information on the analysis of the target compound is available *via* Chemotion repository:
https://doi.org/10.14272/JHKIXXQIDLWUAN-SOFGYWHQSA-N.1
https://doi.org/10.14272/MPYVFYCPJCYBAC-MDZDMXLPSA-N.1

Neopentyl (*E*-*Z*)-1-phenylprop-1-ene-2-sulfonate (8q)

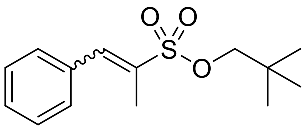

Following the **TP-4**, (*E*)-2-methyl-3-phenylacrylic acid (48.7 mg, 0.3 mmol), neopentyl alcohol (79.3 mg, 3.0 equiv.), and SO_2 stock solution (10.0 equiv.) were used as substrates. The residue was purified by flash chromatography over silica gel (*n*-hexane/EtOAc = 99:1) to obtain **8q** as a colorless liquid as inseparable mixture of the *E*- and the *Z*-isomer (22.4 mg, 28% yield, *E*:*Z* = 3.5:1).

R_f = 0.48 (*n*-hexane/EtOAc = 9:1).

8q-(*E*): **^{1}H NMR** (400 MHz, $CDCl_3$): δ 7.60 (s, 1H), 7.48–7.41 (m, 5H), 3.75 (s, 2H), 2.29 (d, J = 1.4 Hz, 3H), 0.99 (s, 9H).

8q-(*Z*): **^{1}H NMR** (400 MHz, $CDCl_3$): δ 7.41–7.31 (m, 5H), 7.06 (s, 1H), 3.60 (s, 2H), 2.29 (d, J = 1.4 Hz, 3H), 0.81 (s, 9H).

8q-(*E*): **^{13}C{^{1}H}-NMR** (101 MHz, $CDCl_3$): δ 139.0, 129.8, 129.7, 129.4, 128.9, 128.1, 79.5, 31.8, 26.3, 13.7.

8q-(*Z*): **^{13}C{^{1}H}-NMR** (101 MHz, $CDCl_3$): δ 138.7, 133.9, 133.5, 133.3, 132.6, 129.2, 128.9, 125.6, 79.3, 31.6, 26.0, 21.5.

IR (ATR) $\tilde{\nu}$ (cm^{-1}): 3059, 2960, 1345, 1176, 963, 836.

MS (APCI): *m/z* calcd. for $C_{14}H_{21}O_3S$ ($[M+H]^+$) 269.1, found 269.1.

HRMS (EI^+): *m/z* calcd. for $C_{14}H_{20}O_3S$ ($[M]^+$) 268.112768, found 268.112960.

The stereoisomeric ratio was determined to be 3.5/1 (E/Z) by NMR integration of the corresponding methylene signals.

Additional information on the chemical synthesis is available *via* Chemotion repository:
https://doi.org/10.14272/reaction/SA-FUHFF-UHFFFADPSC-YBXNPAWLMZ-UHFFFADPSC-NUHFF-NUHFF-NUHFF-ZZZ
Additional information on the analysis of the target compound is available *via* Chemotion repository:
https://doi.org/10.14272/YBXNPAWLMZRFQT-UHFFFAOYSA-N.1

Neopentyl (*E*)-2-phenylprop-1-ene-1-sulfonate (8r)

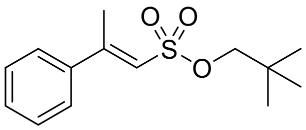

Following the **TP-4**, (*E*)-3-phenylbut-2-enoic acid (48.7 mg, 0.3 mmol), neopentyl alcohol (79.3 mg, 3.0 equiv.), and SO_2 stock solution (10.0 equiv.) were used as substrates. The residue was purified by flash chromatography over silica gel (*n*-hexane/EtOAc = 99:1) to obtain **8r** as a colorless liquid (29.5 mg, 37% yield). Analytical data for **8r** match those previously described in the literature.[42]

R_f = 0.43 (*n*-hexane/EtOAc = 9:1).

^{1}H NMR (400 MHz, $CDCl_3$): δ 7.50–7.38 (m, 5H), 6.50 (d, *J* = 1.2 Hz, 1H), 3.83 (s, 2H), 2.55 (d, *J* = 1.2 Hz, 3H), 1.00 (s, 9H).

^{13}C{^{1}H}-NMR (101 MHz, $CDCl_3$): δ 155.3, 139.8, 130.2, 129.0, 126.4, 121.7, 79.3, 31.8, 26.3, 18.1.

IR (ATR) $\tilde{\nu}$ (cm^{-1}): 3063, 2960, 1352, 1164, 965, 850.

MS (APCI): *m/z* calcd. for $C_{14}H_{21}O_3S$ ($[M+H]^+$) 269.1, found 269.1.

HRMS (EI^+): *m/z* calcd. for $C_{14}H_{20}O_3S$ ($[M]^+$) 268.112768, found 268.112750.

Additional information on the chemical synthesis is available *via* Chemotion repository:
https://doi.org/10.14272/reaction/SA-FUHFF-UHFFFADPSC-GYVBCVAVQX-UHFFFADPSC-NUHFF-NLXOU-NUHFF-ZZZ

Additional information on the analysis of the target compound is available *via* Chemotion repository:
https://doi.org/10.14272/GYVBCVAVQXRFIW-ZRDIBKRKSA-N.1

Neopentyl benzofuran-2-sulfonate (8s)

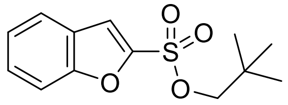

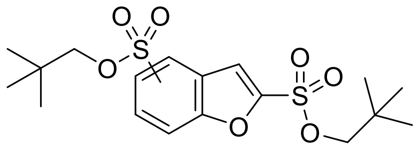

Following the **TP-4**, benzofuran-2-carboxylic acid (49.3 mg, 0.3 mmol), neopentyl alcohol (79.3 mg, 3.0 equiv.), and SO_2 stock solution (10.0 equiv.) were used as substrates. The residue was purified by flash chromatography over silica gel (*n*-hexane/EtOAc = 98:2) to obtain **8s** as a colorless liquid (16.9 mg, 21% yield) together with the side product **10s** (4.5 mg, 4% yield, colorless solid). Analytical data for **8s** match those previously described in the literature.[42]

Analytica data for **8s**:

R_f = 0.38 (*n*-hexane/EtOAc = 9:1).

^{1}H NMR (400 MHz, $CDCl_3$): δ 7.72 (d, *J* = 8.2 Hz, 1H), 7.61 (dd, *J* = 8.2, 0.8 Hz, 1H), 7.55–7.49 (m, 2H), 7.41–7.35 (m, 1H), 3.92 (s, 2H), 0.94 (s, 9H).

^{13}C{^{1}H}-NMR (101 MHz, $CDCl_3$): δ 156.2, 146.6, 128.5, 125.7, 124.6, 123.3, 114.7, 112.6, 81.7, 31.9, 26.1.

IR (ATR) $\tilde{\nu}$ (cm^{-1}): 3129, 2965, 1382, 1172, 932, 756.

MS (APCI): *m/z* calcd. for $C_{13}H_{17}O_4S$ ($[M+H]^+$) 269.1, found 269.3.

HRMS (EI^+): *m/z* calcd. for $C_{13}H_{16}O_4S$ ($[M]^+$) 268.0769, found 268.0781.

Analytica data for **10s**:

10s was formed as single regioisomer. However, the regiochemistry of **10s** could not be assigned unambiguously within this study.

mp: 108.2–109.0 °C. R_f = 0.13 (*n*-hexane/EtOAc = 9:1).

^{1}H NMR (400 MHz, $CDCl_3$): δ 7.97 (dd, *J* = 8.2, 0.8 Hz, 1H), 7.94 (d, *J* = 0.8 Hz, 1H), 7.90 (d, *J* = 8.2 Hz, 1H), 7.71–7.66 (m, 1H), 3.95 (s, 2H), 3.71 (s, 2H), 0.95 (s, 9H), 0.88 (s, 9H).

^{13}C{^{1}H}-NMR (101 MHz, $CDCl_3$): δ 156.0, 149.2, 130.3, 127.9, 125.8, 123.9, 118.2, 113.5, 82.3, 80.5, 32.0, 31.8, 26.1, 26.0.

IR (ATR) $\tilde{\nu}$ (cm^{-1}): 3142, 2962, 1364, 1176, 932, 799.

MS (APCI): *m/z* calcd. for $C_{18}H_{27}O_7S_2$ ($[M+H]^+$) 419.1, found 419.5.

HRMS (EI^+): *m/z* calcd. for $C_{18}H_{26}O_7S_2$ ($[M]^+$) 418.1120, found 418.1137.

Additional information on the chemical synthesis is available *via* Chemotion repository:
https://doi.org/10.14272/reaction/SA-FUHFF-UHFFFADPSC-DOFKASQWDJ-UHFFFADPSC-NUHFF-NUHFF-NUHFF-ZZZ

Additional information on the analysis of the target compound is available *via* Chemotion repository:
https://doi.org/10.14272/DOFKASQWDJPMSY-UHFFFAOYSA-N.1

5.3 Scale-up Reaction

The reaction was carried out using the divided glass cell with a P4 frit as described in section **1.2**.

Anolyte: In a 100 mL pear-shaped flask a mixture of neopentyl alcohol (2.64 g, 30.0 mmol, 3.0 equiv.) and 2,6-lutidine (6.43 g, 6.97 mL, 60.0 mmol, 6.0 equiv.) was prepared. The mixture was chilled to 0 °C in an ice bath and SO_2 stock solution (3.30 M, 30.3 mL, 10.0 equiv.) was added, resulting in a pale yellowish color. The mixture was diluted with acetonitrile (58.6 mL) so that a total volume of 100.0 mL was achieved.
Catholyte: A solution of *n*Bu_4NPF_6 (3.87 g, 10.0 mmol, 1.0 equiv.) and acetic acid (3.00 g, 2.86 mL, 50.0 mmol, 5.0 equiv.) in acetonitrile was prepared so that a total volume of 100.0 mL was reached as well.

The anodic compartment was charged with cinnamic acid (1.48 g, 10.0 mmol, 1.0 equiv.) and the prepared electrolyte mixtures above were transferred to their respective compartments. The cell was equipped with a graphite anode and a graphite cathode. The current was set accordingly so that a current density of 10 $mA\times cm^{-2}$ was reached (90 mA with the setup described herein) and the amount of applied charge was set to 3377 C (corresponding to 3.5 *F*). The electrolysis was conducted at room temperature under constant stirring for 10 h 25 min.

After completion of the electrolysis, both the anolyte and catholyte were combined into a beaker and the compartments were rinsed with acetonitrile (3 x 15 mL each). Water (200 mL) was added. Then the mixture was extracted with EtOAc (4 x 200 mL). The combined organic fractions were washed with water (3 x 100 mL), dried over $MgSO_4$ and the solvent was removed under reduced pressure. The crude product (ca. 12.7 g) was loaded onto silica, purified *via* automated flash column chromatography (cyclohexane/ethyl acetate = 98/2 → 75/25, column: PF-25SiHC-F0080,

detection @ 254 nm) and the desired product neopentyl (*E*)-2-phenylethene-1-sulfonate (**8a**, 1.84 g, 7.22 mmol, 72%) was obtained as a colorless solid.

Spectroscopic data of the isolated compound matched the one obtained from the small-scale reaction described in section **5.2**.
Note: An ^{1}H NMR yield of 70% was calculated by addition of triphenylmethane (684.4 mg, 2.80 mmol) as internal standard to the crude product before column chromatography.

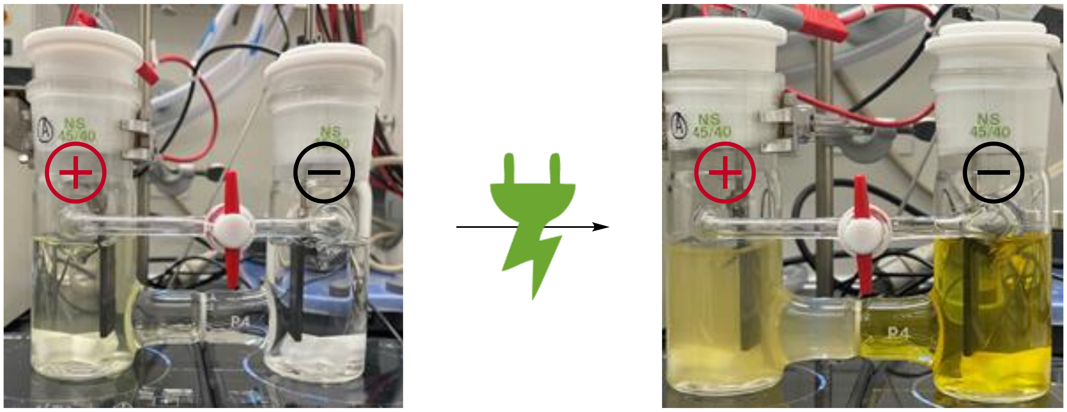

Figure S4: Scale-up experiment in a 2 x 100 mL glass cell before (left) and after (right) the electrolysis.

5.4 Reusability Tests

The reactions were carried out using IKA Pro-Divide cells with a glass frit membrane as described in the section before.

Anolyte: An oven-dried 10.0 mL pear-shaped flask was charged with cinnamic acid (44.5 mg, 0.3 mmol), neopentyl alcohol (79.3 mg, 3.0 equiv.), nBu$_4$NPF$_6$ (116.3 mg, 1.0 equiv.), and anhydrous acetonitrile (2.1 mL). The mixture was cooled to 0 °C in an ice bath, followed by the addition of SO_2 stock solution (4.60 M, 0.65 mL, 10.0 equiv.) and 2,6-lutidine (0.21 mL, 6.0 equiv.) so that a total volume of 3.0 mL was achieved.
Catholyte: An oven-dried 10.0 mL pear-shaped flask was charged with nBu$_4$NPF$_6$ (116.3 mg, 1.0 equiv.), acetic acid (0.09 mL, 5.0 equiv.), and anhydrous acetonitrile (2.9 mL) so that a total volume of 3.0 mL was achieved as well.
The reaction mixtures were transferred with syringes to their respective compartment

simultaneously. The amperage was set accordingly so that a current density of 10 mA×cm^{-2} was reached (8.8 mA with the setup described herein) and the amount of applied charge was set to 3.5 *F*. The electrolysis was conducted at room temperature under constant stirring (400 rpm) for ca. 3 h 11 min. After completion of the electrolysis, the anolyte was transferred to a round bottom flask, the anode compartment was rinsed with acetonitrile (2 x 3.0 mL). ^{1}H NMR yields were calculated by addition of triphenylmethane (73.2 mg, 1.0 equiv.) as the internal standard to this mixture. After 5 min of stirring, 1.0 mL of the mixture was taken, and the solvent was removed under reduced pressure.

For the subsequent runs, the anolyte was prepared as described and transferred to the respective compartment. The graphite electrode, glass frit membrane and catholyte (an additional amount of 0.07 mL acetic acid (4.0 equiv.) was added for each subsequent run to compensate for the loss from H_2 evolution from the previous experiment) were reused in the following experiments. The electrolysis and subsequent workup for determining ^{1}H NMR yield was conducted as described above. The complete procedure was conducted four times in total (**Figure S5**).
During the reusability test, gradual formation of a polymer layer was observed at the anode (**Figure S6**). Attempts to analyze the nature of the polymer layer were unsuccessful.

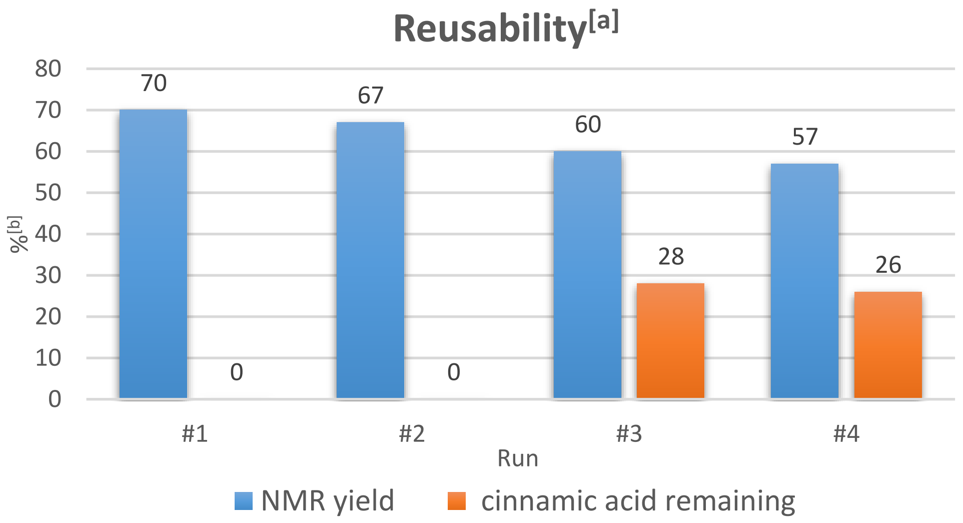

Figure S5: Tests for reusing the electrodes, glass frit membrane and catholyte. [a]Conditions described above. [b]^{1}H-NMR yield with the use of $CHPh_3$ as the internal standard.

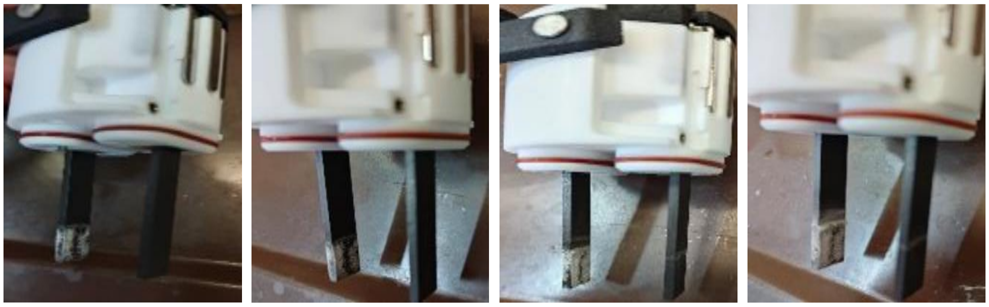

Figure S6: Pictures of the electrodes after run 1 to 4 (from left to right) show the consecutive formation of a polymer layer after each run.

6 Synthesis of Alkyl Alkenesulfonates [Scope of Alcohols]

6.1 Typical Procedure for the Preparation of Alkyl Alkenesulfonates [Scope of Alcohols] (TP-5)

The reactions were carried out using IKA Pro-Divide cells with a glass frit membrane.

Anolyte: An oven-dried 10.0 mL pear-shaped flask was charged with cinnamic acid (44.5 mg, 0.3 mmol), alcohol (3.0 equiv.), *n*Bu_4NPF_6 (116.3 mg, 1.0 equiv.), and anhydrous acetonitrile. The mixture was cooled to 0 °C in an ice bath, followed by the addition of SO_2 stock solution (0.6 mL, 5.0 M in acetonitrile, 10.0 equiv.) and 2,6-lutidine (0.21 mL, 6.0 equiv.) so that a total volume of 3.0 mL was achieved.
Catholyte: An oven-dried 10.0 mL pear-shaped flask was charged with *n*Bu_4NPF_6 (116.3 mg, 1.0 equiv.), acetic acid (0.09 mL, 5.0 equiv.), and anhydrous acetonitrile (2.9 mL) so that a total volume of 3.0 mL was achieved as well.

The reaction mixtures were transferred with syringes to their respective compartment simultaneously. The amperage was set accordingly so that a current density of 10 $mA \times cm^{-2}$ was reached (8.8 mA with the setup described herein) and the amount of applied charge was set to 3.5 *F*. The electrolysis was conducted at room temperature under constant stirring (400 rpm) for ca. 3 h 11 min.

After completion of the electrolysis, the anolyte was transferred to a separatory funnel and the anode compartment was rinsed with additional EtOAc (2 x 3.0 mL). Distilled water (10.0 mL) was added and and the obtained mixture extracted with EtOAc (2 x 10.0 mL). The organic layer was

dried over anhydrous Na_2SO_4 and concentrated in vacuo. The crude residue was subjected to flash column chromatography on silica gel to obtain the product.

6.2 Analytical Data for Compounds of Type 9

Methyl (*E*)-2-phenylethene-1-sulfonate (9a)

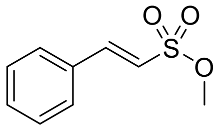

Following the **TP-5**, cinnamic acid (44.5 mg, 0.3 mmol), methanol (36.4 µL, 3.0 equiv.), and SO_2 stock solution (10.0 equiv.) were used as substrates. The residue was purified by flash chromatography over silica gel (*n*-hexane/EtOAc = 90:10) to obtain **9a** as a colorless solid (34.0 mg, 57% yield).

mp: 65.3–65.8 °C. R_f = 0.21 (*n*-hexane/EtOAc = 4:1).

^{1}H NMR (400 MHz, $CDCl_3$): δ 7.63 (d, *J* = 15.6 Hz, 1H), 7.55–7.50 (m, 2H), 7.50–7.41 (m, 3H), 6.73 (d, *J* = 15.6 Hz, 1H), 3.86 (s, 3H).

^{13}C{^{1}H}-NMR (101 MHz, $CDCl_3$): δ 145.5, 132.0, 131.7, 129.3, 128.7, 120.3, 56.3.

IR (ATR) $\tilde{\nu}$ (cm^{-1}): 3072, 2959, 1618, 1344, 1154, 963, 739.

MS (APCI): *m/z* calcd. for $C_9H_{11}O_3S$ ($[M+H]^+$) 199.0, found 199.3.

HRMS (EI^+): *m/z* calcd. for $C_9H_{10}O_3S$ ($[M]^+$) 198.0351, found 198.0360.

Additional information on the chemical synthesis is available *via* Chemotion repository:
https://doi.org/10.14272/reaction/SA-FUHFF-UHFFFADPSC-FSMDHYUPDK-UHFFFADPSC-NUHFF-NSXYH-NUHFF-ZZZ

Additional information on the analysis of the target compound is available *via* Chemotion repository:
https://doi.org/10.14272/FSMDHYUPDKNFEI-BQYQJAHWSA-N.1

Ethyl (*E*)-2-phenylethene-1-sulfonate (9b)

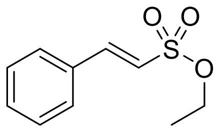

Following the **TP-5**, cinnamic acid (44.5 mg, 0.3 mmol), ethanol (52.5 µL, 3.0 equiv.), and SO_2 stock solution (10.0 equiv.) were used as substrates. The residue was purified by flash chromatography over silica gel (*n*-hexane/EtOAc = 95:5) to obtain **9b** as a colorless liquid (38.3 mg, 60% yield).

R_f = 0.25 (*n*-hexane/EtOAc = 9:1).

^{1}H NMR (400 MHz, $CDCl_3$): δ 7.61 (d, J = 15.6 Hz, 1H), 7.54–7.49 (m, 2H), 7.49–7.40 (m, 3H), 6.75 (d, J = 15.6 Hz, 1H), 4.24 (q, J = 7.2 Hz, 2H), 1.40 (t, J = 7.2 Hz, 3H).

^{13}C{^{1}H}-NMR (101 MHz, $CDCl_3$): δ 144.8, 132.1, 131.6, 129.3, 128.6, 121.3, 67.0, 15.0.

IR (ATR) $\tilde{\nu}$ (cm^{-1}): 3003, 1620, 1358, 1221, 1005, 752.

MS (APCI): *m/z* calcd. for $C_{10}H_{13}O_3S$ ($[M+H]^+$) 213.1, found 213.4.

HRMS (EI^+): *m/z* calcd. for $C_{10}H_{12}O_3S$ ($[M]^+$) 212.0507, found 212.0519.

Additional information on the chemical synthesis is available *via* Chemotion repository:
https://doi.org/10.14272/reaction/SA-FUHFF-UHFFFADPSC-LEDCJBCTEY-UHFFFADPSC-NUHFF-NSEGZ-NUHFF-ZZZ

Additional information on the analysis of the target compound is available *via* Chemotion repository:
https://doi.org/10.14272/LEDCJBCTEYLBJO-CMDGGOBGSA-N.1

Isopropyl (*E*)-2-phenylethene-1-sulfonate (9c)

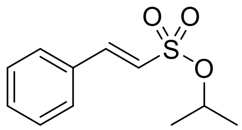

Following the **TP-5**, cinnamic acid (44.5 mg, 0.3 mmol), 2-propanol (68.8 µL, 3.0 equiv.), and SO_2 stock solution (10.0 equiv.) were used as substrates. The residue was purified by flash chromatography over silica gel (*n*-hexane/EtOAc = 95:5) to obtain **9c** as a colorless liquid (37.0 mg, 55% yield).

R_f = 0.35 (*n*-hexane/EtOAc = 9:1).

^{1}H NMR (400 MHz, $CDCl_3$): δ 7.60 (d, *J* = 15.6 Hz, 1H), 7.54–7.48 (m, 2H), 7.48–7.40 (m, 3H), 6.77 (d, *J* = 15.6 Hz, 1H), 4.84 (septet, *J* = 6.2 Hz, 1H), 1.41 (d, *J* = 6.2 Hz, 6H).

^{13}C{^{1}H}-NMR (101 MHz, $CDCl_3$): δ 143.8, 132.2, 131.4, 129.3, 128.5, 122.5, 77.4, 23.1.

IR (ATR) $\tilde{v}$ (cm^{-1}): 3056, 2986, 1618, 1356, 1168, 978, 750.

MS (APCI): *m/z* calcd. for $C_{11}H_{15}O_3S$ ($[M+H]^+$) 227.1, found 227.3.

HRMS (EI^+): *m/z* calcd. for $C_{11}H_{14}O_3S$ ($[M]^+$) 226.0664, found 226.0675.

Additional information on the chemical synthesis is available *via* Chemotion repository:
https://doi.org/10.14272/reaction/SA-FUHFF-UHFFFADPSC-OPTQKNRBAM-UHFFFADPSC-NUHFF-NSEGZ-NUHFF-ZZZ
Additional information on the analysis of the target compound is available *via* Chemotion repository:
https://doi.org/10.14272/OPTQKNRBAMBSSR-CMDGGOBGSA-N.1

Isobutyl (*E*)-2-phenylethene-1-sulfonate (9d)

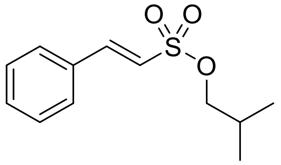

Following the **TP-5**, cinnamic acid (44.5 mg, 0.3 mmol), isobutanol (83.2 µL, 3.0 equiv.), and SO_2 stock solution (10.0 equiv.) were used as substrates. The residue was purified by flash chromatography over silica gel (*n*-hexane/EtOAc = 95:5) to obtain **9d** as a colorless liquid (43.6 mg, 60% yield).

R_f = 0.40 (*n*-hexane/EtOAc = 9:1).

^{1}H NMR (400 MHz, $CDCl_3$): δ 7.60 (d, *J* = 15.6 Hz, 1H), 7.54–7.49 (m, 2H), 7.49–7.41 (m, 3H), 6.75 (d, *J* = 15.6 Hz, 1H), 3.92 (d, *J* = 6.6 Hz, 2H), 2.04 (nonet, *J* = 6.6 Hz, 1H), 0.98 (d, *J* = 6.6 Hz, 6H).

^{13}C{^{1}H}-NMR (101 MHz, $CDCl_3$): δ 144.7, 132.1, 131.5, 129.3, 128.6, 121.2, 76.5, 28.2, 18.8.

IR (ATR) $\tilde{v}$ (cm^{-1}): 3005, 2965, 1621, 1358, 1168, 976, 749.

MS (APCI): *m/z* calcd. for $C_{12}H_{17}O_3S$ ($[M+H]^+$) 241.1, found 241.4.

HRMS (EI^+): *m/z* calcd. for $C_{12}H_{16}O_3S$ ($[M]^+$) 240.0820, found 240.0826.

Additional information on the chemical synthesis is available *via* Chemotion repository:
https://doi.org/10.14272/reaction/SA-FUHFF-UHFFFADPSC-KIKXBEOFOK-UHFFFADPSC-NUHFF-NSEGZ-NUHFF-ZZZ

Additional information on the analysis of the target compound is available *via* Chemotion repository:
https://doi.org/10.14272/KIKXBEOFOKXQCA-CMDGGOBGSA-N.1

Benzyl (*E*)-2-phenylethene-1-sulfonate (9e)

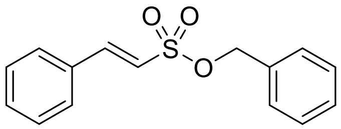

Following the **TP-5**, cinnamic acid (44.5 mg, 0.3 mmol), benzyl alcohol (93.6 µL, 3.0 equiv.), and SO_2 stock solution (10.0 equiv.) were used as substrates. The residue was purified by flash chromatography over silica gel (*n*-hexane/EtOAc = 95:5) to obtain **9e** as a colorless liquid (42.3 mg, 51% yield).

R_f = 0.20 (*n*-hexane/EtOAc = 9:1).

^{1}H NMR (400 MHz, $CDCl_3$): δ 7.59 (d, *J* = 15.6 Hz, 1H), 7.50–7.31 (m, 10H), 6.64 (d, *J* = 15.6 Hz, 1H), 5.19 (s, 2H).

^{13}C{^{1}H}-NMR (101 MHz, $CDCl_3$): δ 144.8, 133.5, 132.1, 131.6, 129.4, 129.3, 128.94, 128.92, 128.6, 121.7, 72.2.

IR (ATR) $\tilde{\nu}$ (cm^{-1}): 3026, 1624, 1169, 968, 745.

MS (APCI): *m/z* calcd. for $C_{15}H_{13}O_3S$ ($[M+H]^+$) 275.1, found 275.4.

HRMS (EI^+): *m/z* calcd. for $C_{15}H_{14}O_3S$ ($[M]^+$) 274.0664, found 274.0658.

Additional information on the chemical synthesis is available *via* Chemotion repository:
https://doi.org/10.14272/reaction/SA-FUHFF-UHFFFADPSC-NAJYROHLEL-UHFFFADPSC-NUHFF-NZJBK-NUHFF-ZZZ

Additional information on the analysis of the target compound is available *via* Chemotion repository:
https://doi.org/10.14272/NAJYROHLELDYQG-VAWYXSNFSA-N.1

Cyclohexyl (*E*)-2-phenylethene-1-sulfonate (9f)

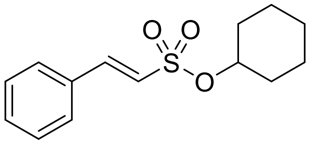

Following the **TP-5**, cinnamic acid (44.5 mg, 0.3 mmol), cyclohexanol (90.1 mg, 3.0 equiv.), and SO_2 stock solution (10.0 equiv.) were used as substrates. The residue was purified by flash chromatography over silica gel (*n*-hexane/EtOAc = 95:5) to obtain **9f** as a colorless liquid (31.4 mg, 39% yield).

R_f = 0.35 (*n*-hexane/EtOAc = 9:1).

^{1}H NMR (400 MHz, $CDCl_3$): δ 7.59 (d, *J* = 15.6 Hz, 1H), 7.54–7.48 (m, 2H), 7.48–7.40 (m, 3H), 6.77 (d, *J* = 15.6 Hz, 1H), 4.65–4.55 (m, 1H), 2.04–1.91 (m, 2H), 1.83–1.72 (m, 2H), 1.72–1.59 (m, 2H), 1.57–1.46 (m, 1H), 1.42–1.22 (m, 3H).

^{13}C{^{1}H}-NMR (101 MHz, $CDCl_3$): δ 143.6, 132.3, 131.4, 129.3, 128.5, 122.8, 81.9, 32.7, 25.0, 23.6.

IR (ATR) $\tilde{\nu}$ (cm^{-1}): 3050, 2936, 1618, 1351, 1162, 926, 746.

MS (APCI): *m/z* calcd. for $C_{14}H_{19}O_3S$ ($[M+H]^+$) 267.1, found 267.4.

HRMS (EI^+): *m/z* calcd. for $C_{14}H_{18}O_3S$ ($[M]^+$) 266.0977, found 266.0992.

Additional information on the chemical synthesis is available *via* Chemotion repository:
https://doi.org/10.14272/reaction/SA-FUHFF-UHFFFADPSC-NABCLFIQWW-UHFFFADPSC-NUHFF-NZJBK-NUHFF-ZZZ

Additional information on the analysis of the target compound is available *via* Chemotion repository:
https://doi.org/10.14272/NABCLFIQWWOVSR-VAWYXSNFSA-N.1

Tetrahydro-2*H*-pyran-4-yl (*E*)-2-phenylethene-1-sulfonate (9g)

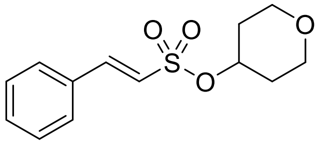

Following the **TP-5**, cinnamic acid (44.5 mg, 0.3 mmol), tetrahydro-2*H*-pyran-4-ol (85.8 µL, 3.0 equiv.), and SO_2 stock solution (10.0 equiv.) were used as substrates. The residue was purified by flash chromatography over silica gel (*n*-hexane/EtOAc = 93:7) to obtain **9g** as a colorless liquid (31.9 mg, 40% yield).

R_f = 0.10 (*n*-hexane/EtOAc = 9:1).

^{1}H NMR (400 MHz, $CDCl_3$): δ 7.62 (d, *J* = 15.6 Hz, 1H), 7.54–7.49 (m, 2H), 7.49–7.41 (m, 3H), 6.79 (d, *J* = 15.6 Hz, 1H), 4.83–4.75 (m, 1H), 4.01–3.89 (m, 2H), 3.58–3.50 (m, 2H), 2.08–1.99 (m, 2H), 1.94–1.83 (m, 2H).

^{13}C{^{1}H}-NMR (101 MHz, $CDCl_3$): δ 144.3, 132.0, 131.6, 129.3, 128.6, 122.3, 77.5, 64.9, 32.8.

IR (ATR) $\tilde{v}$ (cm^{-1}): 3003, 2963, 1622, 1358, 1221, 949, 750.

MS (APCI): *m/z* calcd. for $C_{13}H_{17}O_4S$ ($[M+H]^+$) 269.1, found 269.4.

HRMS (EI^+): *m/z* calcd. for $C_{13}H_{16}O_4S$ ($[M]^+$) 268.0769, found 268.0763.

Additional information on the chemical synthesis is available *via* Chemotion repository:
https://doi.org/10.14272/reaction/SA-FUHFF-UHFFFADPSC-XMTQMZHHYL-UHFFFADPSC-NUHFF-NFMKG-NUHFF-ZZZ

Additional information on the analysis of the target compound is available *via* Chemotion repository:
https://doi.org/10.14272/XMTQMZHHYLURMF-DHZHZOJOSA-N.1

(3a*R*,5*R*,6*S*,6a*R*)-5-((*R*)-2,2-dimethyl-1,3-dioxolan-4-yl)-2,2-dimethyltetrahydrofuro[2,3-*d*][1,3]dioxol-6-yl (*E*)-2-phenylethene-1-sulfonate (9h)

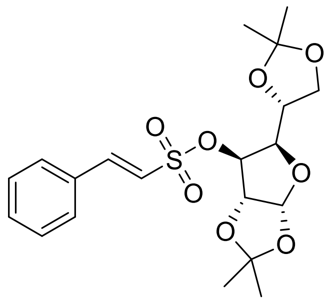

Following the **TP-5**, cinnamic acid (44.5 mg, 0.3 mmol), 1,2:5,6-di-*O*-isopropylidene-α-D-glucofuranose (234.3 mg, 3.0 equiv.), and SO_2 stock solution (10.0 equiv.) were used as substrates. The residue was twofold purified by flash chromatography over silica gel (*n*-hexane/EtOAc = 93:7 then CH_2Cl_2/EtOAc = 95:5) to obtain **9h** as a colorless liquid (21.6 mg, 17% yield).

R_f = 0.20 (*n*-hexane/EtOAc = 9:1).

^{1}H NMR (400 MHz, $CDCl_3$): δ 7.63 (d, *J* = 15.6 Hz, 1H), 7.55–7.50 (m, 2H), 7.50–7.41 (m, 3H), 6.82 (d, *J* = 15.6 Hz, 1H), 5.96 (d, *J* = 3.6 Hz, 1H), 4.87 (d, *J* = 3.6 Hz, 1H), 4.80 (d, *J* = 3.0 Hz, 1H), 4.31–4.22 (m, 1H), 4.13–4.06 (m, 2H), 4.01 (dd, *J* = 9.0, 4.0 Hz, 1H), 1.50 (s, 3H), 1.33 (s, 3H), 1.23 (s, 3H), 1.17 (s, 3H).

^{13}C{^{1}H}-NMR (101 MHz, $CDCl_3$): δ 145.2, 132.1, 131.8, 129.3, 128.6, 121.2, 112.7, 109.6, 105.3, 83.8, 82.3, 79.8, 72.2, 67.5, 27.0, 26.7, 26.3, 25.2.

IR (ATR) $\tilde{\nu}$ (cm^{-1}): 3003, 2922, 1358, 1219, 1171, 981, 750.

MS (APCI): *m/z* calcd. for $C_{20}H_{27}O_8S$ ($[M+H]^+$) 427.1, found 427.6.

HRMS (EI^+): *m/z* calcd. for $C_{20}H_{26}O_8S$ ($[M]^+$) 426.1348, found 426.1371.

Additional information on the chemical synthesis is available *via* Chemotion repository:
https://doi.org/10.14272/reaction/SA-FUHFF-UHFFFADPSC-FOZZOCLQRR-UHFFFADPSC-NUHFF-NBFOL-NUHFF-ZZZ

Additional information on the analysis of the target compound is available *via* Chemotion repository:
https://doi.org/10.14272/FOZZOCLQRRJRQC-PPRZOJQRSA-N.1

Methyl (*E*)-3-((styrylsulfonyl)oxy)butanoate (9i)

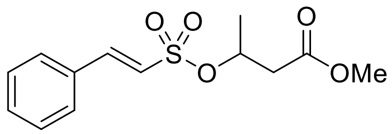

Following the **TP-5**, cinnamic acid (44.5 mg, 0.3 mmol), methyl 3-hydroxybutanoate (101.0 µL, 3.0 equiv.), and SO_2 stock solution (10.0 equiv.) were used as substrates. The residue was purified by flash chromatography over silica gel (*n*-hexane/EtOAc = 94:6) to obtain **9i** as a colorless liquid (13.2 mg, 15% yield).

R_f = 0.13 (*n*-hexane/EtOAc = 9:1).

^{1}H NMR (400 MHz, $CDCl_3$): δ 7.60 (d, J = 15.6 Hz, 1H), 7.56–7.51 (m, 2H), 7.49–7.40 (m, 3H), 6.80 (d, J = 15.6 Hz, 1H), 5.06 (sextet, J = 6.4 Hz, 1H), 3.65 (s, 3H), 2.88–2.79 (m, 1H), 2.63–2.55 (m, 1H), 1.50 (d, J = 6.4 Hz, 3H).

^{13}C{^{1}H}-NMR (101 MHz, $CDCl_3$): δ 170.1, 144.3, 132.3, 131.5, 129.3, 128.6, 122.1, 76.0, 52.1, 41.4, 21.5.

IR (ATR) $\tilde{\nu}$ (cm^{-1}): 3003, 2962, 1710, 1620, 1358, 1221, 1168, 979, 750.

MS (APCI): *m/z* calcd. for $C_{13}H_{17}O_5S$ ($[M+H]^+$) 285.1, found 285.4.

HRMS (EI^+): *m/z* calcd. for $C_{13}H_{16}O_5S$ ($[M]^+$) 284.0718, found 284.0727.

Additional information on the chemical synthesis is available *via* Chemotion repository: https://doi.org/10.14272/reaction/SA-FUHFF-UHFFFADPSC-PFQIXBDZPI-UHFFFADPSC-NUHFF-NSEGZ-NUHFF-ZZZ

Additional information on the analysis of the target compound is available *via* Chemotion repository:

https://doi.org/10.14272/PFQIXBDZPIDCQY-CMDGGOBGSA-N.1

(1*R*,2*S*,5*R*)-2-Isopropyl-5-methylcyclohexyl (*E*)-2-phenylethene-1-sulfonate (9j)

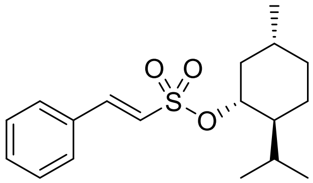

Following the **TP-5**, cinnamic acid (44.5 mg, 0.3 mmol), (-)-menthol (140.6 mg, 3.0 equiv.), and SO_2 stock solution (10.0 equiv.) were used as substrates. The residue was purified by flash chromatography over silica gel (*n*-hexane/EtOAc = 98:2) to obtain **9j** as a colorless solid (42.9 mg, 44% yield).

mp: 74.5–75.3 °C. R_f = 0.38 (*n*-hexane/EtOAc = 9:1).

^{1}H NMR (400 MHz, $CDCl_3$): δ 7.59 (d, *J* = 15.6 Hz, 1H), 7.53–7.47 (m, 2H), 7.47–7.40 (m, 3H), 6.77 (d, *J* = 15.6 Hz, 1H), 4.45 (td, *J* = 10.8, 4.6 Hz, 1H), 2.34–2.26 (m, 1H), 2.11 (septetd, *J* = 7.0, 2.2 Hz, 1H), 1.75–1.63 (m, 2H), 1.53–1.38 (m, 2H), 1.33–1.23 (m, 1H), 1.09–0.96 (m, 1H), 0.93 (d, *J* = 3.4 Hz, 3H), 0.91 (d, *J* = 4.0 Hz, 3H), 0.90–0.81 (m, 1H), 0.76 (d, *J* = 6.8 Hz, 3H).

^{13}C{^{1}H}-NMR (101 MHz, $CDCl_3$): δ 143.4, 132.3, 131.4, 129.3, 128.5, 123.0, 84.0, 47.6, 42.4, 33.9, 31.8, 25.7, 23.1, 22.0, 21.0, 15.8.

IR (ATR) $\tilde{\nu}$ (cm^{-1}): 3029, 2956, 1620, 1345, 1161, 981, 749.

MS (APCI): *m/z* calcd. for $C_{18}H_{27}O_3S$ ($[M+H]^+$) 323.2, found 323.5.

HRMS (EI^+): *m/z* calcd. for $C_{18}H_{26}O_3S$ ($[M]^+$) 322.1603, found 322.1618.

Additional information on the chemical synthesis is available *via* Chemotion repository:
https://doi.org/10.14272/reaction/SA-FUHFF-UHFFFADPSC-FKWGKMBPKN-UHFFFADPSC-NUHFF-NSOOW-NUHFF-ZZZ

Additional information on the analysis of the target compound is available *via* Chemotion repository:
https://doi.org/10.14272/FKWGKMBPKNXHHI-XVOPWDCPSA-N.1

2,2,2-Trifluoroethyl (*E*)-2-phenylethene-1-sulfonate (9k)

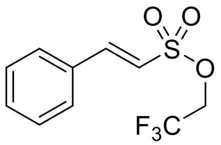

Following the **TP-5**, cinnamic acid (44.5 mg, 0.3 mmol), 2,2,2-trifluoroethanol (64.8 µL, 3.0 equiv.), and SO_2 stock solution (10.0 equiv.) were used as substrates. The residue was purified by flash chromatography over silica gel (*n*-hexane/EtOAc = 98:2) to obtain **9k** as a colorless solid (44.4 mg, 56% yield).

mp: 93.8–94.0 °C. $R_f = 0.35$ (*n*-hexane/EtOAc = 9:1).

^{1}H NMR (400 MHz, $CDCl_3$): δ 7.68 (d, J = 15.6 Hz, 1H), 7.56–7.51 (m, 2H), 7.51–7.42 (m, 3H), 6.78 (d, J = 15.6 Hz, 1H), 4.46 (q, J = 8.0 Hz, 2H).

^{13}C{^{1}H}-NMR (101 MHz, $CDCl_3$): δ 146.8, 132.2, 131.5, 129.5, 128.9, 122.1 (q, $^1J_{C\text{-}F}$ = 277.8 Hz, 1C), 120.0, 64.7 (q, $^2J_{C\text{-}F}$ = 38.0 Hz, 1C).

^{19}F{^{1}H}-NMR (376 MHz, $CDCl_3$): δ -73.8.

IR (ATR) $\tilde{\nu}$ (cm^{-1}): 3073, 2973, 1615, 1358, 1288, 1159, 961, 745.

MS (APCI): *m/z* calcd. for $C_{10}H_{10}O_3F_3S$ ($[M+H]^+$) 267.0, found 267.3.

HRMS (EI^+): *m/z* calcd. for $C_{10}H_9O_3F_3S$ ($[M]^+$) 266.0225, found 266.0244.

Additional information on the chemical synthesis is available *via* Chemotion repository:
https://doi.org/10.14272/reaction/SA-FUHFF-UHFFFADPSC-WTISUMKEBZ-UHFFFADPSC-NUHFF-NZMGP-NUHFF-ZZZ

Additional information on the analysis of the target compound is available *via* Chemotion repository:
https://doi.org/10.14272/WTISUMKEBZKMEP-VOTSOKGWSA-N.1

1,1,1,3,3,3-Hexafluoropropan-2-yl (*E*)-2-phenylethene-1-sulfonate (9l)

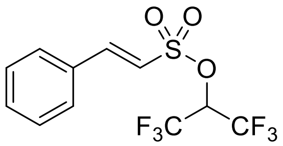

Following the **TP-5**, cinnamic acid (44.5 mg, 0.3 mmol), 1,1,1,3,3,3-hexafluoroisopropanol (94.5 µL, 3.0 equiv.), and SO_2 stock solution (10.0 equiv.) were used as substrates. The residue was purified by flash chromatography over silica gel (*n*-hexane/EtOAc = 98:2) to obtain **9l** as a colorless solid (24.8 mg, 25% yield).

mp: 49.6–49.8 °C. R_f = 0.45 (*n*-hexane/EtOAc = 9:1).

^{1}H NMR (400 MHz, $CDCl_3$): δ 7.74 (d, J = 15.4 Hz, 1H), 7.57–7.50 (m, 3H), 7.50–7.44 (m, 2H), 6.80 (d, J = 15.4 Hz, 1H), 5.24 (septet, J = 5.6 Hz, 1H).

^{13}C{^{1}H}-NMR (101 MHz, $CDCl_3$): δ 147.7, 132.6, 131.3, 129.5, 129.1, 120.1 (qq, $^1J_{C\text{-}F}$ = 282.8 Hz, $^3J_{C\text{-}F}$ = 2.6 Hz, 2C), 119.9, 72.3 (septet, $^2J_{C\text{-}F}$ = 35.4 Hz, 1C).

^{19}F{^{1}H}-NMR (376 MHz, $CDCl_3$): δ -73.1.

IR (ATR) $\tilde{\nu}$ (cm^{-1}): 3073, 2973, 1614, 1361, 1292, 1171, 962, 746.

MS (APCI): *m/z* calcd. for $C_{11}H_7O_3F_6S$ ($[M+H]^+$) 335.0, found 335.5.

HRMS (EI^+): *m/z* calcd. for $C_{11}H_8O_3F_6S$ ($[M]^+$) 334.0098, found 334.0103.

Additional information on the chemical synthesis is available *via* Chemotion repository:
https://doi.org/10.14272/reaction/SA-FUHFF-UHFFFADPSC-ZNKPMXBNDG-UHFFFADPSC-NUHFF-NZMGP-NUHFF-ZZZ

Additional information on the analysis of the target compound is available *via* Chemotion repository:
https://doi.org/10.14272/ZNKPMXBNDGNEFX-VOTSOKGWSA-N.1

7 Unsuccessful Substrates

Cinnamic acids and related substrates:

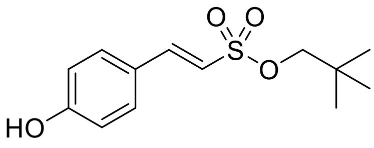

oxidation of phenol

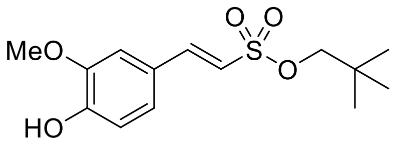

oxidation of phenol

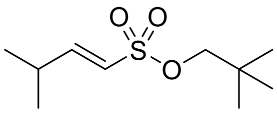

no alkenesulfonate product detected

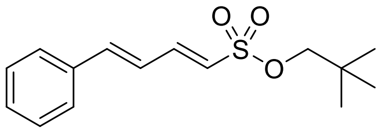

no alkenesulfonate product detected

Alcohols and phenol:

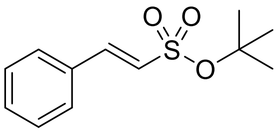

too sterically demanding

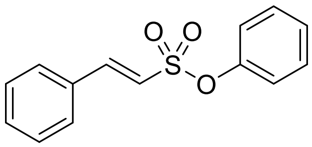

oxidation of phenol

Figure S7: Unsuccessful derivatives under the standard conditions.

8 Control Experiments

The control experiments were performed to elucidate the reaction mechanism. As shown in **Table S3**, no product formation was observed in the absence of a base or when the electric current was omitted (entries 2 and 3). No product was detected in the crude reaction mixture when the radical scavengers, such as 2,6-di-*tert*-butyl-4-methylphenol (BHT) or 2,2,6,6-tetramethylpiperidinyl-oxyl (TEMPO) were added. In both cases, only partial decomposition of cinnamic acid **6a** was observed. In the presence of BHT, the addition of an alkoxy sulfonyl radical species, could detected by GC-MS (**Figure S8**).

Table S3: Control experiments.

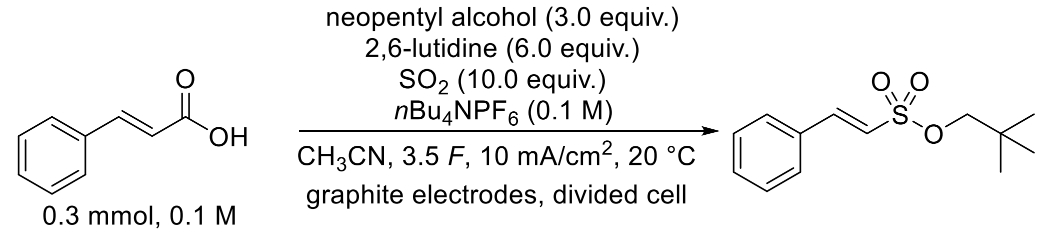

entry	deviation from the standard conditions	yield (%)[a]	cinnamic acid (%)[a]
**1**	**none**	**71 (70)[b]**	**0**
2	no electric current	0	65
3	no 2,6-lutidine	0	trace
4	+BHT (3.0 equiv.)	0	64
5	+TEMPO (3.0 equiv.)	0	trace

[a] ^{1}H-NMR yield with the use of $CHPh_3$ as the internal standard. [b] Isolated yield.

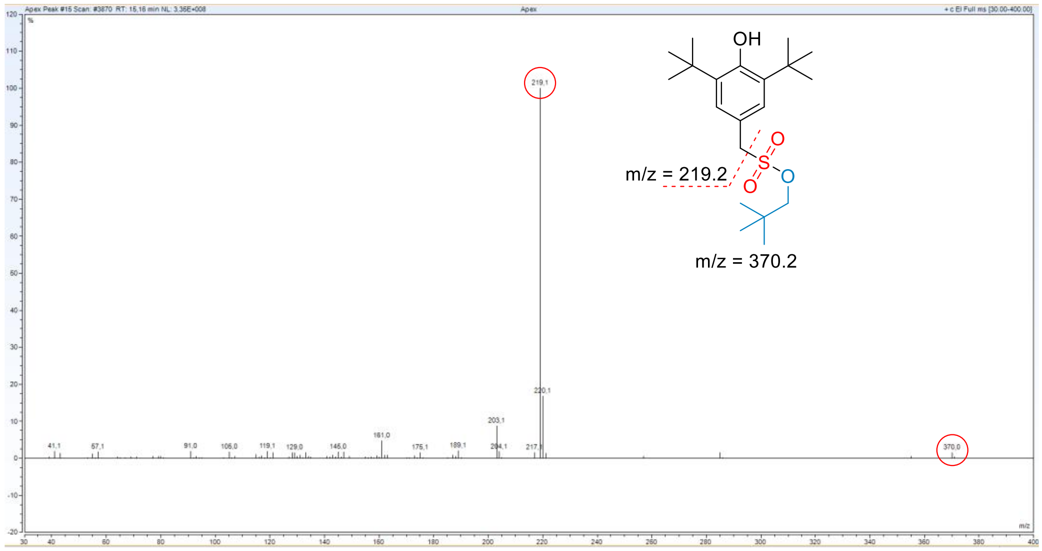

Figure S8: Mass spectrum obtained by GC/MS of a BHT-trapped intermediate.

9 Cyclic Voltammetry Results

The following measurements were used for the postulation of the reaction mechanism: the oxidation of the cinnamate **II** should take place before the oxidation of the monoalkylsulfite **I** (**Figure S9**, Measurement conditions: 0.1 M *n*Bu_4NPF_6 in MeCN; *c*(cinnamic acid or neopentyl alcohol) = 0.01 M; *c*(SO_2) = 0.05 M; *c*(2,6-lutidine) = 0.011 M; v = 100 mV/s.).

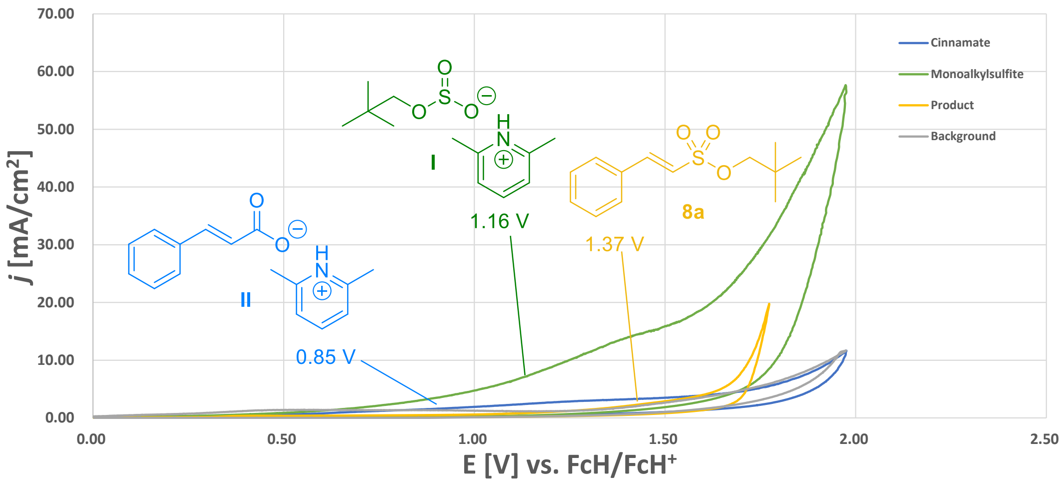

Figure S9: Cyclic voltammograms of cinnamate **II** (blue), monoalkylsulfite **I** (green), alkenesulfonate **8a** (yellow), and blank measurement (0.1 M *n*Bu_4NPF_6 in MeCN; grey).

In the case of the cinnamate **II**, the measured current decreases slightly with the number of scans (**Figure S10**, Measurement conditions: 0.1 M *n*Bu_4NPF_6 in MeCN; *c*(cinnamic acid) = 0.01 M; *c*(2,6-lutidine) = 0.011 M; v = 100 mV/s.).

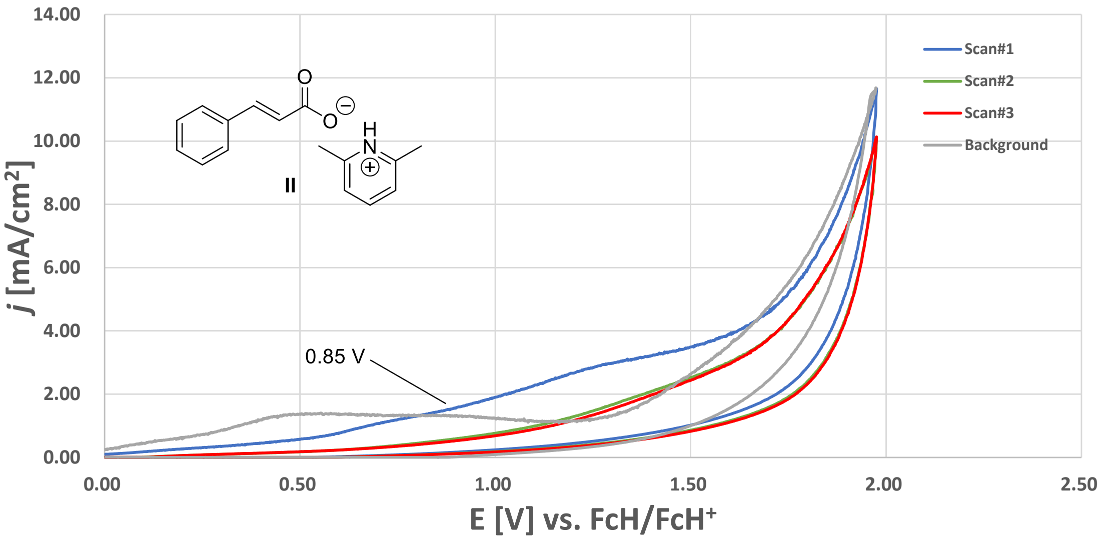

Figure S10: Cyclic voltammograms of cinnamate **II**.

There is no obvious current change with the number of scans when measuring the product **8a**. Thus, overoxidation of the product should be negligible. (**Figure S11**, Measurement conditions: 0.1 M *n*Bu_4NPF_6 in MeCN; *c*(**8a**) = 0.01 M; v = 100 mV/s.).

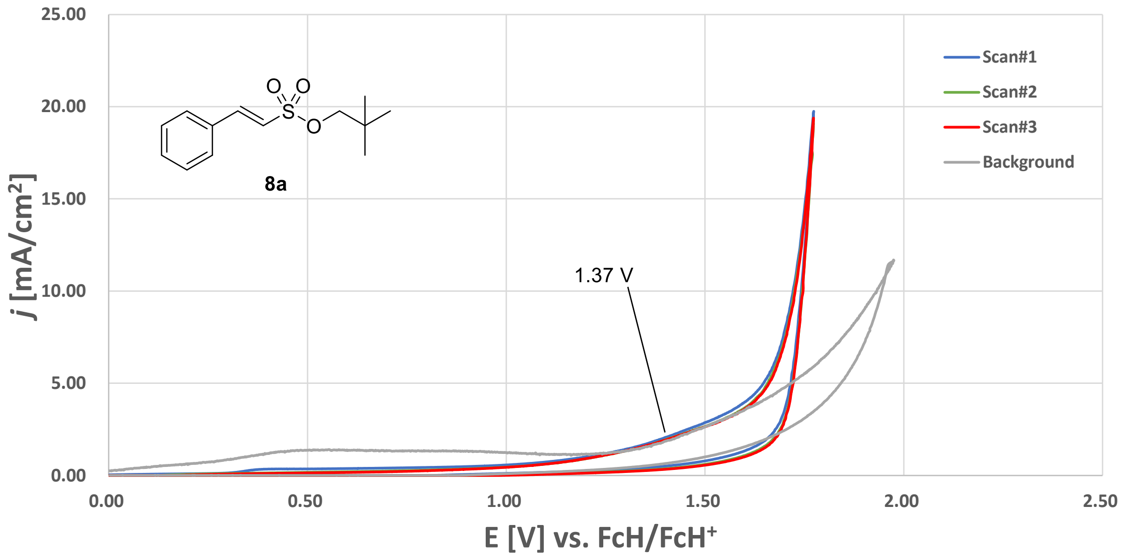

Figure S11: Cyclic voltammograms of neopentyl (*E*)-2-phenylethene-1-sulfonate **8a**.

10 References

[42] A. de A. Bartolomeu, F. A. Breitschaft, D. Schollmeyer, R. A. Pilli, S. R. Waldvogel, "Electrochemical Multicomponent Synthesis of Alkyl Alkenesulfonates using Styrenes, SO_2 and Alcohols." *Chem. Eur. J.* **2024**, *30*, e202400557.

[76] S. Leong, S. Faudzi, F. Abas, M. Aluwi, K. Rullah, L. Wai, N. Lajis, "Synthesis and Sar Study of Diarylpentanoid Analogues as New Anti-Inflammatory Agents." *Molecules* **2014**, *19*, 16058.

[77] S. Maki, A. Kojima, H. Tanba, "Preparation of 4,5-dihydroazole-4-carboxylic acid derivatives as luminescent substrates for luciferase." JP2009184932 A, **2009**.

[78] J. B. Ferguson, "THE IODOMETRIC DETERMINATION OF SULFUR DIOXIDE AND THE SULFITES." *J. Am. Chem. Soc.* **1917**, *39*, 364.

[79] V. Thakur, S. Kumar, P. Das, "Polystyrene Supported Palladium Nanoparticles Catalyzed Cinnamic Acid Synthesis using Maleic Anhydride as Substitute of Acrylic Acid." *Catal. Sci. Technol.* **2017**, *7*, 3692.

[80] F. Krätzschmar, M. Kaßel, D. Delony, A. Breder, "Selenium-Catalyzed C(sp^3)–H Acyloxylation: Application in the Expedient Synthesis of Isobenzofuranones." *Chem. Eur. J.* **2015**, *21*, 7030.

[81] L. Jarrige, F. Blanchard, G. Masson, "Enantioselective Organocatalytic Intramolecular Aza-Diels–Alder Reaction." *Angew. Chem. Int. Ed.* **2017**, *56*, 10573.

11 X-ray crystallographic data for selected compounds

Table S4: Crystal data for **10o** (CCDC no. 2381294, the thermal ellipsoid drawn at 50% probability level.).

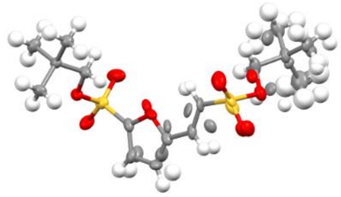

The purified compound **10o** was dissolved in a minimal amount of EtOAc in a glass vial. Hexanes were then added until the solution became turbid. The solution was allowed to slowly evaporate. After a few days, colorless crystals were obtained.

Empirical formula	$C_{16} H_{26} O_7 S_2$	
Formula weight	394.49	
Temperature	100(2) K	
Wavelength	1.54178 Å	
Crystal system	Orthorhombic	
Space group	$Pna2_1$	
Unit cell dimensions	a = 11.8362(6) Å	α= 90°.
	b = 5.6743(3) Å	β= 90°.
	c = 29.0528(14) Å	γ = 90°.
Volume	1951.25(17) $Å^3$	
Z	4	
Density (calculated)	1.343 Mg/m^3	
Absorption coefficient	2.772 mm^{-1}	
F(000)	840	
Crystal size	0.195 x 0.118 x 0.049 mm^3	
Theta range for data collection	6.093 to 70.060°.	
Index ranges	-12<=h<=14, -6<=k<=6, -35<=l<=35	
Reflections collected	25464	
Independent reflections	3640 [R(int) = 0.0683]	
Completeness to theta = 67.679°	99.9 %	
Absorption correction	Semi-empirical from equivalents	
Max. and min. transmission	1.000 and 0.769	
Refinement method	Full-matrix least-squares on F^2	
Data / restraints / parameters	3640 / 205 / 369	
Goodness-of-fit on F^2	1.098	
Final R indices [I>2sigma(I)]	R1 = 0.0288, wR2 = 0.0727	
R indices (all data)	R1 = 0.0334, wR2 = 0.0734	
Absolute structure parameter	0.18(2)	
Extinction coefficient	n/a	
Largest diff. peak and hole	0.303 and -0.246 e.$Å^{-3}$	

12 ^{1}H, ^{13}C NMR, and ^{19}F NMR spectra for compounds 6, 8, 9, and 10

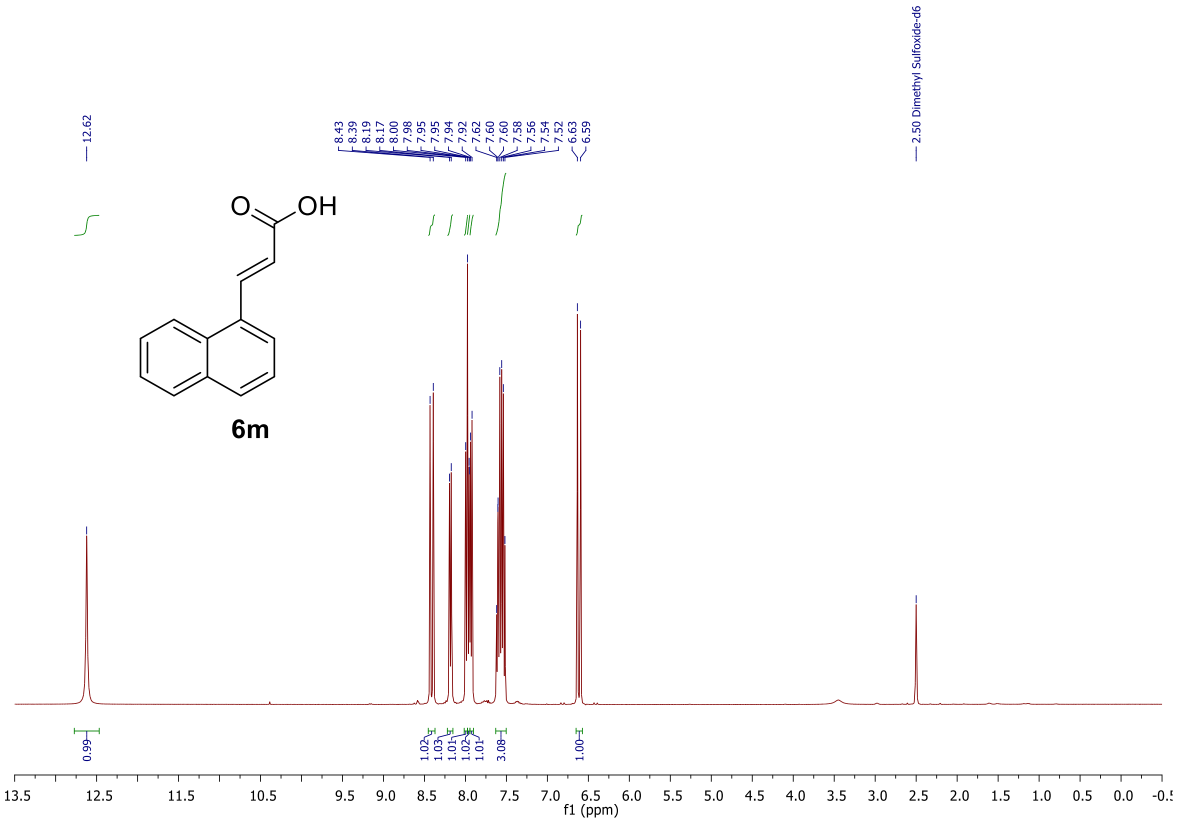

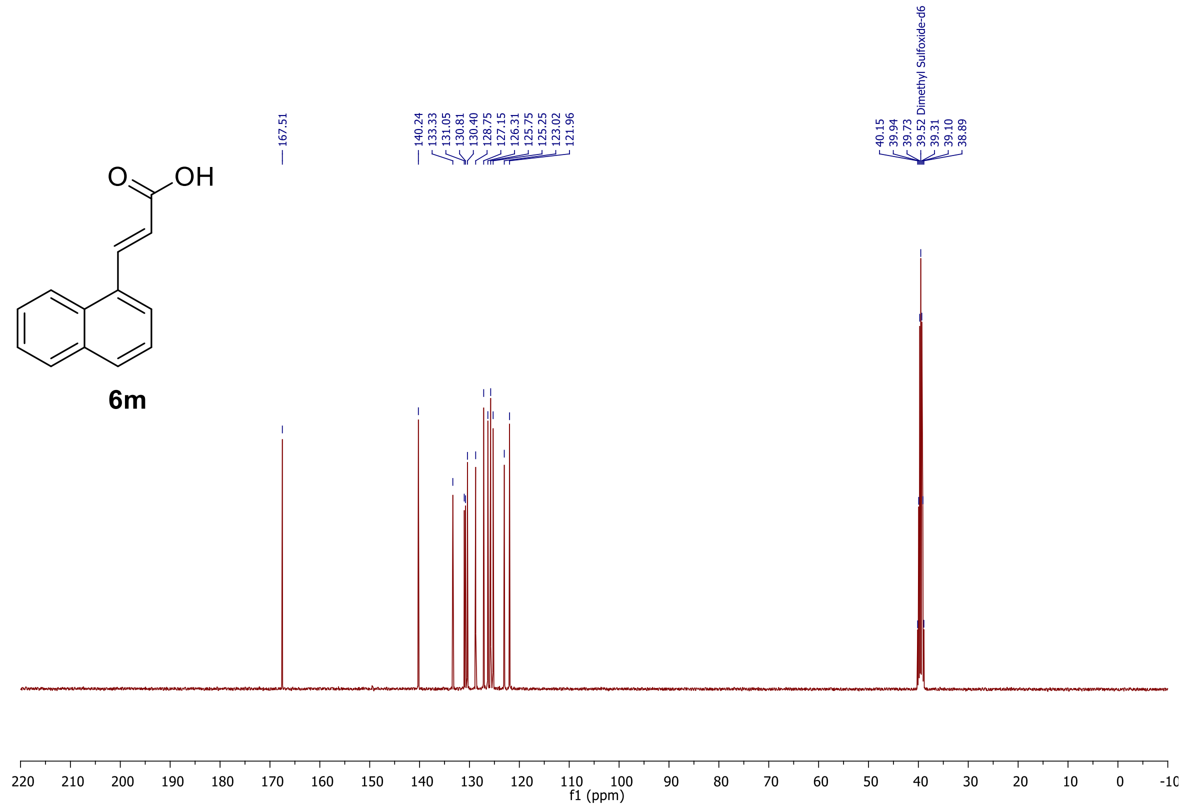

Figure S12: ^{1}H (DMSO-d_6, 400 MHz) and ^{13}C{^{1}H} (DMSO-d_6, 101 MHz) NMR Spectrum of **6m**.

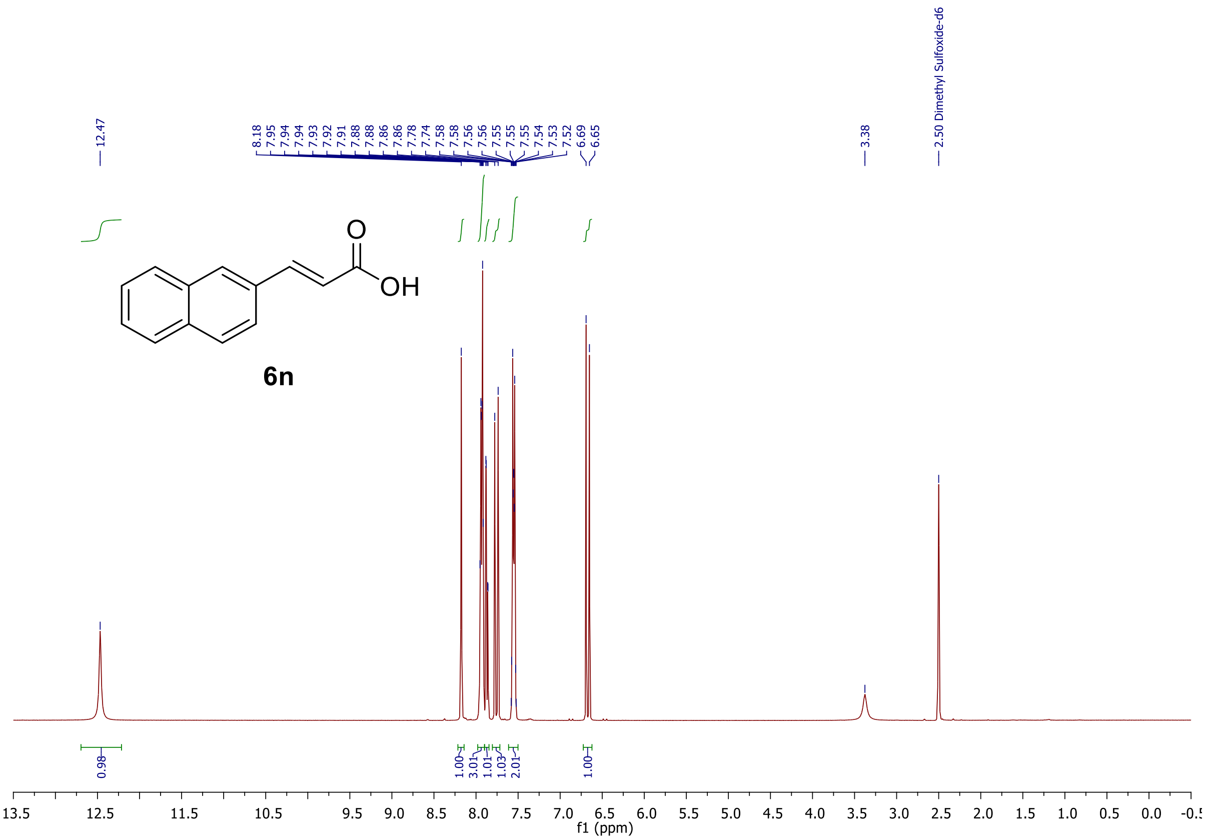

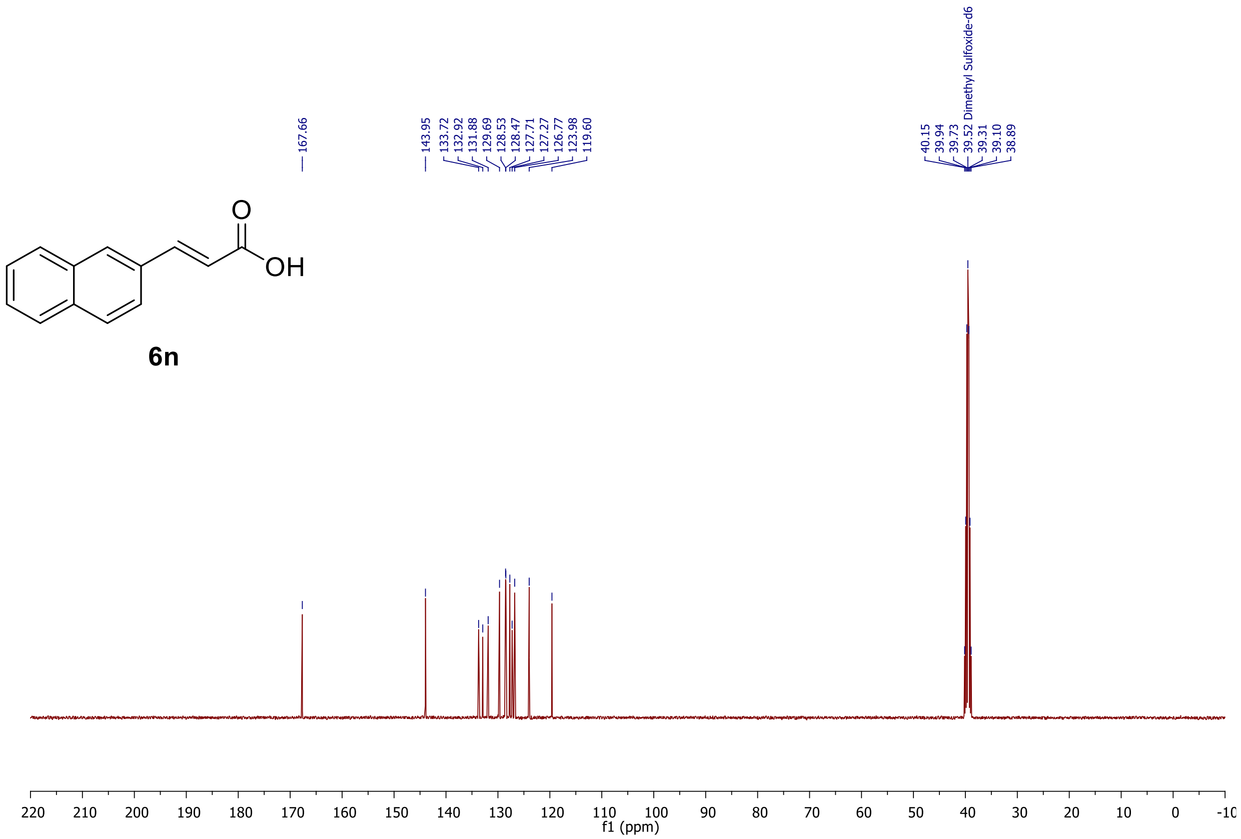

Figure S13: ^{1}H (DMSO-d_6, 400 MHz) and $^{13}C\{^{1}H\}$ (DMSO-d_6, 101 MHz) NMR Spectrum of **6n**.

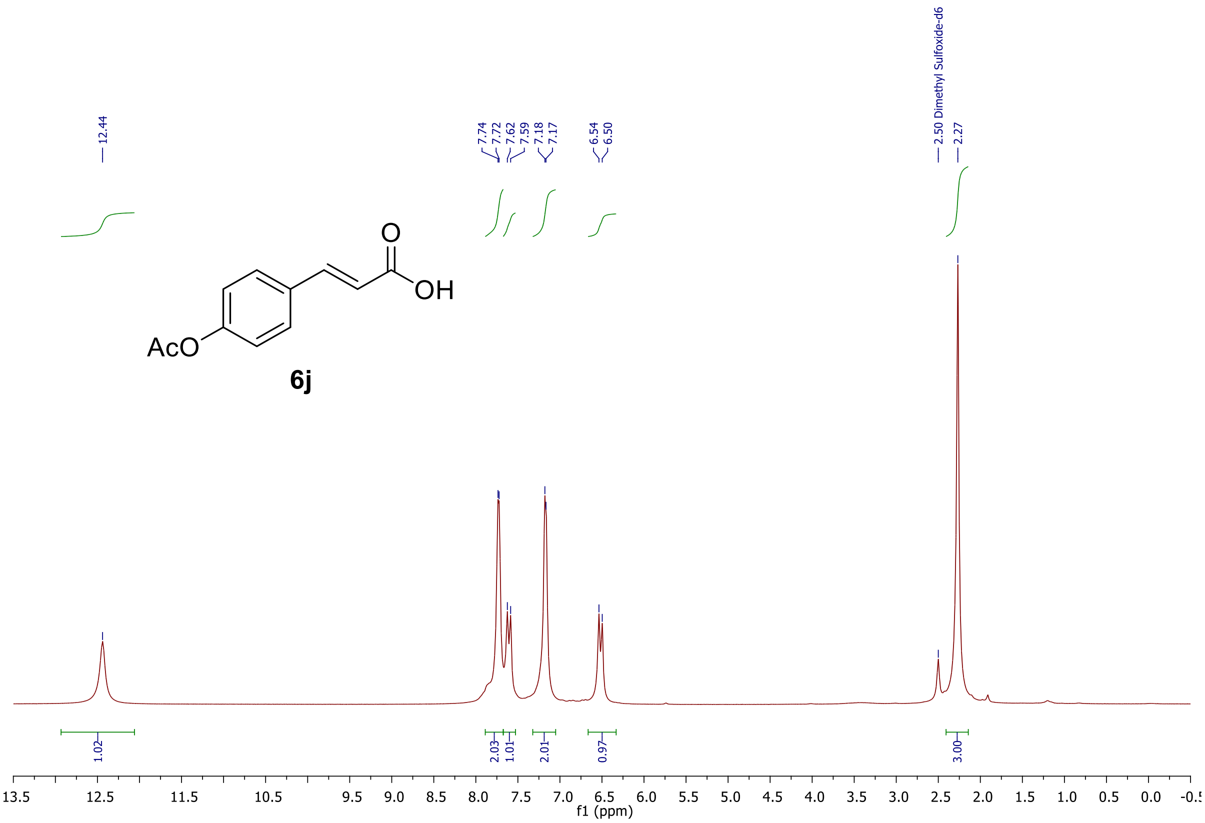

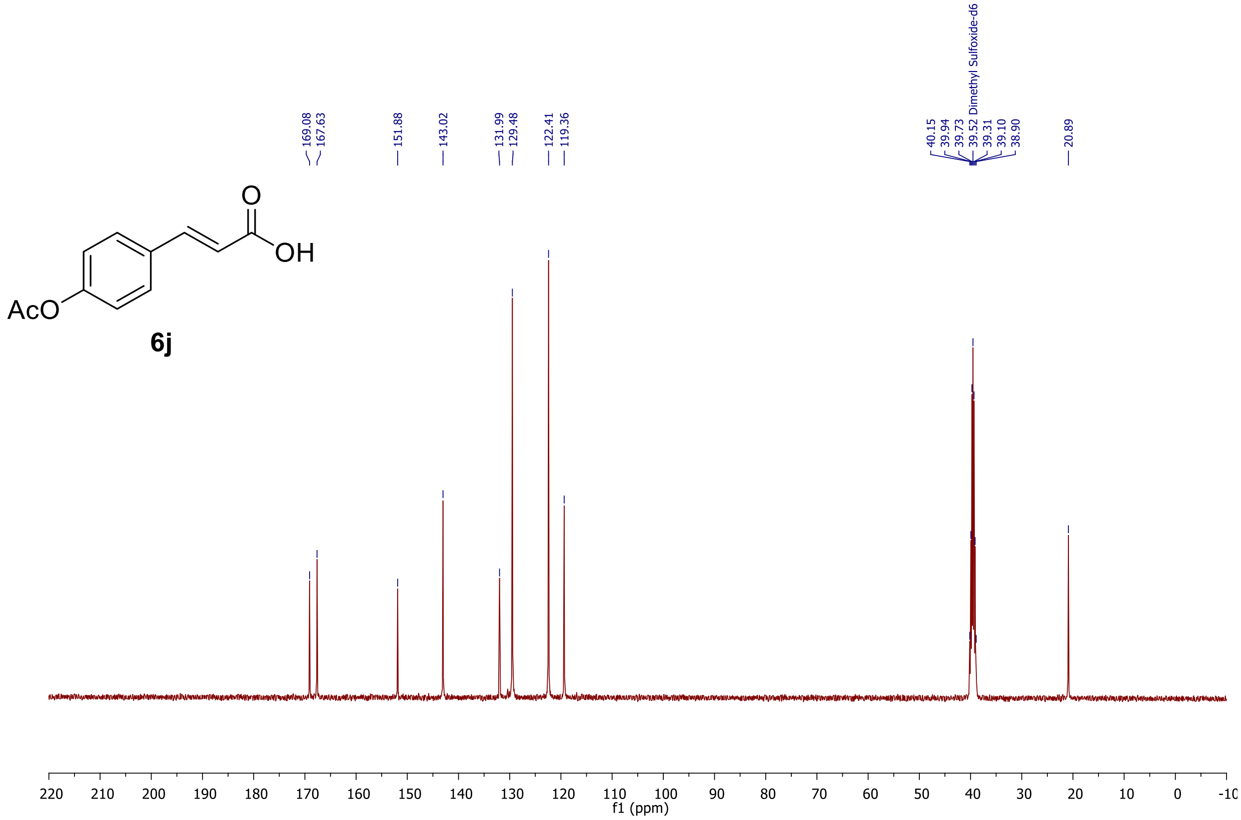

Figure S14: ^{1}H (DMSO-d_6, 400 MHz) and $^{13}C\{^{1}H\}$ (DMSO-d_6, 101 MHz) NMR Spectrum of **6j**.

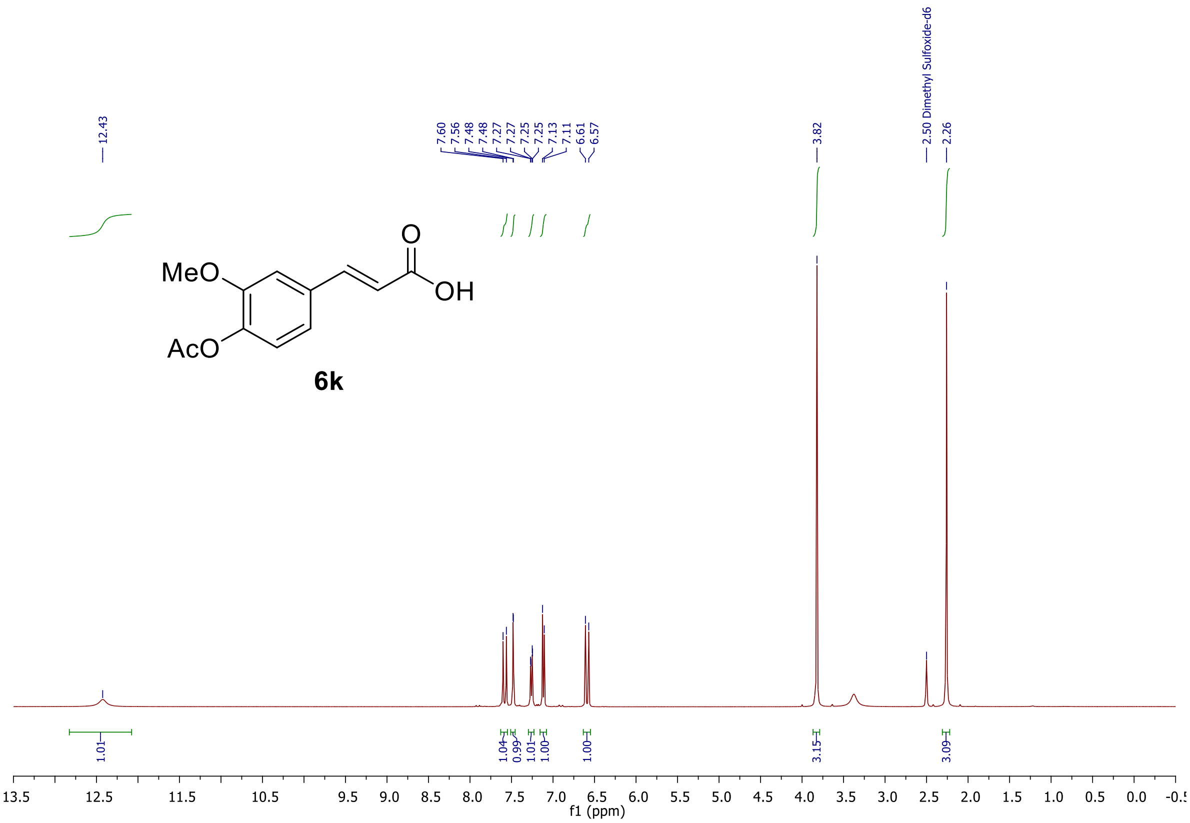

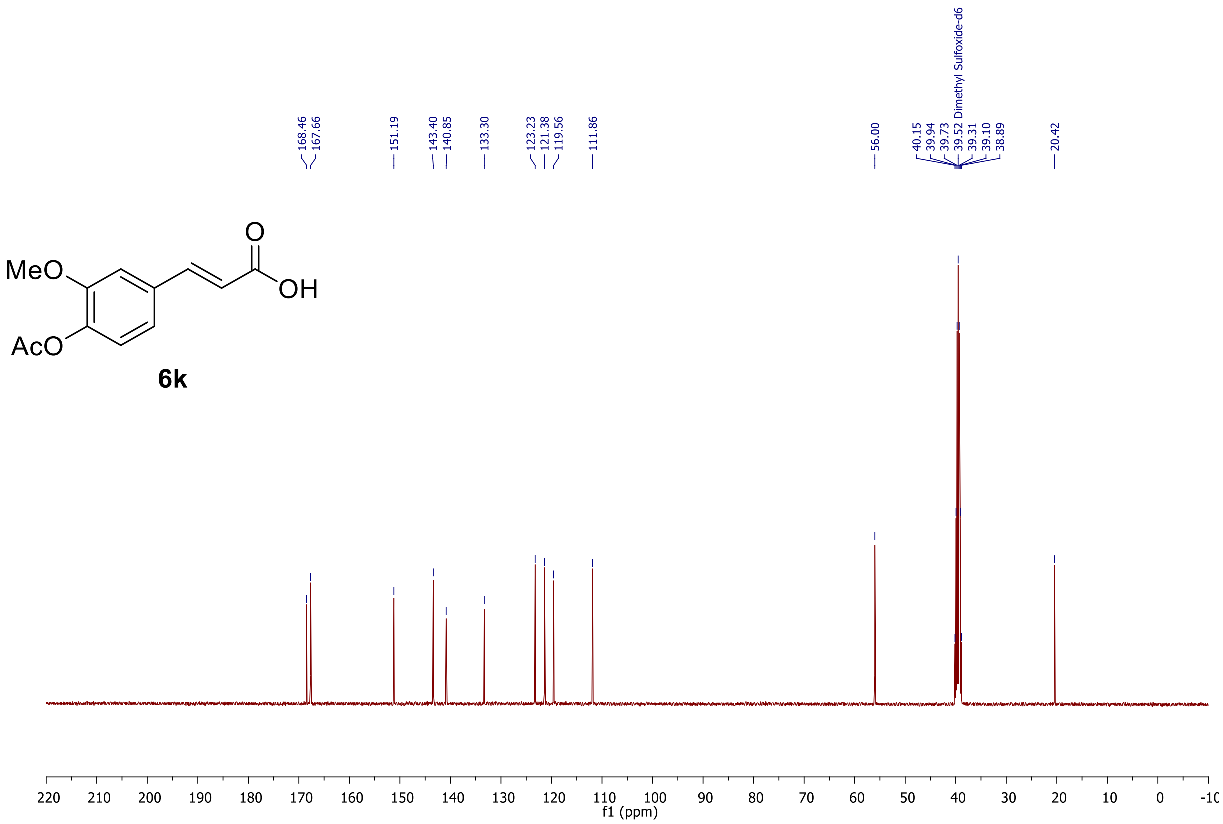

Figure S15: ^{1}H (DMSO-d_6, 400 MHz) and ^{13}C{^{1}H} (DMSO-d_6, 101 MHz) NMR Spectrum of **6k**.

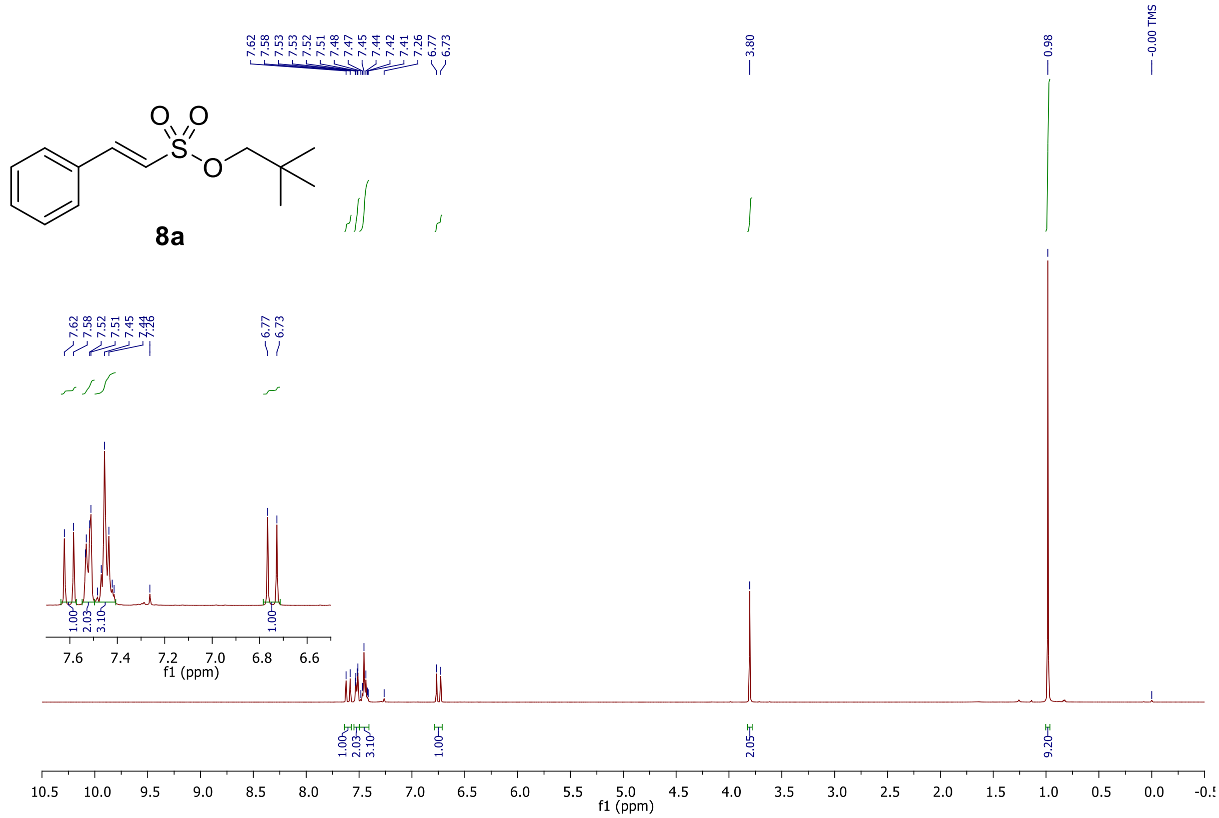

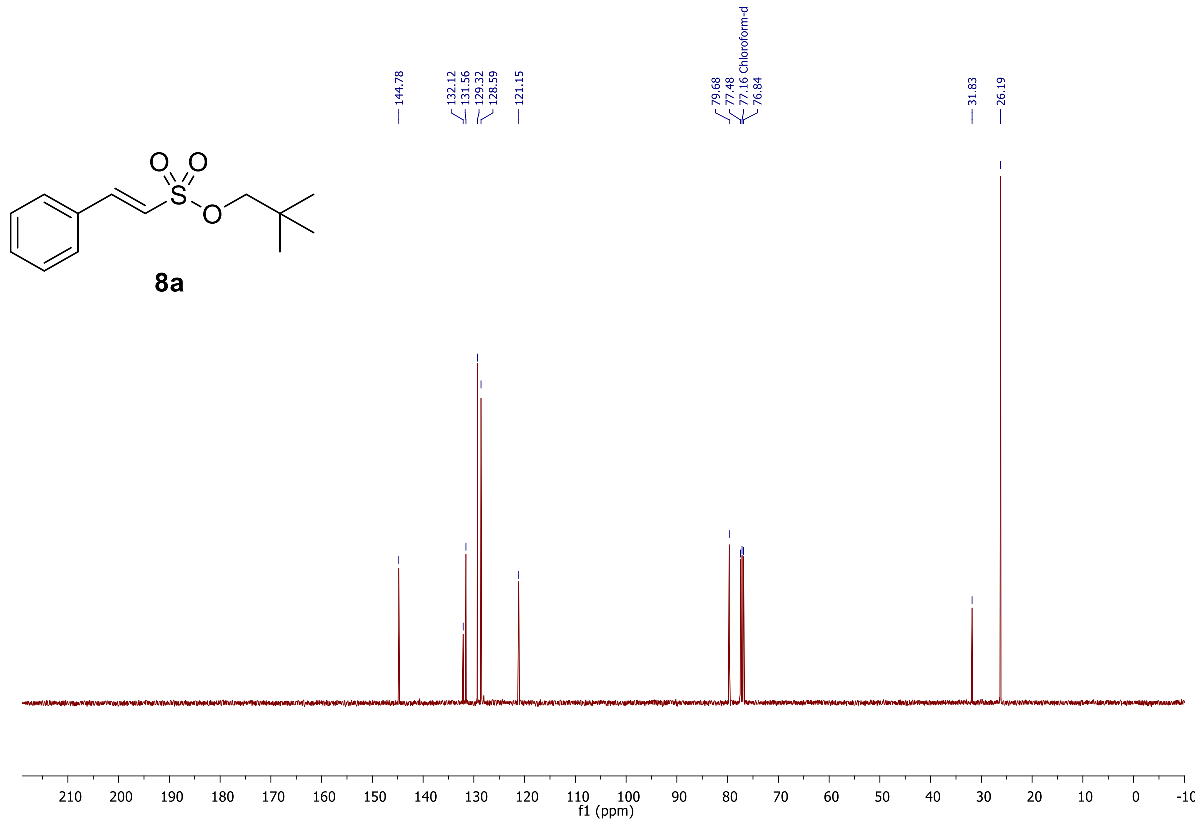

Figure S16: ^{1}H ($CDCl_3$, 400 MHz) and $^{13}C\{^{1}H\}$ ($CDCl_3$, 101 MHz) NMR Spectrum of **8a**.

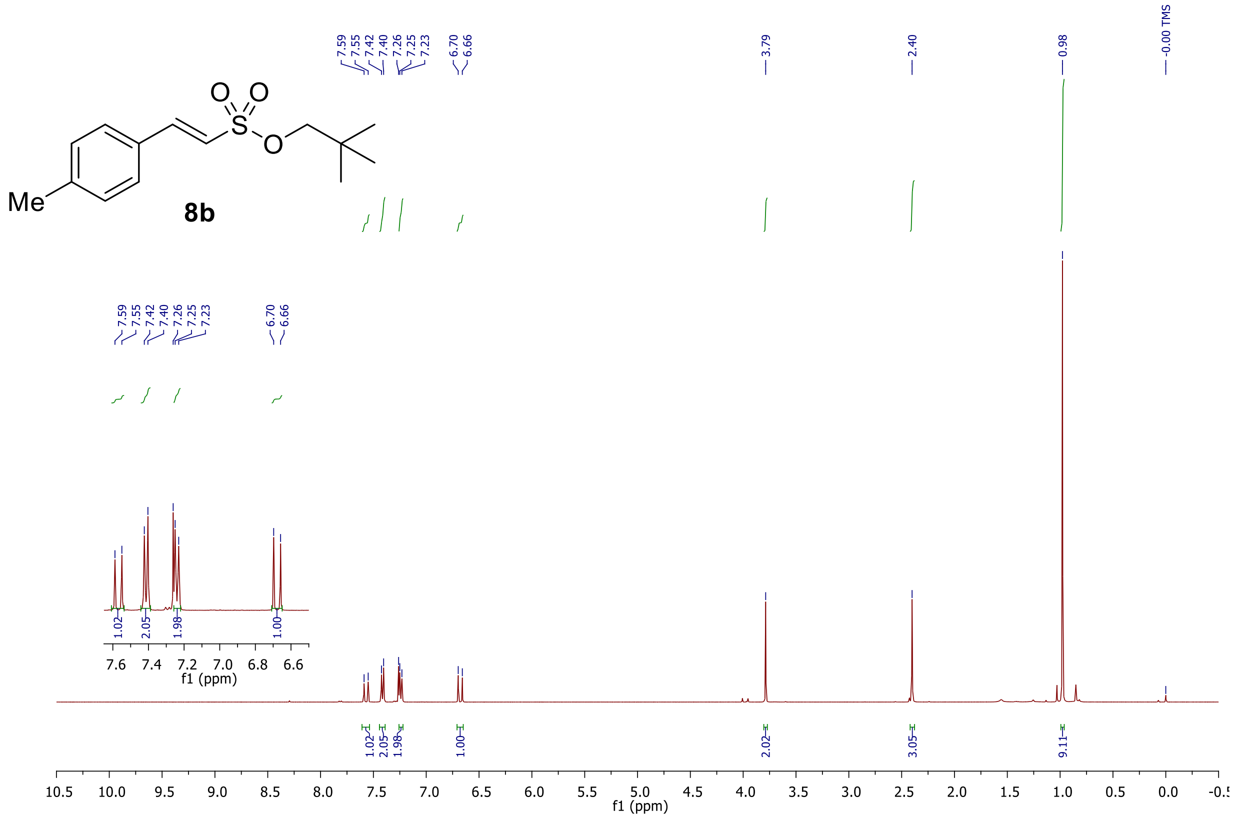

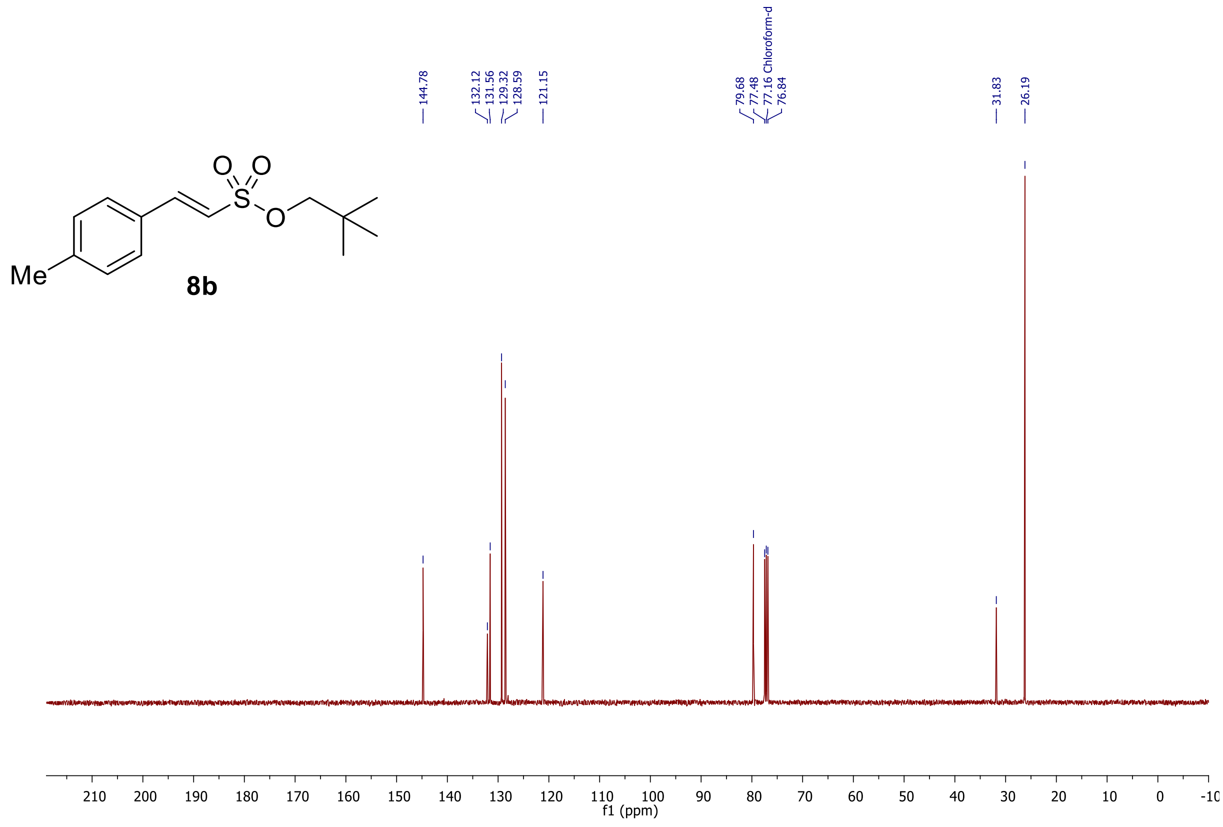

Figure S17: ^{1}H ($CDCl_3$, 400 MHz) and $^{13}C\{^{1}H\}$ ($CDCl_3$, 101 MHz) NMR Spectrum of **8b**.

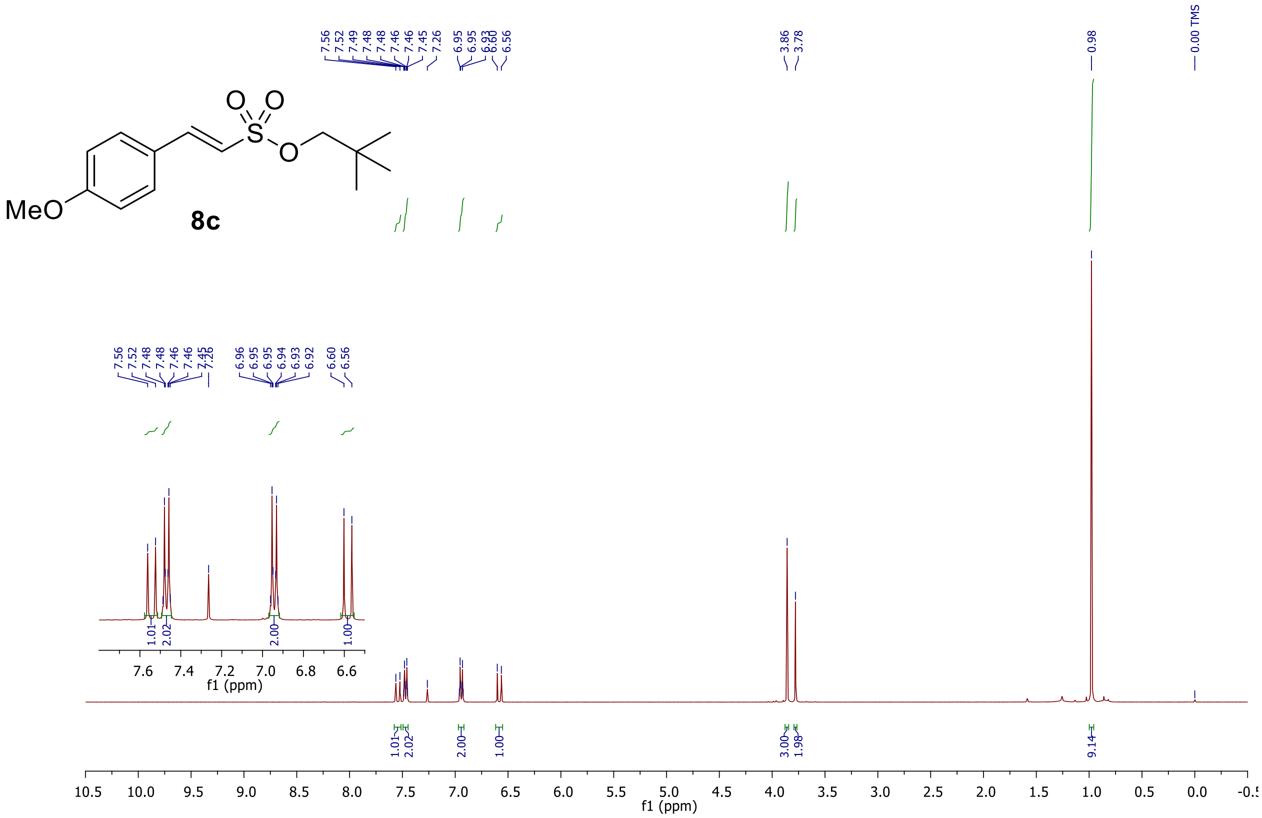

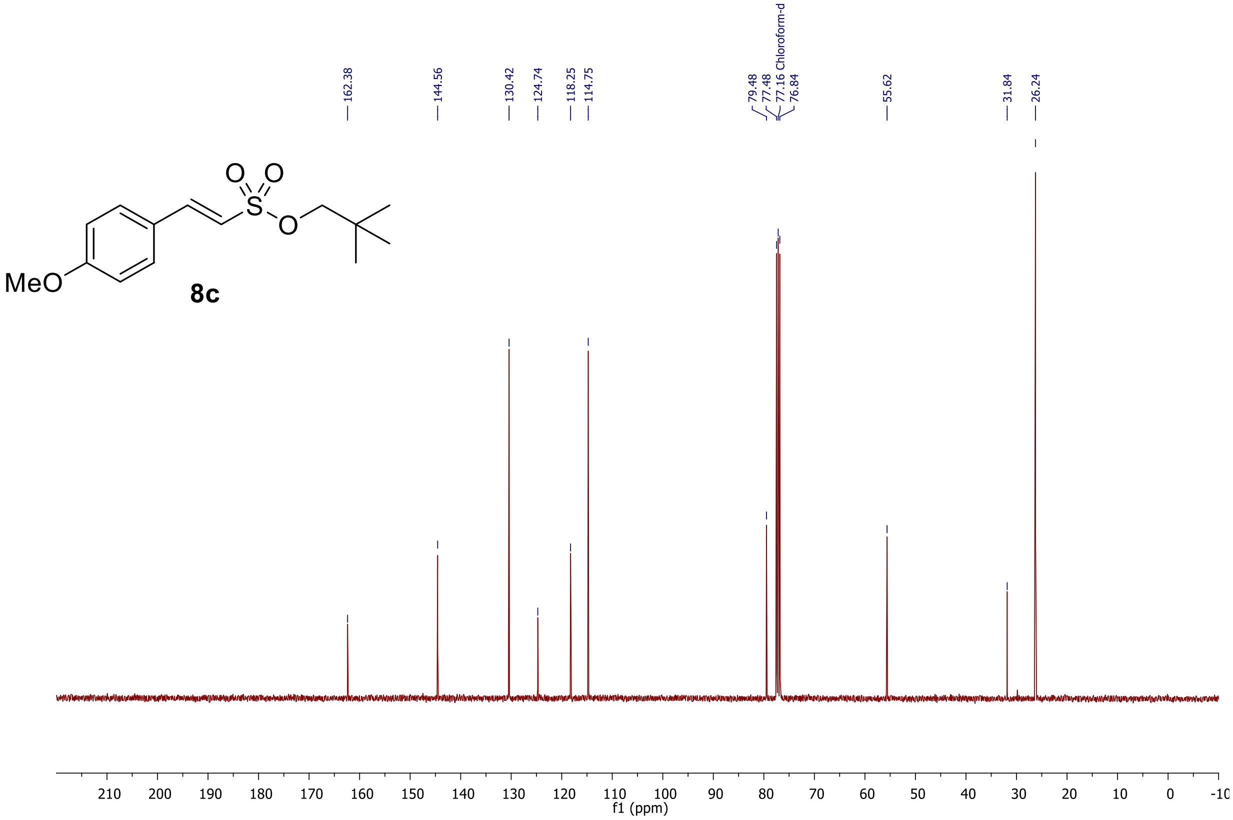

Figure S18: ^{1}H ($CDCl_3$, 400 MHz) and $^{13}C\{^1H\}$ ($CDCl_3$, 101 MHz) NMR Spectrum of **8c**.

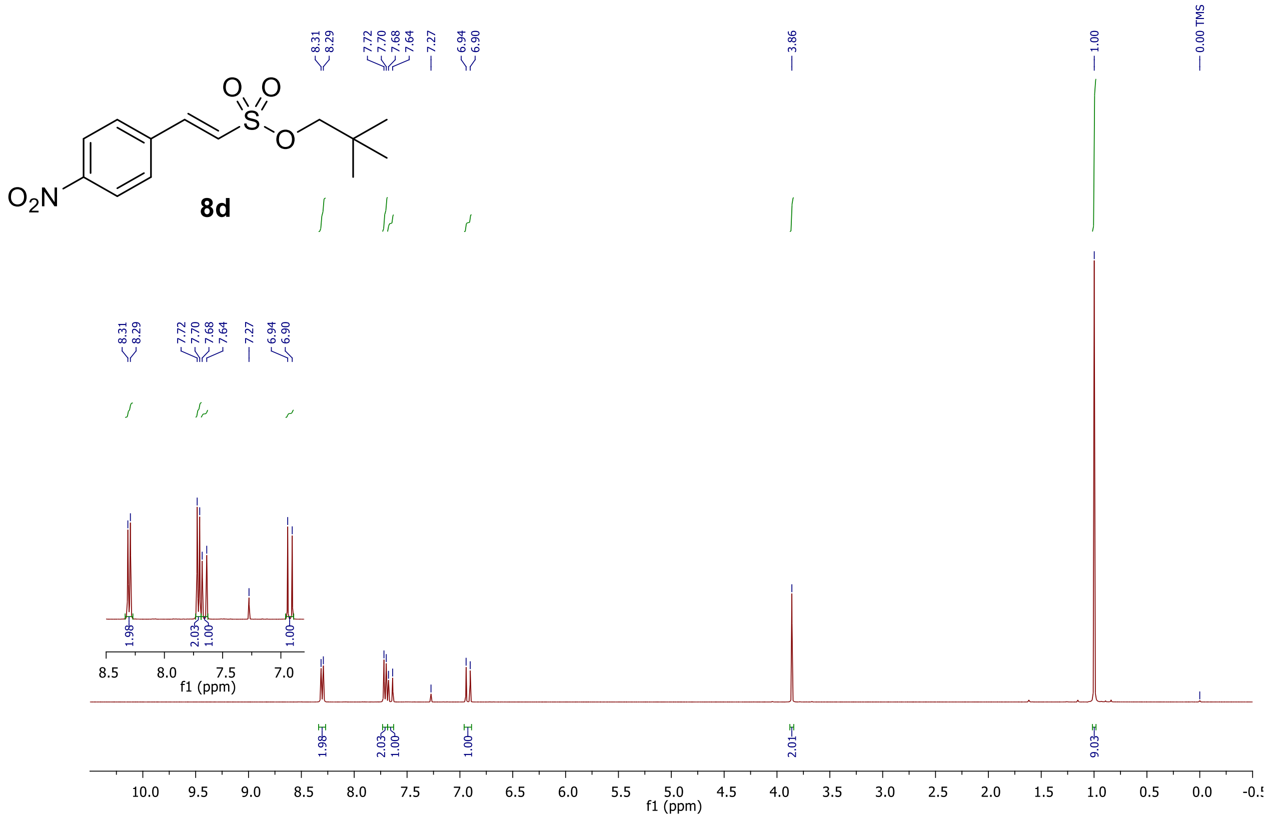

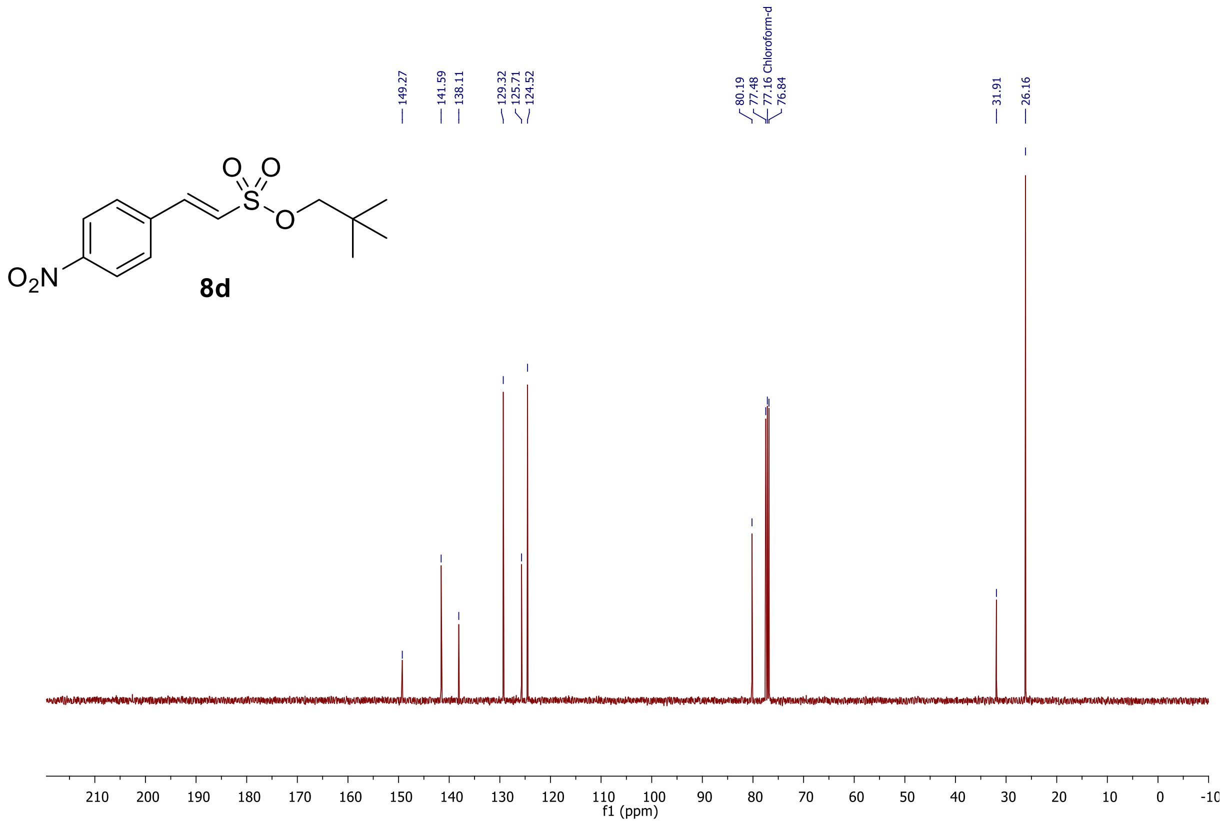

Figure S19: ^{1}H ($CDCl_3$, 400 MHz) and $^{13}C\{^{1}H\}$ ($CDCl_3$, 101 MHz) NMR Spectrum of **8d**.

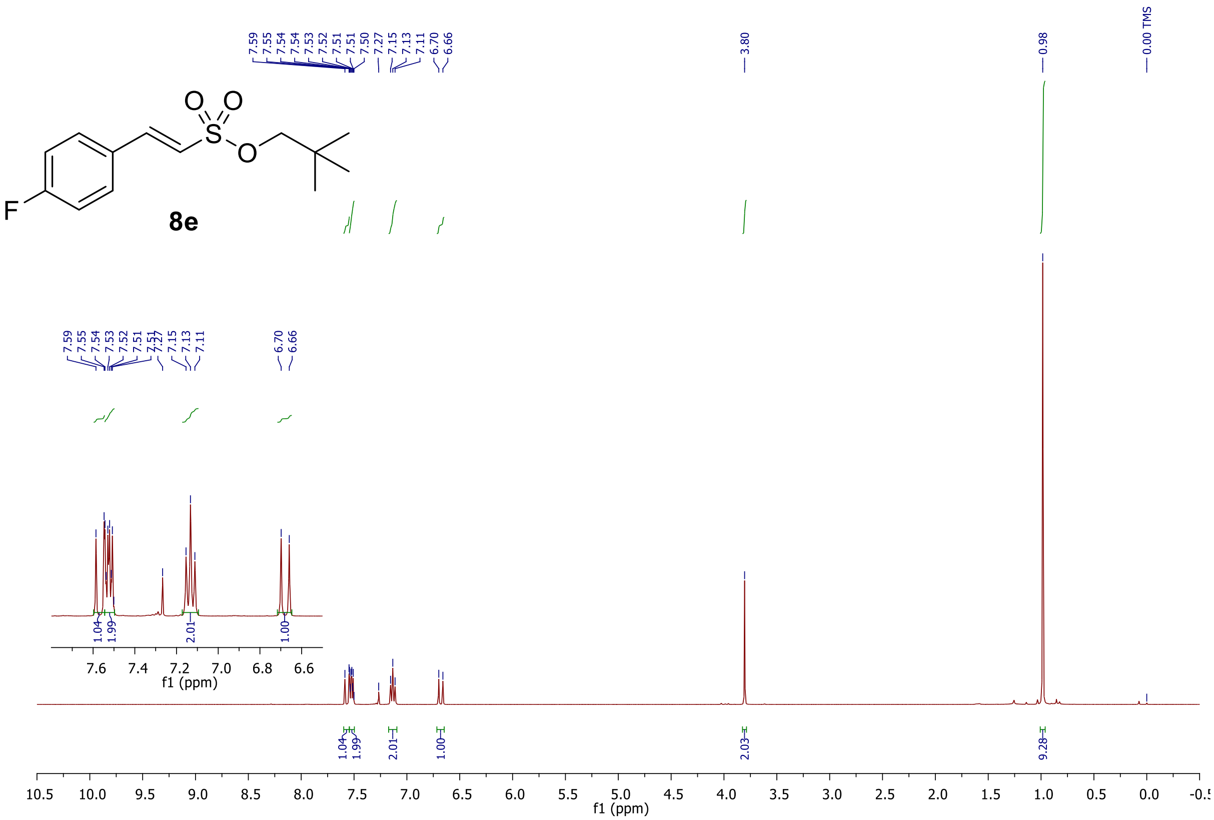
8e
f1 (ppm)

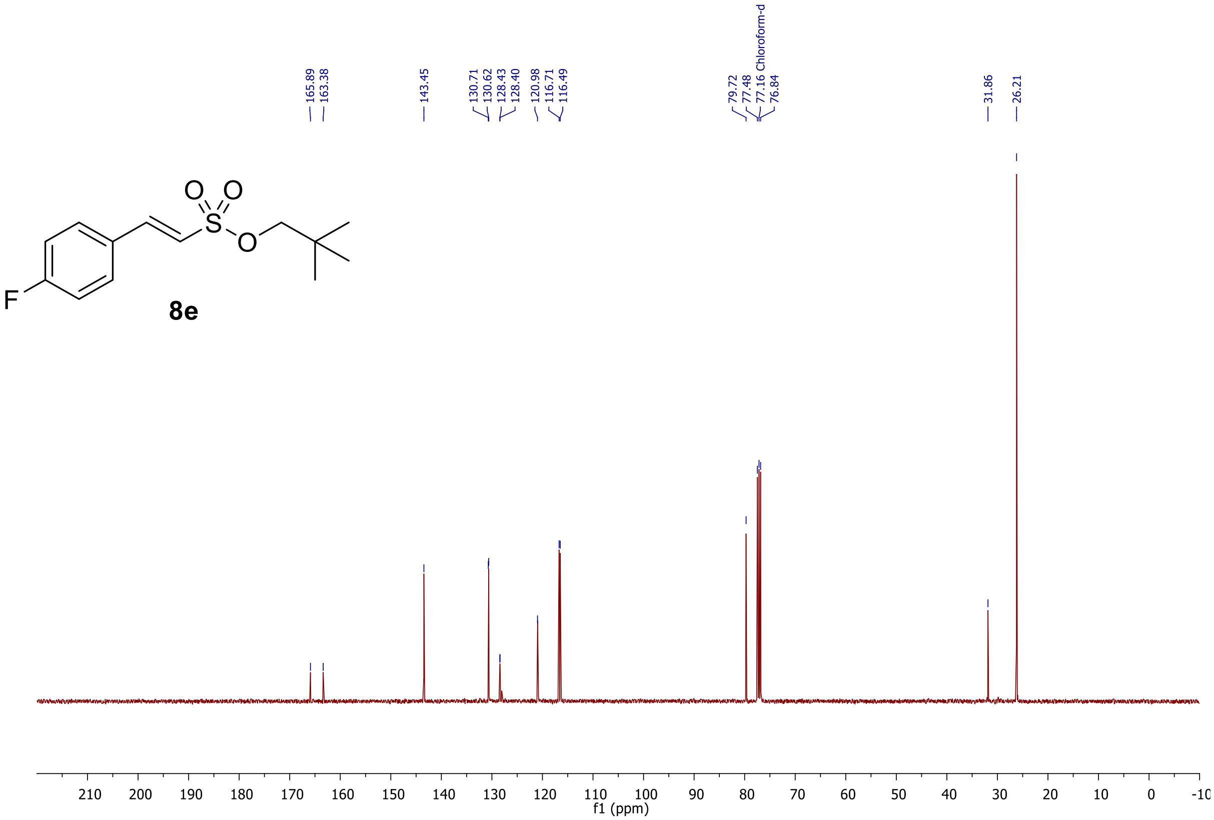
8e
f1 (ppm)

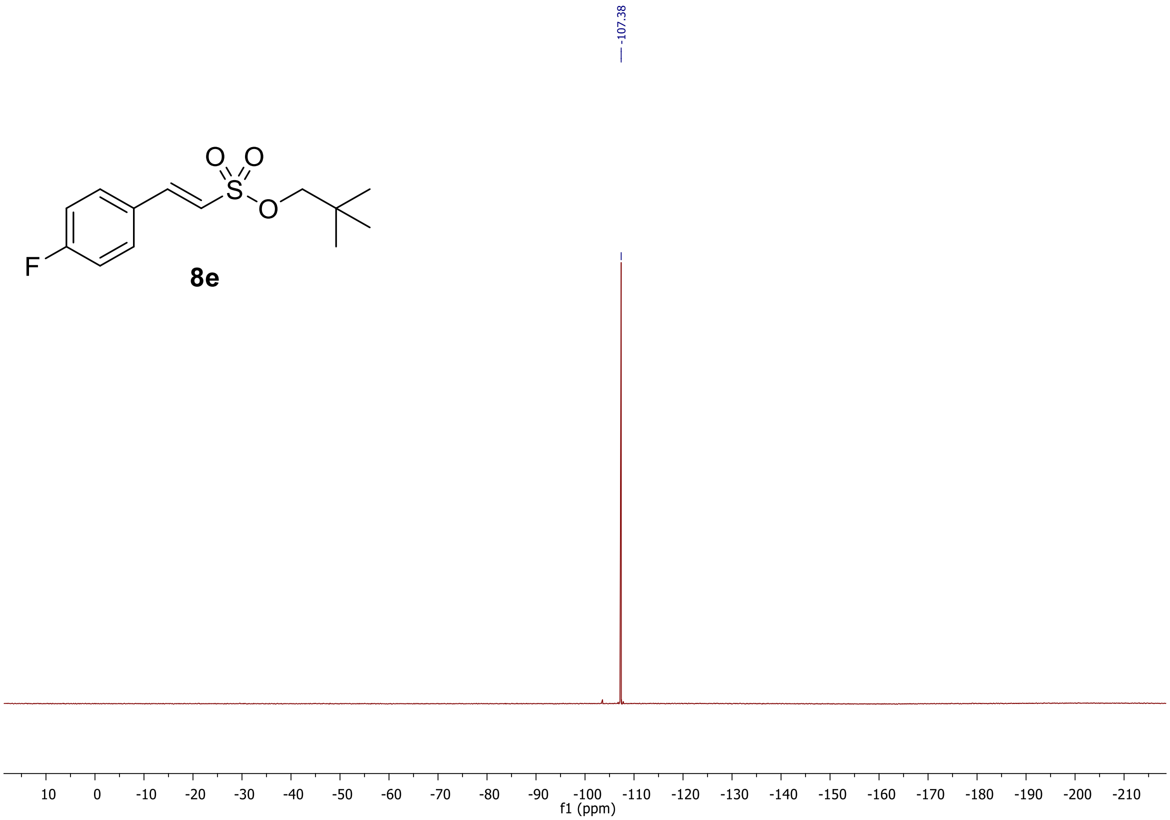

Figure S20: ^{1}H ($CDCl_3$, 400 MHz), $^{13}C\{^{1}H\}$ ($CDCl_3$, 101 MHz), and $^{19}F\{^{1}H\}$ ($CDCl_3$, 376 MHz) NMR Spectrum of **8e**.

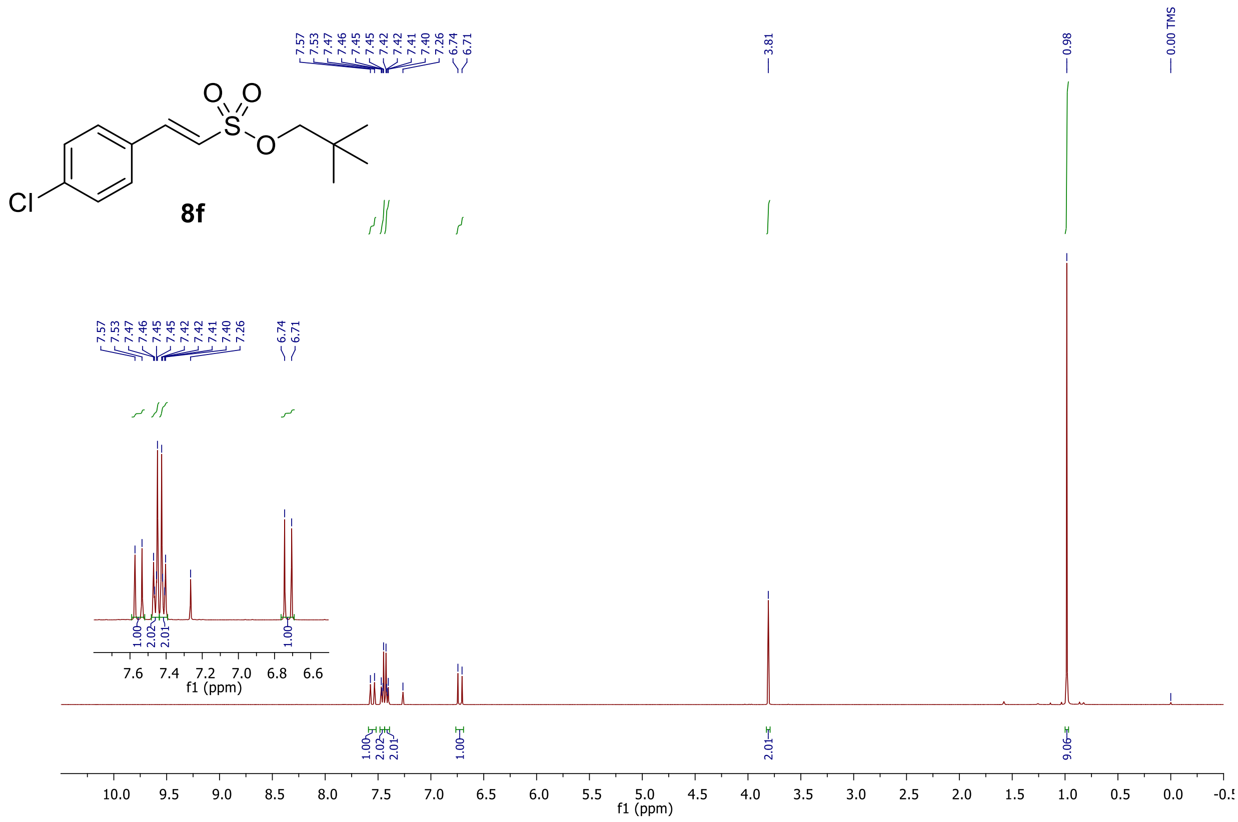

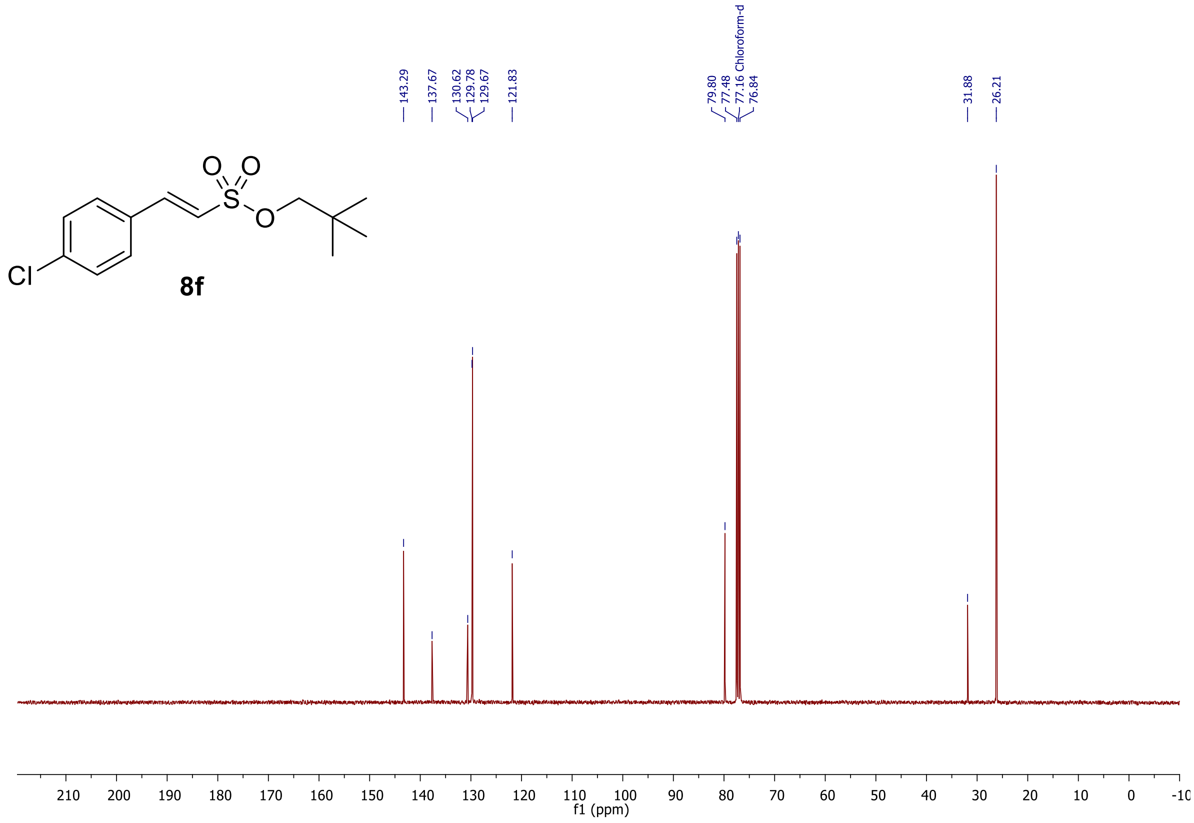

Figure S21: ^{1}H ($CDCl_3$, 400 MHz) and $^{13}C\{^1H\}$ ($CDCl_3$, 101 MHz) NMR Spectrum of **8f**.

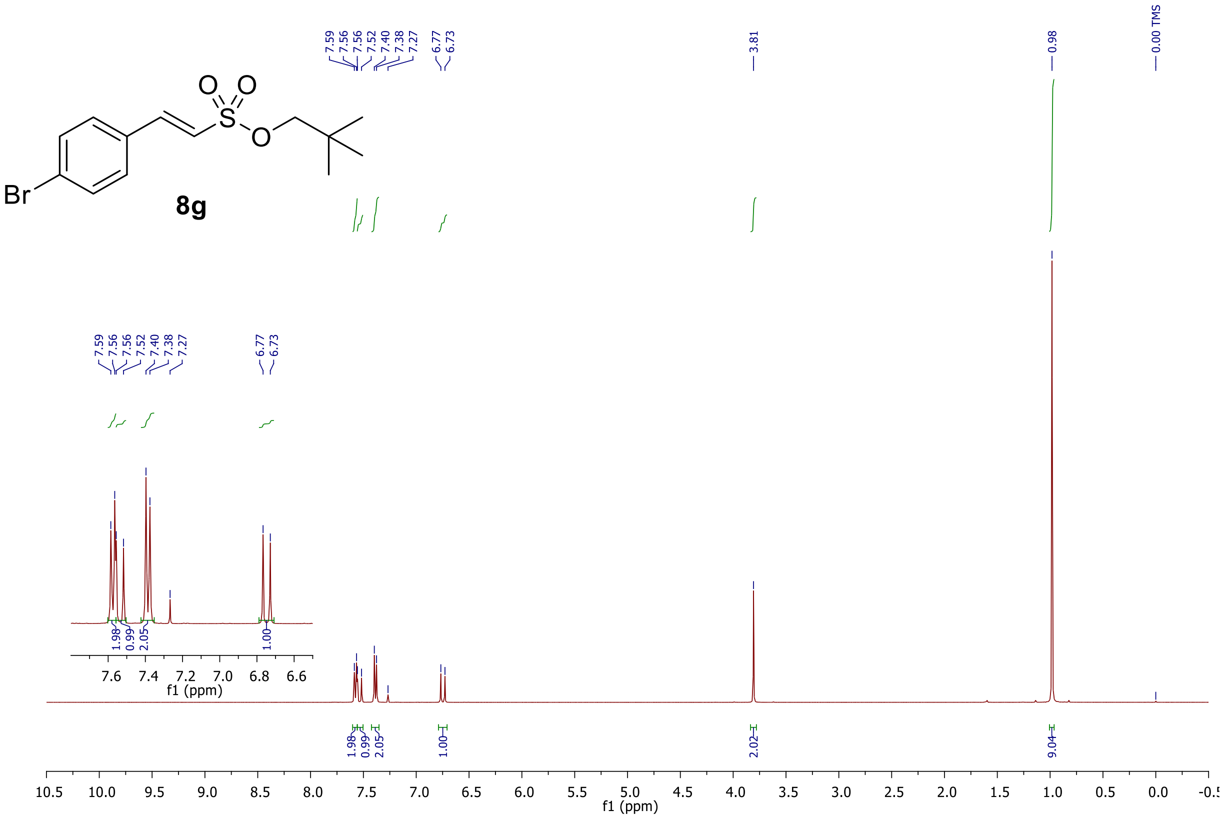

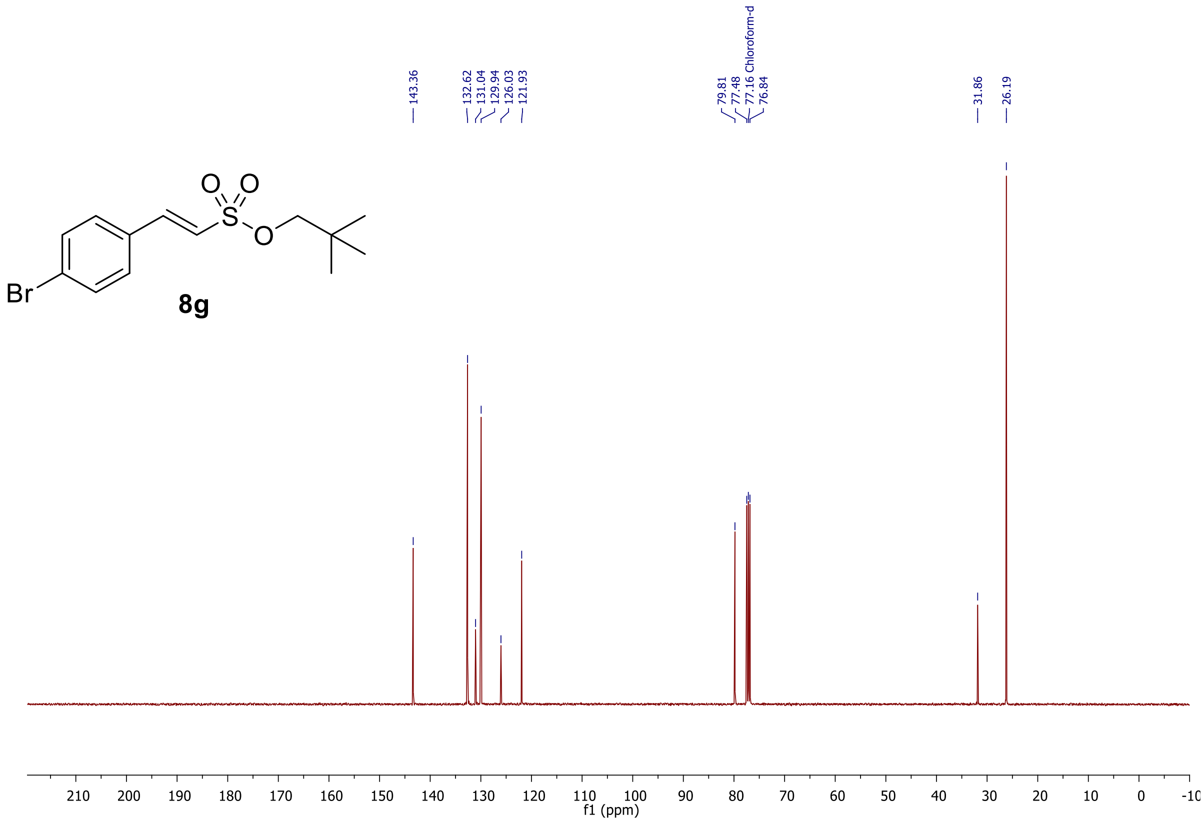

Figure S22: ¹H ($CDCl_3$, 400 MHz) and $^{13}C\{^1H\}$ ($CDCl_3$, 101 MHz) NMR Spectrum of **8g**.

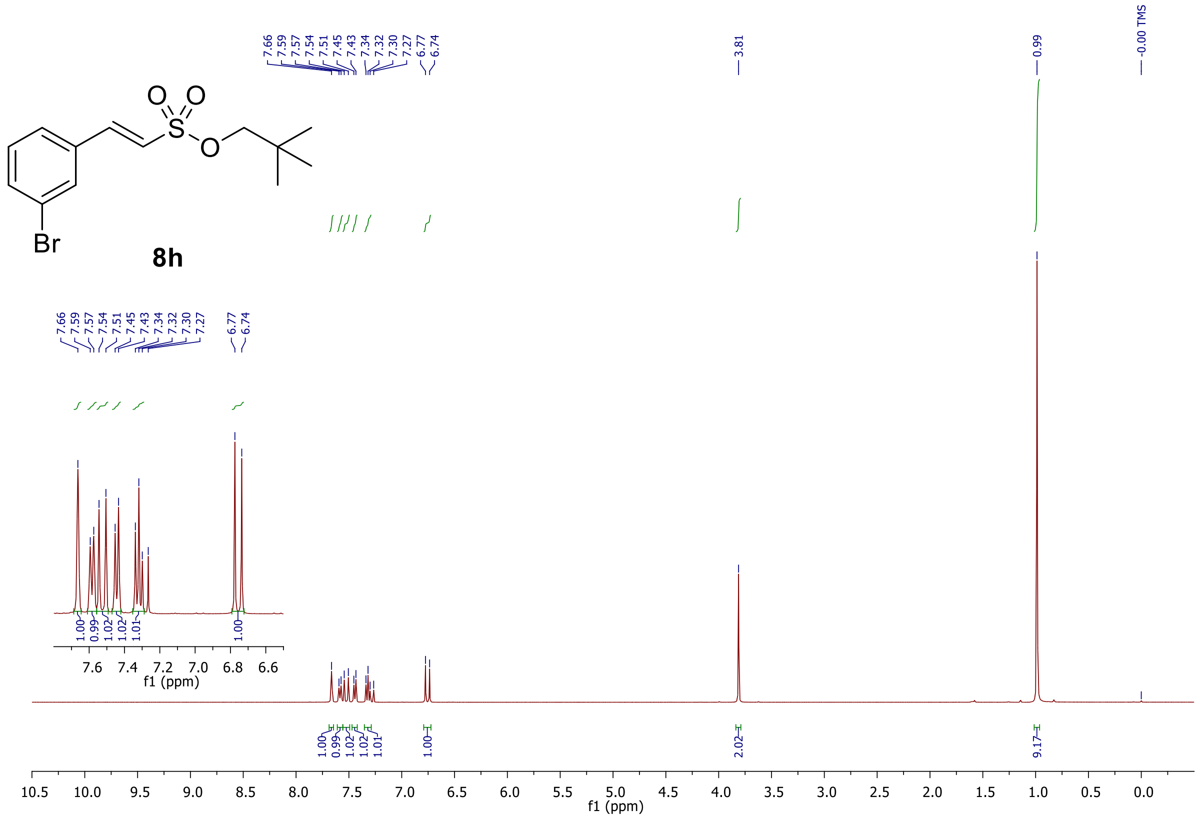

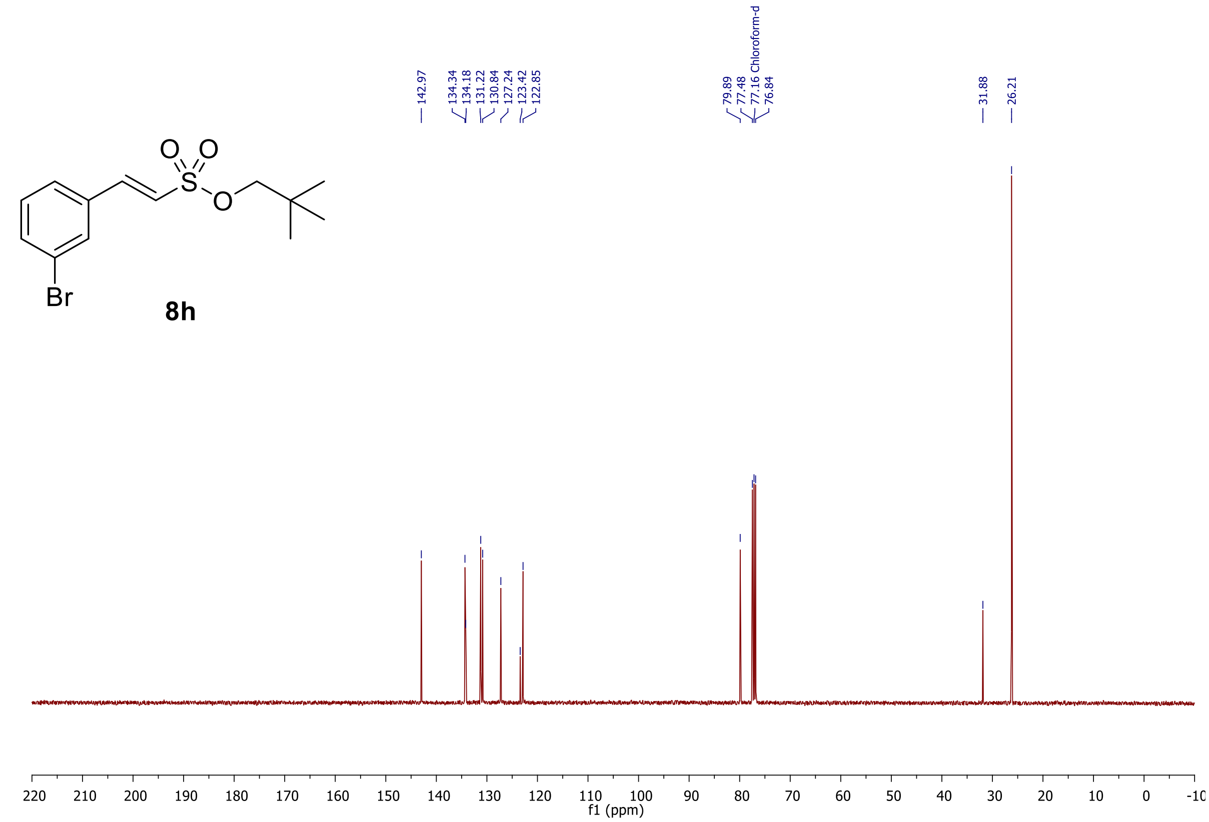

Figure S23: ^{1}H ($CDCl_3$, 400 MHz) and $^{13}C\{^{1}H\}$ ($CDCl_3$, 101 MHz) NMR Spectrum of **8h**.

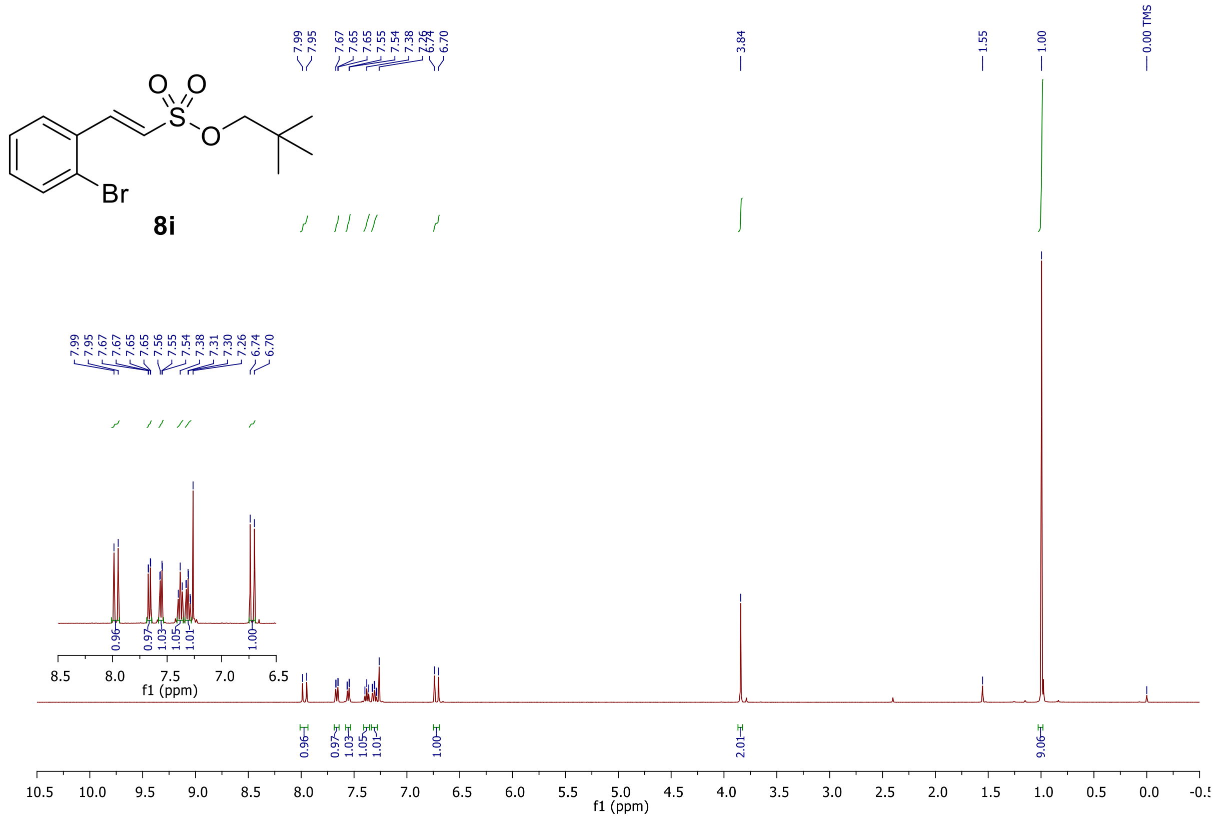

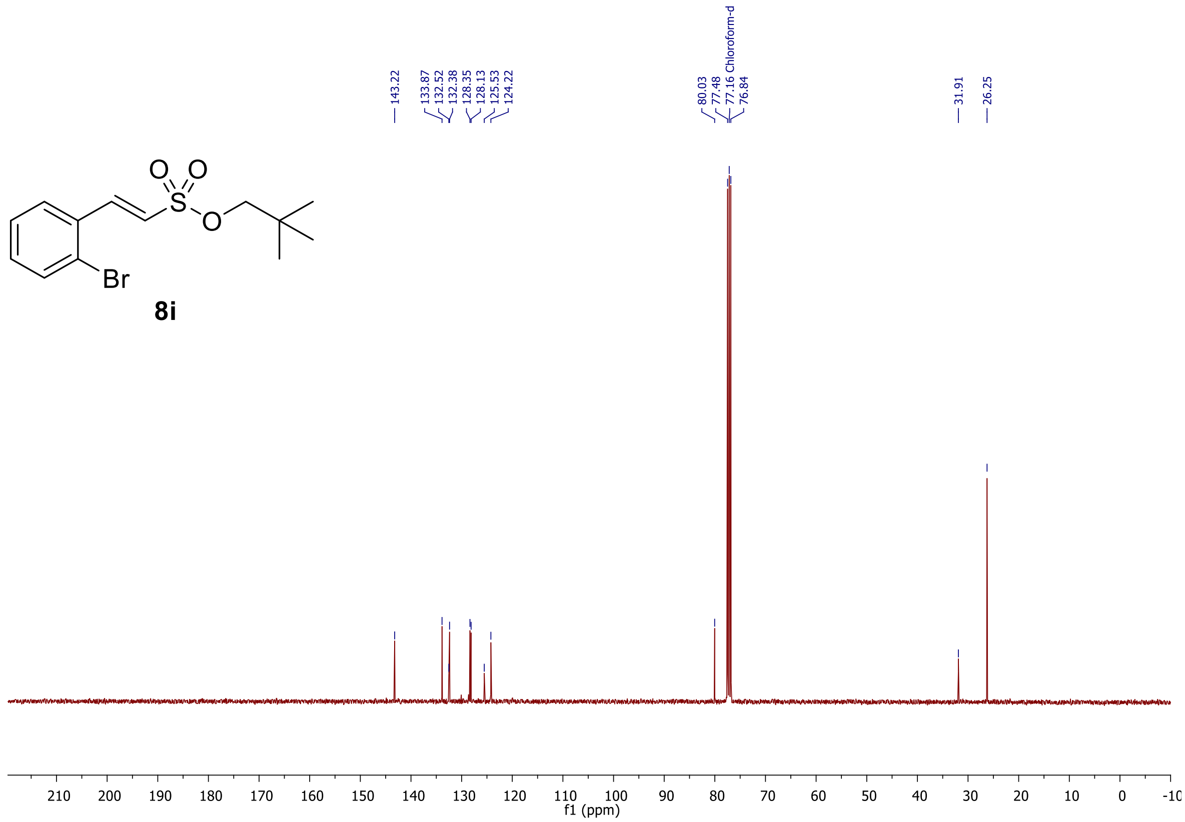

Figure S24: ^{1}H ($CDCl_3$, 400 MHz) and $^{13}C\{^1H\}$ ($CDCl_3$, 101 MHz) NMR Spectrum of **8i**.

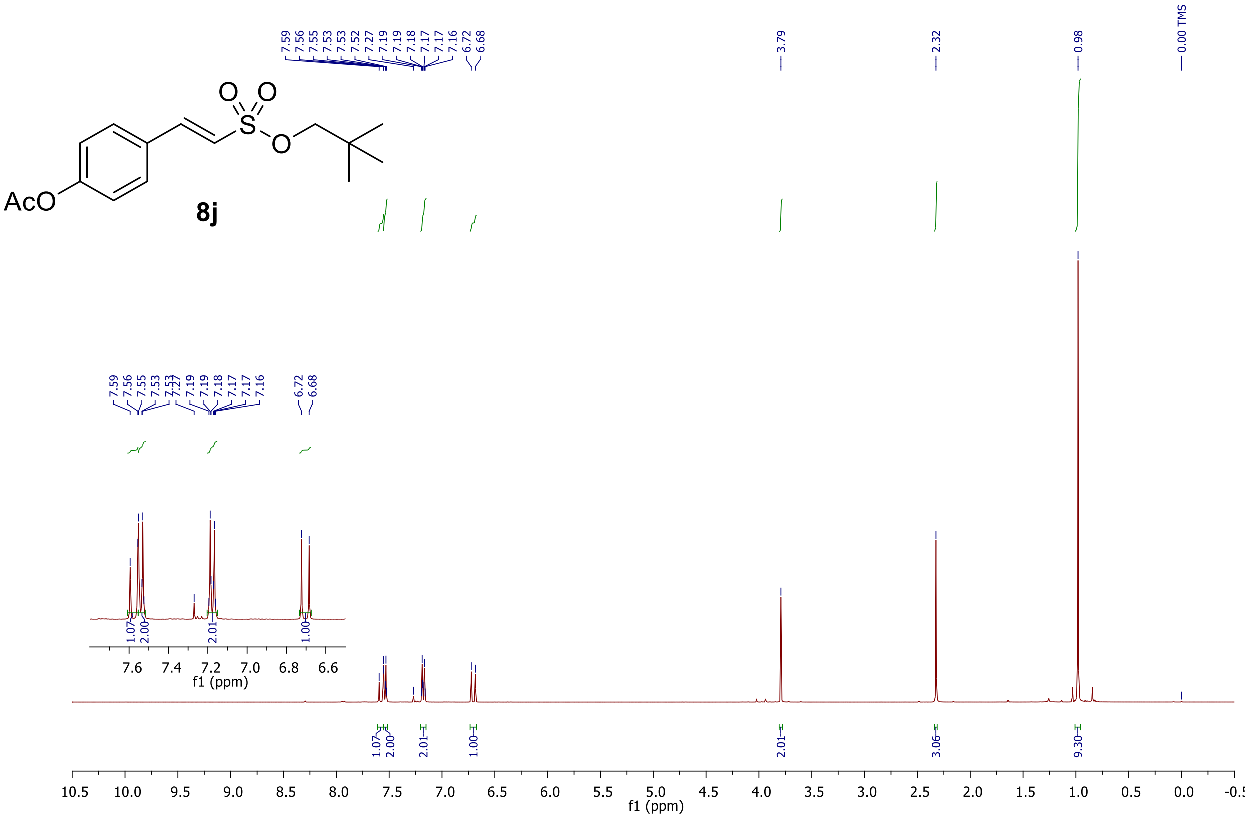

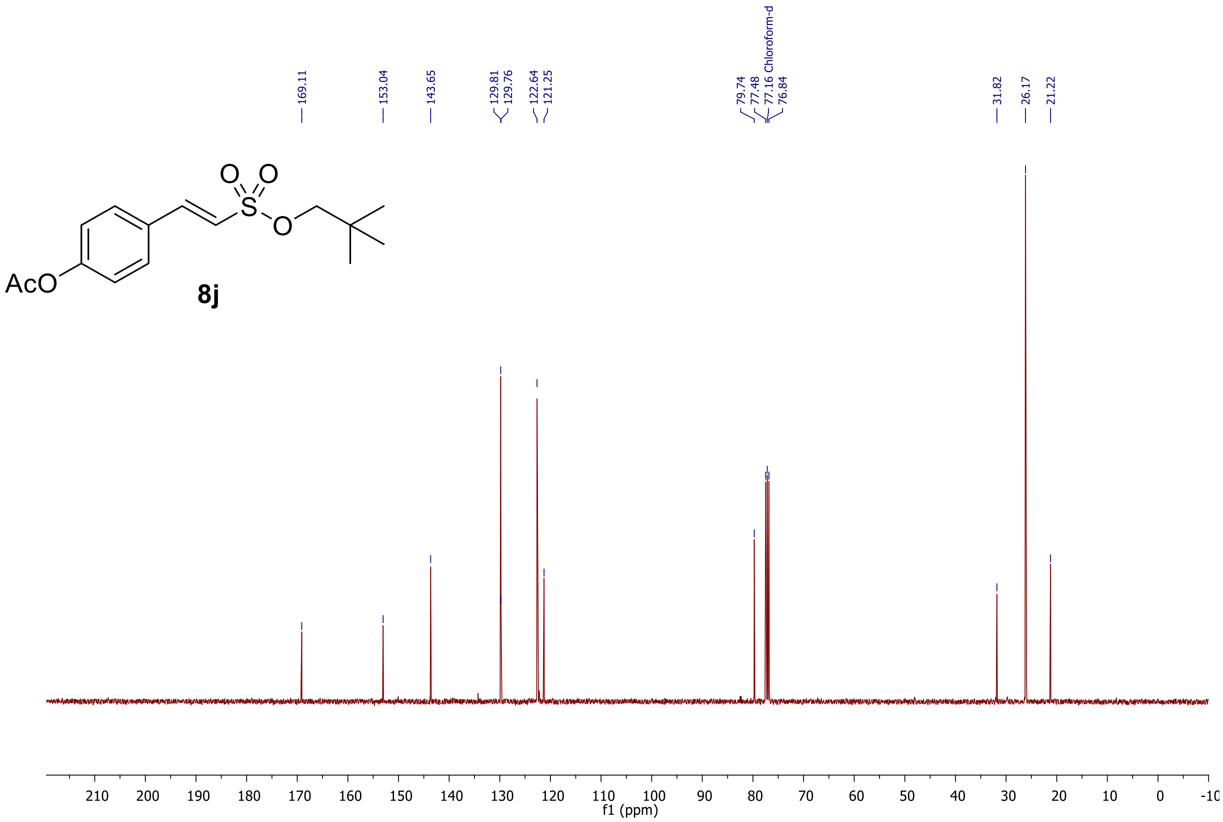

Figure S25: ^{1}H ($CDCl_3$, 400 MHz) and ${}^{13}C\{{}^{1}H\}$ ($CDCl_3$, 101 MHz) NMR Spectrum of **8j**.

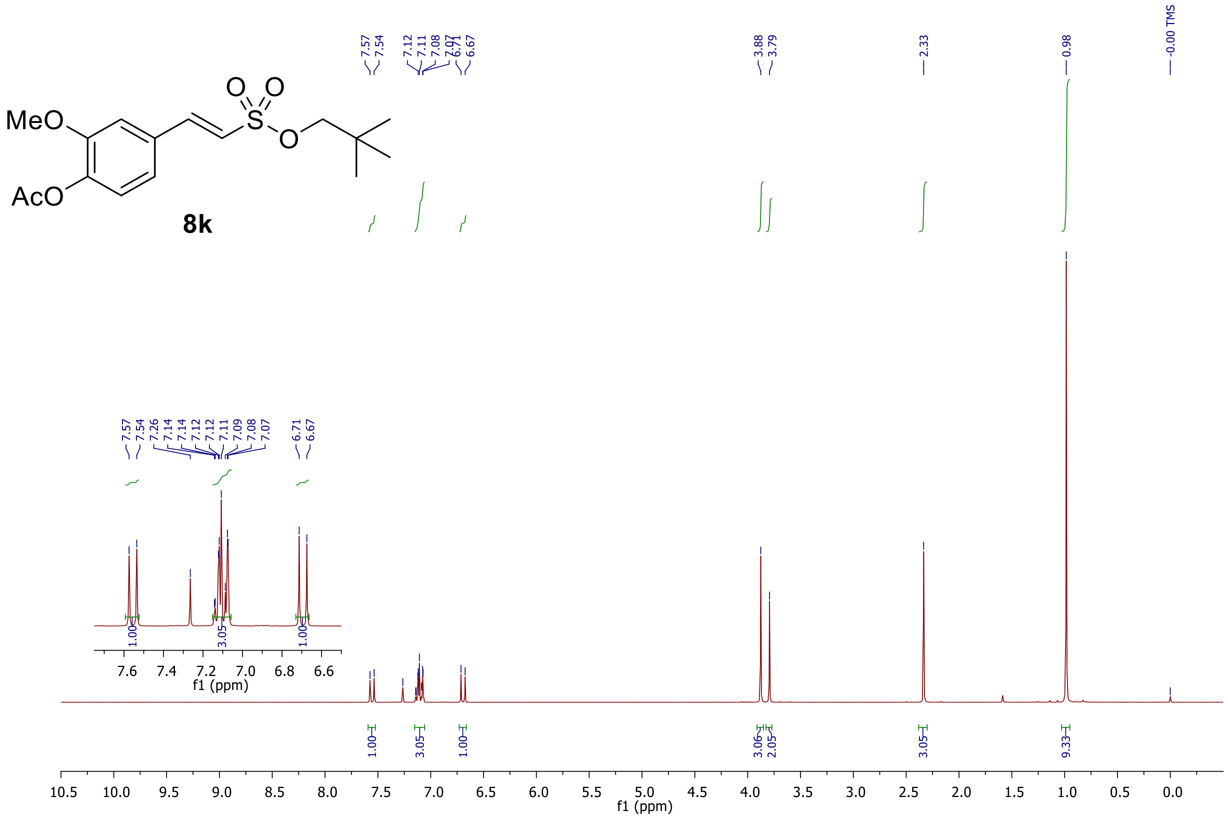

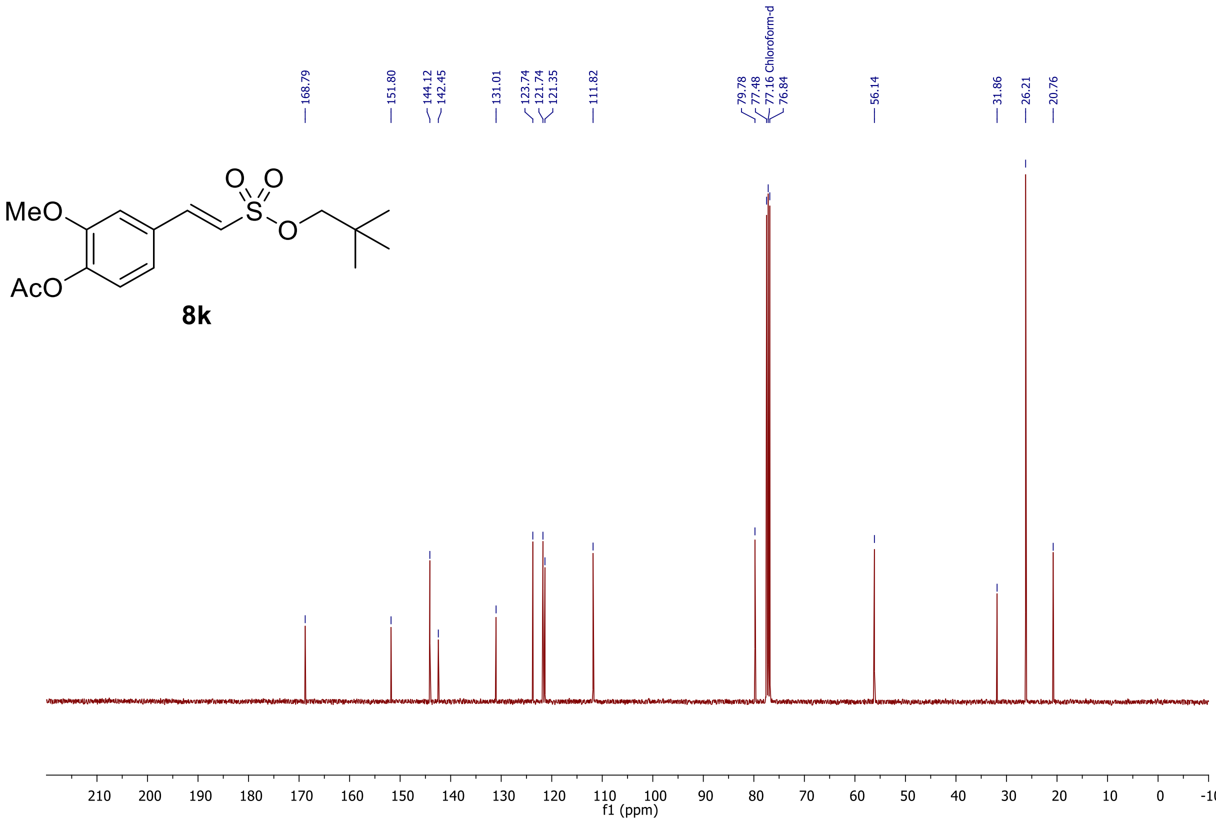

Figure S26: ^{1}H ($CDCl_3$, 400 MHz) and $^{13}C\{^{1}H\}$ ($CDCl_3$, 101 MHz) NMR Spectrum of **8k**.

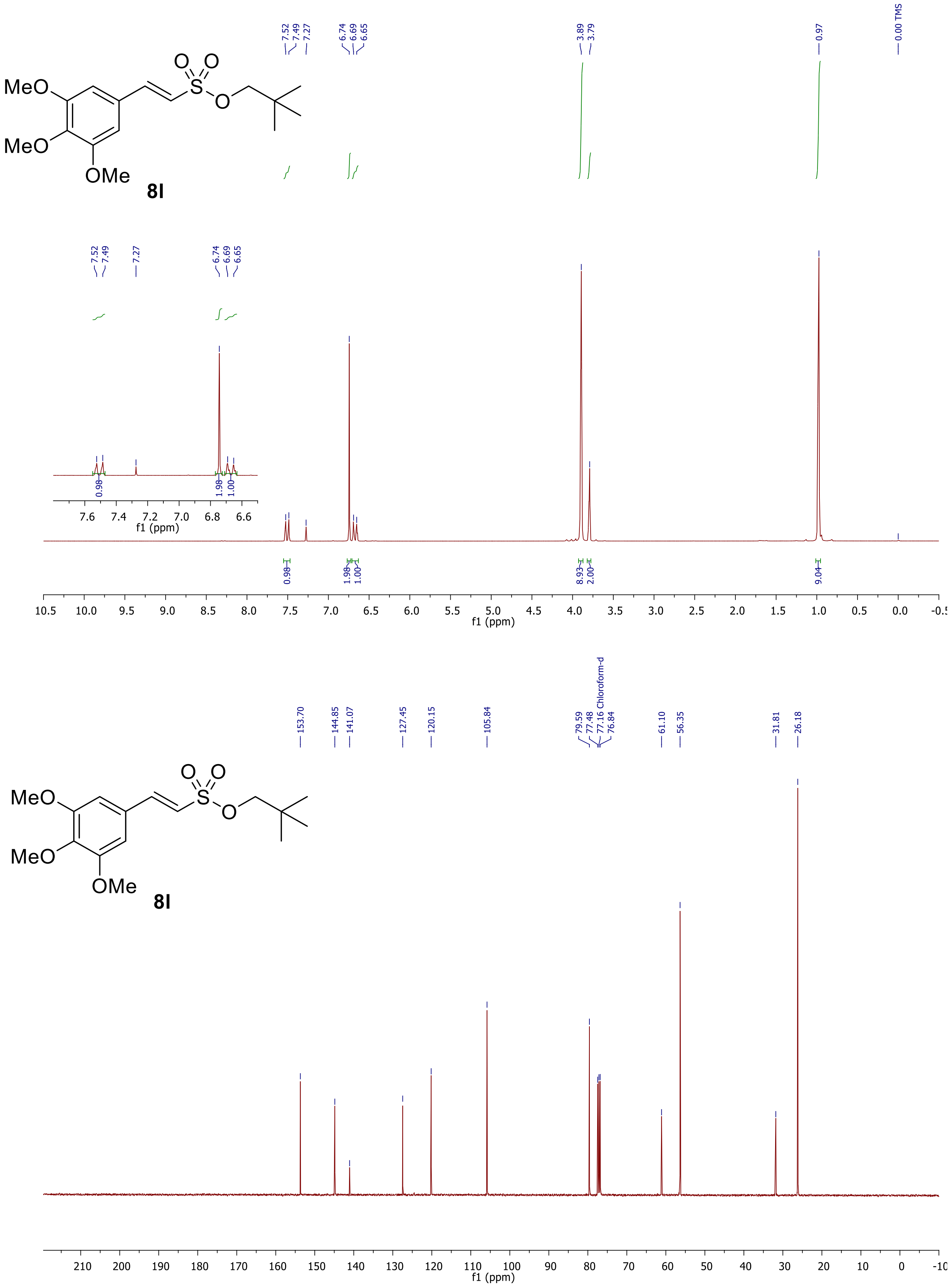

Figure S27: ^{1}H ($CDCl_3$, 400 MHz) and $^{13}C\{^{1}H\}$ ($CDCl_3$, 101 MHz) NMR Spectrum of **8l**.

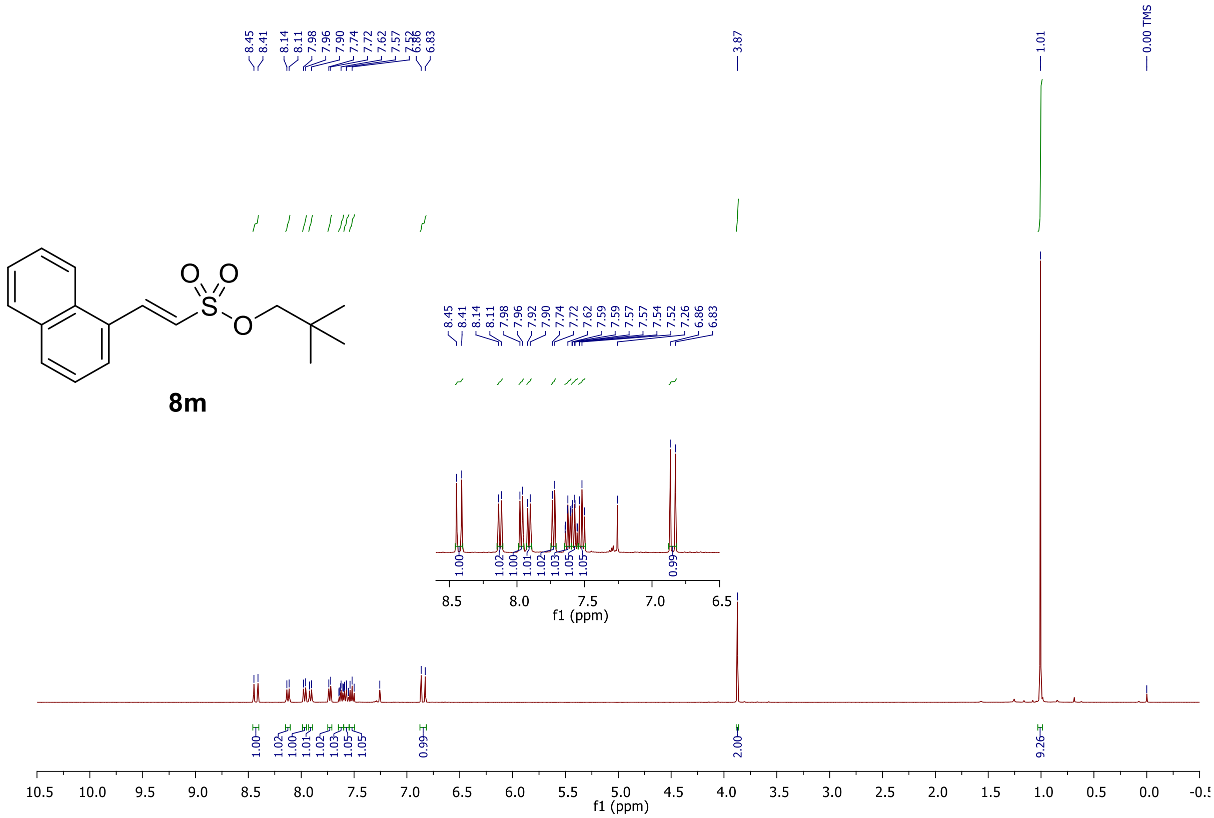

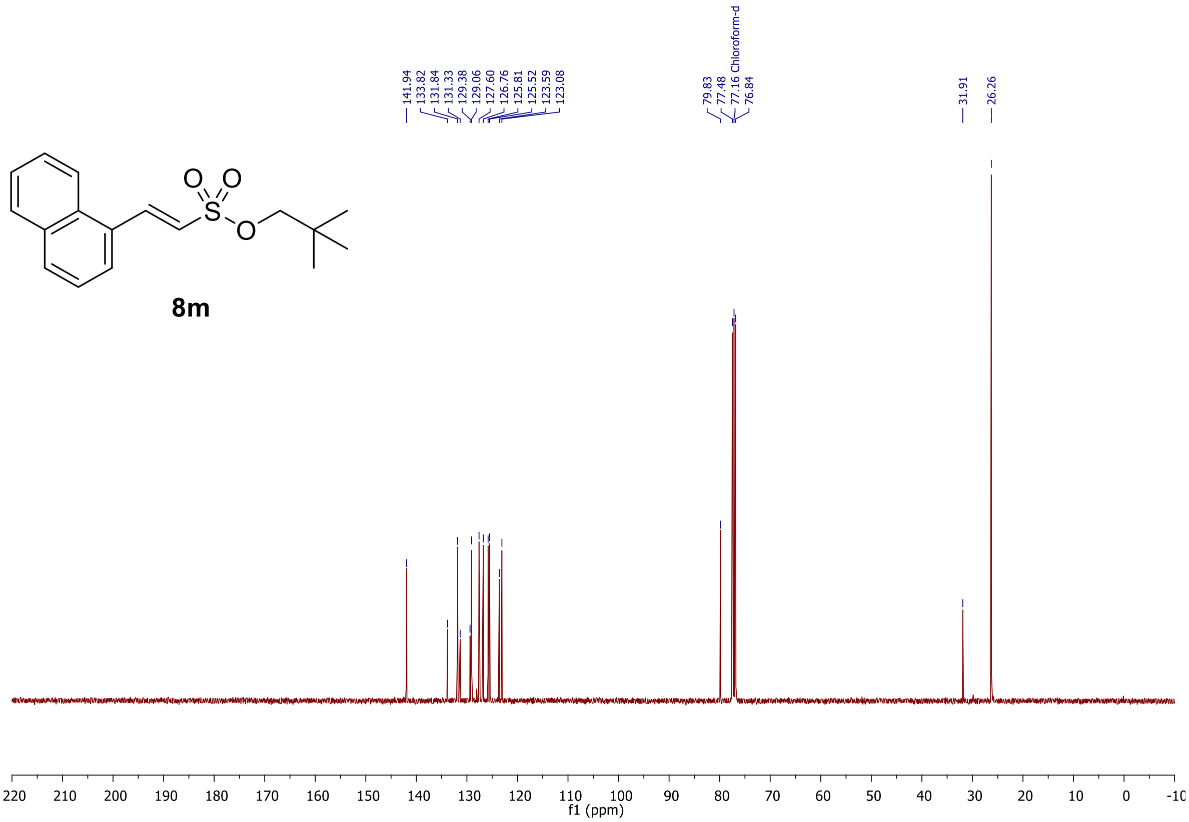

Figure S28: ^{1}H ($CDCl_3$, 400 MHz) and $^{13}C\{^{1}H\}$ ($CDCl_3$, 101 MHz) NMR Spectrum of **8m**.

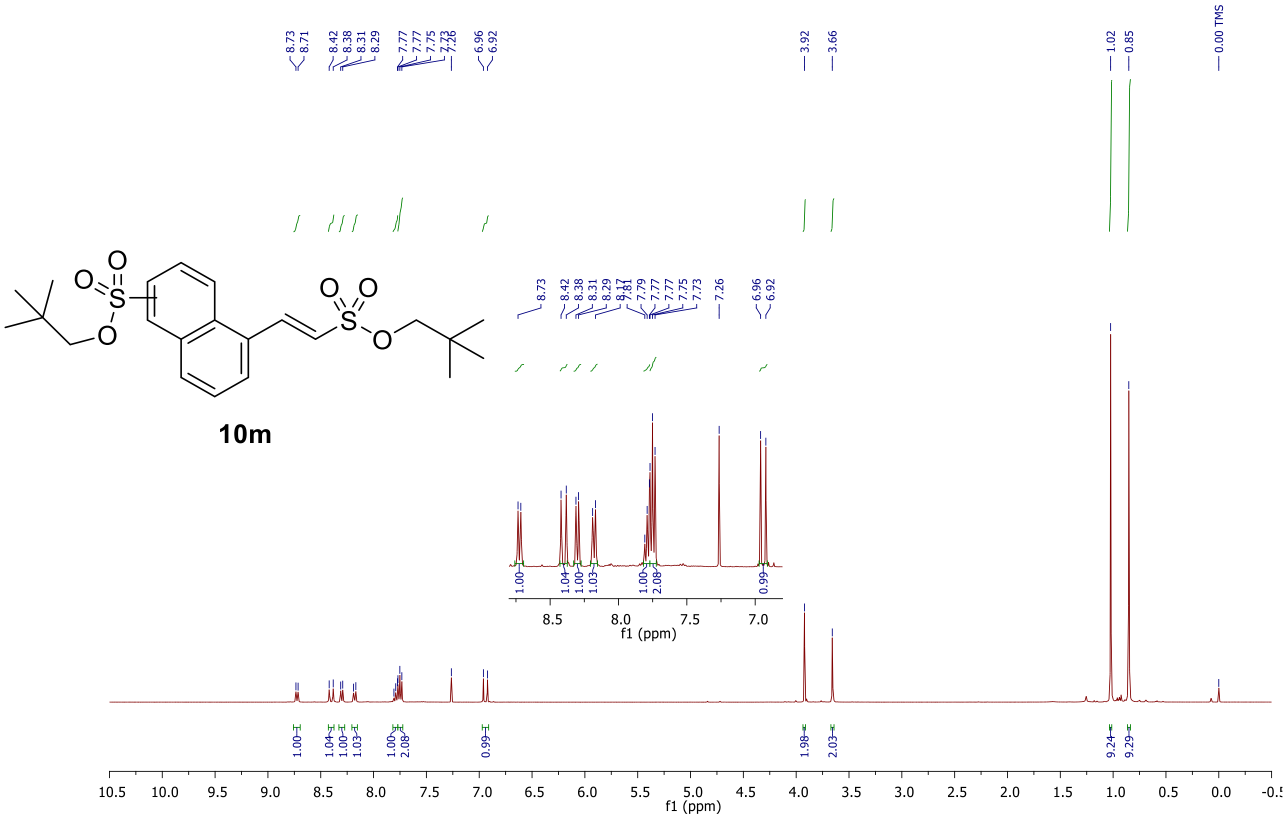

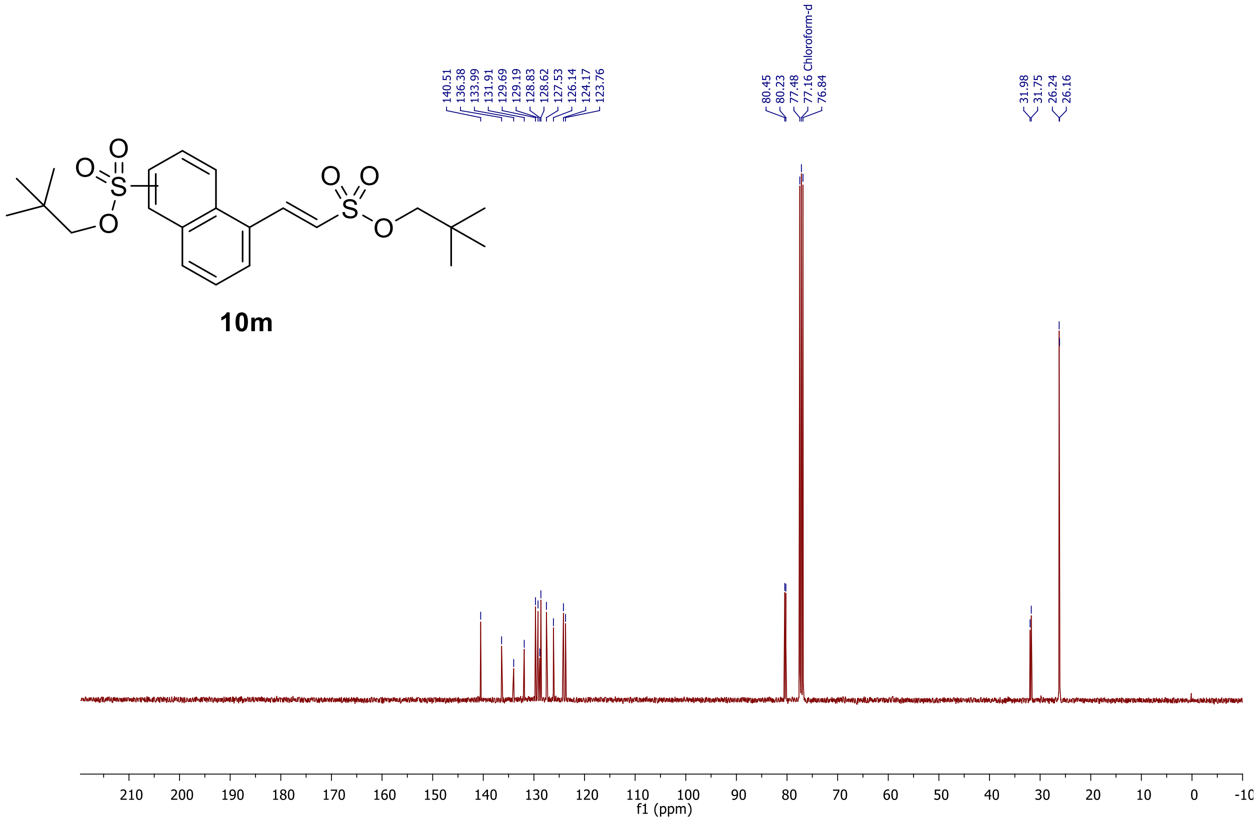

Figure S29: ^{1}H ($CDCl_3$, 400 MHz) and $^{13}C\{^{1}H\}$ ($CDCl_3$, 101 MHz) NMR Spectrum of **10m**.

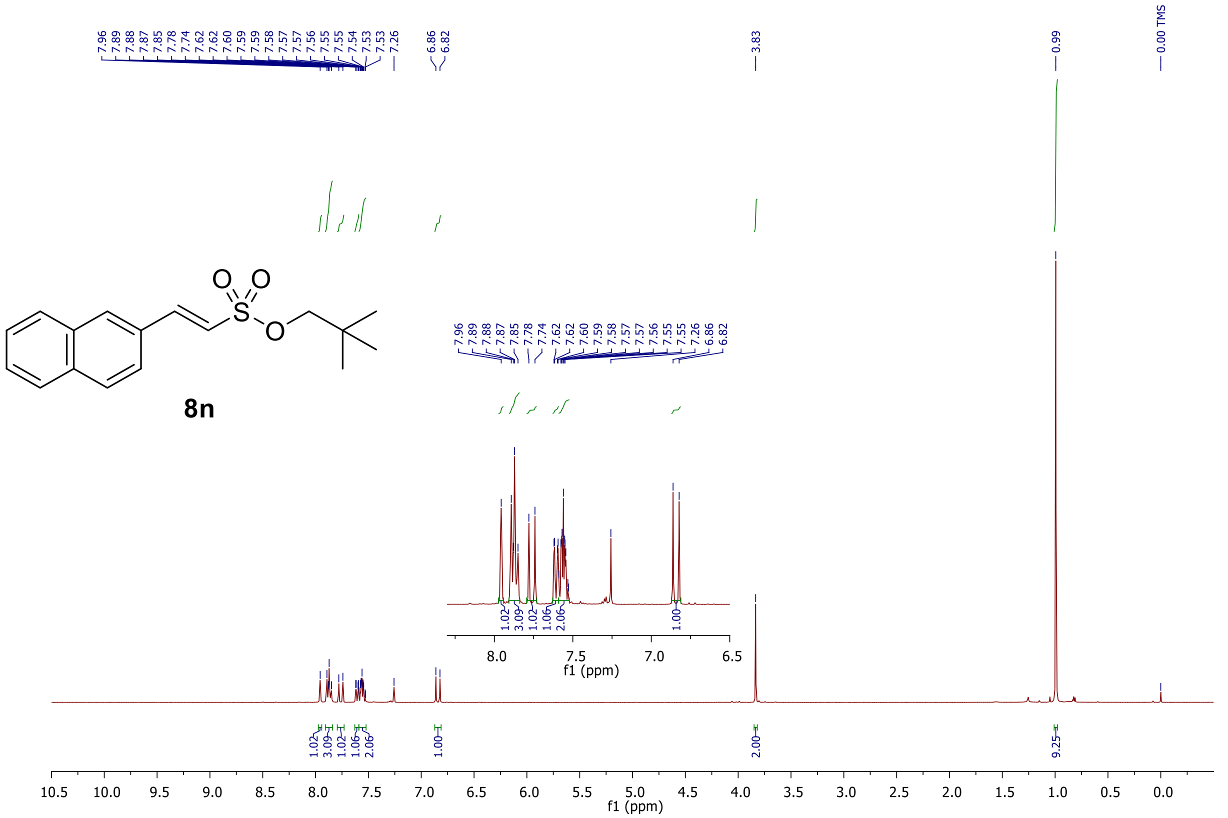

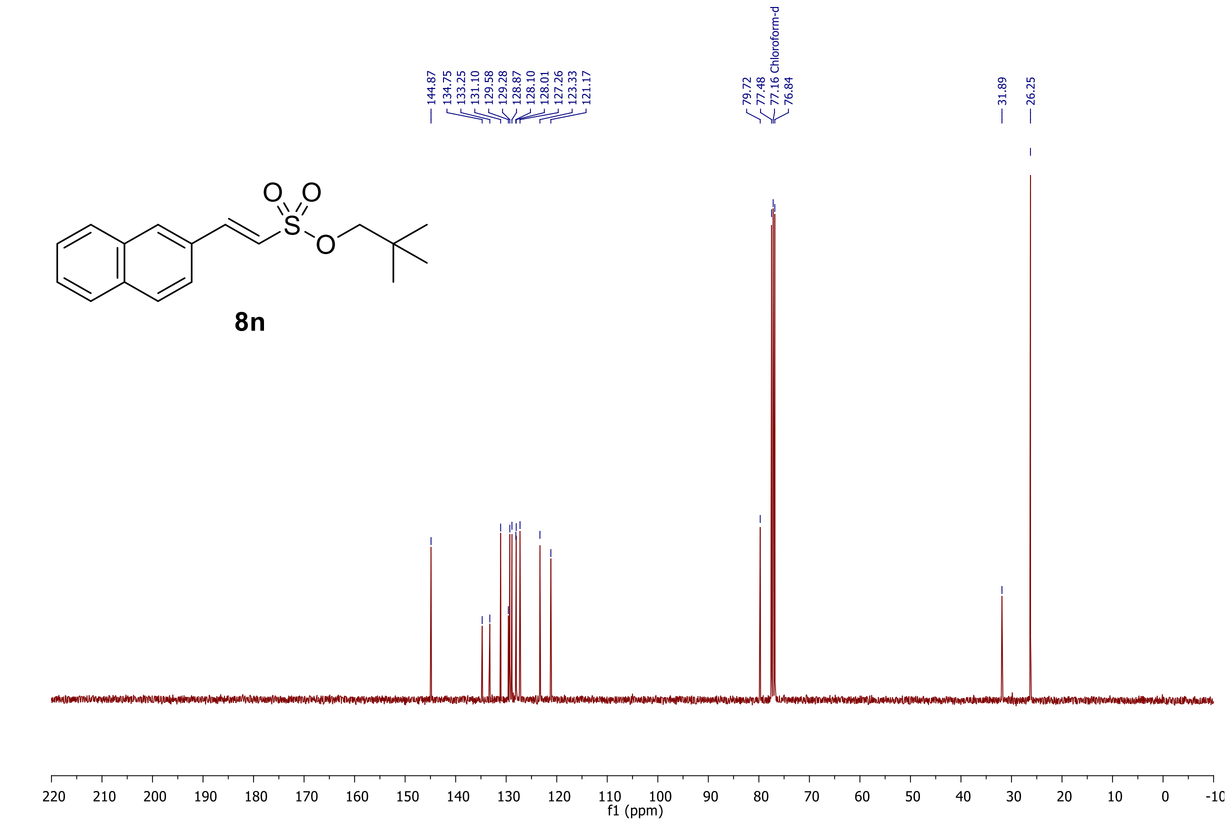

Figure S30: ^{1}H ($CDCl_3$, 400 MHz) and $^{13}C\{^{1}H\}$ ($CDCl_3$, 101 MHz) NMR Spectrum of **8n**.

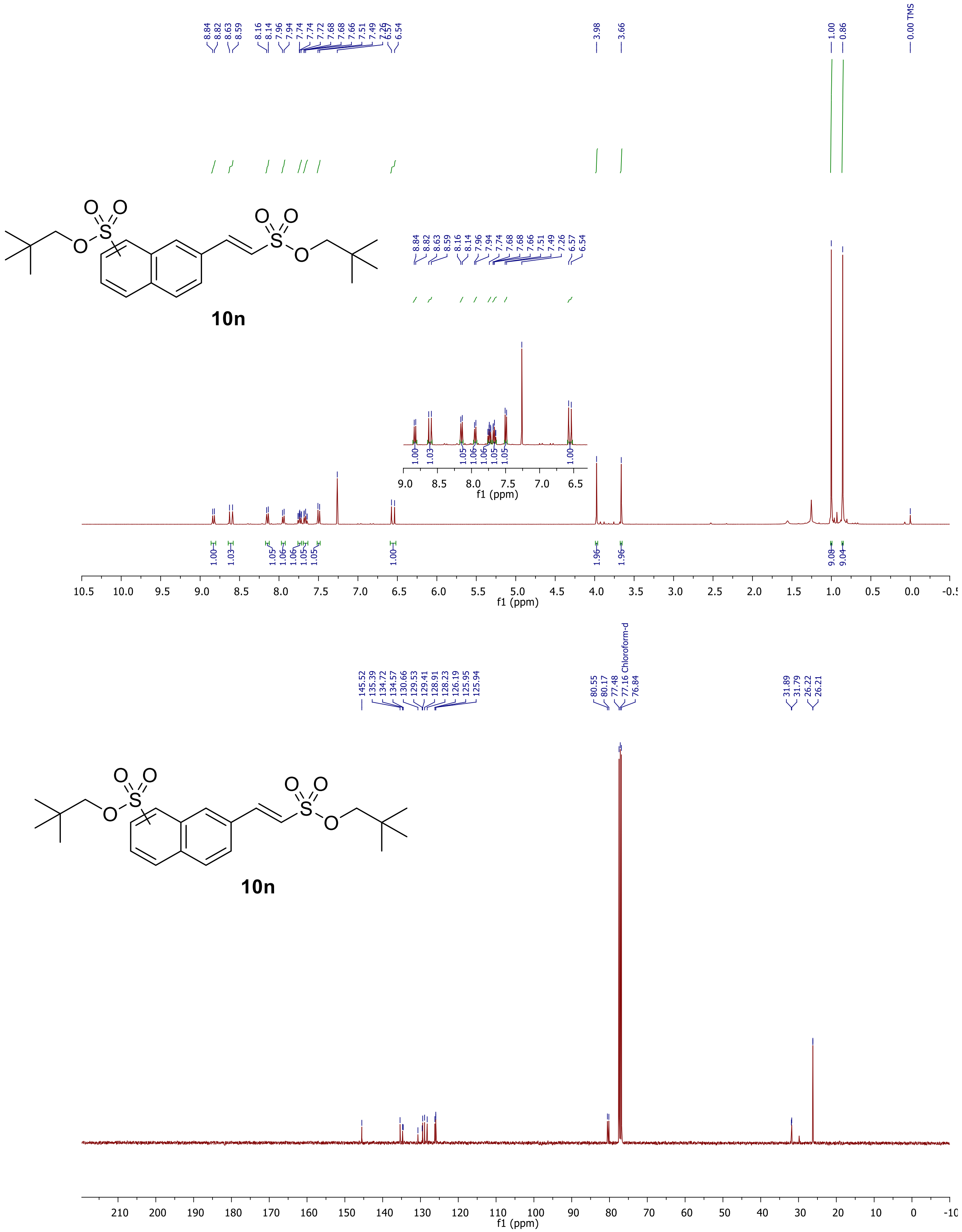

Figure S31: ^{1}H ($CDCl_3$, 400 MHz) and $^{13}C\{^{1}H\}$ ($CDCl_3$, 101 MHz) NMR Spectrum of **10n**.

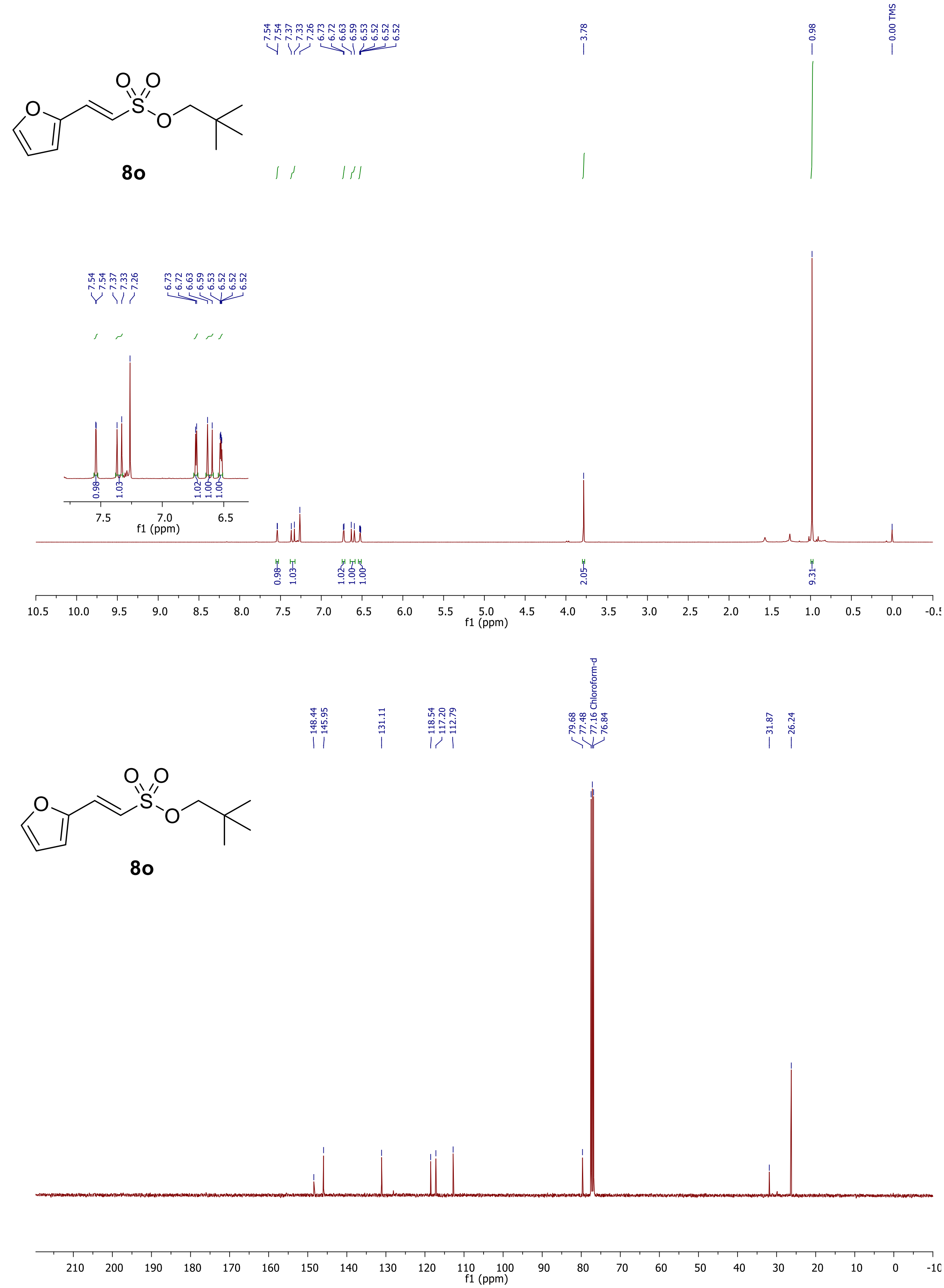

Figure S32: ^{1}H ($CDCl_3$, 400 MHz) and $^{13}C\{^{1}H\}$ ($CDCl_3$, 101 MHz) NMR Spectrum of **8o**.

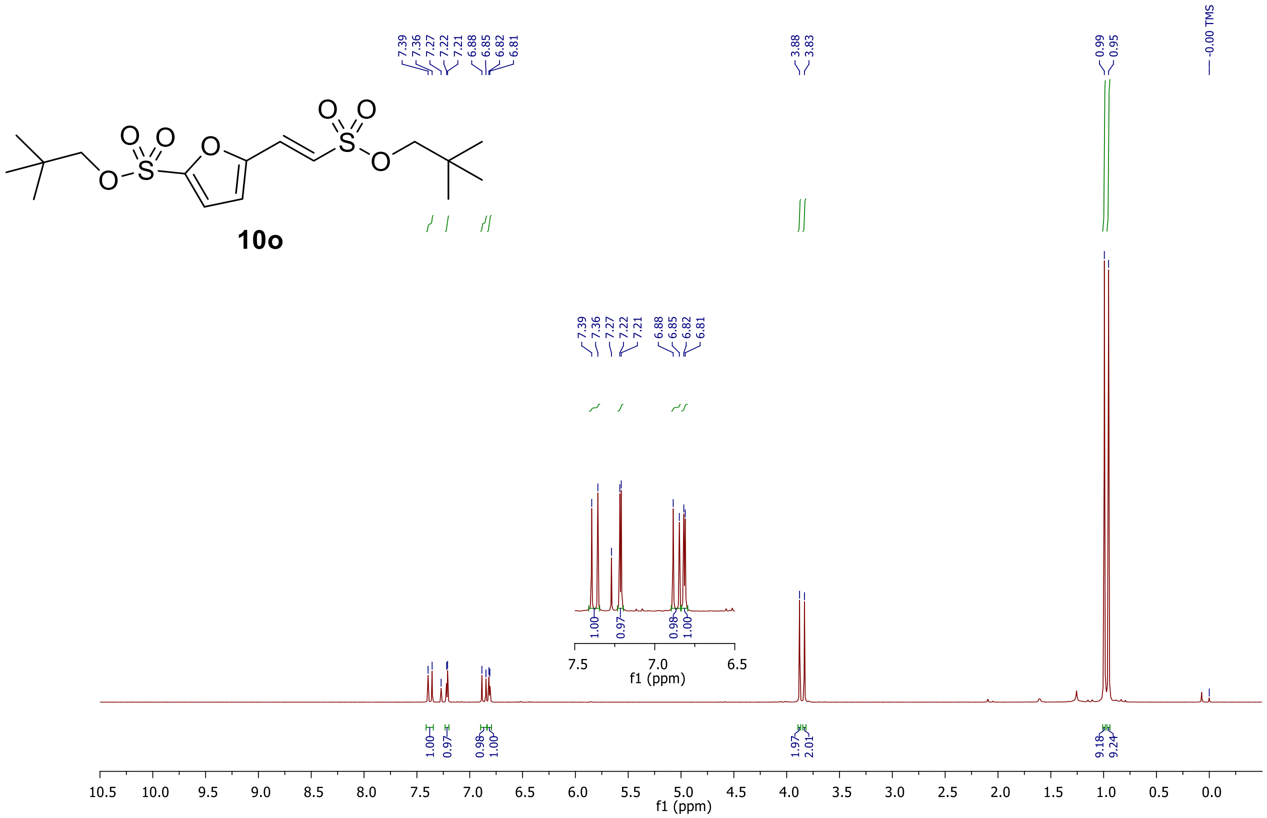

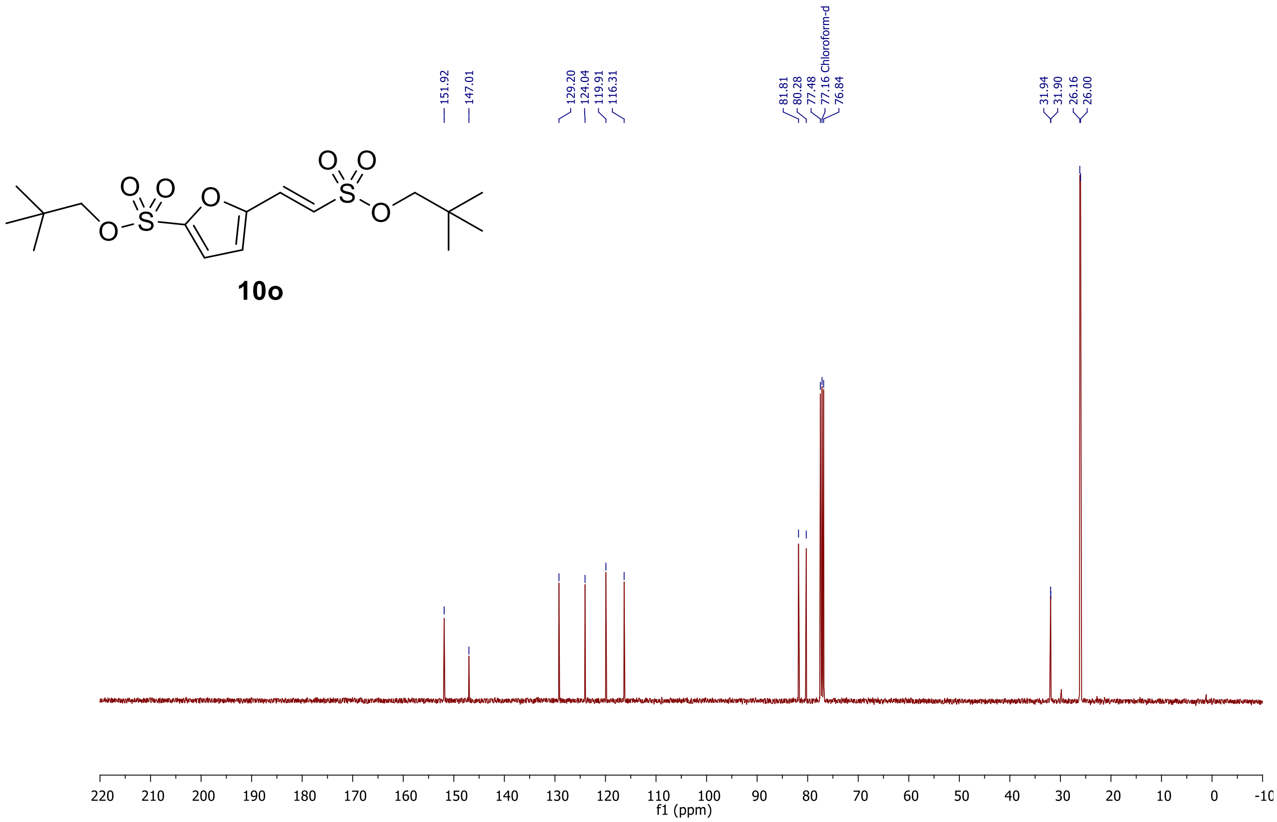

Figure S33: ^{1}H ($CDCl_3$, 400 MHz) and $^{13}C\{^{1}H\}$ ($CDCl_3$, 101 MHz) NMR Spectrum of **10o**.

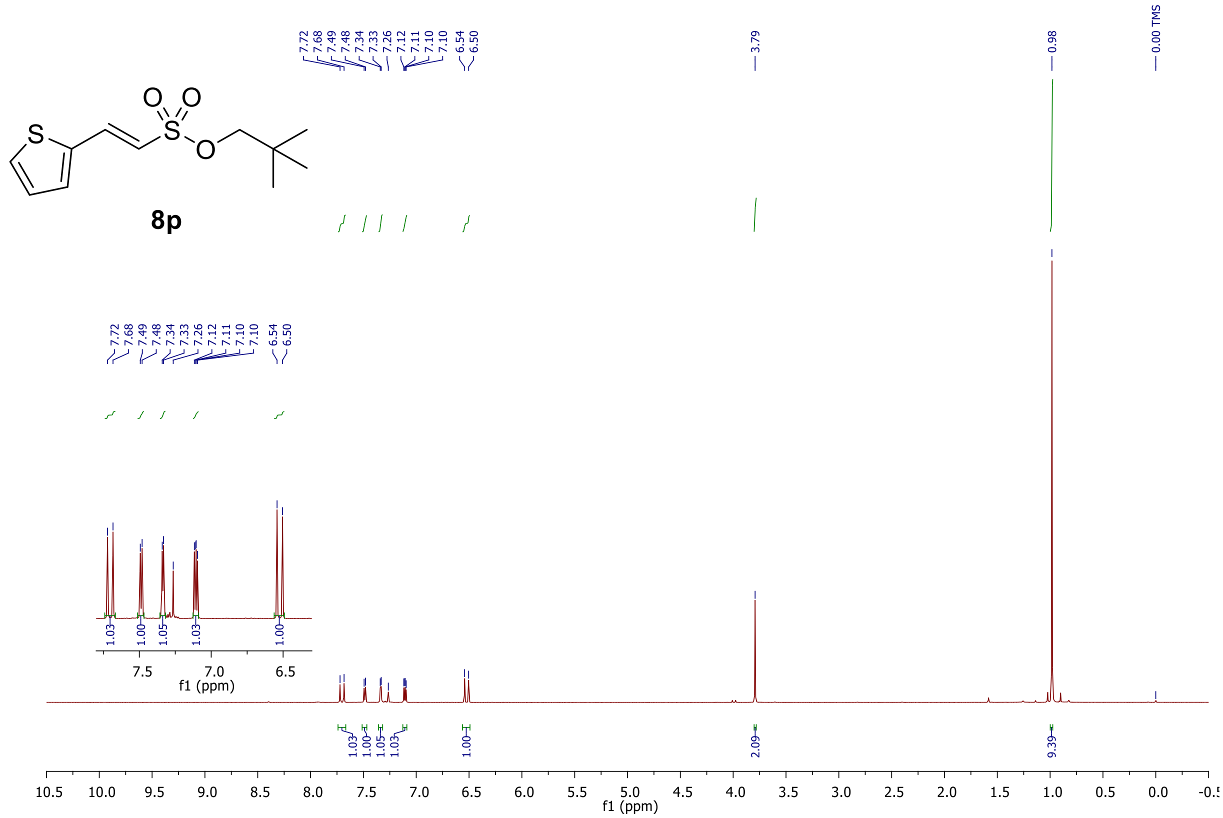

8p

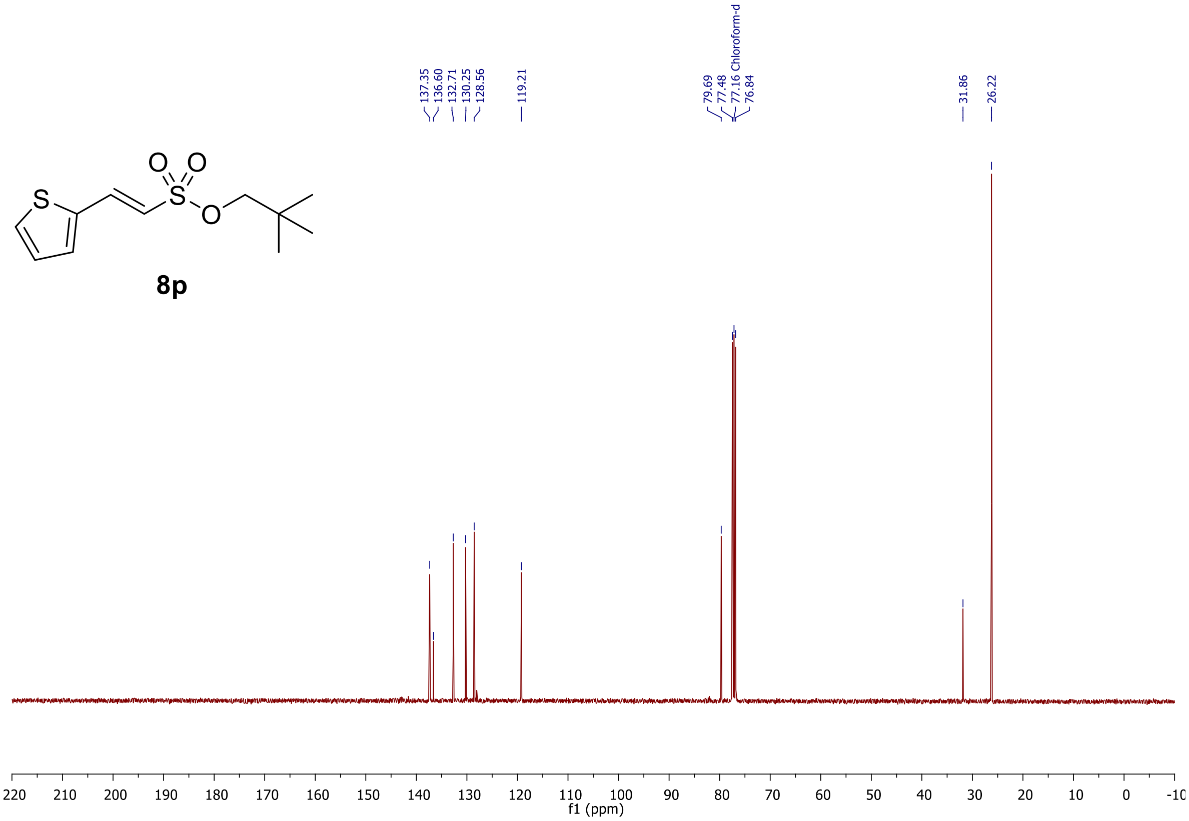

8p

Figure S34: ^{1}H ($CDCl_3$, 400 MHz) and $^{13}C\{^{1}H\}$ ($CDCl_3$, 101 MHz) NMR Spectrum of **8p**.

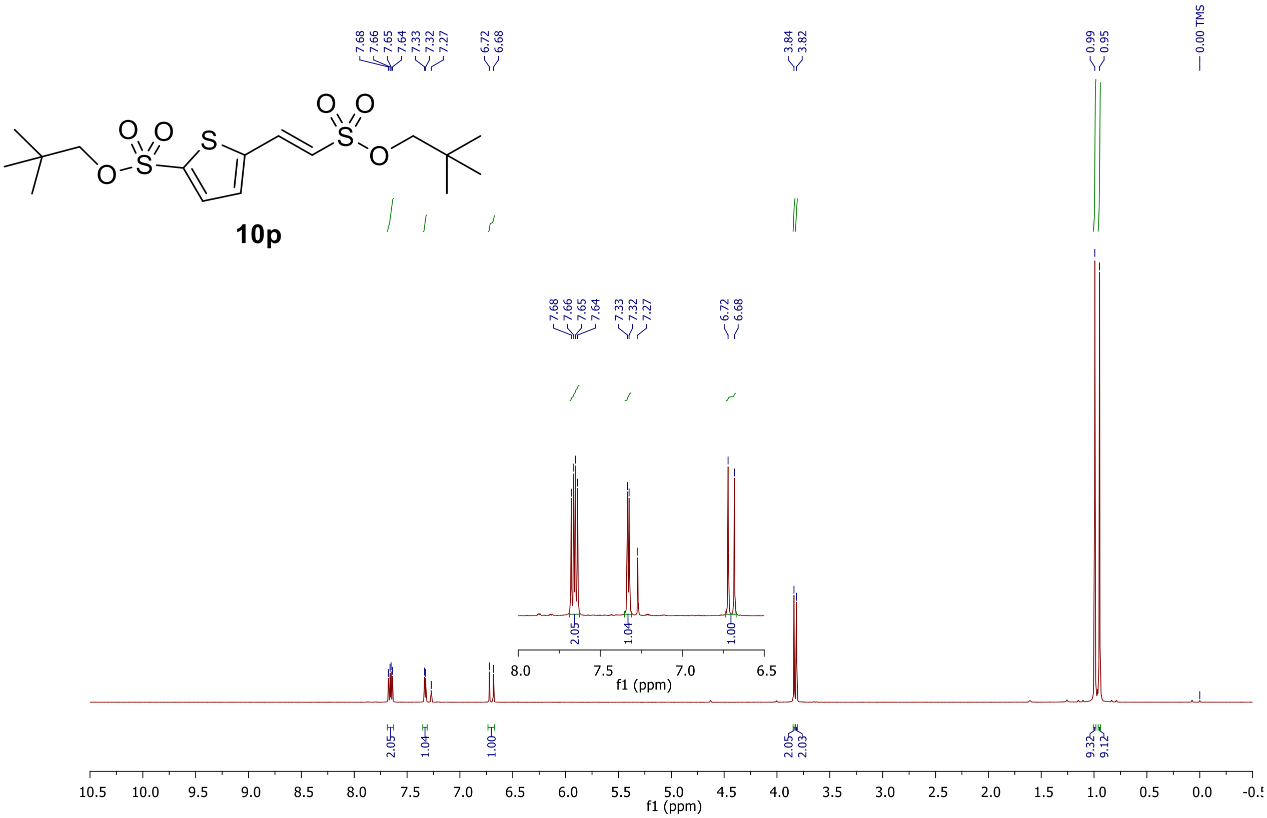

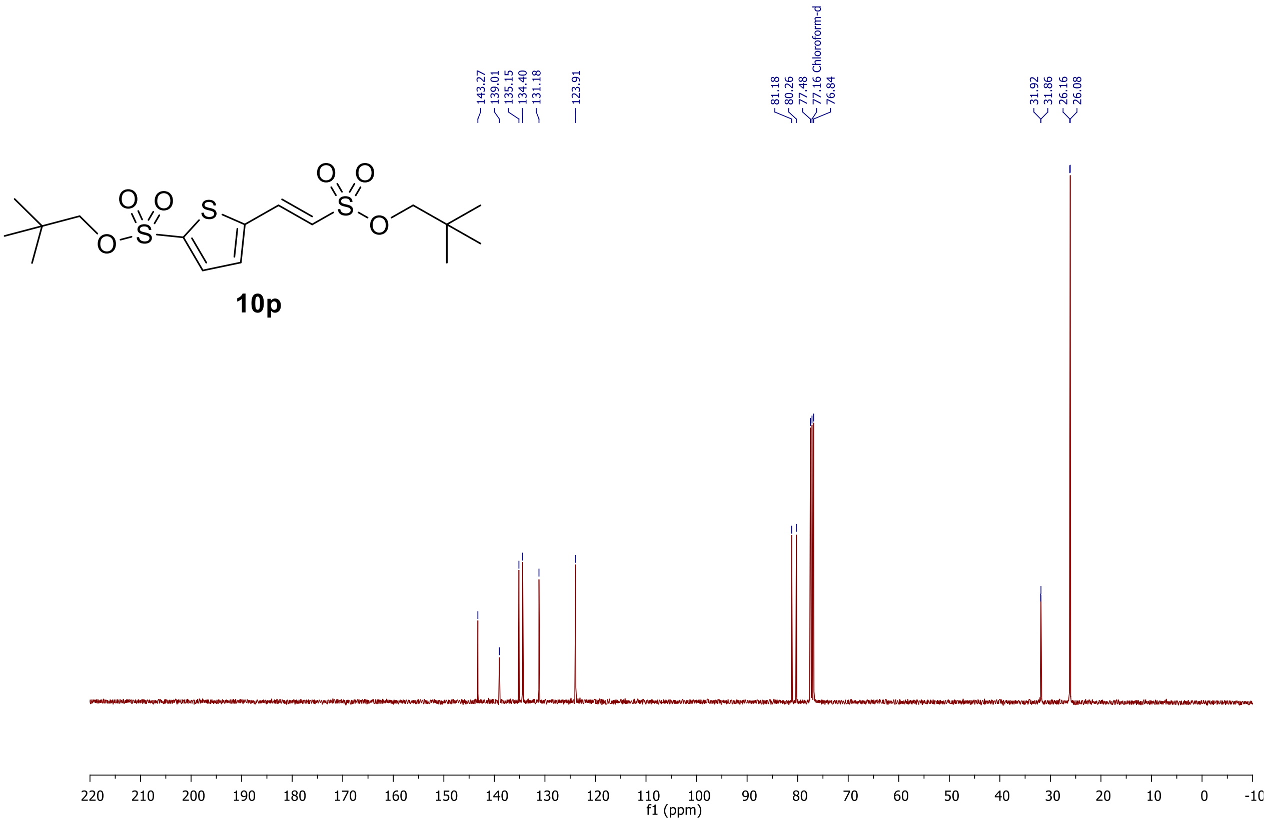

Figure S35: ^{1}H ($CDCl_3$, 400 MHz) and $^{13}C\{^{1}H\}$ ($CDCl_3$, 101 MHz) NMR Spectrum of **10p**.

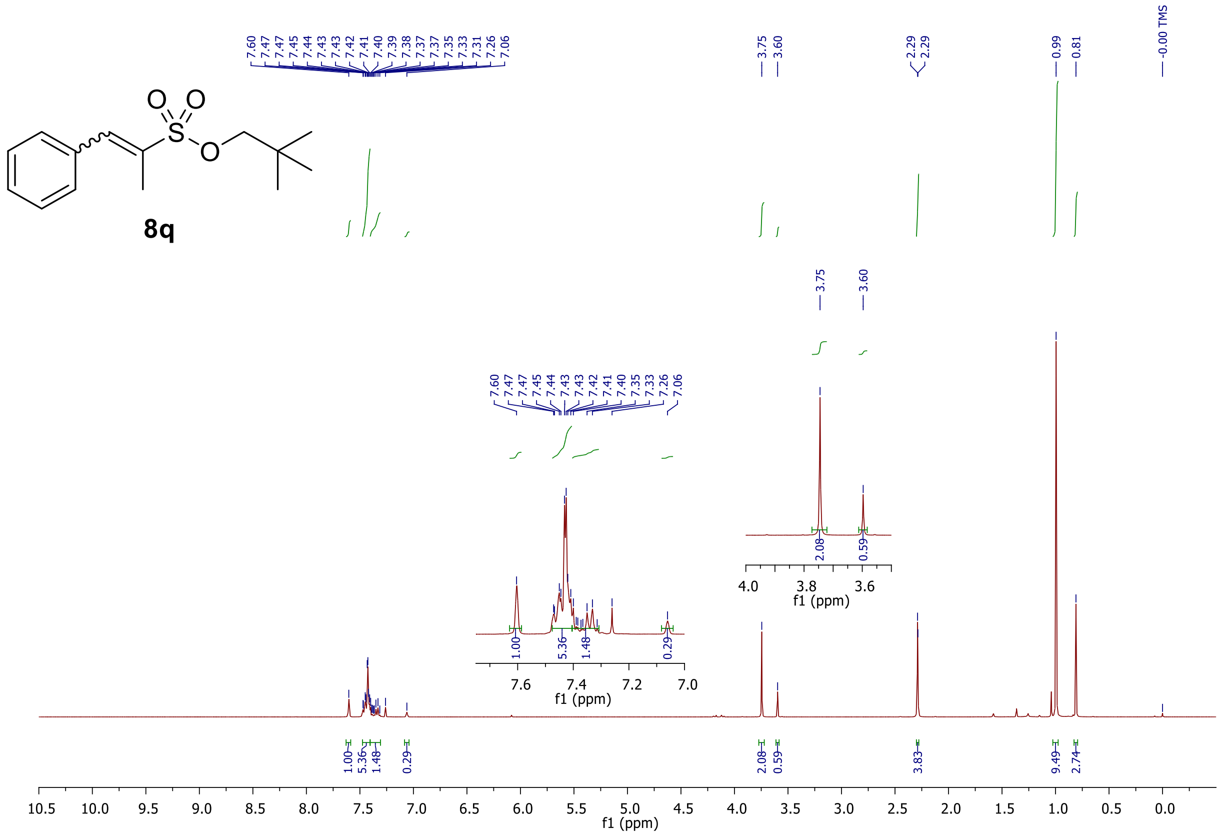

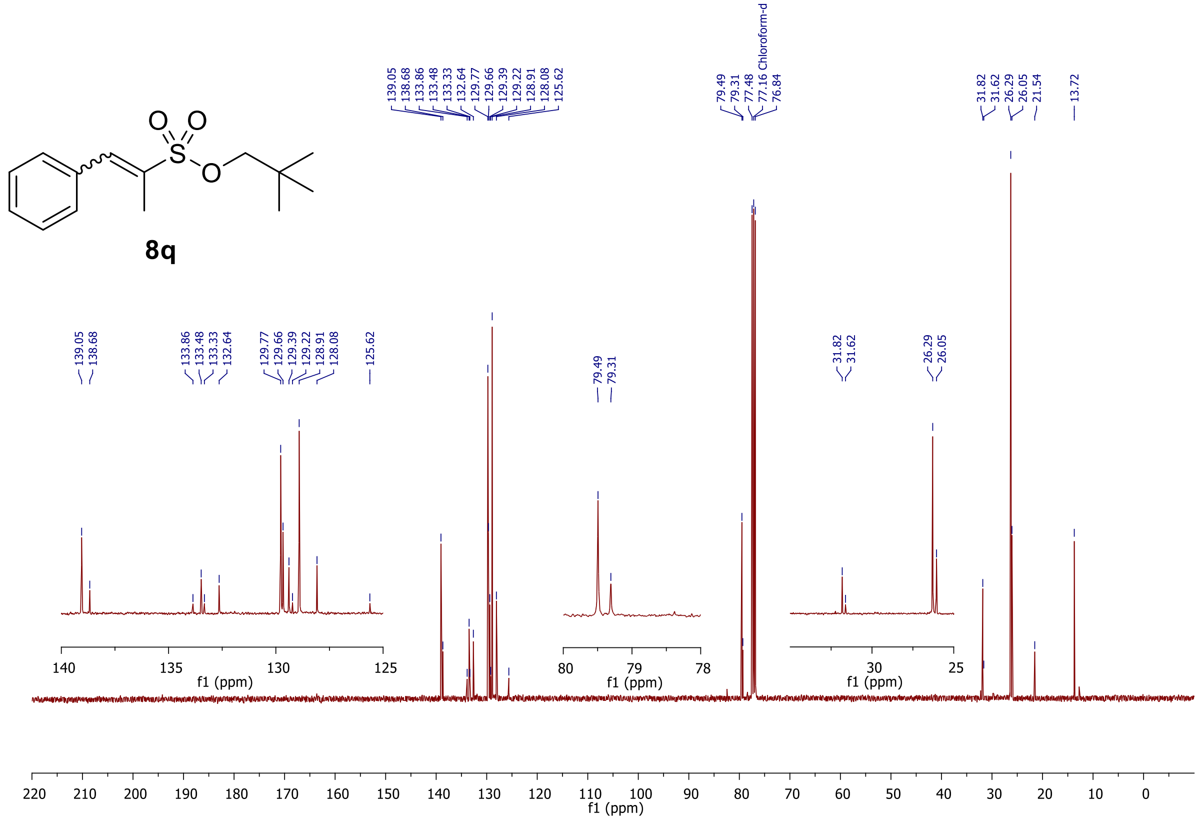

Figure S36: ^{1}H ($CDCl_3$, 400 MHz) and $^{13}C\{^{1}H\}$ ($CDCl_3$, 101 MHz) NMR Spectrum of **8q**.

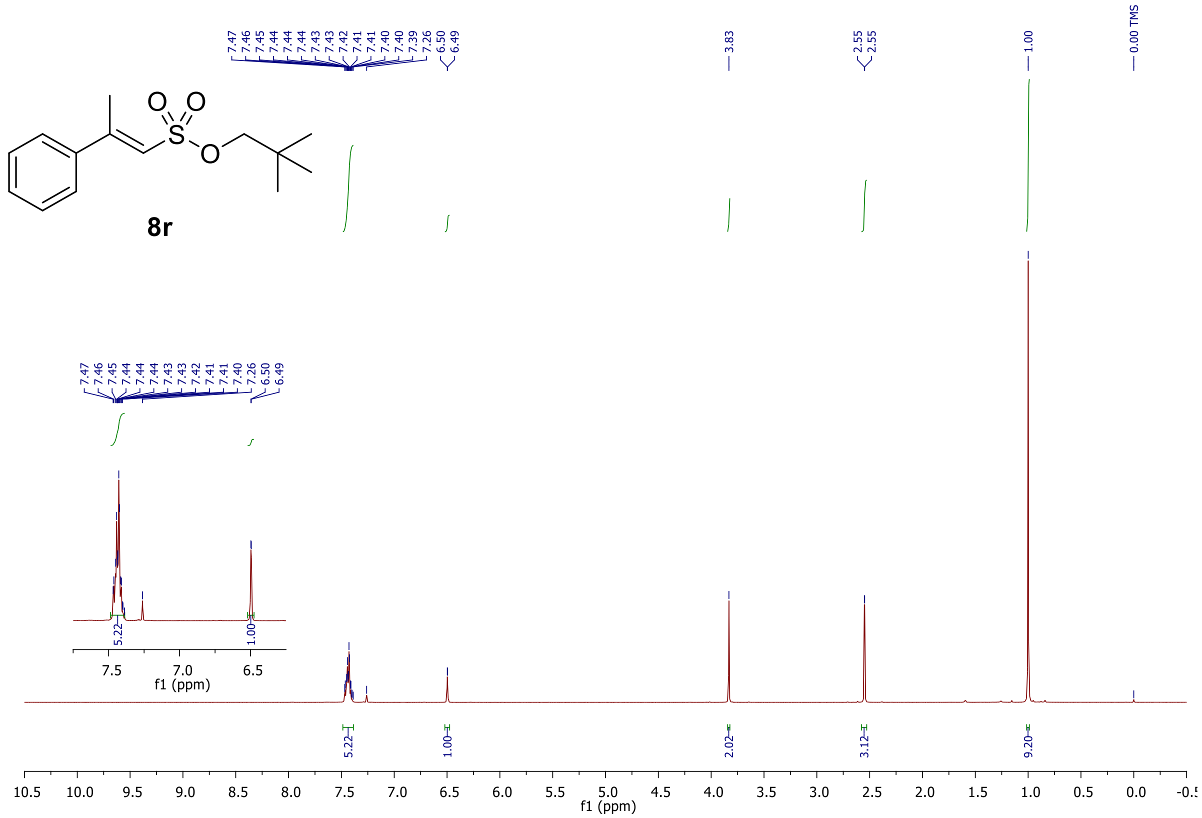

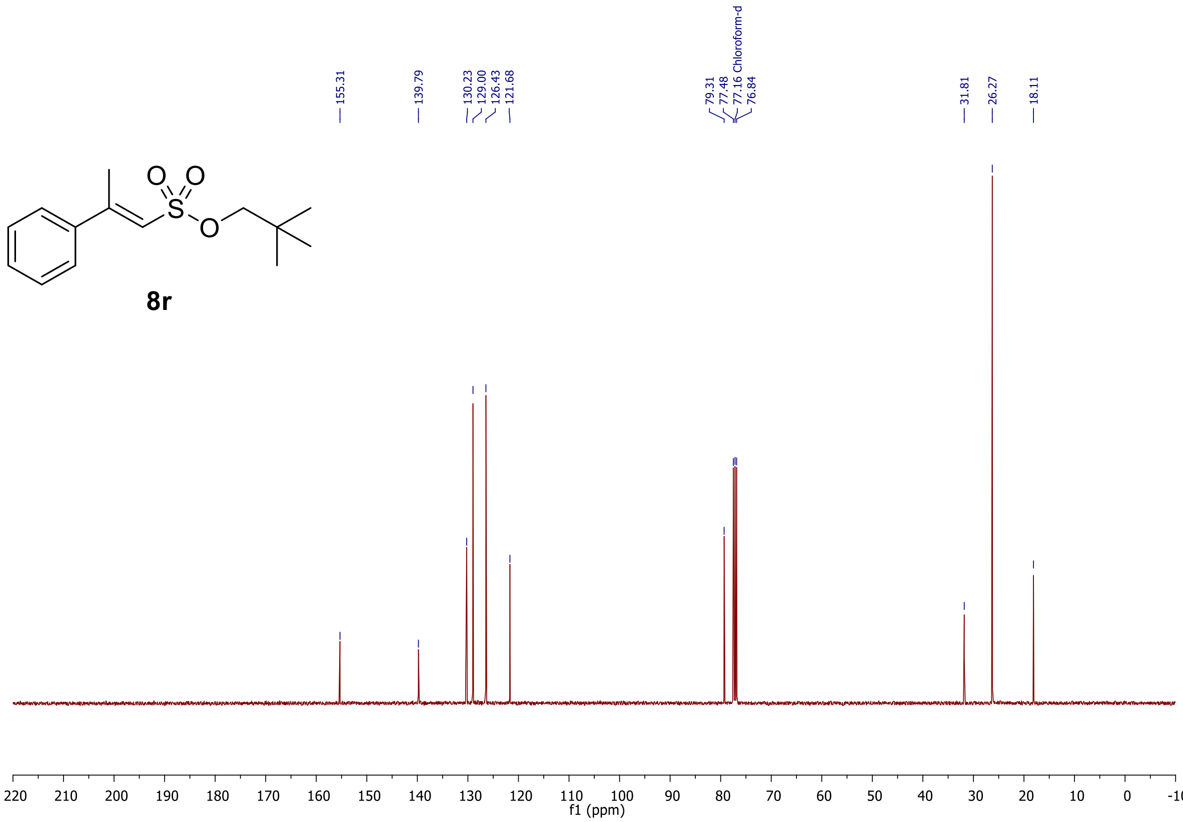

Figure S37: ^{1}H ($CDCl_3$, 400 MHz) and $^{13}C\{^{1}H\}$ ($CDCl_3$, 101 MHz) NMR Spectrum of **8r**.

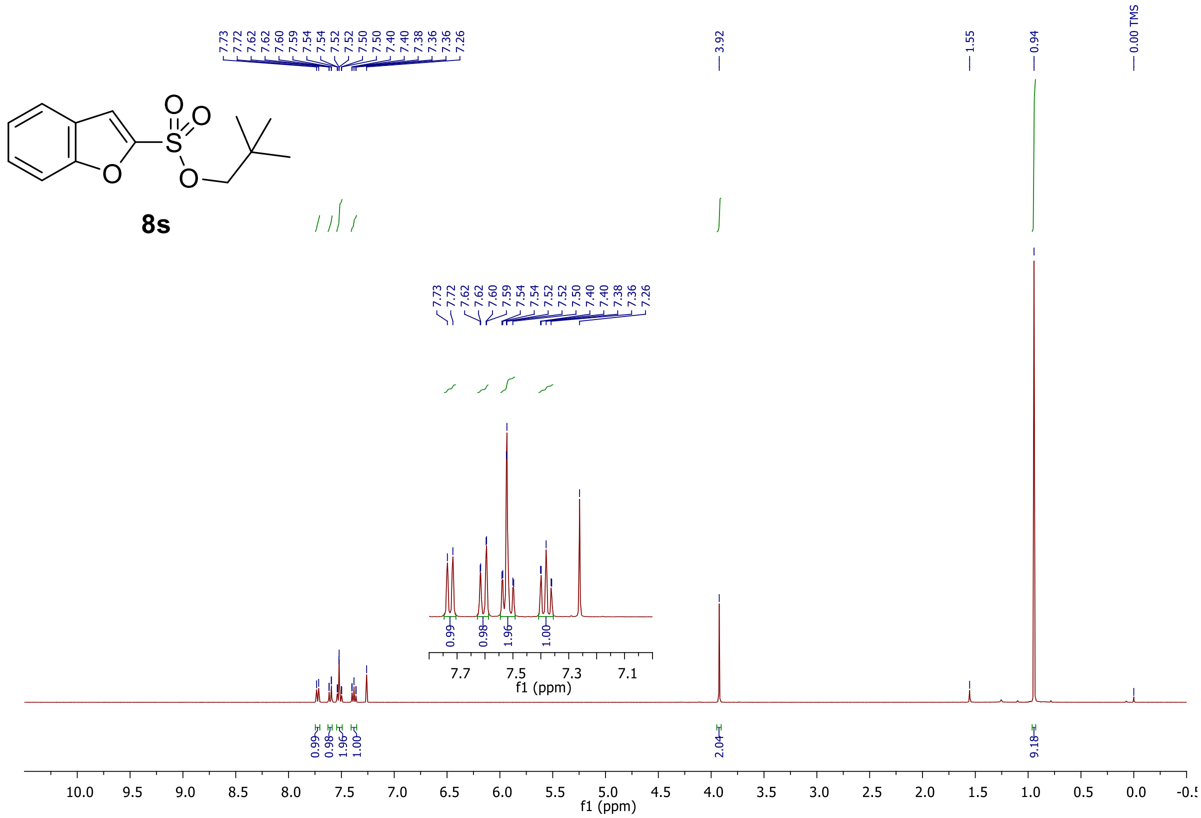

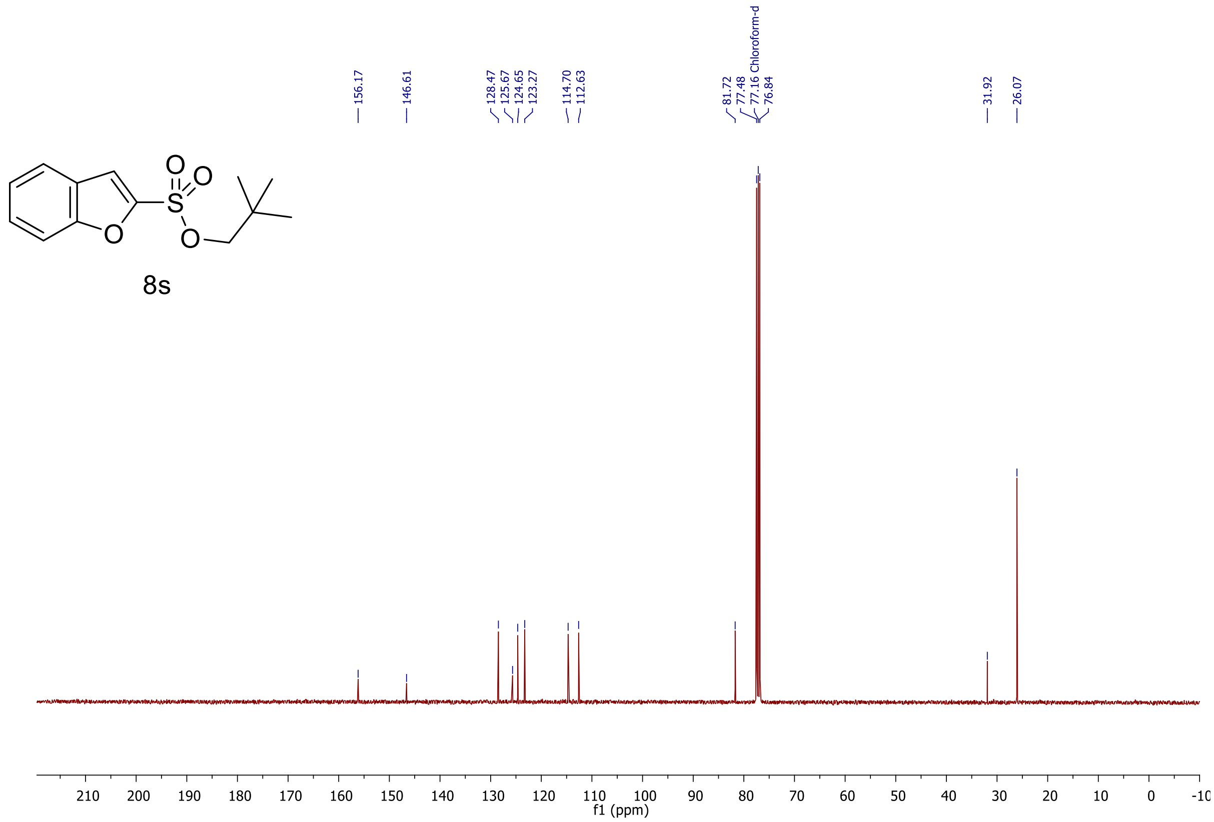

Figure S38: ^{1}H ($CDCl_3$, 400 MHz) and $^{13}C\{^{1}H\}$ ($CDCl_3$, 101 MHz) NMR Spectrum of **8s**.

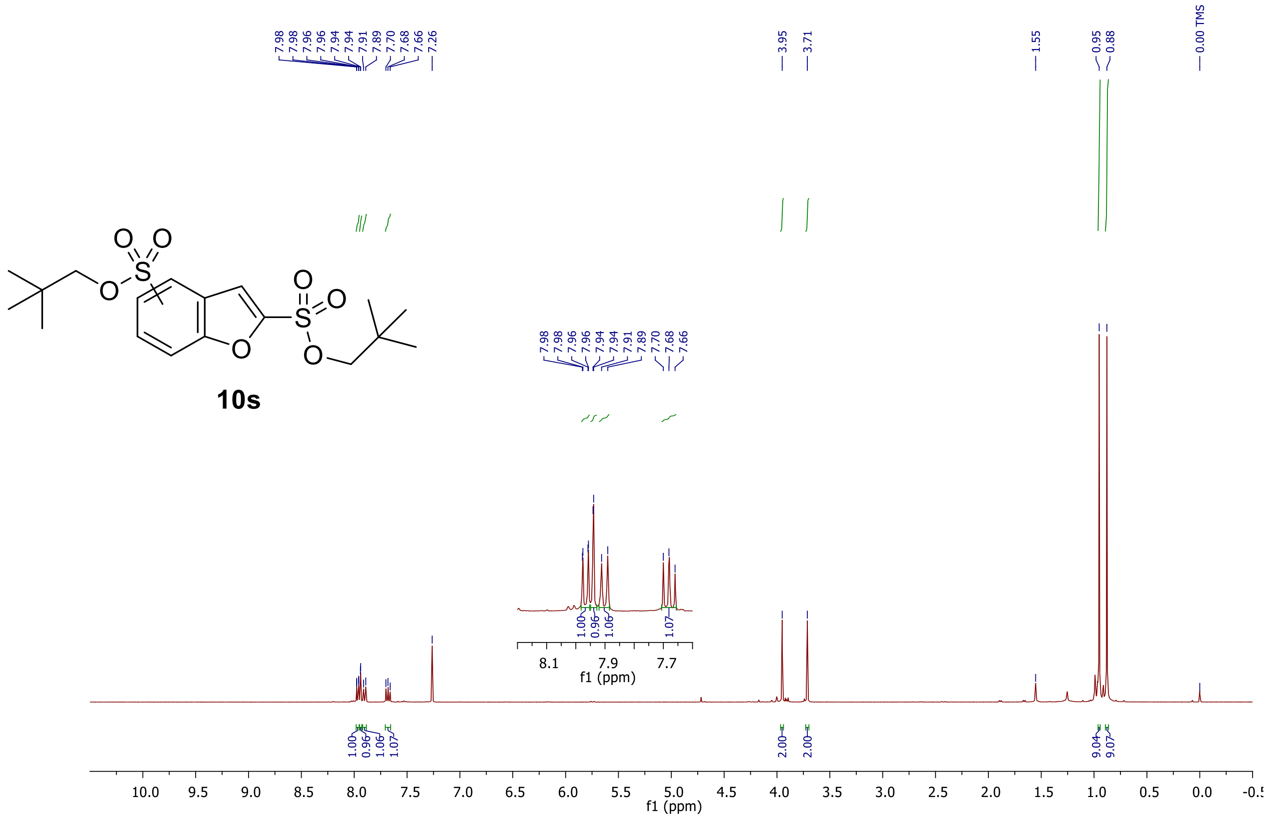

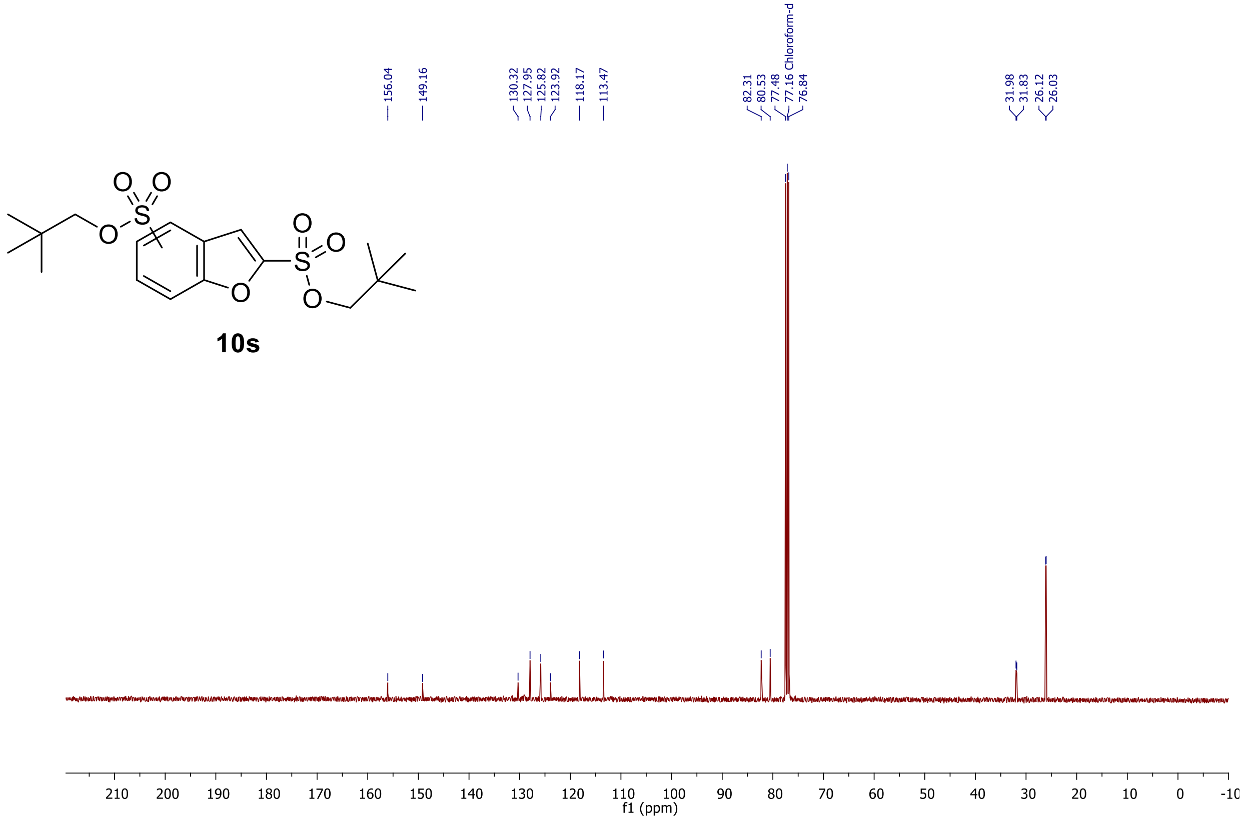

Figure S39: ^{1}H ($CDCl_3$, 400 MHz) and $^{13}C\{^{1}H\}$ ($CDCl_3$, 101 MHz) NMR Spectrum of **10s**.

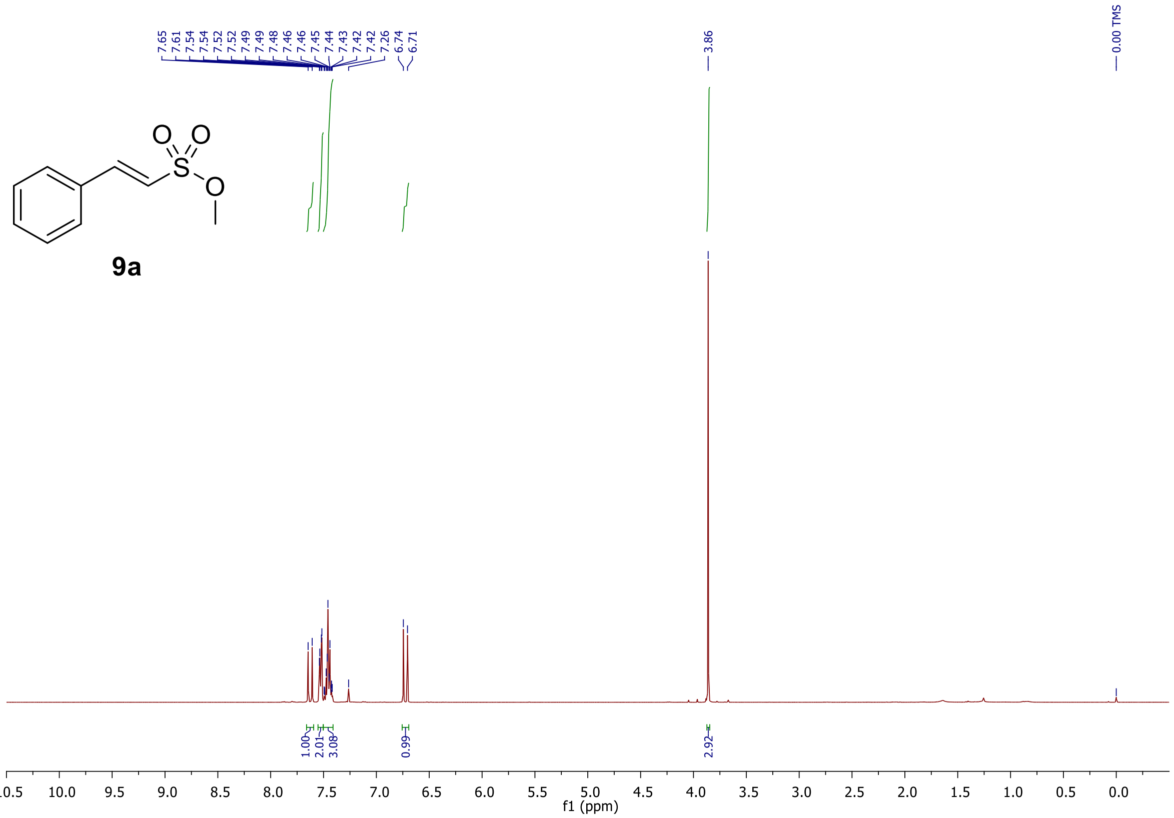

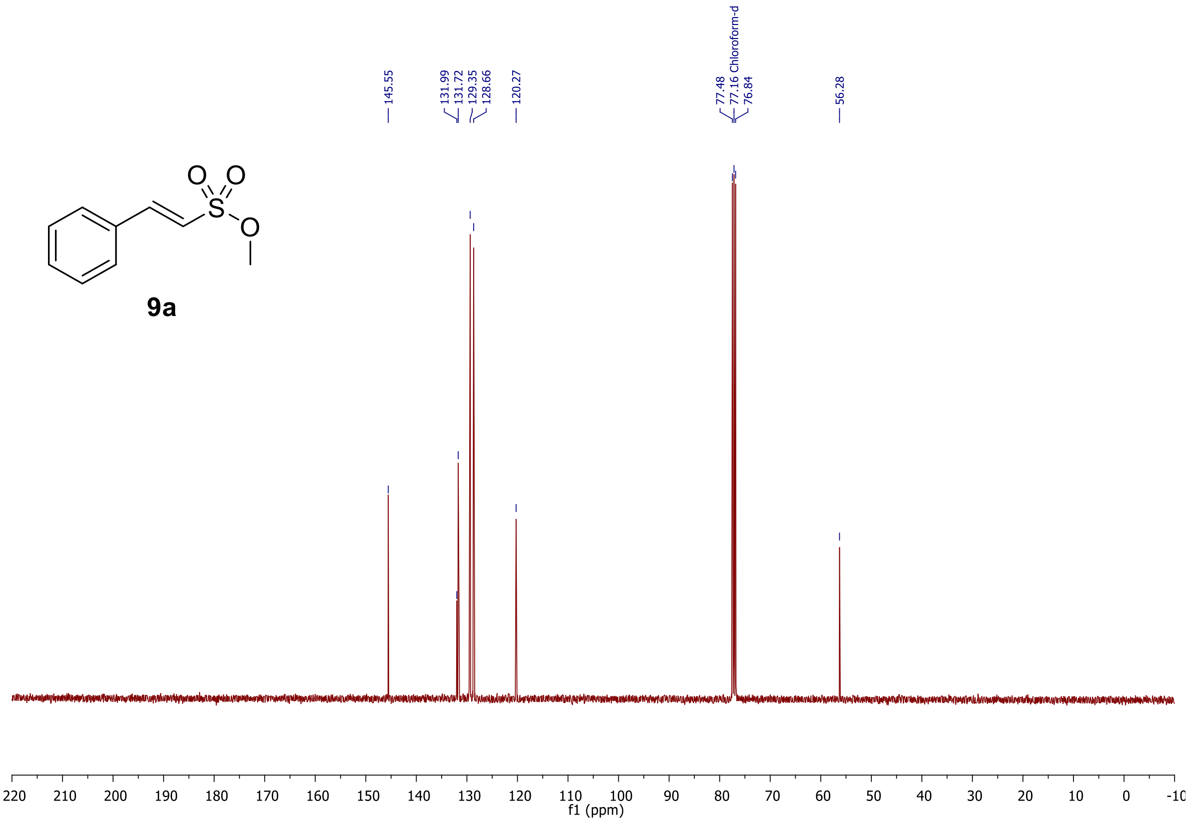

Figure S40: ^{1}H ($CDCl_3$, 400 MHz) and $^{13}C\{^{1}H\}$ ($CDCl_3$, 101 MHz) NMR Spectrum of **9a**.

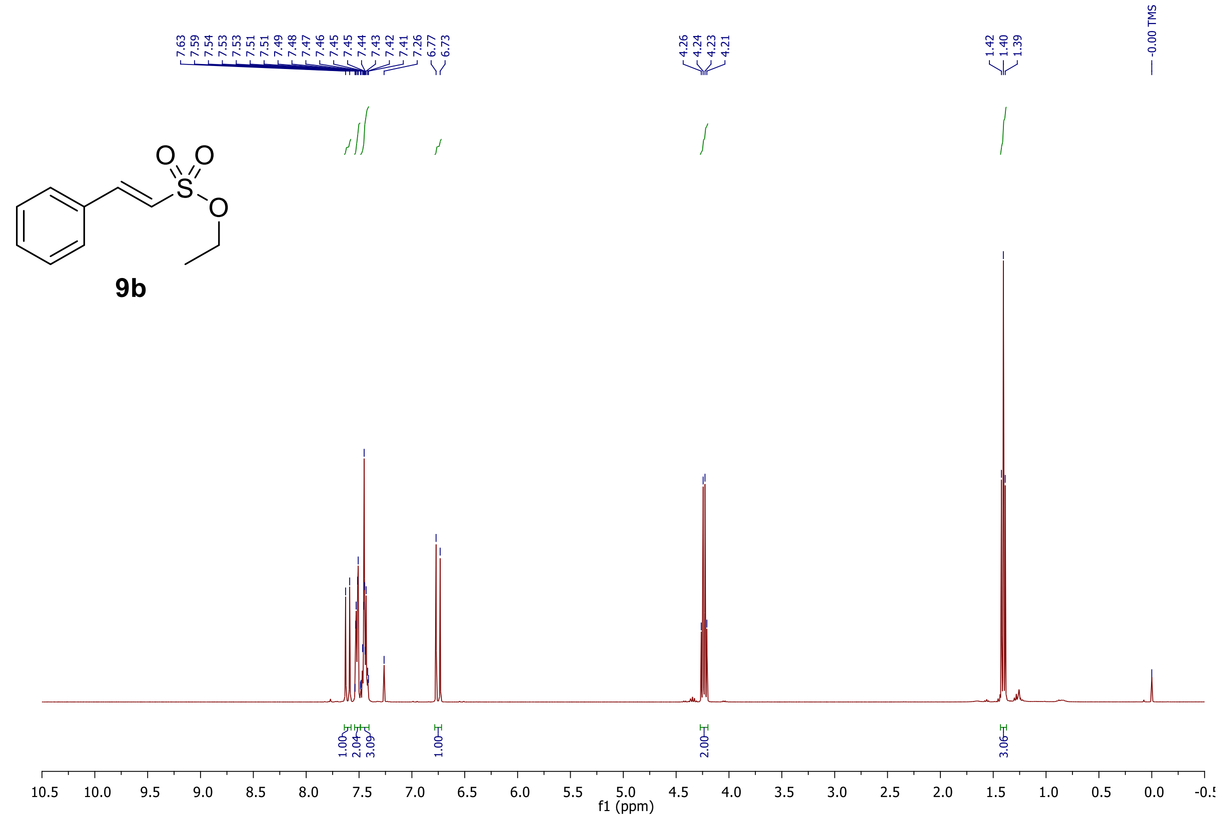

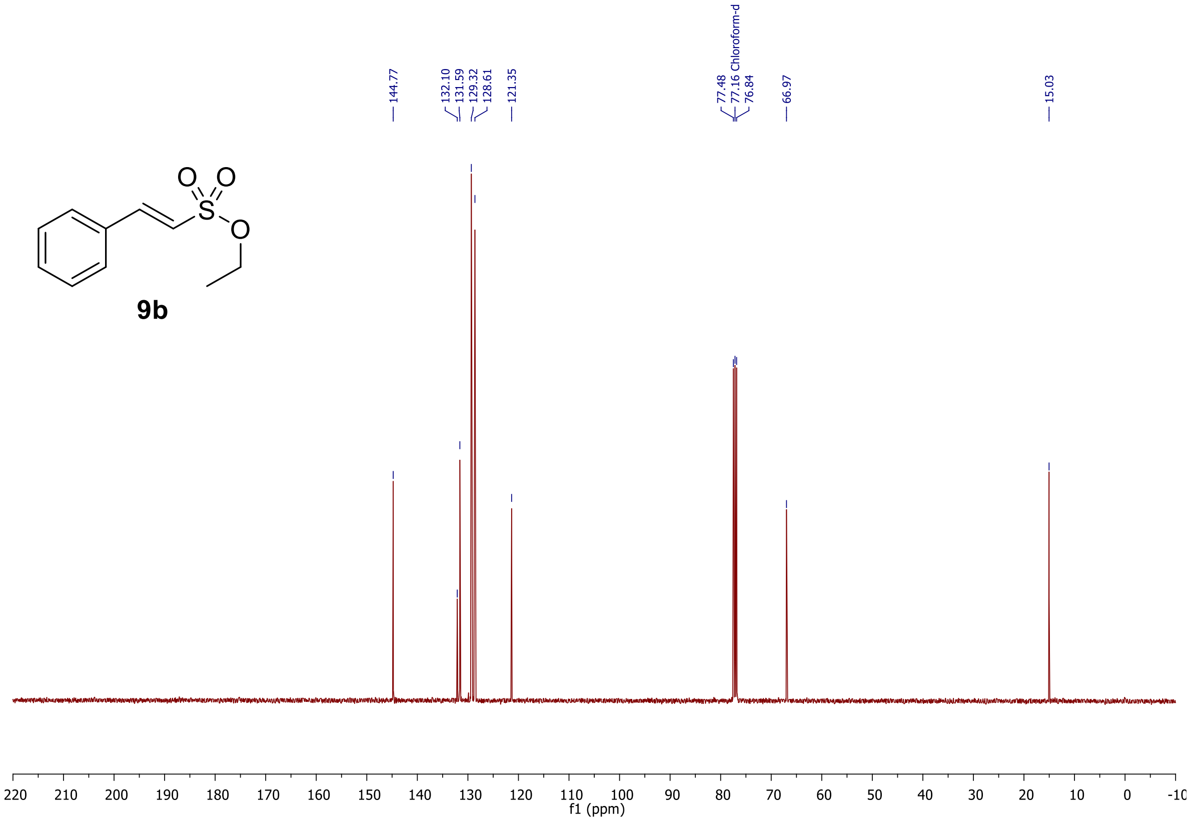

Figure S41: ^{1}H ($CDCl_3$, 400 MHz) and $^{13}C\{^{1}H\}$ ($CDCl_3$, 101 MHz) NMR Spectrum of **9b**.

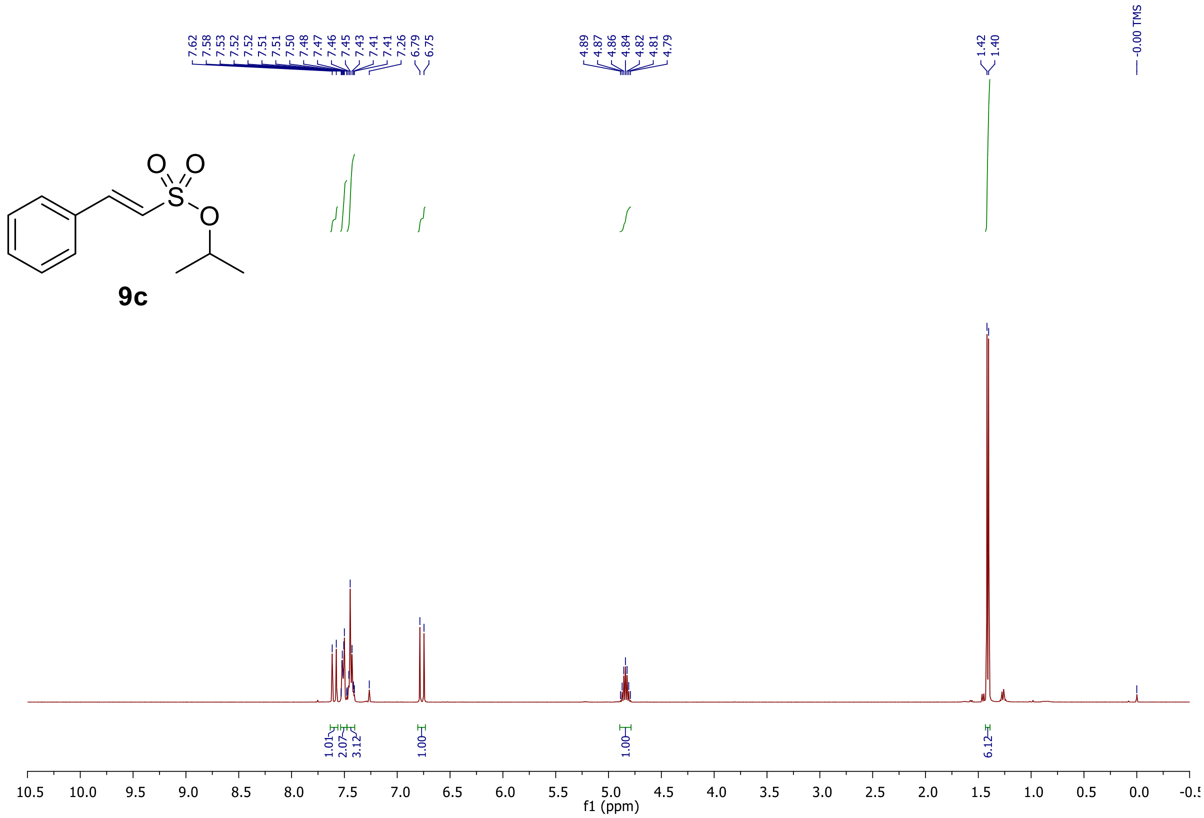

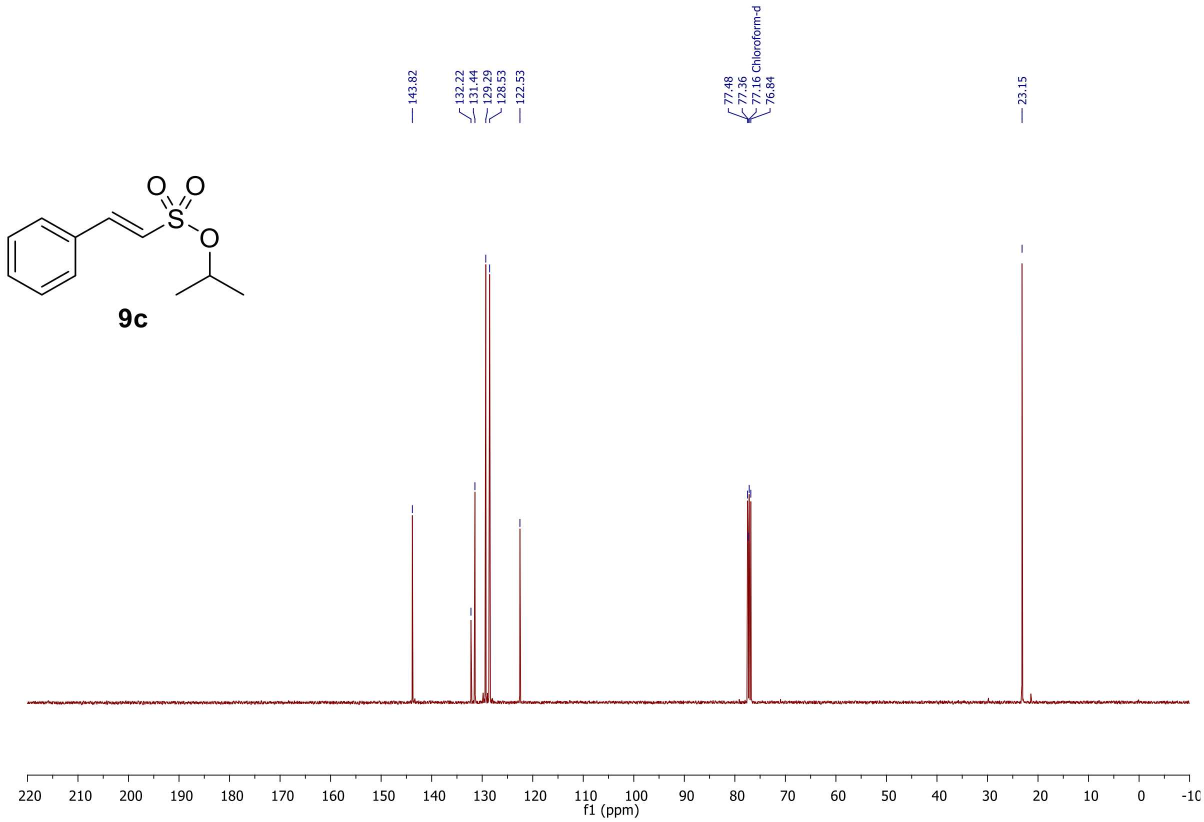

Figure S42: ^{1}H ($CDCl_3$, 400 MHz) and $^{13}C\{^{1}H\}$ ($CDCl_3$, 101 MHz) NMR Spectrum of **9c**.

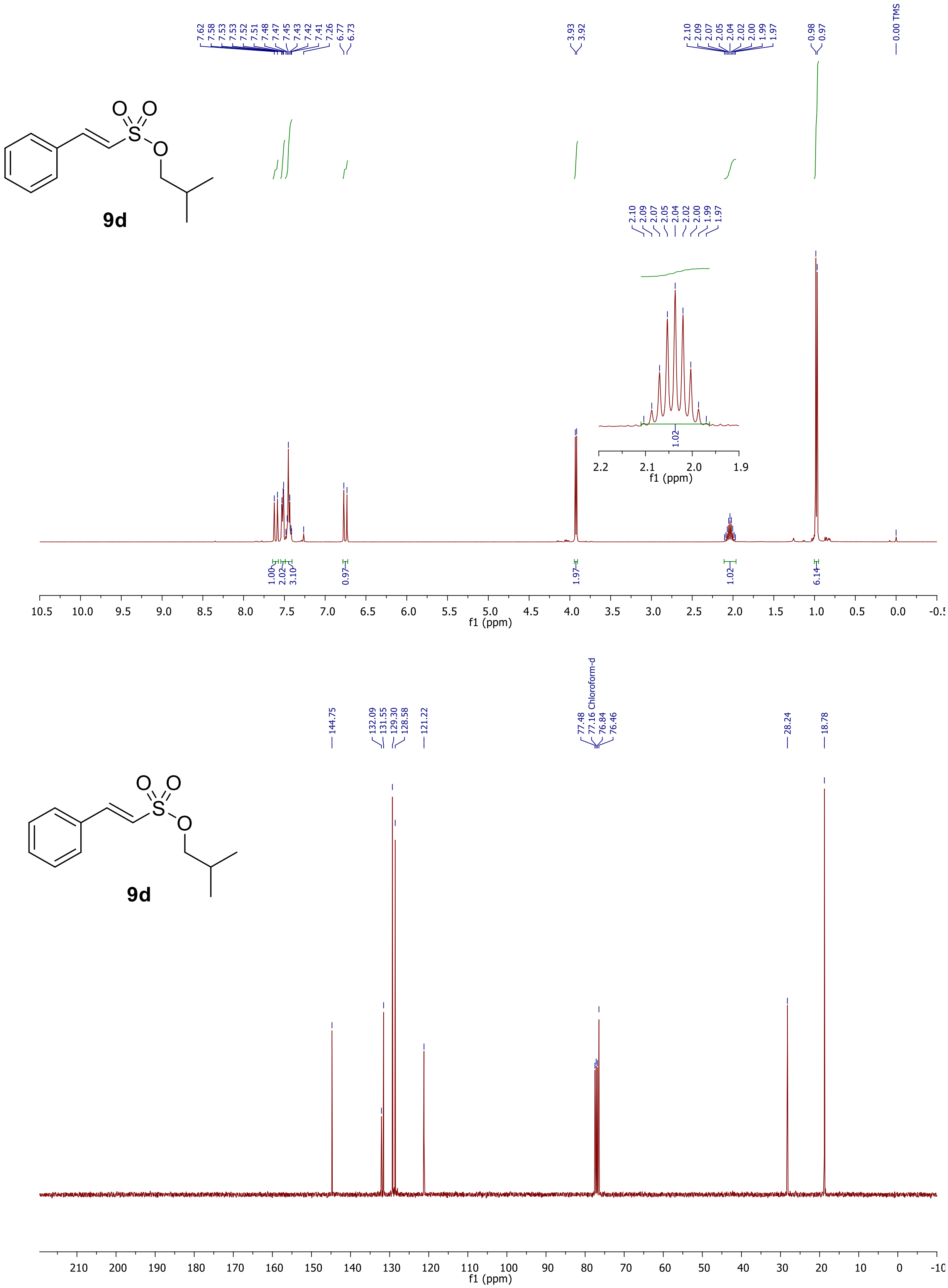

Figure S43: ^{1}H ($CDCl_3$, 400 MHz) and $^{13}C\{^1H\}$ ($CDCl_3$, 101 MHz) NMR Spectrum of **9d**.

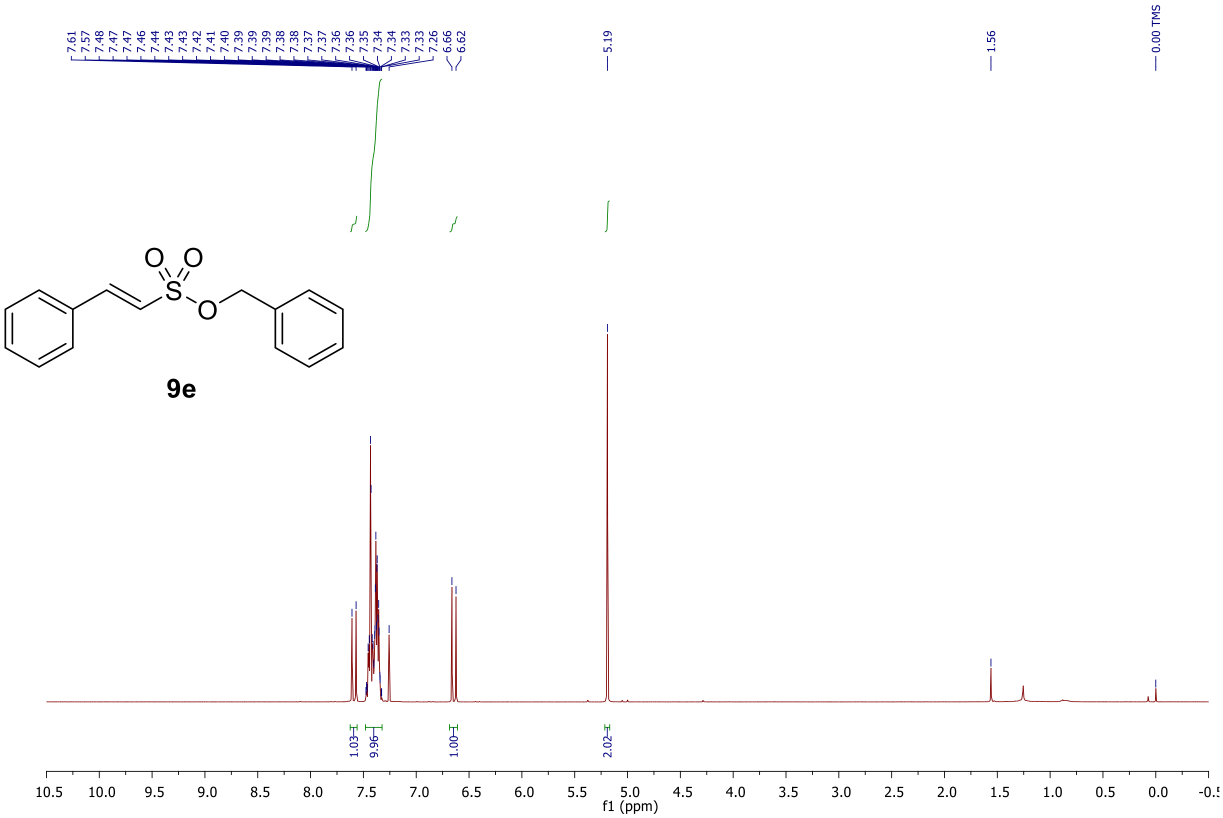

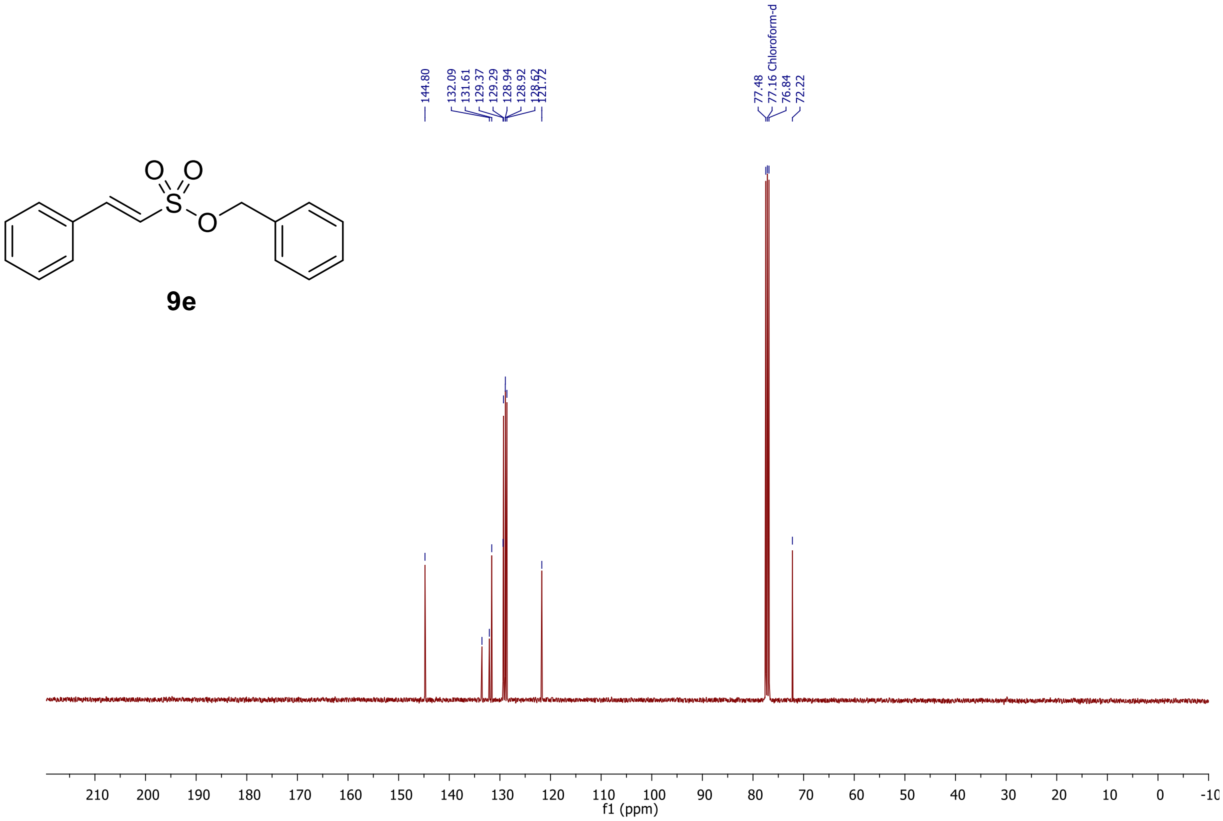

Figure S44: ^{1}H ($CDCl_3$, 400 MHz) and $^{13}C\{^{1}H\}$ ($CDCl_3$, 101 MHz) NMR Spectrum of **9e**.

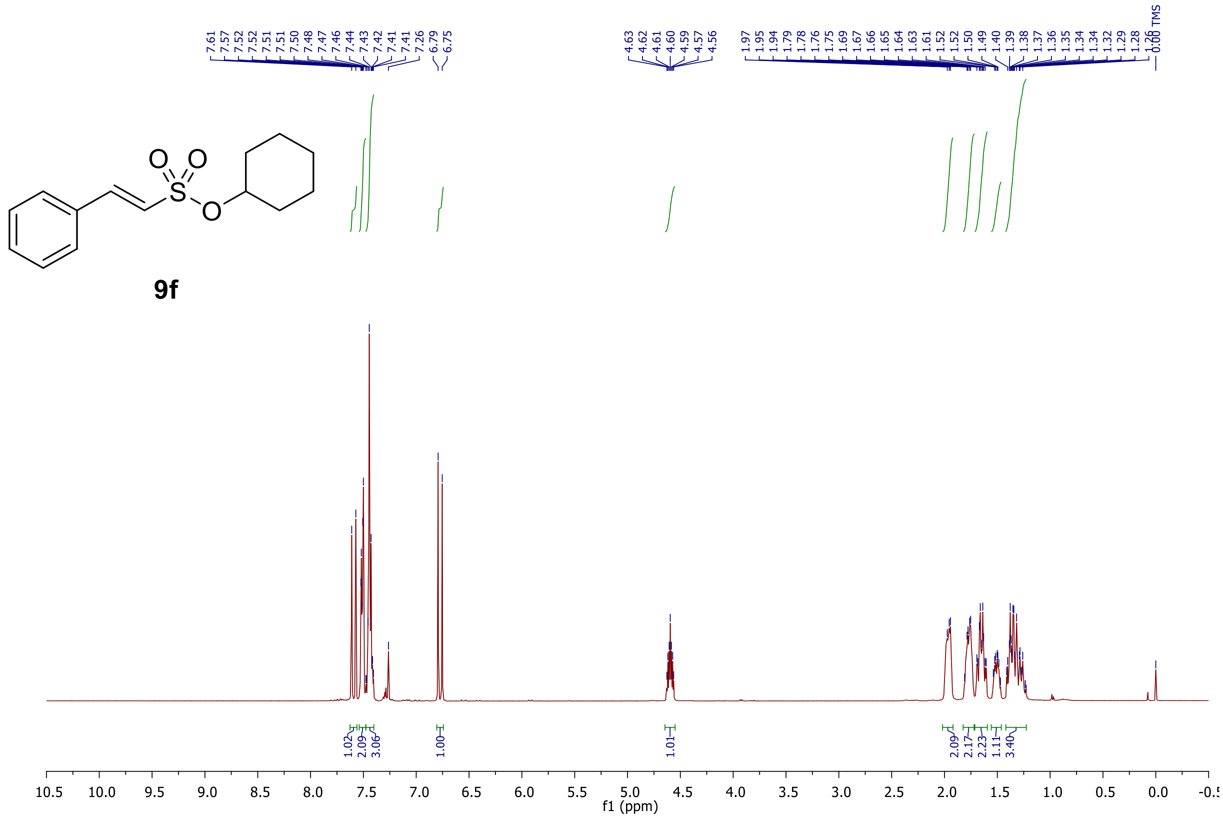

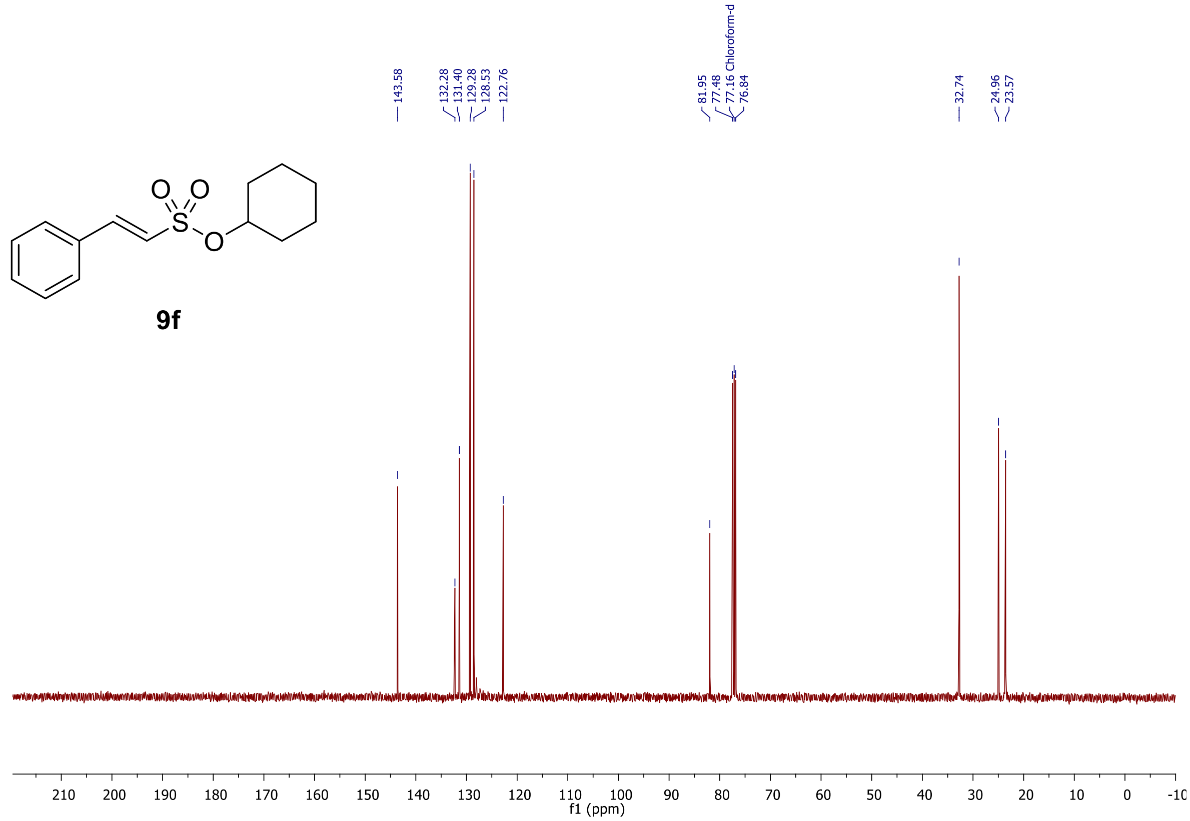

Figure S45: ^{1}H ($CDCl_3$, 400 MHz) and $^{13}C\{^{1}H\}$ ($CDCl_3$, 101 MHz) NMR Spectrum of **9f**.

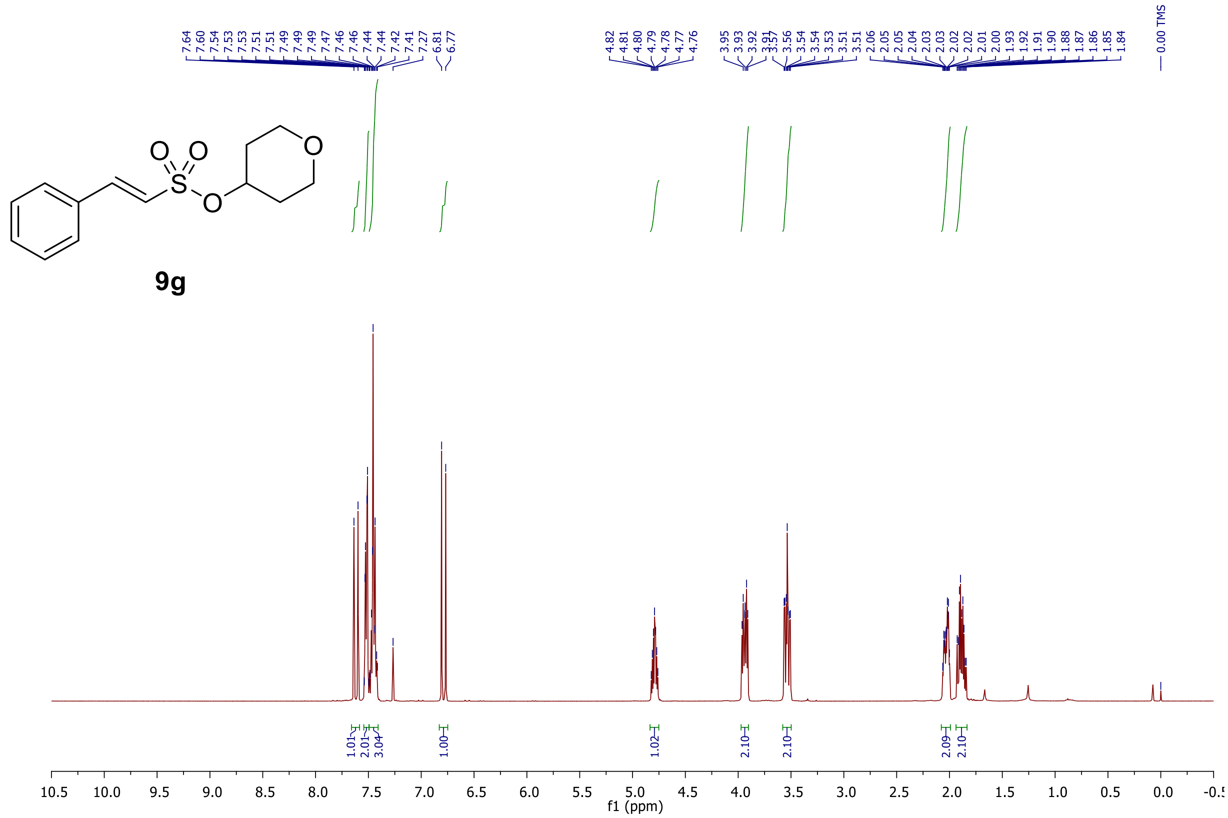

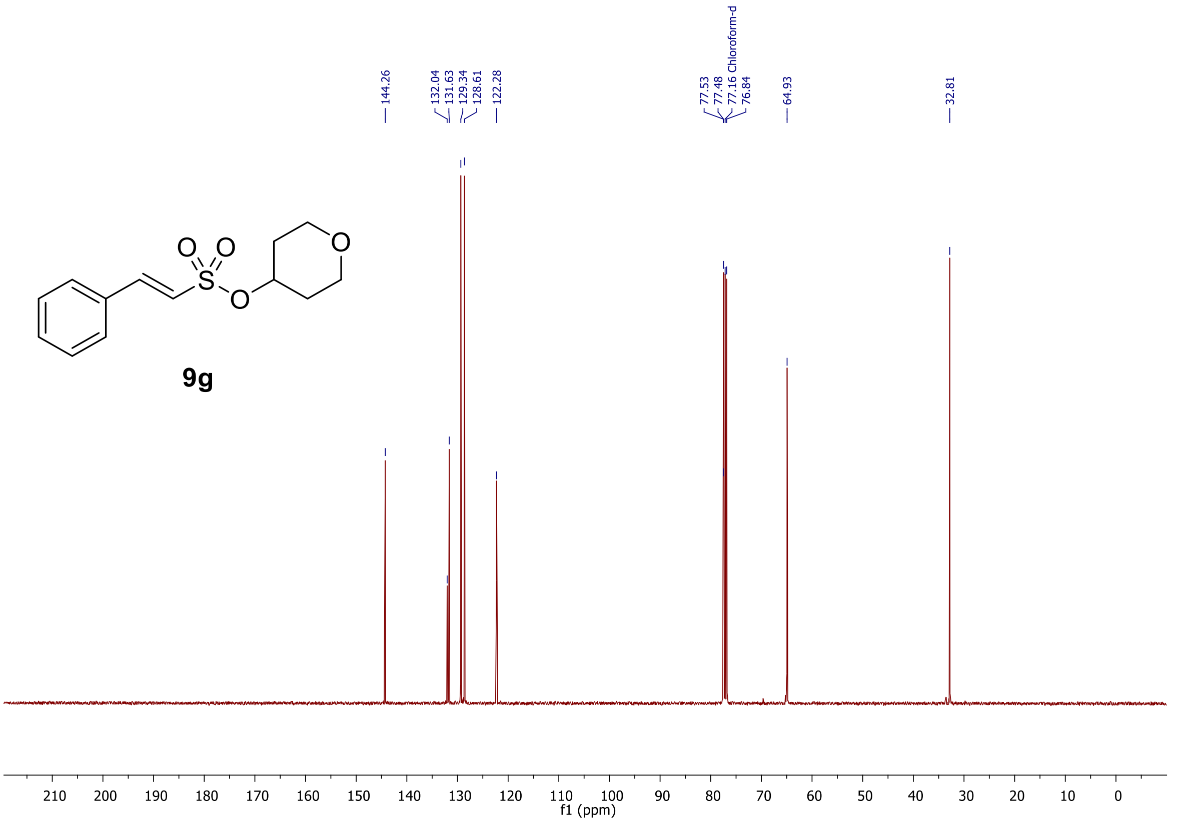

Figure S46: ^{1}H ($CDCl_3$, 400 MHz) and $^{13}C\{^{1}H\}$ ($CDCl_3$, 101 MHz) NMR Spectrum of **9g**.

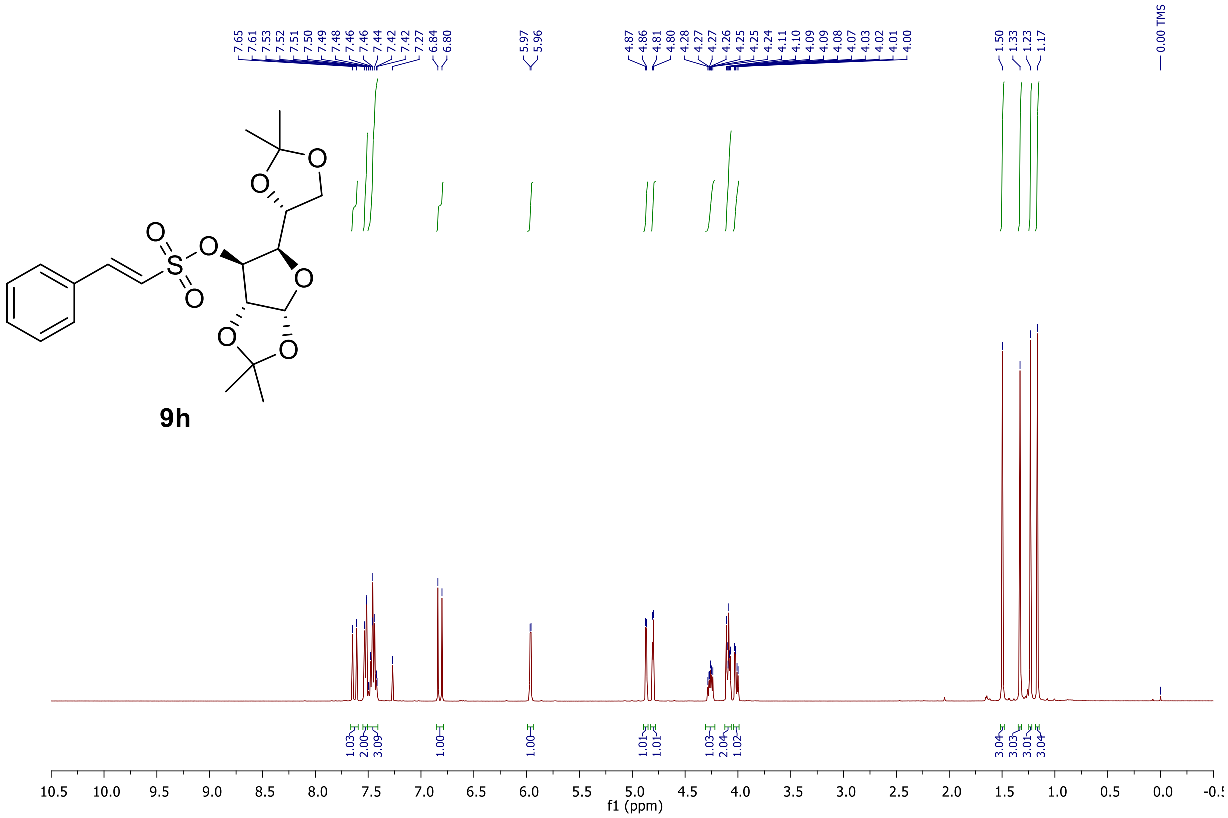

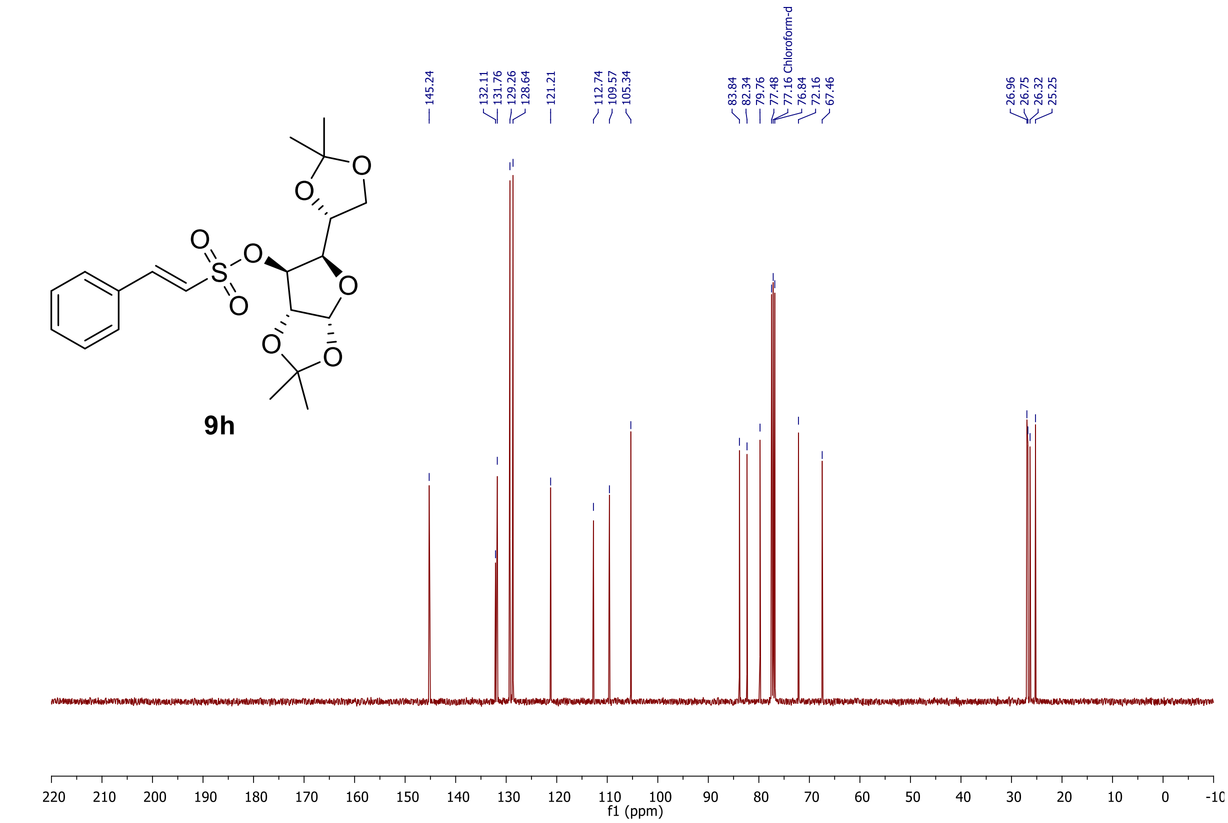

Figure S47: ^{1}H ($CDCl_3$, 400 MHz) and $^{13}C\{^1H\}$ ($CDCl_3$, 101 MHz) NMR Spectrum of **9h**.

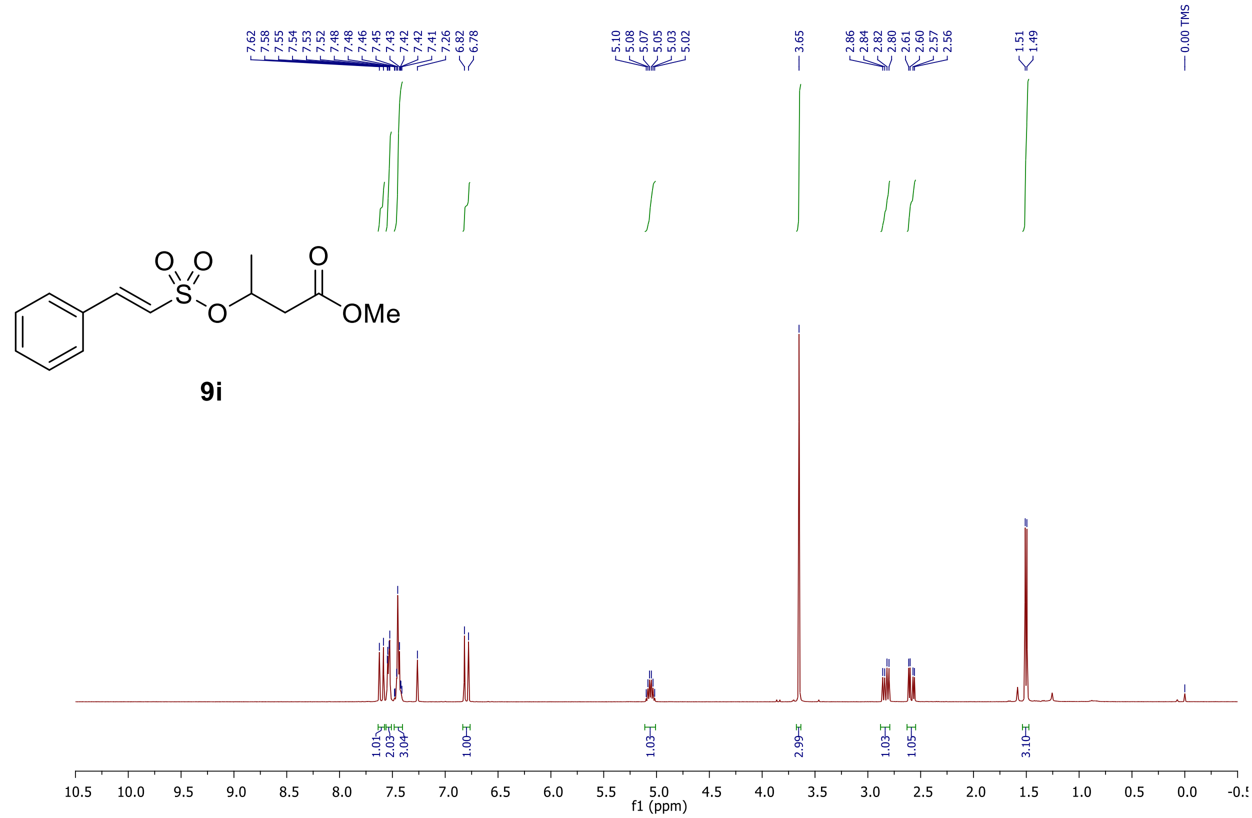

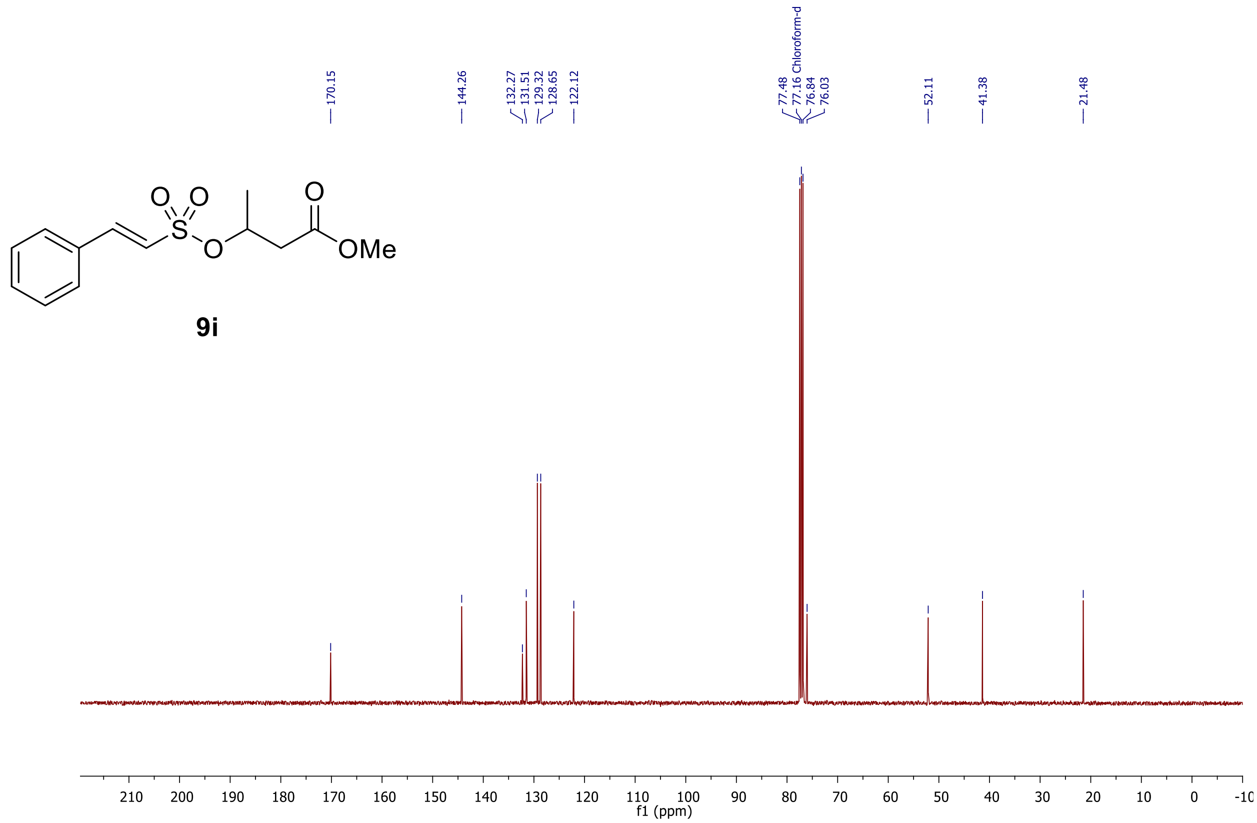

Figure S48: ^{1}H ($CDCl_3$, 400 MHz) and $^{13}C\{^{1}H\}$ ($CDCl_3$, 101 MHz) NMR Spectrum of **9i**.

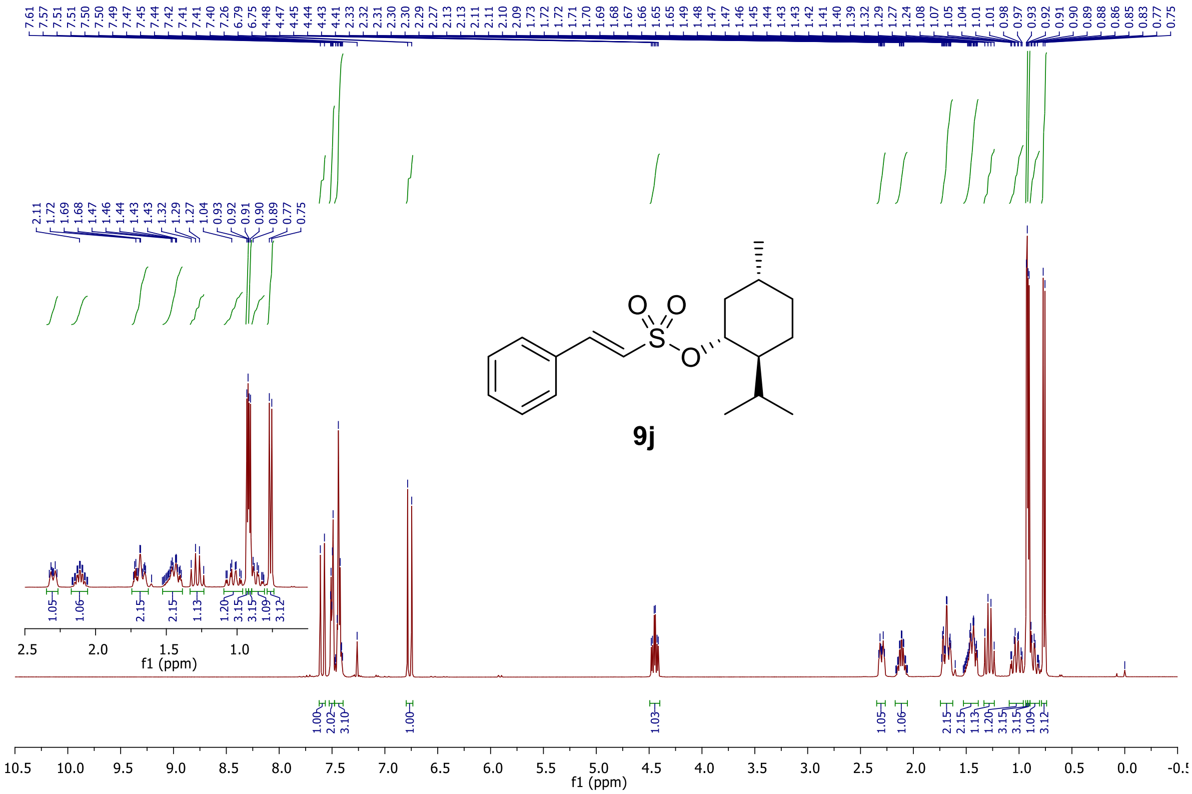

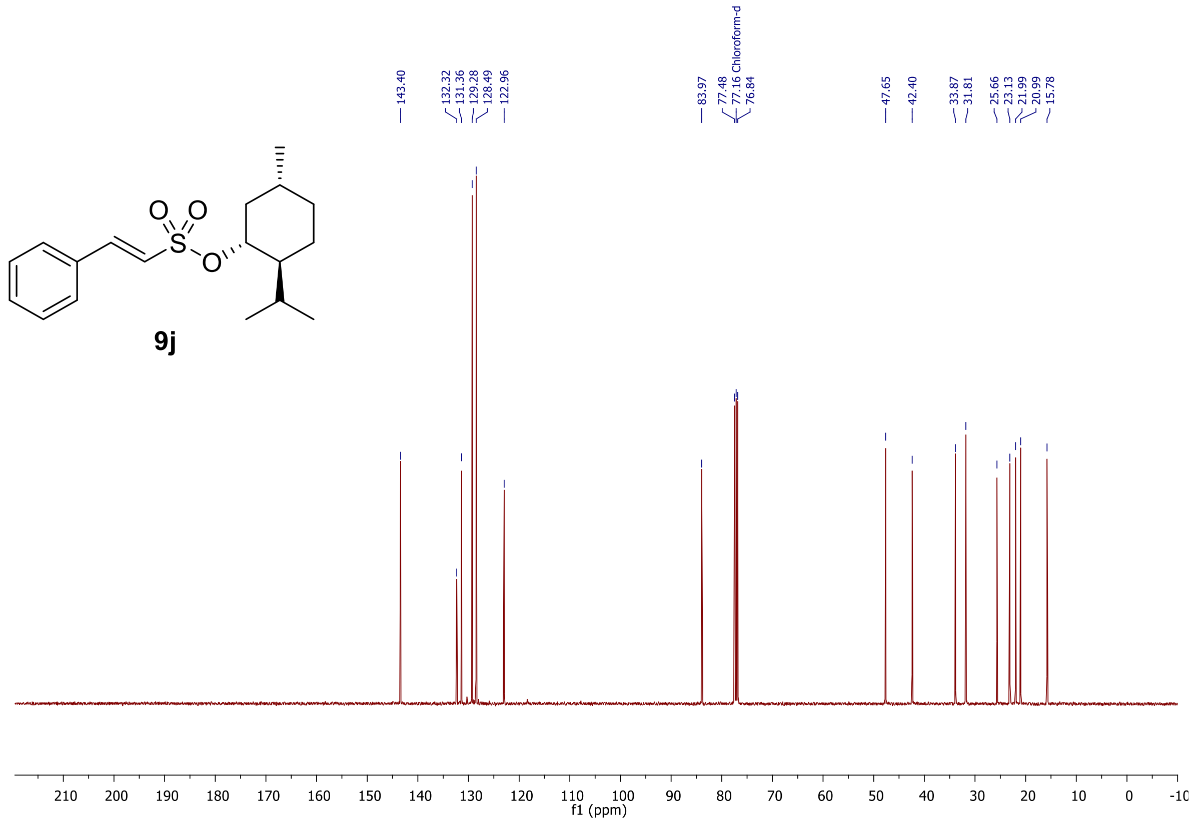

Figure S49: ^{1}H ($CDCl_3$, 400 MHz) and $^{13}C\{^{1}H\}$ ($CDCl_3$, 101 MHz) NMR Spectrum of **9j**.

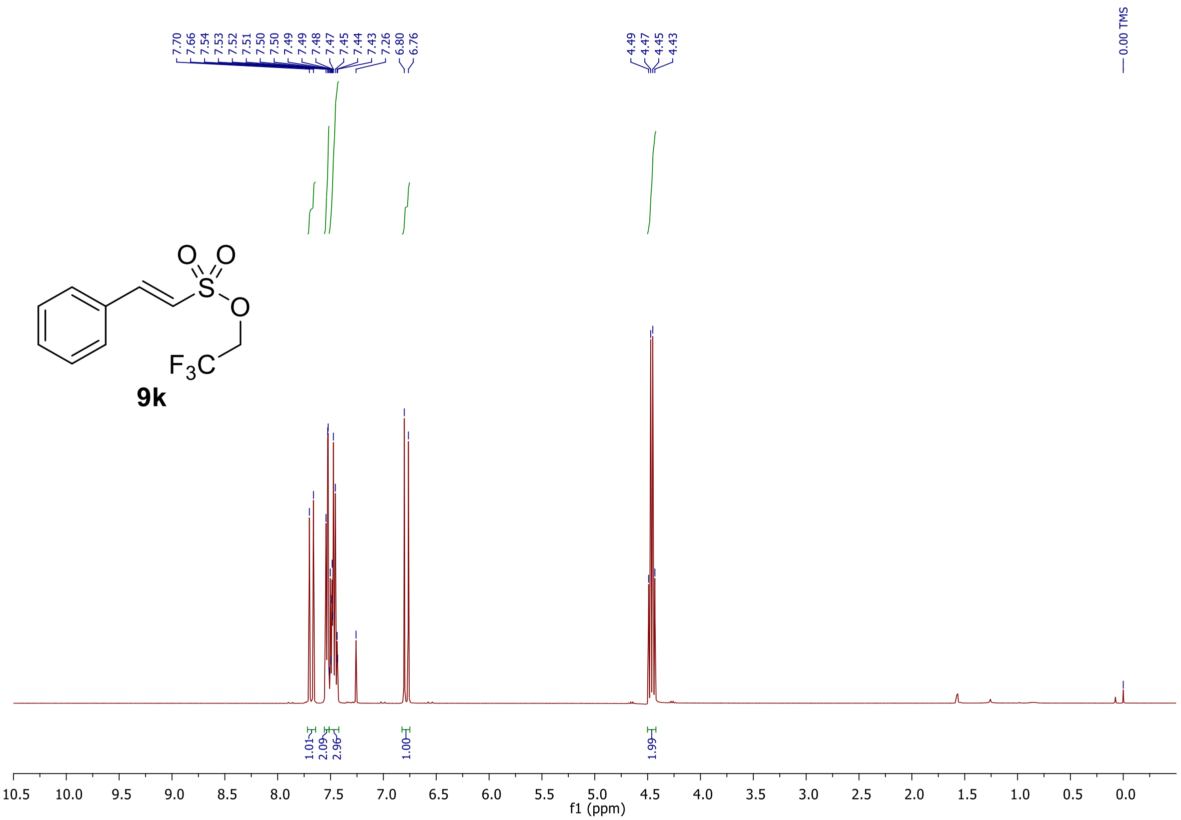
7.70
7.66
7.54
7.53
7.52
7.51
7.50
7.50
7.49
7.49
7.48
7.47
7.45
7.44
7.43
7.26
6.80
6.76
4.49
4.47
4.45
4.43
0.00 TMS
9k
1.01
2.09
2.96
1.00
1.99
f1 (ppm)

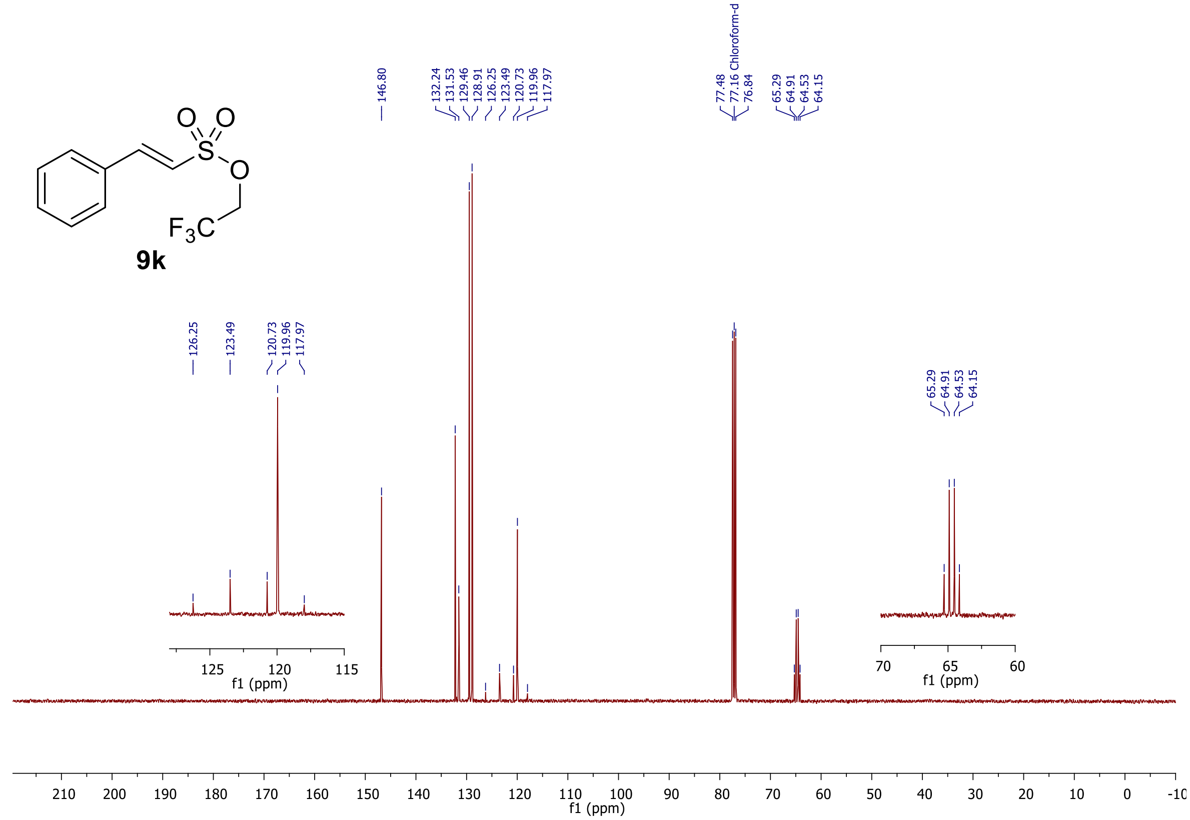
146.80
132.24
131.53
129.46
128.91
126.25
123.49
120.73
119.96
117.97
77.48
77.16 Chloroform-d
76.84
65.29
64.91
64.53
64.15
9k
f1 (ppm)

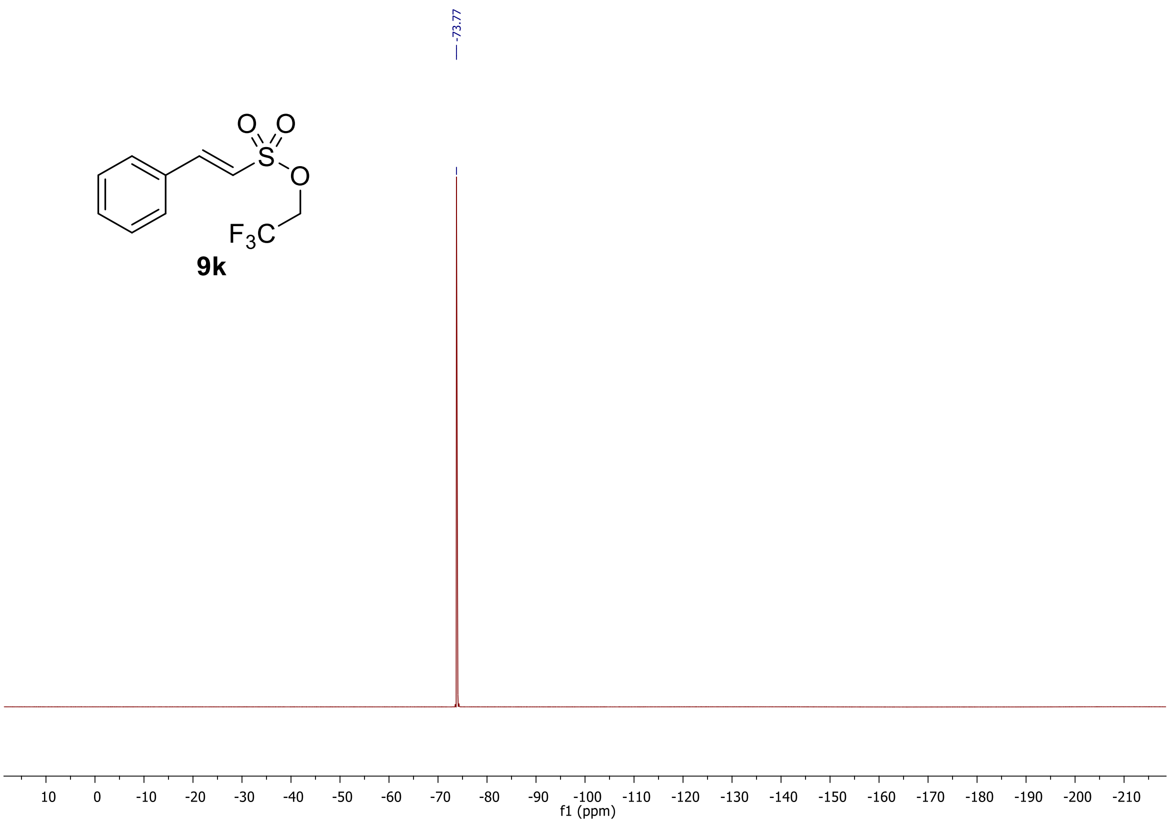

Figure S50: ^{1}H ($CDCl_3$, 400 MHz), $^{13}C\{^{1}H\}$ ($CDCl_3$, 101 MHz), and $^{19}F\{^{1}H\}$ ($CDCl_3$, 376 MHz) NMR Spectrum of **9k**.

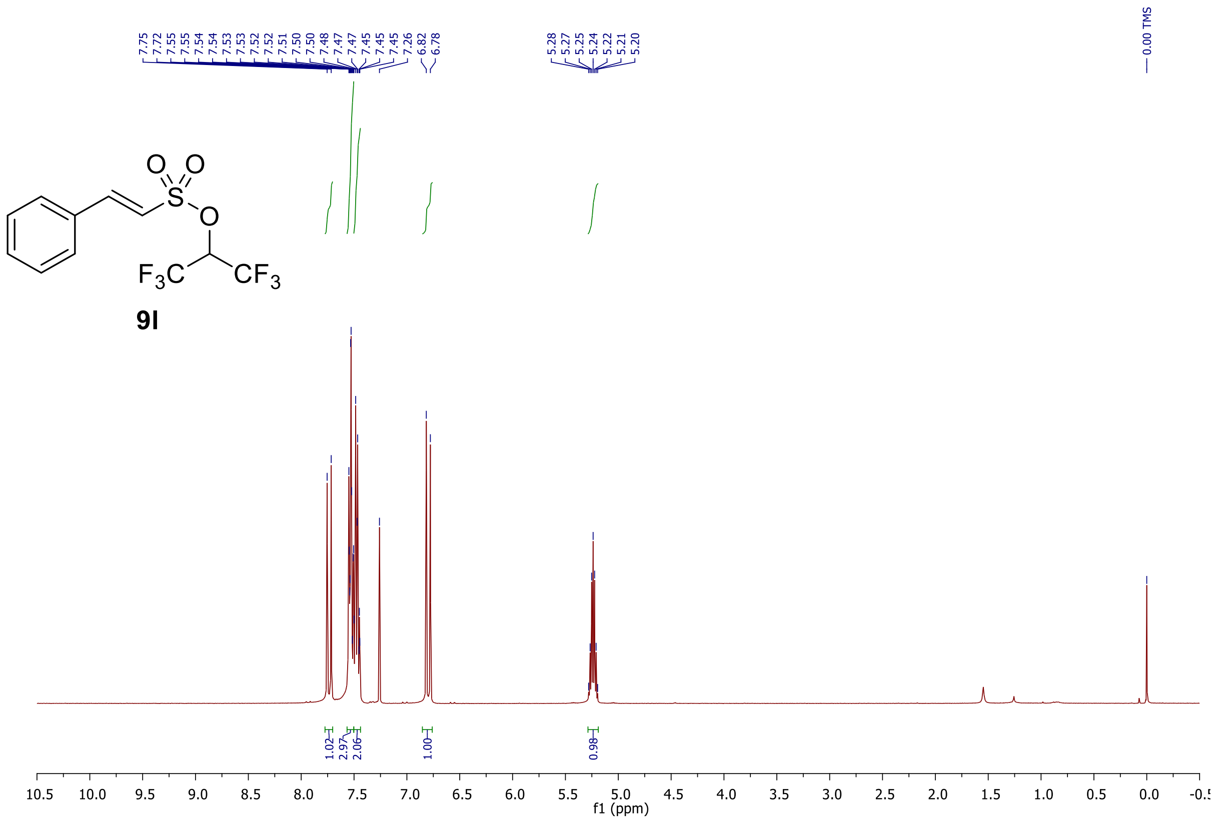
7.75
7.72
7.55
7.55
7.54
7.54
7.53
7.53
7.52
7.52
7.51
7.50
7.50
7.48
7.47
7.47
7.45
7.45
7.45
7.26
6.82
6.78
5.28
5.27
5.25
5.24
5.22
5.21
5.20
0.00 TMS
O
O
S
O
F_3C
CF_3
9l
1.02
2.97
2.06
1.00
0.98
10.5
10.0
9.5
9.0
8.5
8.0
7.5
7.0
6.5
6.0
5.5
5.0
4.5
4.0
3.5
3.0
2.5
2.0
1.5
1.0
0.5
0.0
f1 (ppm)

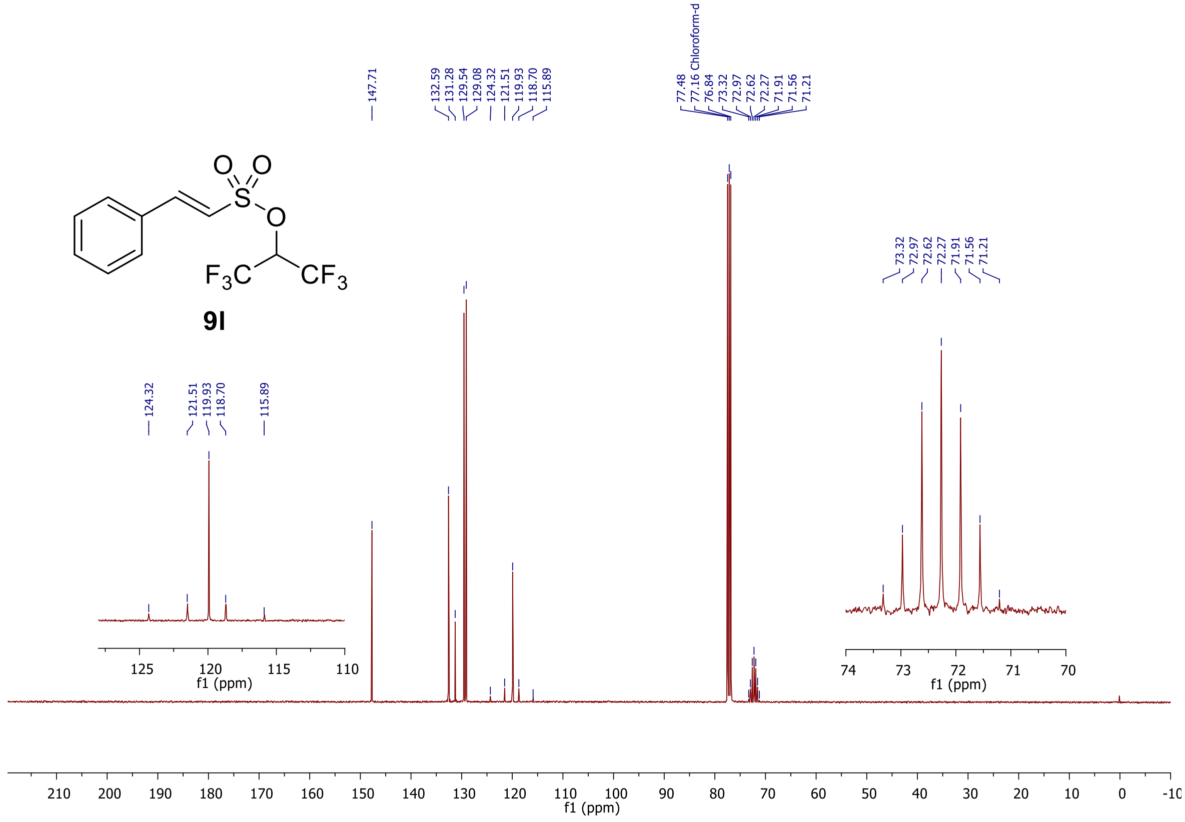
147.71
132.59
131.28
129.54
129.08
124.32
121.51
119.93
118.70
115.89
77.48
77.16 Chloroform-d
76.84
73.32
72.97
72.62
72.27
71.91
71.56
71.21
O
O
S
O
F_3C
CF_3
9l
125
120
115
110
f1 (ppm)
74
73
72
71
70
f1 (ppm)
210
200
190
180
170
160
150
140
130
120
110
100
90
80
70
60
50
40
30
20
10
0
f1 (ppm)

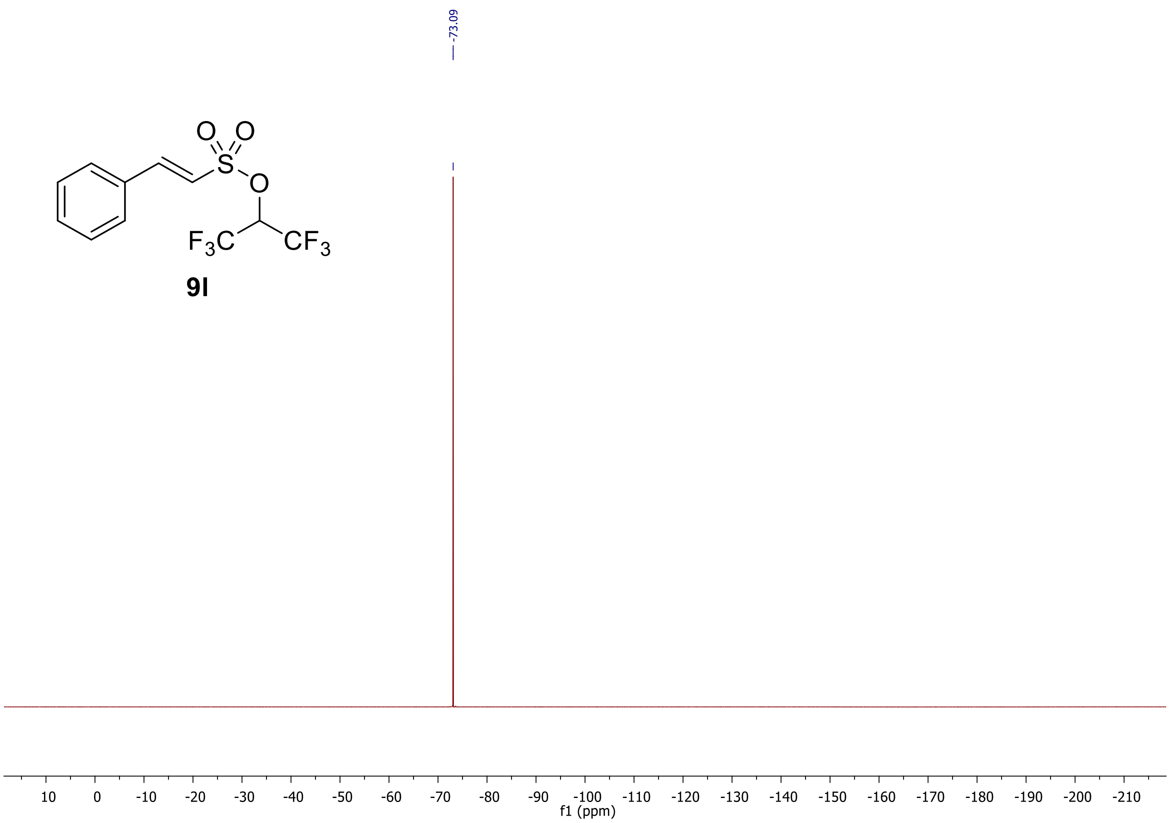

Figure S51: ^{1}H ($CDCl_3$, 400 MHz), $^{13}C\{^{1}H\}$ ($CDCl_3$, 101 MHz), and $^{19}F\{^{1}H\}$ ($CDCl_3$, 376 MHz) NMR Spectrum of **9l**.
